# Comprehensive Effects of Magnesium Supplementation on Cardiometabolic Risk Factors: A Systematic Review and Dose–Response Meta-Analysis

**DOI:** 10.3390/nu18152435

**Published:** 2026-07-25

**Authors:** Shooka Mohammadi, Andrea Palermo, Pantea Ojani, Navid Alaghemand, Pouyan Sanjari Pirayvatlou, Mohammadreza Mirkarimi, Sara Ayazian Mavi, Kia Tahouri, Shokoufeh Shokouhifar, Yeganeh Ettehad, Aida Borzabadi, Damoon Ashtary-Larky, Katsuhiko Suzuki, Cristina Bouzas, Daniela Rodrigues, Josep A. Tur

**Affiliations:** 1 Department of Social and Preventive Medicine, Faculty of Medicine, University of Malaya, Kuala Lumpur 50603, Malaysia; shooka.mohammadi@gmail.com; 2 Unit of Endocrinology and Diabetes, Campus Bio-Medico University, 00128 Rome, Italy; 3 School of Public Health, Boston University, Boston, MA 02118, USA; 4 Nutrition and Metabolic Diseases Research Center, Ahvaz Jundishapur University of Medical Sciences, Ahvaz 6135715794, Iran; 5 Department of Internal Medicine, Saint Agnes Medical Center, Fresno, CA 93720, USA; 6 Department of Pediatrics, Faculty of Medicine, Ahvaz Jundishapur University of Medical Sciences, Ahvaz 6135715794, Iran; 7 Department of Community Medicine, Faculty of Medicine, Ahvaz Jundishapur University of Medical Sciences, Ahvaz 6135715794, Iran; 8 Department of Internal Medicine, Faculty of Medicine, Alborz University of Medical Sciences, Karaj 3149969415, Iran; 9 Faculty of Sport Sciences, Waseda University, Tokorozawa 359-1192, Japan; 10 Research Group on Community Nutrition and Oxidative Stress, University of the Balearic Islands-IUNICS, IDISBA & CIBEROBN (Physiopathology of Obesity and Nutrition CB12/03/30038), Guillem Colom Bldg, 07122 Palma de Mallorca, Spain; 11 Research Centre for Anthropology and Health (CIAS), Department of Life Sciences, University of Coimbra, 3001-401 Coimbra, Portugal; drdc@uc.pt

**Keywords:** magnesium, glycemic profile, cardiometabolic risk factors, blood pressure, inflammation, lipid profile

## Abstract

**Background:** Evidence regarding the impacts of magnesium (Mg) supplementation on cardiometabolic risk factors (CMRFs) remains inconsistent. **Objectives:** This systematic review and dose–response meta-analysis evaluated effects of Mg supplementation on anthropometric indices, liver and kidney function, lipid and glycemic profiles, blood pressure, and inflammatory biomarkers. **Methods:** A systematic search of electronic databases up to May 2026 identified 78 eligible randomized controlled trials. **Results:** Mg supplementation significantly reduced body weight (weighted mean difference [WMD]: −0.70 kg; 95% confidence interval [CI]: −1.30, −0.09), diastolic blood pressure (WMD: −1.58 mmHg; 95% CI: −2.50, −0.65), homeostasis model assessment of insulin resistance (WMD: −0.48; 95% CI: −0.79, −0.18), low-density lipoprotein cholesterol (WMD: −3.21 mg/dL; 95% CI: −5.27, −1.15), fasting blood glucose (WMD: −3.60 mg/dL; 95% CI: −6.13, −1.06), systolic blood pressure (WMD: −2.50 mmHg; 95% CI: −4.08, −0.91), glycated hemoglobin (WMD: −0.15%; 95% CI: −0.26, −0.03), triglycerides (WMD: −9.24 mg/dL; 95% CI: −16.87, −1.61), and interleukin-6 levels (WMD: −1.10 pg/mL; 95% CI: −1.93, −0.27) compared with controls. High-density lipoprotein cholesterol concentrations significantly increased (WMD: 1.45 mg/dL; 95% CI: 0.37, 2.54). No significant effects were identified on hip circumference, alanine aminotransferase, waist circumference, tumor necrosis factor-α, creatinine, C-reactive protein, body mass index, total cholesterol, fasting insulin, body fat percentage, and aspartate aminotransferase. Most RCTs (79.5%) administered Mg doses ≥ 300 mg/day, and 66.7% had intervention durations ≥ 12 weeks; however, evidence from higher-dose (≥500 mg/day) and longer-term (≥25 weeks) interventions remained limited. **Conclusions:** Mg supplementation was associated with significant improvements in several CMRFs, including body weight, lipid and glycemic profiles, blood pressure, and interleukin-6 levels. However, these effects were generally modest, and their clinical relevance remains uncertain given the variable certainty of evidence across outcomes.

## 1. Introduction

Cardiometabolic risk (CMR) refers to the coexistence of metabolic and cardiovascular abnormalities that collectively increase the risk of developing dyslipidemia, hypertension (HTN), metabolic syndrome (MetS), cardiovascular disease (CVD), and type 2 diabetes mellitus (T2DM) [1]. The burden of cardiometabolic disorders (CMDs) has increased substantially over recent decades and now represents a major global public health challenge [2,3,4]. CVDs remain among the leading causes of disability and mortality worldwide, and the prevalence of MetS is increasing across populations [3,4]. Underlying CMDs are multifactorial processes involving genetic, lifestyle, and metabolic factors, with chronic low-grade systemic inflammation and oxidative stress (OS) acting as central drivers of endothelial dysfunction, insulin resistance (IR), lipid abnormalities, and vascular injury [5,6,7,8,9]. In addition, elevated liver enzymes and impaired renal function are recognized indicators of metabolic dysfunction and systemic inflammation associated with adverse cardiometabolic outcomes [10,11]. Given the growing global burden and clinical consequences of CMDs, increasing attention has been directed toward nutritional and dietary interventions as complementary strategies to improve cardiometabolic health (CMH) [12,13,14,15,16,17,18,19,20].

Among dietary micronutrients, magnesium (Mg) has emerged as an important factor because of its involvement in numerous physiological and biochemical processes related to CMH [21,22,23,24]. Mg is the fourth most prevalent mineral in the human body and participates as a cofactor in over 300 enzymatic reactions involved in glucose homeostasis, lipid metabolism, adenosine triphosphate (ATP) production, protein synthesis, vascular tone regulation, neuromuscular function, and maintenance of electrolyte balance [21,22,23]. Dietary sources of Mg include nuts, legumes, whole grains, seeds, and green leafy vegetables [25]; however, inadequate Mg intake has become increasingly common because of poor dietary quality and widespread consumption of processed foods [21,26]. It has been estimated that a substantial proportion of adults worldwide do not meet the recommended dietary intake of Mg [26,27]. Emerging evidence also suggests that Mg deficiency may contribute to IR, endothelial dysfunction, OS, chronic low-grade inflammation, and abnormal lipid metabolism, all of which are key mechanisms underlying CMDs [5,6,7,21,22,28]. It has been reported that low dietary or circulating Mg levels are related to increased risks of CVD, T2DM, MetS, and HTN [29,30,31,32,33]. In addition, previous meta-analyses and randomized controlled trials (RCTs) have suggested that Mg supplementation may improve several cardiometabolic parameters [34,35,36,37,38,39,40,41,42,43,44,45,46,47,48,49].

Multiple systematic reviews and meta-analyses have assessed the effects of Mg supplementation on individual cardiometabolic parameters, including blood pressure (BP) [38,50,51,52,53], lipid profiles [39,54,55], glycemic parameters [35,44,45,46,47], anthropometric indices [48,49], and inflammatory biomarkers [36,56,57]. Despite the evidence provided by these studies, their findings are largely confined to single cardiometabolic domains and do not reflect the interconnected nature of cardiometabolic risk factors (CMRFs). Notably, no previous meta-analysis has comprehensively evaluated the integrated impacts of Mg supplementation across a broad spectrum of interconnected CMRFs, including liver and renal function biomarkers, lipid and glycemic profiles, inflammatory markers, and anthropometric indices, within a single analytical framework. These limitations, together with substantial between-study heterogeneity in participant characteristics, bioavailability and chemical formulation of Mg, supplementation dose, and intervention duration, which may affect supplementation efficacy, have contributed to the lack of consensus regarding the overall impact of Mg supplementation on CMH. Given that the effects of Mg supplementation may vary according to dosage, duration, and formulation, a comprehensive dose–response assessment is warranted. Therefore, the current systematic review and dose–response meta-analysis of RCTs aimed to provide updated and methodologically rigorous evidence on the impacts of supplementation with Mg on major CMRFs.

## 2. Materials and Methods

This study was designed and reported based on the PRISMA 2020 recommendations for systematic reviews and meta-analyses [58]. Methodological procedures were also guided by the Cochrane Handbook for Systematic Reviews of Interventions [59]. Before conducting the study, the protocol was registered in the PROSPERO database (registration ID: CRD420261328217).

### 2.1. Search Strategy

Two reviewers independently conducted a systematic search of Scopus, PubMed/MEDLINE, and Web of Science from their inception until May 2026 to identify eligible RCTs. To maximize retrieval of relevant studies, gray literature sources, including major clinical trial registries and Google Scholar, were also searched. Additionally, the reference lists of relevant review articles were manually examined to find any further eligible RCTs. No language or publication date restrictions were applied.

The search strategy was developed according to the PICOS framework, encompassing the target population (adults), intervention (Mg supplementation), comparator (placebo or control group), outcomes (CMRFs), and study design (RCTs). Outcomes of interest included anthropometric indices, indicators of kidney and liver function, lipid and glycemic profiles, BP, and inflammatory biomarkers. Lipid profile parameters included high-density lipoprotein cholesterol (HDL-C), total cholesterol (TC), low-density lipoprotein cholesterol (LDL-C), and triglycerides (TG). Anthropometric variables comprised body mass index (BMI), waist circumference (WC), hip circumference (HC), body weight (BW), and body fat percentage (BFP). Glycemic outcomes included fasting insulin (FI), hemoglobin A1c (HbA1c), fasting blood glucose (FBG), and homeostatic model assessment of insulin resistance (HOMA-IR). Blood pressure outcomes included diastolic blood pressure (DBP) and systolic blood pressure (SBP). Inflammatory markers included interleukin-6 (IL-6), C-reactive protein (CRP), and tumor necrosis factor-α (TNF-α). Kidney function was assessed using serum creatinine (Cr) levels, and liver function markers included serum alanine aminotransferase (ALT) and aspartate aminotransferase (AST). The search strategy was tailored to each database by combining Medical Subject Headings (MeSH) terms with relevant free-text terms, connected with Boolean operators (“AND” and “OR”). The complete PubMed search syntax is provided in Table S1.

### 2.2. Eligibility Criteria and Study Selection

EndNote software (version 21) was utilized to organize the retrieved citations and remove duplicate records. Title screening, abstract, and full-text evaluations were conducted independently by two researchers using predefined eligibility criteria. Differences in reviewers’ decisions were addressed through discussion with a third reviewer until agreement was reached.

Trials were eligible if they met the following criteria: (1) RCT design with either parallel or crossover allocation; (2) evaluation of Mg supplementation compared with placebo or control; (3) a minimum intervention period of at least two weeks; and (4) provision of post-intervention and baseline data for at least one cardiometabolic outcome in both study arms. Studies were removed if they were non-randomized, lacked a placebo/control group, administered Mg as part of a multi-nutrient intervention, enrolled pregnant participants, or included individuals younger than 18 years.

### 2.3. Data Extraction

Two investigators independently extracted the data using a standardized, pilot-tested Excel form, and discrepancies were resolved through agreement with a third investigator. The following data were extracted from each eligible study: study design, sample size, study location, publication year, first author, intervention duration, Mg dosage, chemical formulation of Mg supplement, participant characteristics (age, sex, and mean BMI), and outcome measurements at post-intervention and baseline. In cases where outcome data were incomplete, unclear, or unavailable in the published article, the corresponding author was contacted for additional information.

### 2.4. Risk of Bias Assessment

Two independent researchers evaluated the risk of bias (RoB) in the included trials using the revised Cochrane RoB2 tool [60]. Any disagreements between them were settled through discussion with a third investigator. The RoB assessment examined five domains, including bias arising from the randomization process, deviations from intended interventions, missing outcome data, measurement of the outcome, and selection of the reported result. The RoB within each domain was evaluated and classified into one of three levels: low, some concerns, or high.

### 2.5. Certainty of Evidence

Evidence certainty was assessed using the Grading of Recommendations Assessment, Development, and Evaluation (GRADE) framework [61]. Ratings ranged from high to very low and were based on assessments of RoB, inconsistency, indirectness, imprecision, and publication bias.

### 2.6. Statistical Analysis

Statistical analyses were performed using STATA 17 software (StataCorp, College Station, TX, USA). Effect sizes were derived from the reported changes in means and their associated standard deviations from each study [62]. The pooled results were reported as weighted mean differences (WMDs) and 95% confidence intervals (CIs) comparing baseline-to-endpoint changes between the Mg and control groups. For crossover trials, paired analyses were used whenever reported. As the within-participant correlation coefficient required for paired analyses was not reported in the included crossover trials, participant numbers were halved to approximate the effective sample size when paired analyses were unavailable. For multi-arm parallel trials with a shared comparison group, participants in the control group were divided approximately equally across the relevant pairwise comparisons. Considering the expected variability across studies in both methodology and clinical features, pooled analyses were performed using a random-effects approach [62]. Between-study heterogeneity was evaluated using Cochran’s Q test and quantified using the I^2^ statistic. Heterogeneity was interpreted as moderate (26–50%), low (≤25%), high (51–75%), or very high (>75%) [63].

Mg supplementation interventions were classified by both the chemical form of Mg and the estimated oral bioavailability. The Mg formulations were categorized into four groups: organic, inorganic, chelated, and mixed. Organic forms included Mg citrate, lactate, gluconate, aspartate, pidolate, and related organic salts. Inorganic forms included Mg oxide, chloride, sulfate, hydroxide, bicarbonate, and other inorganic salts. Chelated formulations comprised amino acid–bound Mg compounds, such as Mg diglycinate and other chelated forms, whereas mixed formulations referred to interventions containing two or more Mg salts. A secondary classification based on the estimated relative bioavailability of Mg formulations was also performed. The categories were defined a priori based on available evidence regarding the gastrointestinal solubility and intestinal absorption of different oral Mg formulations, which together influence their relative bioavailability, as reported in published studies [64,65,66]. Mg citrate, lactate, gluconate, aspartate, pidolate, and chelated formulations were considered to have high bioavailability, whereas Mg chloride, sulfate, and bicarbonate were categorized as having moderate bioavailability. Mg oxide and hydroxide were considered to have low bioavailability. Interventions that included multiple Mg formulations with different estimated bioavailability profiles were classified as having mixed bioavailability.

Subgroup analyses were additionally performed to investigate probable factors contributing to between-study heterogeneity. Analyses were stratified according to baseline LDL-C, DBP, HDL-C, SBP, TG, FBG, and TC levels; Mg dosage (≥300 mg/day vs. <300 mg/day); intervention duration (≥12 weeks vs. <12 weeks); Mg formulation (organic, inorganic, chelated, or mixed); bioavailability of Mg form (high, moderate, low, or mixed); BMI category (normal weight, overweight, or obese); age (≥50 years vs. <50 years); and sex (both sexes, male, or female).

A leave-one-out sensitivity analysis was applied to determine whether the overall findings were influenced by any single study through sequential omission of each study. Probable publication bias was evaluated through funnel plot assessment as well as Egger’s and Begg’s tests [67,68]. Possible nonlinear dose–response associations between Mg dosage or intervention period and study outcomes were investigated using fractional polynomial models, whereas linear associations were evaluated using meta-regression analyses [69]. Statistical significance was established using a two-tailed significance level of 0.05.

## 3. Results

### 3.1. Study Selection

**Figure 1:**
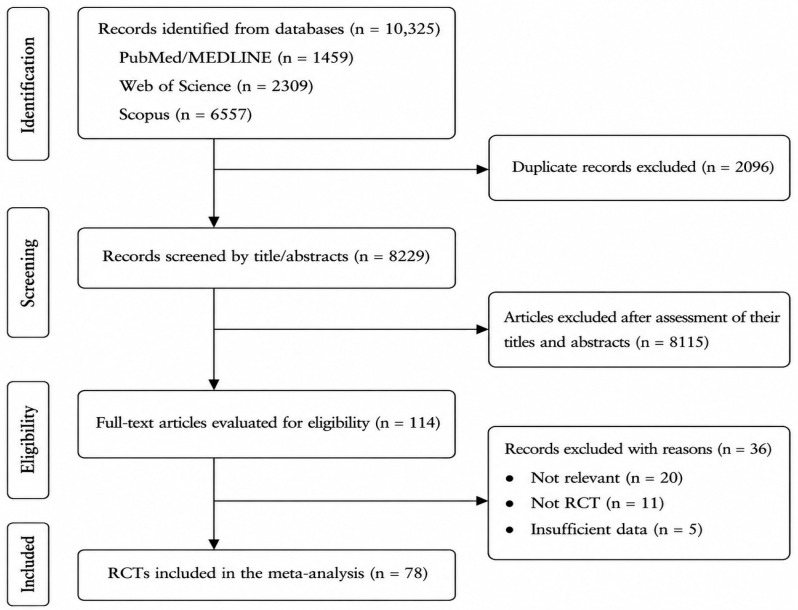
Flow diagram of study selection.

A total of 10,325 citations were retrieved from the electronic databases. After the removal of 2096 duplicates, 8229 articles were screened, and 8115 were excluded based on the eligibility criteria. Following full-text evaluations of 114 trials, 78 RCTs [70,71,72,73,74,75,76,77,78,79,80,81,82,83,84,85,86,87,88,89,90,91,92,93,94,95,96,97,98,99,100,101,102,103,104,105,106,107,108,109,110,111,112,113,114,115,116,117,118,119,120,121,122,123,124,125,126,127,128,129,130,131,132,133,134,135,136,137,138,139,140,141,142,143,144,145,146,147] were included in the current meta-analysis. Figure 1 displays the study selection process.

### 3.2. Study Characteristics

This systematic review and dose–response meta-analysis included 78 RCTs [70,71,72,73,74,75,76,77,78,79,80,81,82,83,84,85,86,87,88,89,90,91,92,93,94,95,96,97,98,99,100,101,102,103,104,105,106,107,108,109,110,111,112,113,114,115,116,117,118,119,120,121,122,123,124,125,126,127,128,129,130,131,132,133,134,135,136,137,138,139,140,141,142,143,144,145,146,147]. The characteristics of trials are shown in Table 1. Of these studies, 67 implemented a parallel design [70,71,72,73,74,75,79,80,81,82,83,85,86,87,88,89,90,91,92,93,94,95,96,97,98,99,100,101,102,103,104,105,106,108,109,110,112,115,116,117,118,119,120,121,122,123,124,125,126,127,129,130,131,132,133,134,135,136,137,138,139,142,143,144,145,146,147], and 11 used a crossover design [76,77,78,84,107,111,113,114,128,140,141]. A total of 5226 participants were included (Mg group, *n* = 2625; control group, *n* = 2601). The sample sizes of the RCTs varied from 13 to 461. The mean age of participants across studies was 23–73 years, while the mean BMI ranged from 22 to 39 kg/m^2^. Most trials included mixed-sex participants [70,71,72,74,75,76,77,81,83,84,85,86,87,89,90,91,92,93,94,95,97,98,99,100,101,102,103,104,105,106,107,108,109,111,112,113,114,115,117,118,119,120,121,122,123,124,126,127,128,129,131,132,133,134,135,136,137,138,140,141,143,144,145,146], while ten included only females [73,79,80,82,88,96,110,125,130,142], and four only males [78,116,139,147].

The included trials enrolled participants with diverse clinical characteristics, including patients with T2DM [72,74,81,83,84,86,87,90,102,111,113,114,118,120,136], non-insulin-dependent diabetes mellitus (NIDDM) [94], MetS [71,82,96,103,122], polycystic ovary syndrome (PCOS) [73,88,130], HTN [75,76,79,85,89,90,97,105,112,124,128,138,140,141,142,146], IR [92], chronic obstructive pulmonary disease (COPD) [145], asthma [98], and nonalcoholic fatty liver disease (NAFLD) [100]. Other clinical conditions included coronary artery disease (CAD) [116,131,134], chronic kidney disease (CKD) [137], diabetic nephropathy (DN) [95,126], ischemic heart disease (IHD) [119], and heart failure (HF) [85]. In addition, RCTs included individuals with prediabetes [91,127,132,133,137,144], overweight or obesity [70,77,99,104,108,110,129,135,137], hypomagnesemia [91], pre-HTN [123], metabolically obese normal weight (MONW) individuals [121], women with low habitual Mg intake [125], healthy participants [78,93,98,106,107,139,143], postmenopausal women [80], endurance-trained athletes [147], hemodialysis patients [109], individuals with depression and Mg deficiency [117], alcohol use disorder [115], and insomnia [102].

The articles were published between 1983 and 2026. The trials were conducted in multiple countries, including Sweden [85,105,124,140,141], the Netherlands [83,84,99,129,142], Iran [70,73,88,100,109,110,117,118,126,127,130,134,135,136], Mexico [74,90,91,92,93,111,120,121,122,123,132,133], Italy [75,78,89,113,114,145], Brazil [79,81,82,128], the United States of America (USA) [77,101,106,107,125,131,143,146], the United Kingdom (UK) [76,138], Germany [71,103,108], Saudi Arabia [72], Australia [80,112], Austria [86,116], Denmark [97,119], Egypt [95], Finland [115], France [139], Greece [147], Iraq [96], Japan [98], Norway [94], Pakistan [102], Palestine [87], China [144], South Korea [104], and Turkey [137].

Across the included RCTs, Mg doses varied from 20 to 1782 mg/day, and intervention durations ranged from 3 to 52 weeks. Sixty-two of 78 included trials (79.5%) administered Mg doses ≥ 300 mg/day, whereas 16 studies (20.5%) evaluated doses < 300 mg/day. Among these 62 RCTs, 20 (25.6% of all included trials) investigated higher Mg doses (≥500 mg/day). Regarding intervention duration, 52 studies (66.7%) lasted ≥12 weeks, whereas 26 (33.3%) had durations < 12 weeks. Among the included trials, only three (3.8%) evaluated longer-term interventions (≥25 weeks).

### 3.3. Meta-Analysis

**Figure 2:**
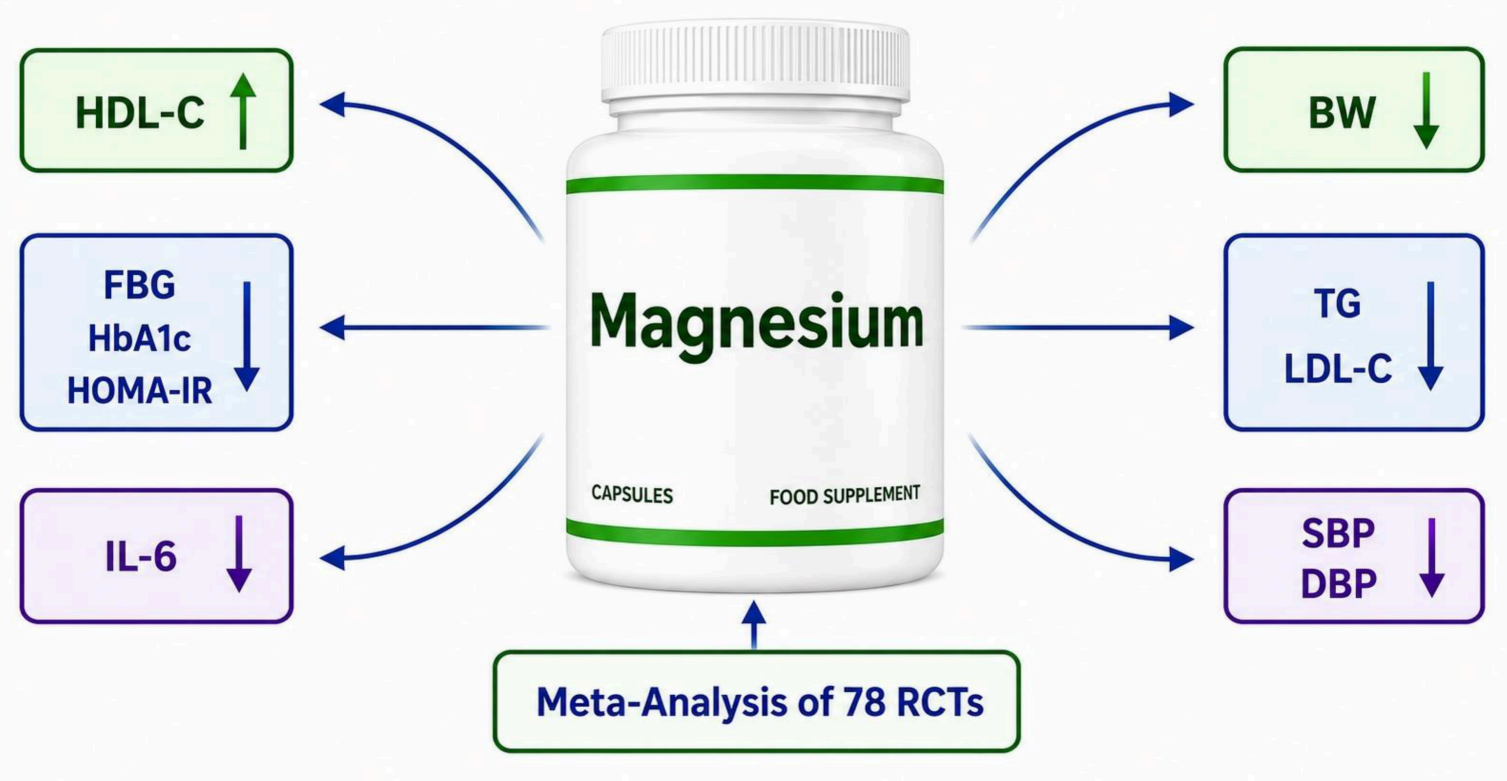
Overview of the impacts of Mg supplementation on CMRFs based on the meta-analysis of 78 RCTs. Arrows show the direction of change (↓ reduction, ↑ increase).

An overview of the meta-analysis results is presented in Table 2 and Figure 2. The assessed outcomes included glycemic markers (*n* = 4), anthropometric indices (*n* = 5), hepatic and renal function markers (*n* = 3), lipid profile parameters (*n* = 4), BP indices (*n* = 2), and inflammatory biomarkers (*n* = 3).

#### 3.3.1. Effects of Mg Supplementation on Anthropometric Measures

**Figure 3:**
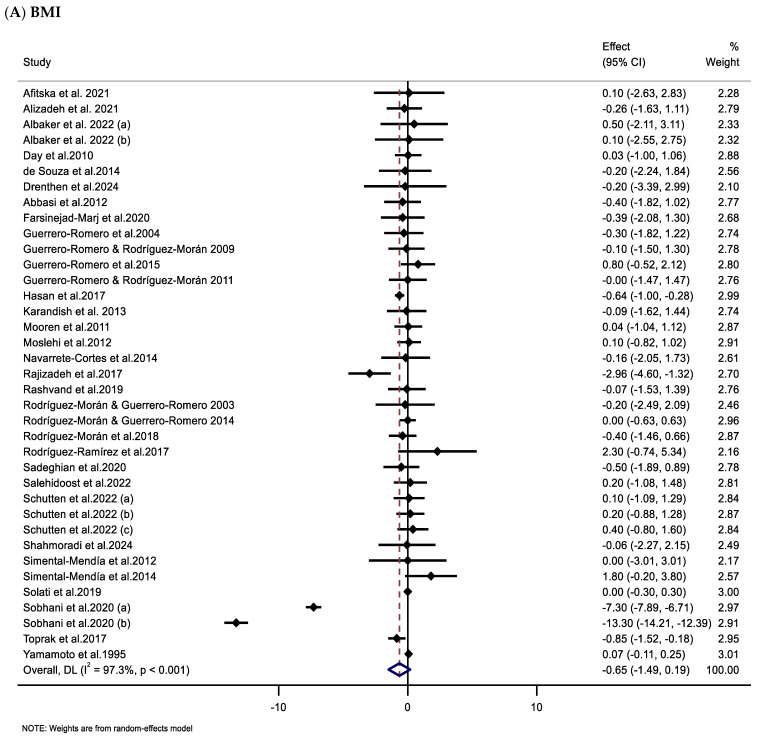

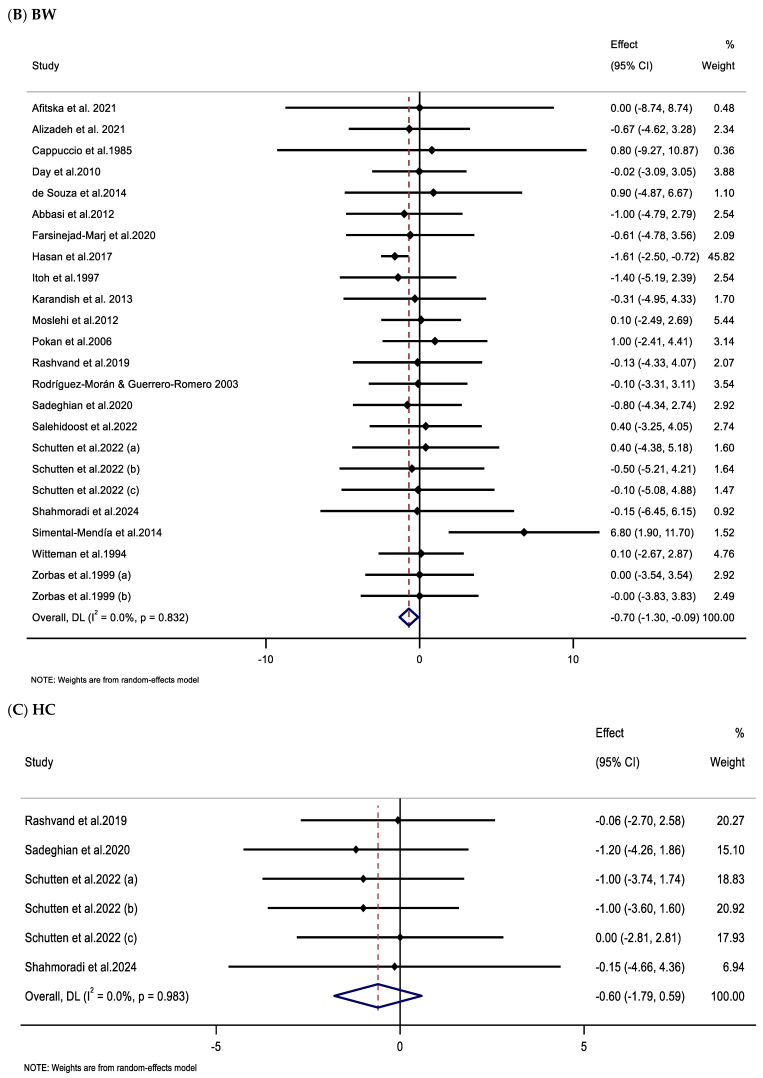

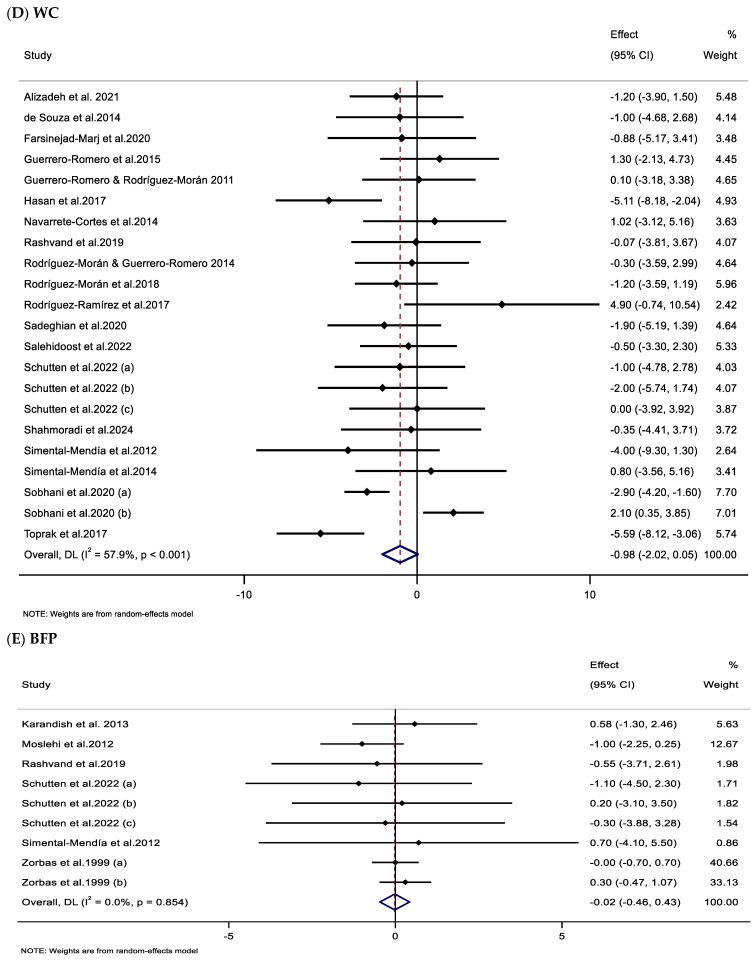
Forest plots present WMDs and 95% CIs for the impacts of Mg supplementation on anthropometric indices, including (**A**) BMI (kg/m^2^), (**B**) BW (kg), (**C**) HC (cm), (**D**) WC (cm), and (**E**) BFP (%) [70,71,72,73,76,80,82,84,88,90,91,92,93,96,98,100,108,110,111,116,117,118,120,121,122,123,126,127,129,130,132,133,134,135,137,142,143,147].

This meta-analysis of RCTs suggested that Mg supplementation was associated with a significant reduction in BW (WMD: −0.70 kg, 95% CI: −1.30, −0.09) compared with placebo or control groups. In contrast, no substantial differences were observed in HC (WMD: −0.60 cm, 95% CI: −1.79, 0.59), WC (WMD: −0.98 cm, 95% CI: −2.02, 0.05), BMI (WMD: −0.65 kg/m^2^, 95% CI: −1.49, 0.19), or BFP (WMD: −0.02%, 95% CI: −0.46, 0.43) (Figure 3 and Table 2).

Subgroup analyses indicated that reductions in BW were observed among participants who received Mg supplementation in studies with intervention durations < 12 weeks, Mg doses < 300 mg/day, organic Mg formulations, high Mg bioavailability, female participants, and individuals aged ≥50 years. BMI was significantly decreased in RCTs that used Mg doses < 300 mg/day, organic Mg formulations, and intervention durations < 12 weeks, particularly among women and individuals with obesity. Significant reductions in WC were observed in RCTs that provided Mg doses < 300 mg/day and used lower-bioavailability Mg formulations, particularly among female participants (Table S2).

#### 3.3.2. Effects of Mg Supplementation on Glycemic Profile

**Figure 4:**
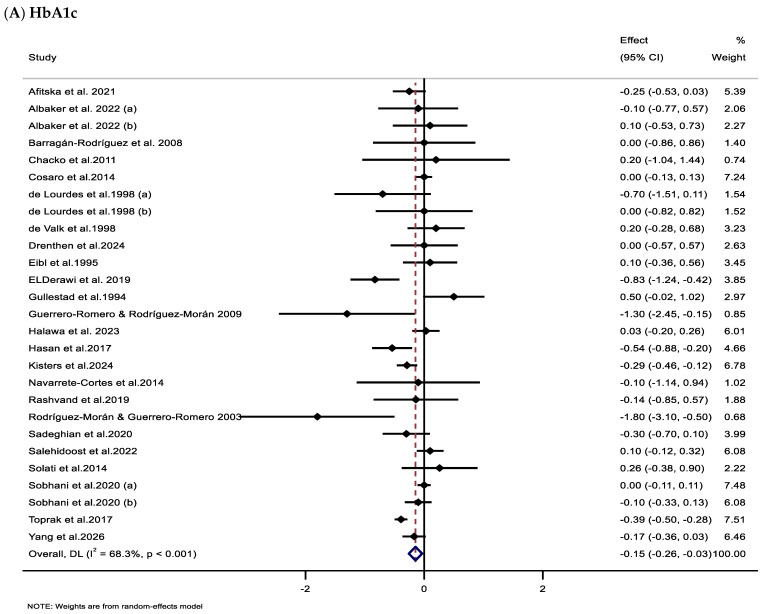

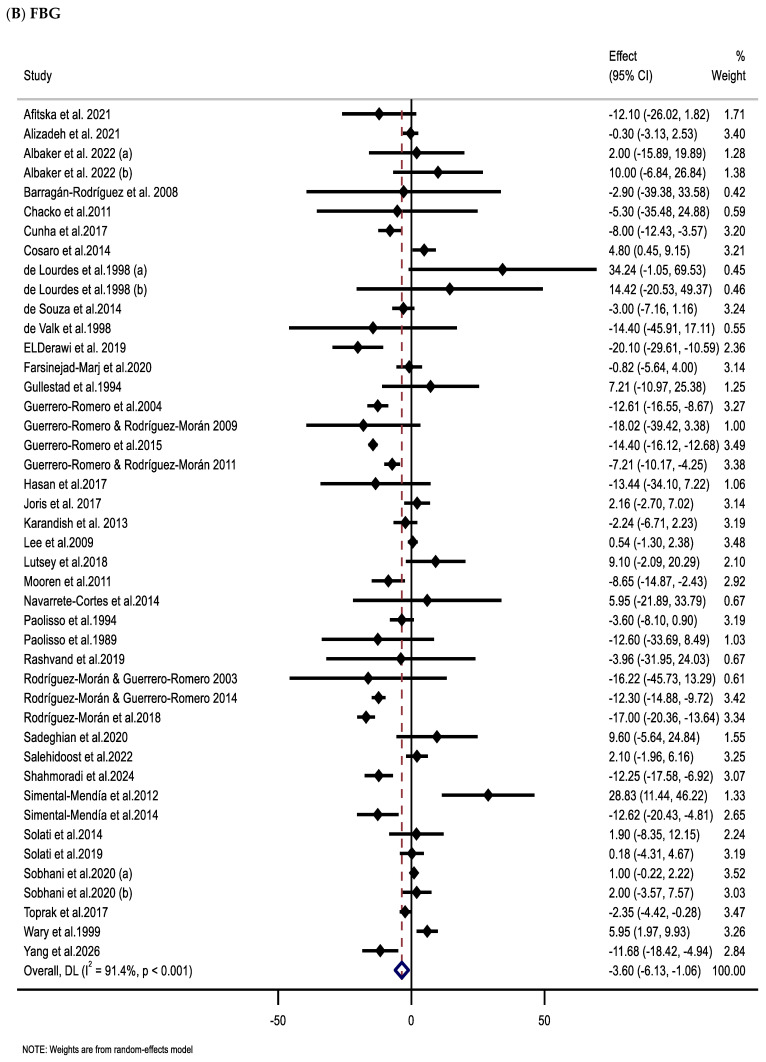

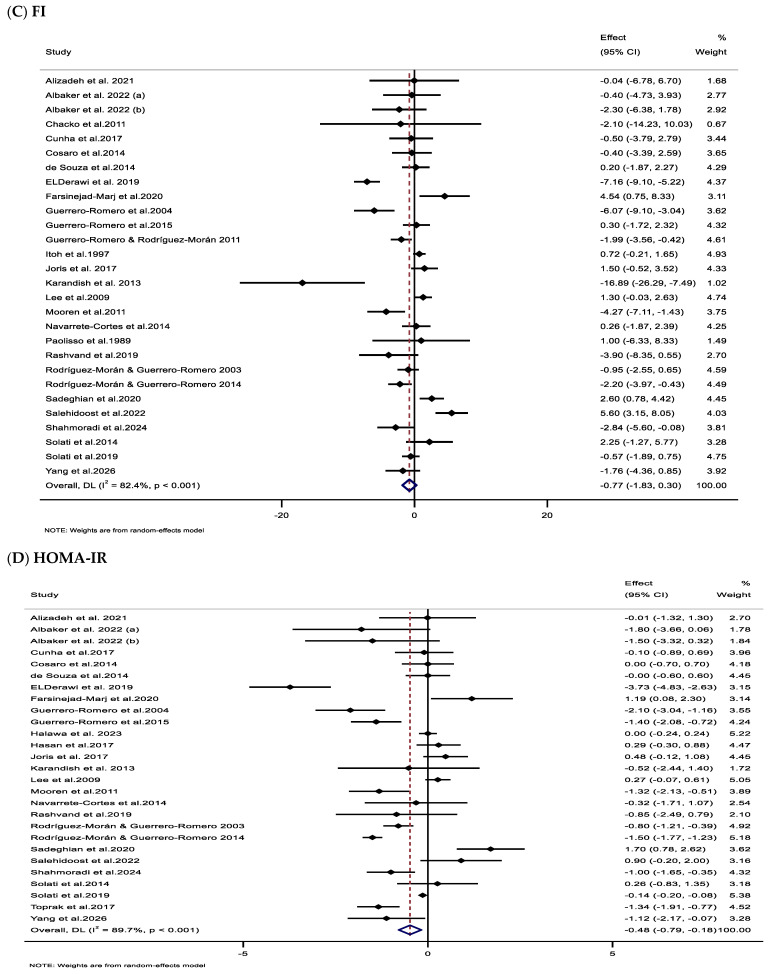
Forest plots present WMDs and 95% CIs for the impacts of Mg supplementation on glycemic profile, including (**A**) HbA1c (%), (**B**) FBG (mg/dL), (**C**) FI (µIU/mL), and (**D**) HOMA-IR [71,72,73,74,77,78,79,81,82,83,84,86,87,88,90,91,92,93,94,95,96,98,99,100,103,104,106,108,111,113,114,118,120,121,122,126,127,130,132,133,134,135,136,137,139,144].

The pooled analysis revealed that Mg supplementation significantly reduced HOMA-IR (WMD: −0.48, 95% CI: −0.79, −0.18), serum HbA1c (WMD: −0.15%, 95% CI: −0.26, −0.03), and FBG (WMD: −3.60 mg/dL, 95% CI: −6.13, −1.06) in comparison with the placebo group. However, substantial heterogeneity was detected among the studies (Figure 4 and Table 2). No considerable impact of Mg supplementation was observed on FI concentrations (WMD: −0.77 µIU/mL, 95% CI: −1.83, 0.30).

Subgroup analyses showed that Mg supplementation substantially reduced serum FBG in trials with intervention durations ≥ 12 weeks, Mg doses ≥ 300 mg/day, inorganic, chelated, or mixed Mg formulations, and moderate or mixed Mg bioavailability among participants with overweight, those aged ≥50 years, females, or participants of both sexes, and individuals with baseline FBG ≥ 100 mg/dL. In contrast, a significant increase in FBG levels was detected among men. FI was significantly reduced in studies that used mixed Mg formulations and moderate or mixed Mg bioavailability. HbA1c was substantially lower in trials with a duration ≥ 12 weeks, inorganic or mixed Mg formulations, low or mixed Mg bioavailability, among individuals with obesity, females, both sexes, and those aged ≥50 years. Similarly, HOMA-IR displayed a significant decrease in RCTs with a duration ≥ 12 weeks, Mg doses ≥ 300 mg/day, inorganic or mixed Mg formulations, moderate or mixed Mg bioavailability, among participants with overweight, both sexes, and those aged ≥50 years (Table S2).

#### 3.3.3. Effects of Mg Supplementation on Lipid Profile

**Figure 5:**
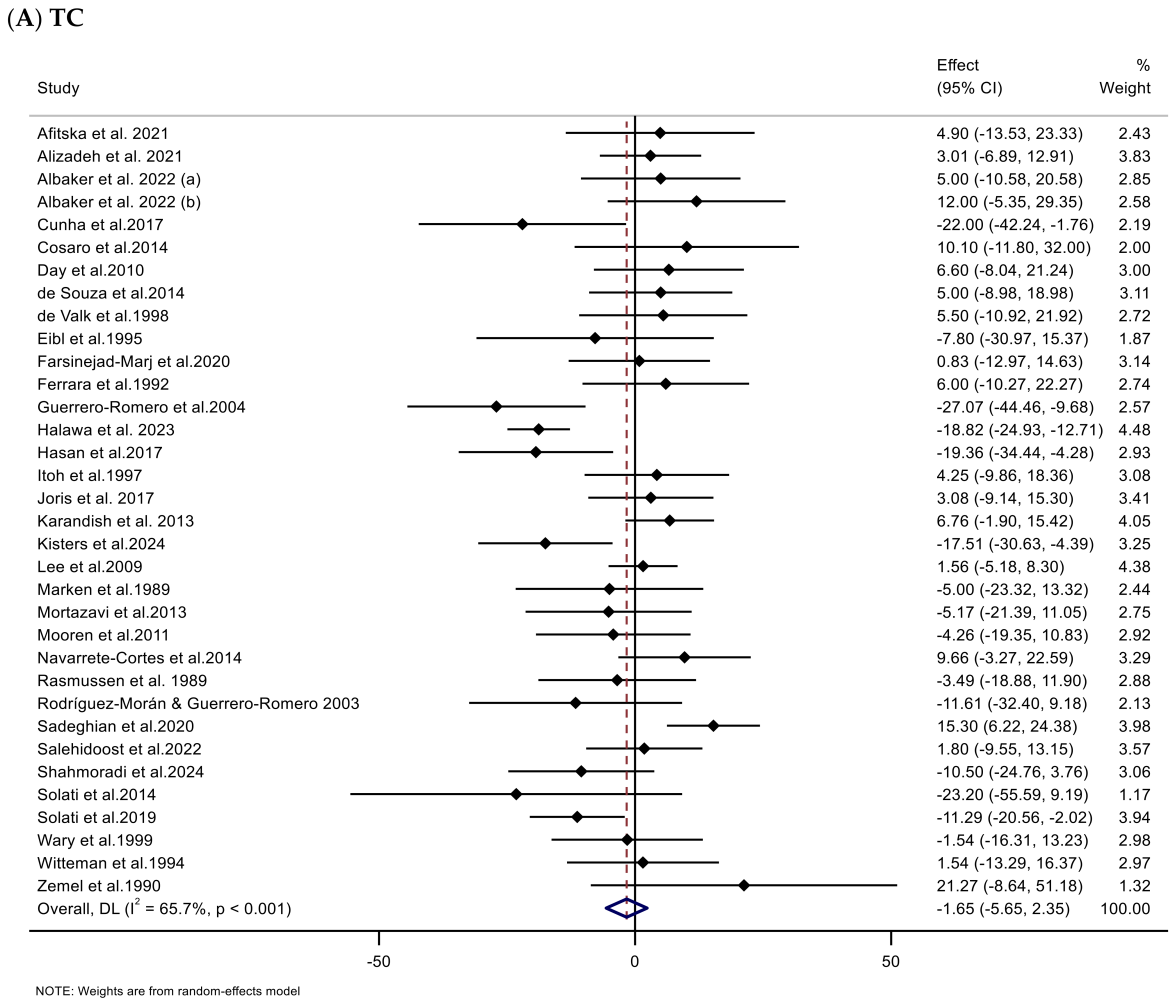

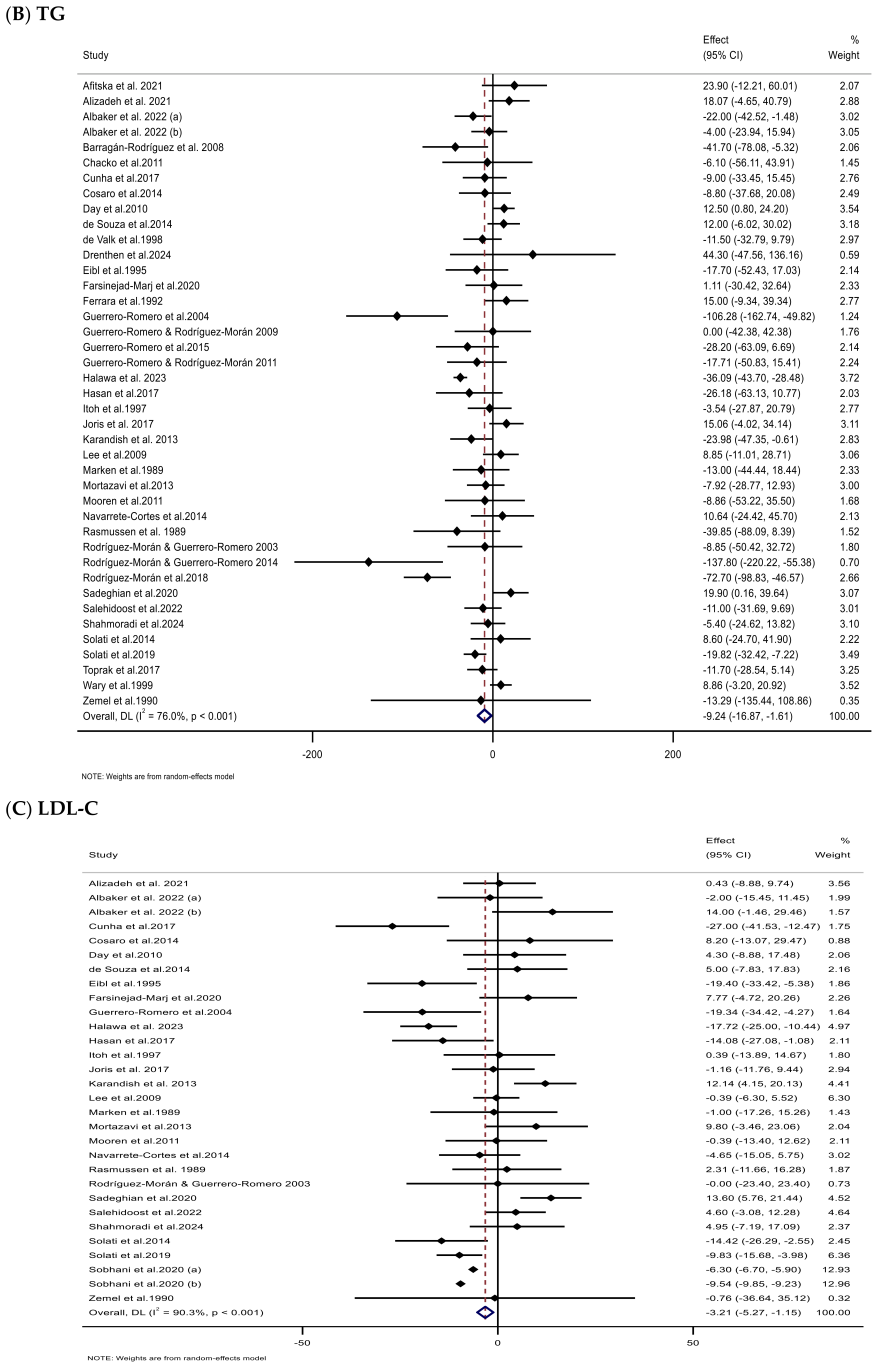

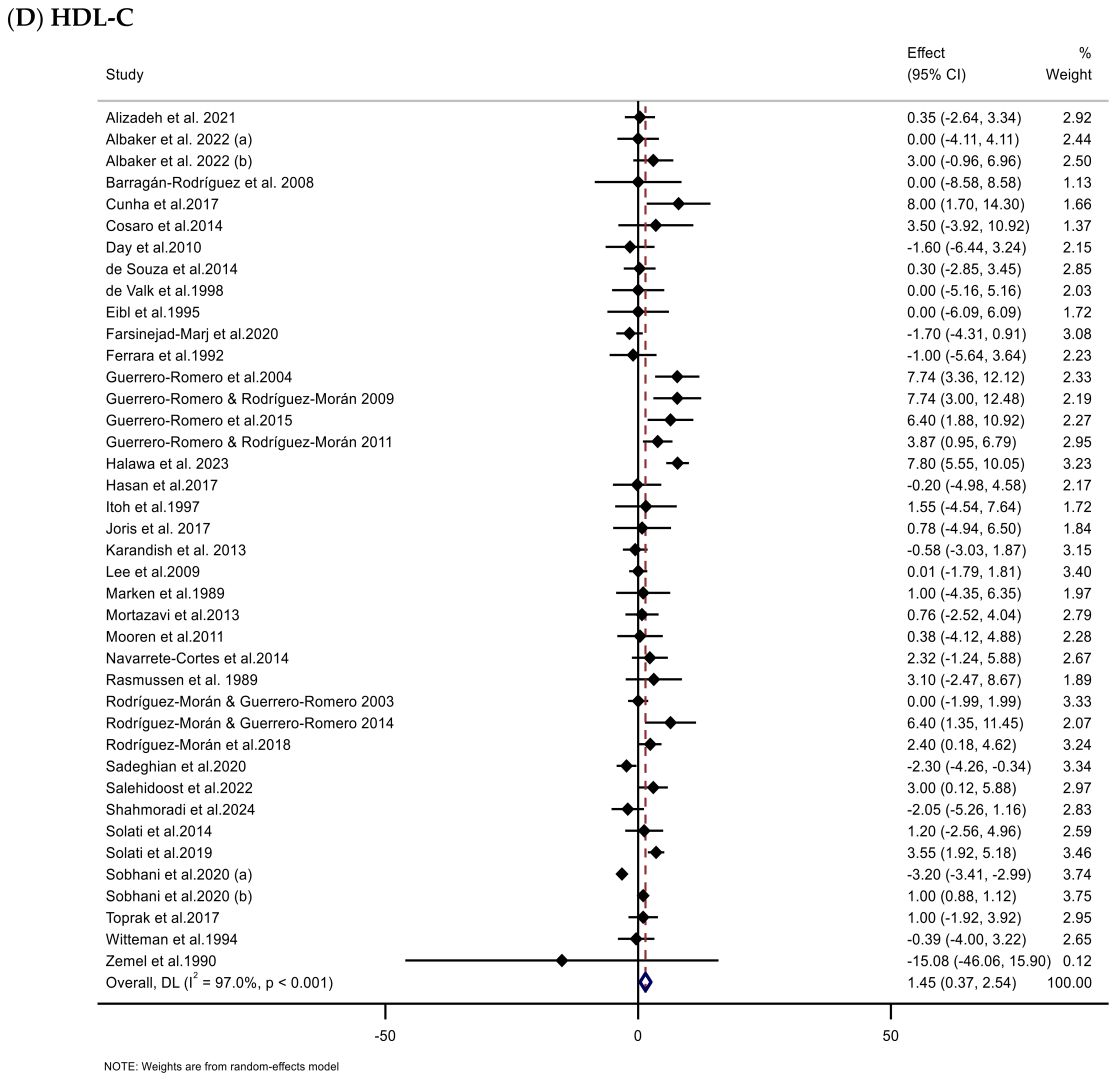
Forest plots present WMDs and 95% CIs for the impacts of Mg supplementation on lipid profile, including (**A**) TC (mg/dL), (**B**) TG (mg/dL), (**C**) LDL-C (mg/dL), and (**D**) HDL-C (mg/dL) [71,72,73,74,77,78,79,80,82,83,84,86,88,89,90,91,92,93,95,96,98,99,100,103,104,107,108,109,111,119,120,121,122,126,127,130,134,135,136,137,139,142,146].

The meta-analysis revealed that Mg supplementation considerably decreased TG (WMD: −9.24 mg/dL, 95% CI: −16.87 to −1.61) and LDL-C levels (WMD: −3.21 mg/dL, 95% CI: −5.27, −1.15) in comparison with the placebo group. HDL-C levels significantly increased following Mg supplementation (WMD: 1.45 mg/dL, 95% CI: 0.37, 2.54). However, no substantial effects were found on TC (WMD: −1.65 mg/dL, 95% CI: −5.65, 2.35). Considerable heterogeneity was identified across the trials (Figure 5 and Table 2).

Subgroup analyses suggested that Mg supplementation substantially reduced TG in RCTs with a duration ≥ 12 weeks, Mg doses ≥ 300 mg/day, inorganic Mg formulations, moderate Mg bioavailability, among participants with overweight, both sexes, and those with baseline TG ≥ 150 mg/dL. Serum LDL-C levels were significantly reduced in RCTs with a duration ≥ 12 weeks, Mg doses ≥ 300 mg/day, organic Mg formulations, high or moderate Mg bioavailability, among individuals of both sexes with normal BMI or overweight and baseline LDL-C < 100 mg/dL, as well as those aged ≥50 years. Conversely, low Mg bioavailability was related to a considerable increase in serum LDL-C concentrations. HDL-C significantly increased in RCTs with a duration ≥ 12 weeks, Mg doses ≥ 300 mg/day, inorganic Mg formulations, moderate Mg bioavailability, among individuals with overweight, both sexes, and participants with baseline HDL-C < 50 mg/dL (Table S2).

#### 3.3.4. Effects of Mg Supplementation on Blood Pressure

**Figure 6:**
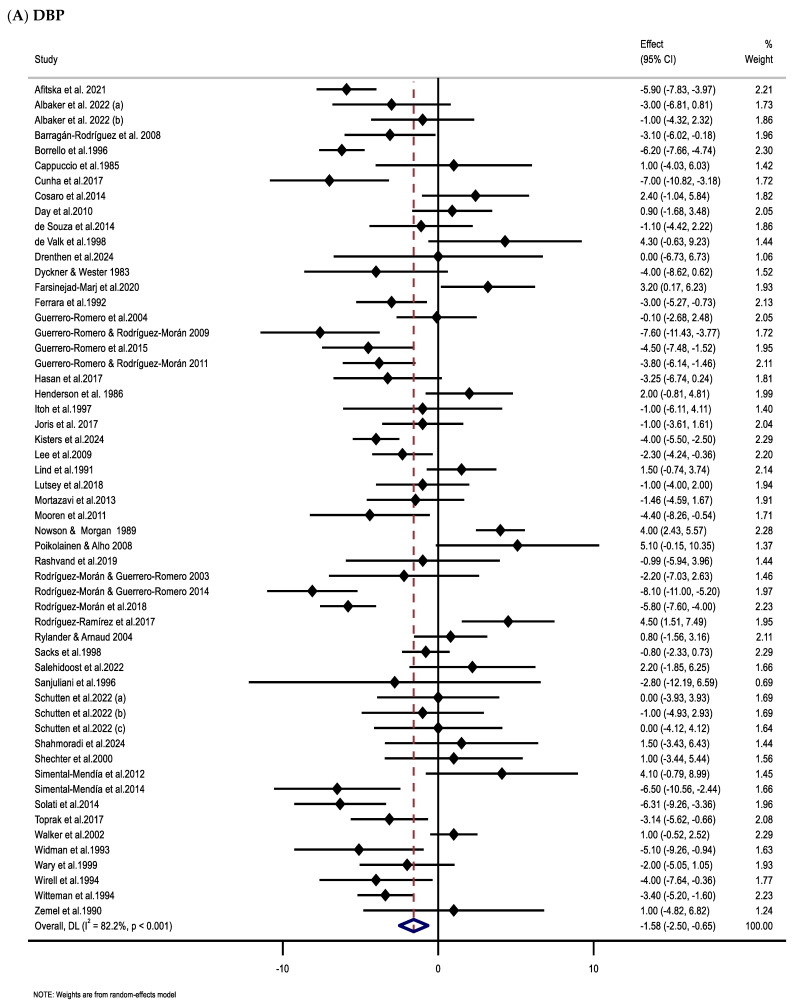

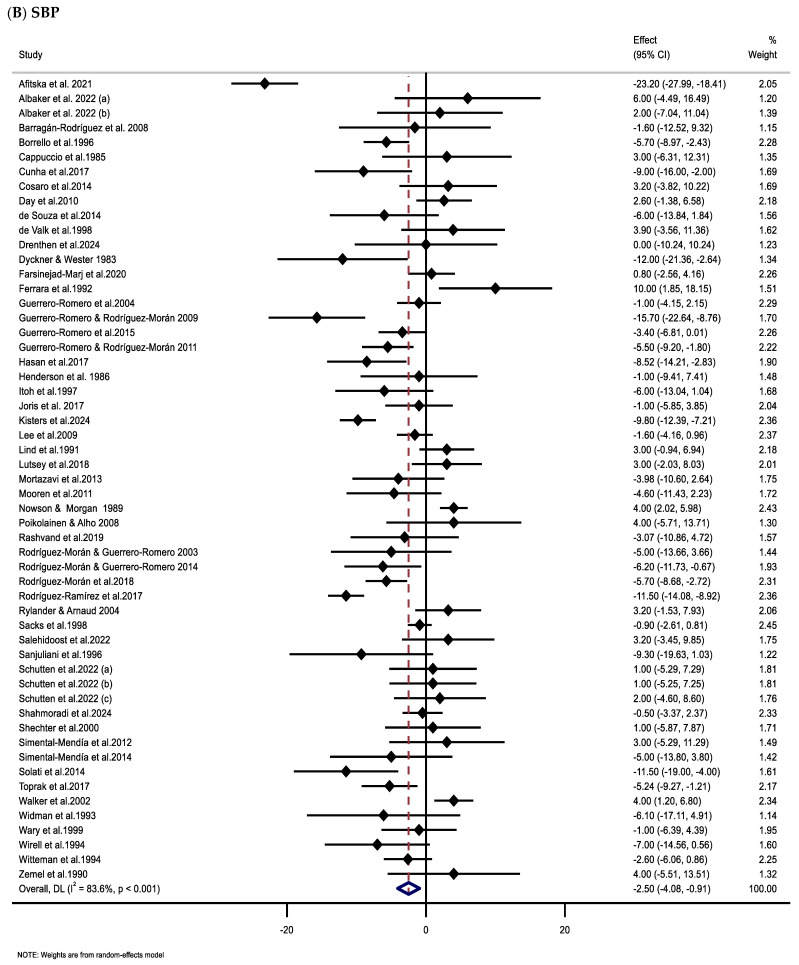
Forest plots present WMDs and 95% CIs for the impacts of Mg supplementation on blood pressure, including (**A**) DBP (mmHg) and (**B**) SBP (mmHg) [71,72,74,75,76,78,79,80,82,83,84,85,88,89,90,91,92,93,96,97,98,99,103,104,105,106,108,109,112,115,118,120,121,122,123,124,125,127,128,129,130,131,132,133,136,137,138,139,140,141,142,146].

The pooled analysis indicated that Mg supplementation was associated with significantly lower DBP (WMD: −1.58 mmHg, 95% CI: −2.50, −0.65) and SBP (WMD: −2.50 mmHg, 95% CI: −4.08, −0.91) compared with the placebo or control groups (Figure 6 and Table 2). Substantial heterogeneity was detected among the included trials.

Subgroup analyses revealed that Mg supplementation substantially decreased SBP in trials with a duration ≥ 12 weeks, Mg doses ≥ 300 mg/day, inorganic Mg formulations, low or moderate Mg bioavailability, among participants with baseline SBP ≥ 130 mmHg, obesity or overweight, both sexes, and those in both age categories (<50 and ≥50 years). Likewise, DBP showed a significant decrease in RCTs with a duration ≥ 12 weeks, Mg doses ≥ 300 mg/day, inorganic Mg formulations, moderate Mg bioavailability, among participants with baseline DBP ≥ 80 or <80 mmHg, individuals with obesity, both sexes, and those aged ≥50 or <50 years (Table S2).

#### 3.3.5. Effects of Mg Supplementation on Inflammatory Biomarkers

**Figure 7:**
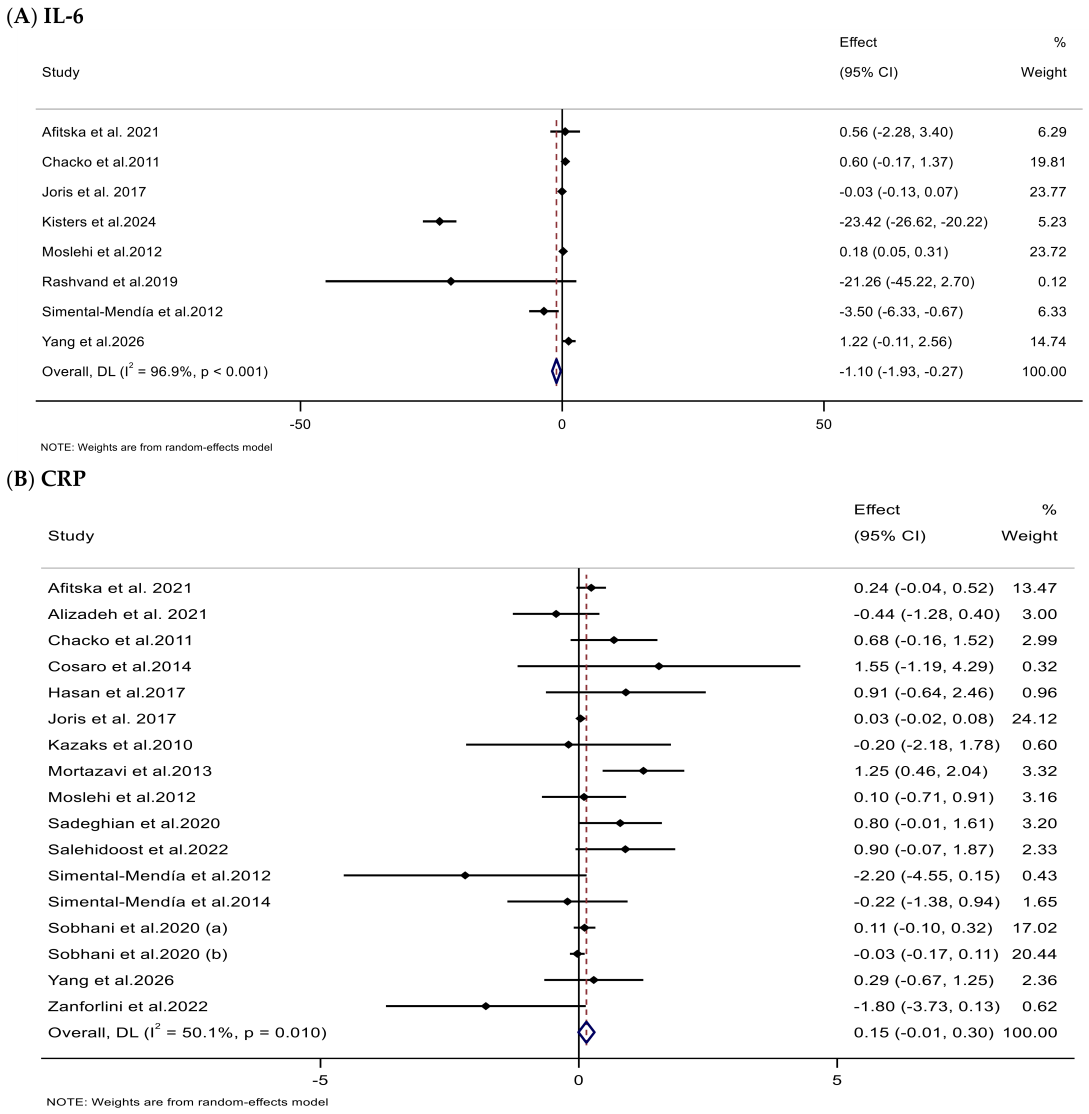

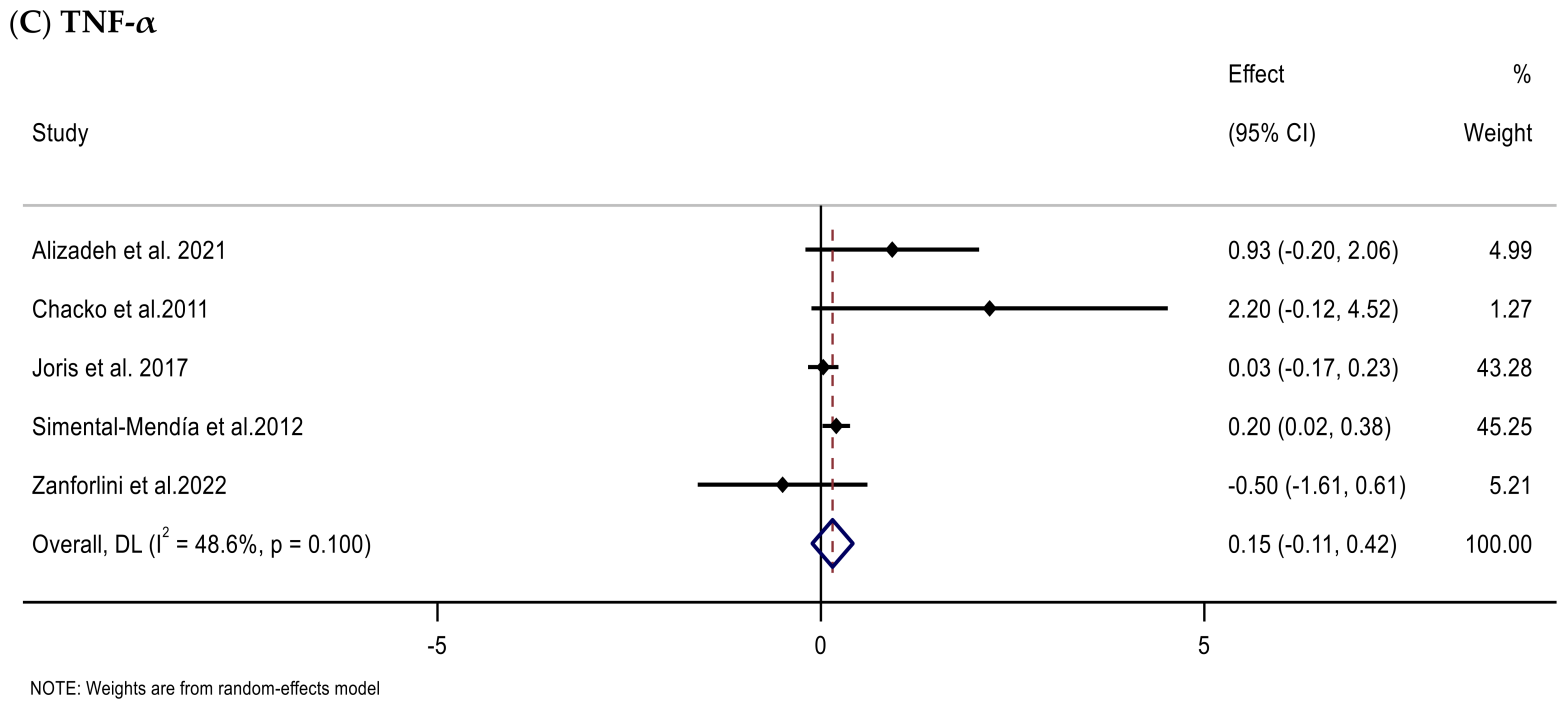
Forest plots present WMDs and 95% CIs for the impacts of Mg supplementation on inflammatory parameters, including (**A**) IL-6 (pg/mL), (**B**) CRP (mg/L), and (**C**) TNF-α (pg/mL) [71,73,77,78,96,99,101,103,109,110,118,126,127,132,133,134,144,145].

The meta-analysis demonstrated that Mg supplementation significantly reduced serum IL-6 levels (WMD: −1.10 pg/mL, 95% CI: −1.93, −0.27) in comparison with the control group. In contrast, no significant changes were observed in CRP (WMD: 0.15 mg/L, 95% CI: −0.01, 0.30) or TNF-α (WMD: 0.15 pg/mL, 95% CI: −0.11, 0.42). Considerable heterogeneity was identified across the included trials (Figure 7 and Table 2).

Subgroup analyses indicated that Mg supplementation was associated with a significant increase in CRP levels at Mg doses < 300 mg/day. IL-6 levels decreased significantly with Mg doses ≥ 300 mg/day, organic Mg formulations, and high or moderate Mg bioavailability among participants of both sexes. However, Mg doses < 300 mg/day in females were associated with significant increases in IL-6 levels. TNF-α displayed a significant increase in trials with a duration of <12 weeks, moderate Mg bioavailability, and participants with obesity (Table S2).

#### 3.3.6. Effects of Mg Supplementation on Kidney and Liver Function Markers

**Figure 8:**
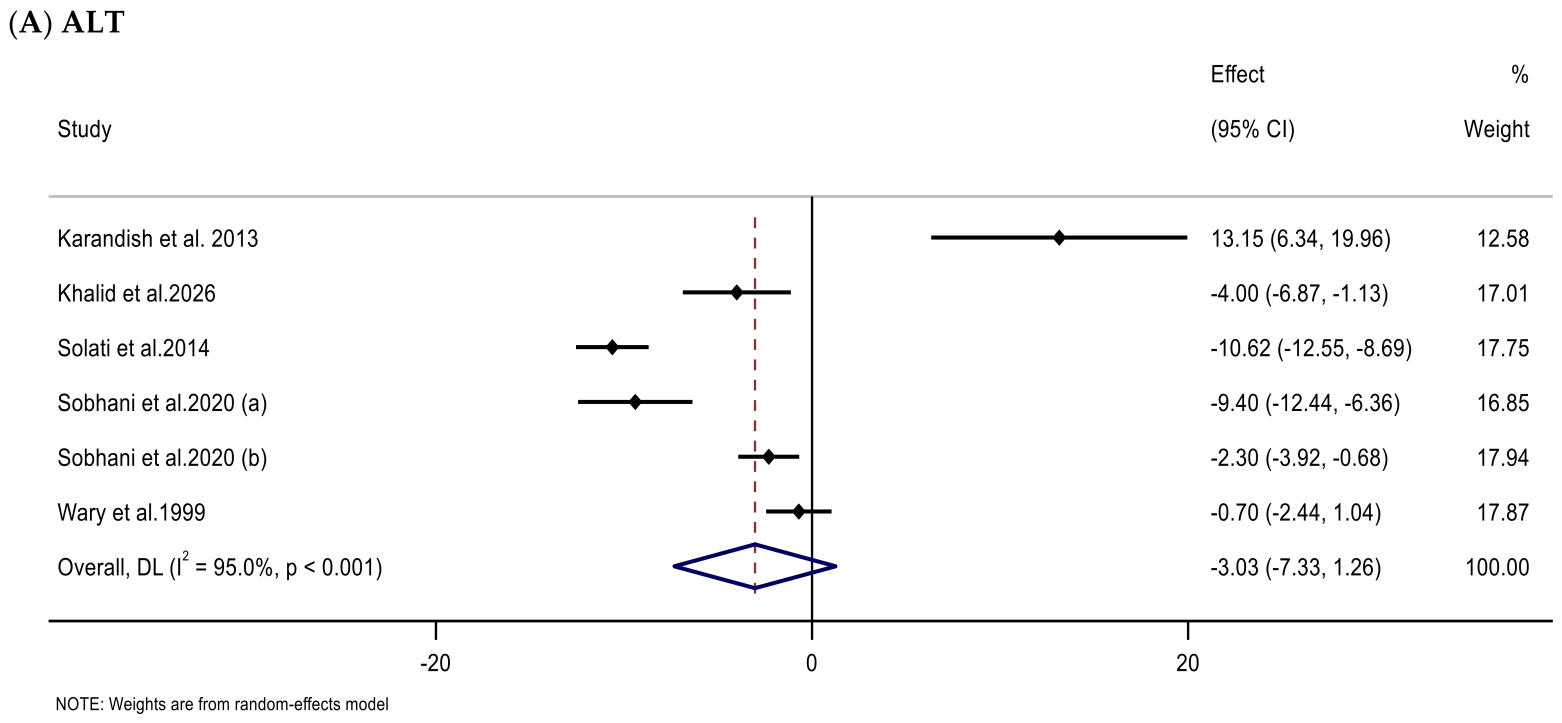

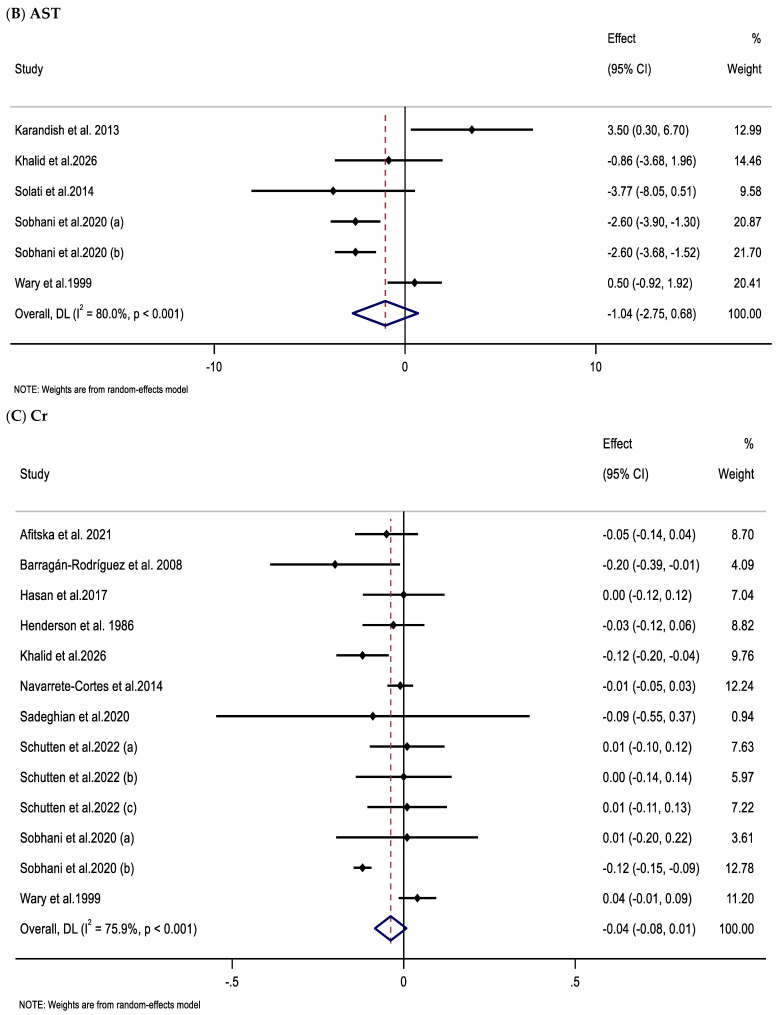
Forest plots present WMDs and 95% CIs for the impacts of Mg supplementation on liver and kidney function markers, including (**A**) ALT (IU/L), (**B**) AST (IU/L), and (**C**) Cr (mg/dL) [71,74,96,97,100,102,111,126,129,134,136,139].

The meta-analysis revealed that Mg supplementation did not substantially affect serum AST (WMD: −1.04 IU/L, 95% CI: −2.75, 0.68), ALT (WMD: −3.03 IU/L, 95% CI: −7.33, 1.26), or Cr levels (WMD: −0.04 mg/dL, 95% CI: −0.08, 0.01) compared with the control group (Figure 8 and Table 2).

Subgroup analyses displayed that AST levels decreased significantly in trials with moderate Mg bioavailability among individuals aged ≥50 years, whereas low Mg bioavailability was associated with substantial increases in AST levels. ALT levels decreased significantly with moderate Mg bioavailability among individuals with overweight; however, low Mg bioavailability was associated with substantial increases in ALT levels. Serum Cr levels decreased significantly in trials that used Mg doses ≥300 mg/day and among participants of both sexes (Table S2).

### 3.4. Risk of Bias Evaluation

Table S3 summarizes the RoB assessment of the included RCTs. Among the included studies, 10 trials [72,74,83,85,87,95,96,102,114,147] were classified as having a high RoB, 64 RCTs [70,71,73,75,76,77,78,79,80,82,84,86,88,89,90,91,92,93,94,97,99,100,101,103,104,106,107,108,109,110,111,112,113,115,116,117,118,119,120,121,122,123,124,126,127,128,129,130,131,132,133,134,135,136,137,138,139,140,141,142,143,144,145,146] were considered low risk, and four trials [81,98,105,125] were rated as raising some concerns.

### 3.5. GRADE

A summary of the GRADE for each outcome is displayed in Table 2 and Table S4. High-certainty evidence was identified for BFP and TNF-α. Moderate-certainty evidence was observed for BW, BMI, HC, HbA1c, HOMA-IR, DBP, IL-6, CRP, and Cr. Low-certainty evidence was found for WC, FBG, FI, TC, HDL-C, SBP, AST, and ALT. Very low-certainty evidence was detected for TG and LDL-C.

### 3.6. Sensitivity Analysis

Sensitivity analyses showed that the pooled findings were generally consistent after the stepwise removal of individual RCTs, indicating that no single study substantially influenced the results for most outcomes, including HC, BMI, HbA1c, BFP, FBG, HDL-C, HOMA-IR, TC, FI, TG, SBP, DBP, CRP, and TNF-α. However, the pooled estimates were influenced by the removal of certain trials for BW [96], LDL-C [134], Cr [139], WC [91,111,123,132,134], IL-6 [103], ALT [100], and AST [100].

### 3.7. Publication Bias

Egger’s test revealed possible publication bias for LDL-C and BW, whereas Begg’s test suggested possible publication bias for TG. For all other outcomes, neither Begg’s nor Egger’s test displayed significant evidence of publication bias. In addition, visual inspection of the funnel plots displayed varying degrees of asymmetry across several outcomes (Figure S1).

### 3.8. Nonlinear and Linear Dose–Response Associations

No significant linear (Figures S2 and S3) or nonlinear (Figures S4 and S5) dose–response relationships were observed between Mg dose or trial duration and changes in BFP, FI, BW, HOMA-IR, HDL-C, SBP, TC, CRP, BMI, AST, IL-6, TNF-α, or ALT. Fractional polynomial analyses identified significant nonlinear associations between Mg dose and DBP (coefficient = −2.58, *p* = 0.025) and between trial duration and WC (coefficient = −11.26, *p* = 0.028), HC (coefficient = −17.11, *p* = 0.014), FBG (coefficient = −13.51, *p* = 0.008), HbA1c (coefficient = −0.81, *p* = 0.039), TG (coefficient = −33.29, *p* = 0.001), and Cr (coefficient = −0.38, *p* = 0.007). These coefficients represent fractional polynomial model parameters and should not be interpreted as the magnitude of change in outcomes per unit increase in Mg dose or intervention duration. Linear meta-regression analyses further indicated that Mg dose was significantly associated with changes in LDL-C (β = −0.02, *p* = 0.042) (Figure S2).

## 4. Discussion

### 4.1. Summary of Findings

This meta-analysis of 78 RCTs showed that Mg supplementation significantly decreased BW, DBP, LDL-C, HOMA-IR, SBP, TG, IL-6, HbA1c, and FBG, while increasing HDL-C levels. However, no substantial impacts were identified on CRP, TNF-α, HC, Cr, BFP, TC, BMI, FI, WC, ALT, and AST.

Subgroup analyses suggested that the beneficial effects of Mg supplementation on glycemic control, lipid profile, and BP were generally more pronounced in trials with longer intervention durations (≥12 weeks) and higher Mg doses (≥300 mg/day), particularly among participants with less favorable baseline metabolic characteristics, including high FBG, TG, and BP levels or lower HDL-C concentrations. Several outcomes also varied according to Mg formulation and estimated bioavailability, suggesting that these factors may influence the magnitude of response to supplementation. In contrast, improvements in anthropometric measures (BW, BMI, and WC) were more frequently observed in studies with shorter intervention durations (<12 weeks) and lower Mg doses (<300 mg/day), indicating that anthropometric and metabolic outcomes may respond differently to supplementation strategies. The findings for inflammatory, renal, and hepatic biomarkers were less consistent across analyses. Overall, subgroup analyses suggested that intervention duration, Mg dose, formulation, bioavailability, and baseline metabolic status may influence the response to Mg supplementation.

Dose–response analyses indicated several nonlinear associations between Mg supplementation and cardiometabolic outcomes. Specifically, Mg dose was nonlinearly associated with changes in DBP, whereas intervention duration showed nonlinear associations with changes in HC, WC, FBG, HbA1c, TG, and Cr. These findings suggest that the response to Mg supplementation may vary across different doses and intervention periods rather than following a simple linear pattern. In addition, linear meta-regression analyses found a significant inverse association between Mg dose and LDL-C, indicating that higher Mg doses were associated with greater reductions in LDL-C. No significant dose–response relationships were observed for the remaining outcomes.

The available evidence was predominantly based on RCTs that used Mg doses ≥ 300 mg/day (79.5%) and intervention durations ≥ 12 weeks (66.7%). However, evidence from higher-dose Mg supplementation (≥500 mg/day) and longer-term interventions (≥25 weeks) was limited. Although subgroup and dose–response analyses provide insights into potential variations according to dose and duration, these findings should be considered in the context of the available supplementation protocols and heterogeneity across included trials.

Despite subgroup, sensitivity, and dose–response analyses, substantial residual heterogeneity remained for several outcomes, suggesting that other unexplored sources of variability likely contributed to differences across studies. Accordingly, pooled effect estimates for these outcomes should be interpreted with caution. Moreover, although several pooled effects were statistically significant, their effect sizes were generally modest, and the clinical significance of these findings remains uncertain due to variable certainty of evidence across outcomes. Furthermore, universally accepted minimal clinically important differences (MCIDs) have not been established for many cardiometabolic biomarkers evaluated in nutritional intervention studies, making direct assessment of clinical relevance challenging. Therefore, these results indicate favorable changes in intermediate cardiometabolic biomarkers rather than definitive evidence of clinically meaningful therapeutic effects.

### 4.2. Findings in the Context of Existing Literature

Several meta-analyses have investigated the impacts of Mg supplementation on individual cardiometabolic outcomes; however, their findings have not always been consistent. Previous dose–response meta-analyses reported significant decreases in anthropometric indices, including reductions in BW, BMI, and WC, following Mg supplementation [48,49]. In the current, larger and more comprehensive analysis, Mg supplementation significantly reduced BW, whereas no significant overall effects were detected on WC, BMI, HC, or BFP.

Regarding glycemic control, the findings of this meta-analysis are generally consistent with prior evidence demonstrating effects of Mg supplementation in reducing HbA1c, FBG, and HOMA-IR, particularly among individuals with T2DM or IR [35,40,43]. However, unlike several earlier meta-analyses [45,46], this study did not demonstrate a substantial overall impact on FI. This finding is not entirely unexpected, as a previous meta-analysis similarly reported no significant effect on FI despite improvements in HOMA-IR [47]. Overall, these results suggest that Mg supplementation may ameliorate glucose homeostasis, although its impacts on insulin-related biomarkers are less consistent.

In terms of lipid profile, the current meta-analysis displayed significant reductions in LDL-C and TG levels, as well as increases in HDL-C concentrations following Mg supplementation. However, a recent dose–response meta-analysis similarly reported a significant increase in HDL-C levels but found no significant effects on TG, TC, or LDL-C concentrations [39]. In contrast, some earlier meta-analyses reported improvements in TG and LDL-C levels following Mg supplementation [54,55], with more pronounced impacts identified in metabolically compromised populations [54]. These findings suggest that the lipid-related effects of Mg supplementation may vary according to baseline metabolic status and study characteristics. No significant impact on TC despite favorable changes in LDL-C, TG, and HDL-C may reflect the composite nature of TC and the variability in lipid responses across study populations.

The BP-lowering effects observed in this study are highly consistent with the findings of multiple previous meta-analyses [38,41,42,50,51,52,53], which demonstrated significant reductions in both SBP and DBP following Mg supplementation, particularly among individuals with high CMR.

Evidence regarding the anti-inflammatory effects of Mg supplementation remains inconsistent. Some meta-analyses reported reductions in selected inflammatory biomarkers following Mg supplementation [34,56], whereas others found limited or no significant effects [37,57]. For example, a meta-analysis [57] concluded that Mg supplementation did not significantly improve IL-6, CRP, or TNF-α levels in the general adult population, while another [37] observed significant reductions primarily in CRP without conclusive effects on other inflammatory markers. In this meta-analysis, Mg supplementation significantly decreased serum levels of IL-6 but had no overall effect on CRP or TNF-α.

Limited evidence is currently available regarding the effects of Mg supplementation on hepatic and renal biomarkers. The current study found no significant overall effects on ALT, AST, or Cr levels. The relatively small number of trials reporting these outcomes, together with the variability in baseline hepatic and renal status across study populations, may partly explain these null findings.

Several methodological differences between this study and previous meta-analyses may help explain the discrepancies across the findings. This meta-analysis included a larger number of recently published RCTs and evaluated a broader range of CMRFs. Moreover, variability in the intervention duration, Mg dose, formulation, participant characteristics, and baseline metabolic status across studies may have contributed to the differences in the observed effects. Such heterogeneity may partly explain why some previous meta-analyses reported significant findings for outcomes such as BMI, FI, or CRP, which were not consistently observed in the present larger and more heterogeneous analysis.

### 4.3. Possible Underlying Mechanisms

Mg is an essential intracellular cation involved in numerous enzymatic reactions related to energy metabolism, glucose homeostasis, vascular function, and inflammation [21,22,148]. The significant impacts of supplementation with Mg on BW in the current meta-analysis may be partly explained by its role in regulating energy metabolism and insulin sensitivity [149,150,151]. Mg deficiency has been related to chronic low-grade inflammation, impaired glucose utilization, and metabolic dysfunction, all of which may contribute to adiposity and weight gain [150,152]. Improved insulin action and metabolic regulation following Mg supplementation may contribute to reductions in body weight [153].

The significant reductions in HOMA-IR, HbA1c, and FBG may be related to the important role of Mg in insulin signaling pathways and glucose metabolism [151,154,155]. Mg functions as a cofactor for multiple enzymes involved in carbohydrate metabolism and is essential for normal insulin receptor activity as well as downstream post-receptor signaling pathways [22,155]. Low Mg status has been related to impaired insulin sensitivity, defective glucose uptake, and increased risk of T2DM [44,156]. Moreover, Mg may help improve pancreatic β-cell function and cellular glucose transport, thereby contributing to improved glycemic control [157].

The significant decreases in TG and LDL-C levels, as well as increases in HDL-C concentrations, may be associated with the role of Mg in lipid metabolism and insulin sensitivity [39,151]. Mg deficiency has been linked to dyslipidemia, possibly through impaired activity of enzymes involved in lipoprotein metabolism and altered lipid transport [158]. Improvements in IR may also indirectly contribute to favorable changes in circulating HDL-C and TG [159].

Significant declines in SBP and DBP identified in this meta-analysis may be explained by several vascular and metabolic effects of Mg [160,161]. Mg contributes to endothelial function and vascular tone regulation and acts as a natural calcium antagonist that promotes vascular relaxation [162,163]. It may also enhance nitric oxide availability and reduce vascular resistance, thereby contributing to the reduction in BP [164]. Furthermore, the antioxidative and anti-inflammatory properties of Mg may help attenuate endothelial dysfunction and vascular stiffness [162,165].

Significant reductions in IL-6 concentrations may reflect the anti-inflammatory properties of Mg supplementation [166]. Mg deficiency has been related to increased production of pro-inflammatory cytokines and activation of inflammatory pathways [152]. Experimental and clinical evidence suggests that adequate Mg status may suppress inflammatory signaling pathways and decrease the production of pro-inflammatory cytokines, including IL-6, thereby contributing to an improved inflammatory status [56,167].

The proposed mechanisms should be viewed as biologically plausible interpretations based on prior experimental and clinical evidence. As this meta-analysis was not designed to assess mechanistic pathways, causal inferences cannot be established.

### 4.4. Safety of Mg Supplements

The safety profile of Mg supplementation is generally well established, with adverse events primarily dose-dependent and gastrointestinal in nature. The most frequently reported side effect is diarrhea [168], which is attributed to the osmotic activity of unabsorbed Mg salts in the intestine and colon [64]. However, contemporary evidence suggests that the current Tolerable Upper Intake Level (UL) of 350 mg/day for Supplemental Mg has been questioned and may be conservative based on clinical tolerance data. A systematic review of 49 clinical trials of oral Mg supplementation, with doses reaching up to 972 mg/day, reported no serious adverse reactions; all adverse events were minor and transient [169]. Serious adverse events, such as hypermagnesemia, remain exceedingly rare in individuals with normal renal function; however, the risk is substantially elevated in patients with CKD, in whom impaired renal excretion may lead to Mg accumulation, even at moderate doses [170]. For healthy individuals with normal renal function, Mg supplementation is generally considered safe at commonly recommended doses, with gastrointestinal symptoms being the most frequently reported adverse effect and serious toxicity being rare [171].

### 4.5. Clinical Implications

In this meta-analysis, Mg supplementation was associated with statistically significant improvements in several major CMRFs, including BW, glycemic and lipid profiles, BP, and IL-6 levels. However, the magnitude of these effects was generally modest. These results suggest that Mg supplementation may be a beneficial adjunctive intervention for improving CMH. However, because the included RCTs evaluated surrogate cardiometabolic biomarkers rather than hard clinical endpoints, these findings should not be interpreted as direct evidence of reduced CMD risk or improved long-term clinical outcomes. The findings may be particularly relevant for individuals with obesity, IR, T2DM, HTN, hyperlipidemia, or inadequate dietary Mg intake, who are more likely to have suboptimal Mg status. Therefore, Mg should be considered a complementary nutritional strategy rather than a substitute for established lifestyle or pharmacological interventions. Consistent with current dietary recommendations, Mg requirements should ideally be met through a balanced diet, with supplementation considered when dietary intake is inadequate or clinically indicated. Furthermore, the certainty of the evidence varied across outcomes, and baseline Mg status was inconsistently reported across studies. Although individuals with inadequate Mg status may derive greater benefit from supplementation, the current evidence is insufficient to adequately evaluate this possibility.

Despite these promising findings, important clinical considerations, such as supplementation duration, formulation, optimal dose, and target populations, remain insufficiently established. Therefore, large-scale and long-term RCTs are required before routine clinical recommendations can be fully established.

### 4.6. Strengths and Limitations

This dose–response meta-analysis evaluated the impacts of Mg supplementation across a wide spectrum of CMRFs. A major strength of this study was the inclusion of diverse cardiometabolic outcomes, providing a broader understanding of Mg’s potential metabolic effects. This study included only RCTs and applied subgroup, sensitivity, dose–response, and publication bias analyses to further explore the robustness of the findings. Importantly, subgroup analyses based on Mg formulation (organic and inorganic chemical forms) and bioavailability categories provided further insights into the potential influence of Mg type on cardiometabolic outcomes. Furthermore, most of the included RCTs had low RoB, and the GRADE revealed moderate-to-high certainty of evidence for several major outcomes, supporting the credibility of the results.

This meta-analysis had several limitations. Substantial heterogeneity existed across the included RCTs with respect to Mg dose, formulation, intervention duration, sample size, participant characteristics, and baseline metabolic status. The included populations varied considerably, ranging from healthy individuals to patients with obesity, T2DM, HTN, MetS, and other chronic diseases, which may have contributed to the observed variability in effect estimates. Consequently, the pooled estimates should be interpreted with caution, particularly for outcomes with substantial residual heterogeneity. Moreover, baseline Mg status, dietary Mg intake, medication use, renal function, and lifestyle-related factors were reported inconsistently across studies, which may limit the ability to determine whether specific subgroups experience greater effects from Mg supplementation. The majority of included RCTs were not designed to recruit participants according to Mg status, and only a limited number of studies specifically enrolled individuals with Mg deficiency or hypomagnesemia. Therefore, subgroup analyses according to baseline Mg status were not feasible. Another limitation was the relatively small number of RCTs available for some outcomes (AST, TNF-α, HC, and ALT) and several subgroup analyses. Furthermore, the available evidence was derived from a limited range of Mg doses and intervention durations, restricting conclusions regarding the optimal supplementation strategy and long-term efficacy of Mg supplementation. In addition, the certainty of evidence was rated as very low or low for several outcomes, including LDL-C, FI, HDL-C, WC, TG, FBG, SBP, ALT, TC, and AST. Therefore, although some of these outcomes reached statistical significance, the findings require confirmation by additional well-designed RCTs.

Regardless of these limitations, this meta-analysis provides updated evidence regarding the potential role of Mg supplementation in CMH and offers important insights for future clinical trials and nutritional strategies targeting the prevention and management of CMD.

## 5. Conclusions

This meta-analysis, which included 78 RCTs, revealed that Mg supplementation was associated with significant improvements in several CMRFs, including BW, LDL-C, SBP, HOMA-IR, DBP, HbA1c, TG, FBG, IL-6, and HDL-C. However, no substantial effects were identified on CRP, TNF-α, HC, Cr, BFP, TC, BMI, FI, WC, ALT, or AST. The results suggest that Mg supplementation may contribute to improvements in CMH, particularly in glycemic and lipid profiles, BP, and IL-6 levels. However, the observed effect sizes were generally modest, and the certainty of evidence ranged from very low to high across outcomes. The majority of the included trials used Mg doses ≥ 300 mg/day and intervention durations ≥ 12 weeks; however, evidence on higher-dose supplementation (≥500 mg/day) and longer-term interventions (≥25 weeks) was limited. In addition, uncertainties remain regarding the optimal dose, formulation, bioavailability, and duration of Mg supplementation. Moreover, long-term safety outcomes were insufficiently evaluated in the included RCTs. Additional long-term and large-scale RCTs are required to establish evidence-based recommendations and identify the populations most likely to benefit from Mg supplementation.

## Figures and Tables

**Table 1:** Characteristics of included RCTs in the meta-analysis.

Reference	Study Region	Study Design	Participants	Sex	Sample Size		Trial Duration (Weeks)	Mean Age		Mean BMI		Intervention		
					IG	CG		IG	CG	IG	CG	Mg Dose (mg/Day)	Mg Formulations	CG
Afitska et al. 2021 [71]	Germany	P, R, DB, PC	Patients with MetS	♀/♂	13	11	12	61.8 ± 10.7	71.9 ± 7.8	33.7 ± 5.1	35.2 ± 5.6	400	Mg citrate	PL
Alizadeh et al. 2021 [73]	Iran	P, R, DB, PC	Women with PCOS	♀	21	20	8	25.5 ± 4.8	26.2 ± 5.7	27.9 ± 3.2	26.9 ± 3.8	250	Mg oxide	PL
Albaker et al. 2022 (a) [72]	Saudi Arabia	P, R, CO	Patients with T2DM	♀/♂	33	37	12	57.5 ± 7.0	59.20 ± 8.7	32.0 ± 4.0	32.7 ± 8.1	20	Mg chloride	CO (desalinated water)
Albaker et al. 2022 (b) [72]	Saudi Arabia	P, R, CO	Patients with T2DM	♀/♂	32	37	12	55.9 ± 8.9	59.20 ± 8.7	31.1 ± 4.7	32.7 ± 8.1	50	Mg chloride	CO (desalinated water)
Barragán-Rodríguez et al. 2008 [74]	Mexico	P, R, SB, CO	Elderly with T2DM	♀/♂	12	9	12	69 ± 5.9	66.4 ± 6.1	NR	NR	450	Mg chloride	CO (imipramine)
Borrello et al. 1996 [75]	Italy	P, R, DB, PC	Patients with HTN	♀/♂	42	41	12	51 ± 7	49 ± 5	NR	NR	200	Mg oxide	PL
Cappuccio et al. 1985 [76]	UK	C, R, DB, PC	Patients with HTN	♀/♂	17	17	4	51.7 ± 8.3	51.7 ± 8.3	NR	NR	365	Mg aspartate	PL
Chacko et al. 2011 [77]	USA	C, R, DB, PC	Individuals with OW	♀/♂	13	13	4	44.4 ± 48.6	44.4 ± 48.6	28.2 ± 6.7	28.2 ± 6.7	500	Mg citrate	PL
Cunha et al. 2017 [79]	Brazil	P, R, DB, PC	Women with HTN	♀	17	18	24	54 ± 7	57 ± 5	29.7 ± 4.1	26.8 ± 3.9	600	Mg chelate	PL
Cosaro et al. 2014 [78]	Italy	C, R, DB, PC	Healthy men	♂	14	14	8	26.3 ± 3.1	26.3 ± 3.1	24.0 ± 3.1	24.0 ± 3.1	368	Mg pidolate	PL (lactose)
Day et al. 2010 [80]	Australia	P, R, DB, PC	Postmenopausal women	♀	34	33	12	59 ± 5.5	57 ± 4.4	24.1 ± 3.1	25.0 ± 3.6	180–216	Mg bicarbonate	PL (spring water)
de Lourdes et al. 1998 (a) [81]	Brazil	P, R, DB, PC	Patients with T2DM	♀/♂	35	54	4	55.4 ± 10.2	55.5 ± 8.3	25.3 ± 8.0	25.5 ± 6.5	503	Mg oxide	PL
de Lourdes et al. 1998 (b) [81]	Brazil	P, R, DB, PC	Patients with T2DM	♀/♂	39	54	4	51.2 ± 11.0	55.5 ± 8.3	25.5 ± 6.5	25.5 ± 6.5	1007	Mg oxide	PL
de Souza et al. 2014 [82]	Brazil	P, R, DB, PC	Women with MetS	♀	30	32	12	44.6 ± 9.7	46.6 ± 12.3	35.5 ± 8.2	35.1 ± 6.3	400	Mg chelate	PL
de Valk et al. 1998 [83]	Netherlands	P, R, CO	Patients with T2DM	♀/♂	25	25	12	63.0 ± 8.2	62.0 ± 7.3	28.7 ± 5.4	27.1 ± 4.5	365	Mg aspartate	CO
Drenthen et al. 2024 [84]	Netherlands	C, R, DB, PC	Patients with T2DM	♀/♂	14	14	6	67 ± 6	67 ± 6	31.3 ± 4.9	31.3 ± 4.9	360	Mg gluconate	PL
Dyckner & Wester 1983 [85]	Sweden	P, R, CO	Patients with HTN or congestive HF	♀/♂	20	19	24	62.2 ± 4.2	67 8 ± 4	NR	NR	365	Mg aspartate	CO
Eibl et al. 1995 [86]	Austria	P, R, DB, PC	Patients with T2DM	♀/♂	18	20	12	63 ± 8	54 ± 1.5	27.5 ± 3.2	29.3 ± 5	730	Mg citrate	PL
Abbasi et al. 2012 [70]	Iran	P, R, DB, PC	Individuals with OW or OB	♀/♂	21	22	8	64.7 ± 4.7	65.4 ± 4.5	28.3 ± 3.1	30.1 ± 4.1	500	Mg oxide	PL (starch)
ELDerawi et al. 2019 [87]	Palestine	P, R, CO	Patients with T2DM	♀/♂	20	20	12	51.15 ± 7.0	51.55 ± 8.2	29.0 ± 5.0	30.0 ± 4.5	250	Mg oxide, gluconate, & lactate	CO
Farsinejad-Marj et al. 2020 [88]	Iran	P, R, DB, PC	Women with PCOS	♀	30	30	8	26.3 ± 3.9	26 ± 5.0	27.8 ± 5.0	27.7 ± 5.4	250	Mg oxide	PL (lactose, starch, etc.)
Ferrara et al. 1992 [89]	Italy	P, R, DB, PC	Patients with HTN	♀/♂	7	7	24	48 ± 6	47 ± 3	26.48	25.93	365	Mg pidolate	PL
Gullestad et al. 1994 [94]	Norway	P, R, DB, PC	Patients with NIDDM	♀/♂	25	29	16	64 ± 8	64 ± 8	25.4 ± 3.7	25.3 ± 4.1	365	Mg lactate & Mg citrate	PL
Guerrero-Romero et al. 2004 [92]	Mexico	P, R, DB, PC	Non-diabetic individuals with IR	♀/♂	32	31	12	43.0 ± 7.9	42.2 ± 6.8	29.3 ± 6.3	29.1 ± 3.1	300	Mg chloride	PL
Guerrero-Romero & Rodríguez-Morán 2009 [90]	Mexico	P, R, DB, PC	Patients with T2DM & HTN	♀/♂	40	39	16	58.9 ± 8.5	60.5 ± 9.4	29.9 ± 5.2	29.0 ± 5.1	450	Mg chloride	PL
Guerrero-Romero et al. 2015 [91]	Mexico	P, R, DB, PC	Individuals with prediabetes & hypomagnesaemia	♀/♂	59	57	16	42.5 ± 9.5	42.3 ± 8.0	30.6 ± 6.4	31.7 ± 4.7	382	Mg chloride	PL
Guerrero-Romero & Rodríguez-Morán 2011 [93]	Mexico	P, R, DB, PC	Healthy individuals	♀/♂	49	48	12	39.8 ± 5.3	41.4 ± 6.2	29.8 ± 4.9	28.4 ± 6.8	450	Mg chloride	PL
Halawa et al. 2023 [95]	Egypt	P, R, CO	Patients with DN	♀/♂	26	28	12	61.4 ± 7.5	63 ± 7.2	30.4 ± 3.6	30.5 ± 4.4	360	Mg citrate	CO
Hasan et al. 2017 [96]	Iraq	P, R, PC	Women with MetS	♀	30	17	8	53.7 ± 8.2	50.1 ± 6.9	34.8 ± 4.9	34.9 ± 3.7	168	Mg lactate	PL
Henderson et al. 1986 [97]	Denmark	P, R, DB, PC	Patients with HTN	♀/♂	20	20	24	62	62	NR	NR	301	Mg oxide	PL
Itoh et al. 1997 [98]	Japan	P, R, DB, PC	Healthy individuals	♀/♂	23	10	4	64 ± 9	66 ± 18	23.5 ± 3.9	23.3 ± 3.8	411–548	Mg hydroxide	PL
Joris et al. 2017 [99]	Netherlands	P, R, DB, PC	Individuals with OW or OB	♀/♂	26	25	24	62 ± 6	62 ± 6	29.3 ± 2.6	29.9 ± 3.1	350	Mg citrate	PL (starch)
Karandish et al. 2013 [100]	Iran	P, R, DB, PC	Patients with NAFLD	♀/♂	34	34	13	36 ± 7	36 ± 7	30.6 ± 4.3	31.7 ± 5.6	350	Mg oxide	PL (lactose)
Kazaks et al. 2010 [101]	USA	P, R, DB, PC	Patients with asthma	♀/♂	27	25	26	37 ± 10.4	37 ± 10	29 ± 5.2	28 ± 5	340	Mg citrate	PL (starch & cellulose)
Khalid et al. 2026 [102]	Pakistan	P, R, SB, PC	Patients with T2DM & insomnia	♀/♂	75	68	8	49.0 ± 9.50	49.0 ± 9.5	31.0 ± 23.6	31.0 ± 23.6	500	Mg gluconate	PL (starch)
Kisters et al. 2024 [103]	Germany	P, R, DB, PC	Patients with MetS	♀/♂	27	27	12	60.2 ± 12.5	64.6 ± 13.2	31.4 ± 2.8	30.8 ± 1.5	400	Mg citrate	PL
Lee et al. 2009 [104]	South Korea	P, R, DB, PC	Individuals with OW	♀/♂	75	80	12	39.6 ± 7.9	40.5 ± 7.3	26.7 ± 2.8	26.1 ± 2.3	300	Mg oxide	PL
Lind et al. 1991 [105]	Sweden	P, R, DB, PC	Patients with mild HTN or high-normal BP	♀/♂	49	22	24	60 ± 9.4	62 ± 7.8	NR	NR	365	Mg lactate & Mg citrate	PL
Lutsey et al. 2018 [106]	USA	P, R, DB, PC	Healthy individuals	♀/♂	29	30	12	61.3 ± 5.3	61.6 ± 5.2	27.7 ± 4.9	28.0 ± 4.5	400	Mg oxide	PL (lactose)
Marken et al. 1989 [107]	USA	C, R, DB, PC	Healthy individuals	♀/♂	35	35	8	30 ± 7.3	30 ± 7.3	NR	NR	800	Mg oxide	PL
Mortazavi et al. 2013 [109]	Iran	P, R, DB, PC	Hemodialysis Patients	♀/♂	27	25	24	56.9 ± 12.1	56.3 ± 11.1	NR	NR	188.6	Mg oxide	PL
Mooren et al. 2011 [108]	Germany	P, R, DB, PC	Individuals with OW	♀/♂	25	22	24	30–70	30 -70	30.9 ± 3.2	30.8 ± 3.6	365	Mg aspartate	PL
Moslehi et al. 2012 [110]	Iran	P, R, DB, PC	Women with OW	♀	35	34	8	46.5 ± 3.8	46.1 ± 4.6	28.0 ± 3.2	28.1 ± 2.9	250	Mg oxide	PL (starch, lactose & stearic acid)
Navarrete-Cortes et al. 2014 [111]	Mexico	C, R, DB, PC	Patients with T2DM	♀/♂	56	56	12	52.8 ± 8.4	52.8 ± 8.4	30.5 ± 5.6	30.5 ± 5.8	360	Mg lactate	PL
Nowson & Morgan 1989 [112]	Australia	P, R, DB, PC	Patients with HTN	♀/♂	12	13	8	62.7 ± 2.1	62.6 ± 1.7	NR	NR	243.3	Mg aspartate	PL
Paolisso et al. 1994 [113]	Italy	C, R, DB, PC	Patients with T2DM	♀/♂	9	9	4	73 ± 7.5	73 ± 7.5	25.8 ± 0.3	25.8 ± 0.3	384.5	Mg pidolate	PL
Paolisso et al. 1989 [114]	Italy	C, R, PC	Patients with T2DM	♀/♂	8	8	4	72.2 ± 5.7	72.2 ± 5.7	NR	NR	170	Mg pidolate	PL
Poikolainen & Alho 2008 [115]	Finland	P, R, DB, PC	Individuals with alcohol use disorder	♀/♂	27	31	8	48.7 ± 9.8	48.7 ± 9.8	NR	NR	500	Mg carbonate, acetate, & hydroxide	PL
Pokan et al. 2006 [116]	Austria	P, R, DB, PC	Patients with CAD	♂	28	25	24	61 ± 9	58 ± 10	27.0 ± 4.1	26.7 ± 3.9	730	Mg citrate	PL
Rajizadeh et al. 2017 [117]	Iran	P, R, DB, PC	Patients with depression and Mg deficiency	♀/♂	26	27	8	32.2 ± 9.5	32.0 ± 7.6	28.5 ± 4.5	25.8 ± 5.6	500	Mg oxide	PL
Rasmussen et al. 1989 [119]	Denmark	P, R, DB, PC	Patients with IHD	♀/♂	22	21	12	62	61.4	NR	NR	365	Mg hydroxide	PL (amylum solany & methylcellulose
Rashvand et al. 2019 [118]	Iran	P, R, DB, PC	Patients with T2DM	♀/♂	18	19	8	49.8 ± 7.8	48.2 ± 14.2	29.6 ± 3.2	29.3 ± 3.7	500	Mg oxide	PL (starch)
Rodríguez-Morán & Guerrero-Romero 2003 [120]	Mexico	P, R, DB, PC	Patients with T2DM	♀/♂	32	31	16	59.7 ± 8.3	54.1 ± 9.6	27.6 ± 9.1	28.6 ± 4.2	637	Mg chloride	PL
Rodríguez-Morán & Guerrero-Romero 2014 [121]	Mexico	P, R, DB, PC	MONW individuals	♀/♂	24	23	16	31.9	39.5	22.4 ± 1.6	22.7 ± 1.9	382	Mg chloride	PL
Rodríguez-Morán et al. 2018 [122]	Mexico	P, R, DB, PC	Patients with MetS	♀/♂	100	98	16	39.4 ± 9.8	40.4 ± 10.6	29.7 ± 6.5	30.5 ± 5.9	382	Mg chloride	PL
Rodríguez-Ramírez et al. 2017 [123]	Mexico	P, R, DB, PC	Individuals with pre-HTN	♀/♂	18	18	16	53.1 ± 5.9	50.4 ± 3.1	27.3 ± 3.1	29.4 ± 6.9	360	Mg lactate	PL
Rylander & Arnaud 2004 [124]	Sweden	P, R, DB, PC	Individuals with borderline HTN	♀/♂	18	18	4	45–64	45–64	NR	NR	82.3	Mg sulfate	PL (water low in minerals)
Sacks et al. 1998 [125]	USA	P, R, DB, PC	Women with low habitual intake of Mg	♀	50	103	16	39 ± 4.4	38 ± 4.5	23.5 ± 3.5	23.2 ± 3.1	336	Mg lactate	PL
Sadeghian et al. 2020 [126]	Iran	P, R, DB, PC	Patients with DN	♀/♂	40	40	12	41.2 ± 8.8	42.8 ± 8.4	31.2 ± 5.5	30.9 ± 4.4	250	Mg oxide	PL (lactose, starch, etc.)
Salehidoost et al. 2022 [127]	Iran	P, R, DB, PC	Individuals with prediabetes	♀/♂	34	37	12	56.7 ± 5.9	54.8 ± 4.9	30.0 ± 3.3	30.7 ± 5.1	250	Mg oxide	PL (starch)
Sanjuliani et al. 1996 [128]	Brazil	C, R, DB, PC	Patients with HTN	♀/♂	15	15	6	35–65	35–65	NR	NR	600	Mg oxide	PL
Schutten et al. 2022 (a) [129]	Netherlands	P, R, DB, PC	Individuals with OW or OB	♀/♂	46	26	24	64.1 ± 6.1	63.8 ± 7.8	28.7 ± 3.4	27.8 ± 2.4	450	Mg citrate	PL (starch)
Schutten et al. 2022 (b) [129]	Netherlands	P, R, DB, PC	Individuals with OW or OB	♀/♂	46	26	24	63.2 ± 6.7	63.8 ± 7.8	27.5 ± 2.1	27.8 ± 2.4	450	Mg oxide	PL (starch)
Schutten et al. 2022 (c) [129]	Netherlands	P, R, DB, PC	Individuals with OW or OB	♀/♂	46	26	24	62.0 ± 6.9	63.8 ± 7.8	28.4 ± 2.9	27.8 ± 2.4	450	Mg sulfate	PL (starch)
Shahmoradi et al. 2024 [130]	Iran	P, R, TB, PC	Women with PCOS	♀	20	20	8	27.4 ± 5.0	29.0 ± 4.2	29.7 ± 6.4	30.6 ± 5.0	250	Mg oxide	PL
Shechter et al. 2000 [131]	USA	P, R, DB, PC	Patients with CAD	♀/♂	25	25	24	68 ± 10	66 ± 12	25 ± 4	26 ± 4	730	Mg oxide & Mg carbonate	PL
Simental-Mendía et al. 2012 [133]	Mexico	P, R, DB, PC	Individuals with prediabetes	♀/♂	11	11	12	44.2 ± 10.8	43.2 ± 7.8	30.5 ± 4.4	35.1 ± 7.0	382	Mg chloride	PL
Simental-Mendía et al. 2014 [132]	Mexico	P, R, DB, PC	Individuals with prediabetes	♀/♂	29	28	12	39.8 ± 16	41.1 ± 13.1	31.4 ± 6.7	32.7 ± 5.9	382	Mg chloride	PL (sodium bicarbonate)
Solati et al. 2014 [136]	Iran	P, R, DB, PC	Patients with T2DM	♀/♂	25	22	12	46.7 ± 9	50.1 ± 6.9	26.1 ± 2.8	26.8 ± 5.2	300	Mg sulfate	PL (wheat bran)
Solati et al. 2019 [135]	Iran	P, R, DB, PC	Individuals with OW	♀/♂	35	35	24	40.7 ± 11.9	40.6 ± 12.7	28.8 ± 0.1	28.9 ± 0.1	300	Mg sulfate	PL (wheat bran)
Sobhani et al. 2020 (a) [134]	Iran	P, R, DB, PC	Patients with CAD (one atherosclerotic vessel)	♀/♂	13	13	24	59.2 ± 2.1	62 ± 1.6	23.9 ± 0.8	23.9 ± 0.3	300	Mg sulfate	PL
Sobhani et al. 2020 (b) [134]	Iran	P, R, DB, PC	Patients with CAD (two atherosclerotic vessels)	♀/♂	19	19	24	60.6 ± 1.8	61.2 ± 1.5	24.7 ± 0.7	23.5 ± 0.2	300	Mg sulfate	PL
Toprak et al. 2017 [137]	Turkey	P, R, DB, PC	Patients with obesity, pre diabetes, & CKD	♀/♂	57	61	12	55.5 ± 13.7	57.0 ± 12.9	33.8 ± 2.8	33.9 ± 2.6	365	Mg oxide	PL
Walker et al. 2002 [138]	UK	P, R, DB, PC	Patients with HTN	♀/♂	9	10	10	53.2 ± 11.4	49.4 ± 13	25.5 ± 4.8	27.5 ± 2.2	600	Mg chelate	PL (cellulose)
Widman et al. 1993 [140]	Sweden	C, R, DB, PC	Patients with HTN	♀/♂	16	17	3	50 ± 6	50 ± 6	NR	NR	960	Mg hydroxide	PL
Wary et al. 1999 [139]	France	P, R, DB, PC	Healthy male	♂	15	15	4	23.7 ± 4.5	23.7 ± 4.5	21.6 ± 2.6	21.8 ± 2.8	288	Mg lactate	PL
Wirell et al. 1994 [141]	Sweden	C, R, DB, PC	Patients with HTN	♀/♂	39	39	8	35.4 ± 10.8	35.4 ± 10.8	NR	NR	365	Mg aspartate	PL
Witteman et al. 1994 [142]	Netherlands	P, R, DB, PC	Women with HTN	♀	47	44	24	57.4 ± 11.9	57.1 ± 11.7	26.3 ± 4.0	26.4 ± 4.0	485	Mg aspartate	PL
Yang et al. 2026 [144]	China	P, R, DB, PC	Individuals with pre-diabetes	♀/♂	36	35	16	68.0 ± 7.2	69.4 ± 4.5	24.8 ± 3.4	24.6 ± 2.7	360	Mg oxide	PL (starch)
Yamamoto et al. 1995 [143]	USA	P, R, DB, PC	Healthy individuals	♀/♂	227	234	24	42.7 ± 6.2	43.2 ± 6.7	27.4 ± 3.7	27 ± 3.7	360	Mg diglycinate	PL
Zanforlini et al. 2022 [145]	Italy	P, R, DB, PC	Patients with COPD	♀/♂	25	24	24	73.0 ± 8.9	72.2 ± 11.0	26.9 ± 4.3	27.1 ± 4.3	300	Mg citrate	PL (maltodextrin)
Zemel et al. 1990 [146]	USA	P, R, DB, PC	Patients with HTN	♀/♂	7	6	12	53 ± 13.2	46 ± 9.8	26.5 ± 2.3	30.7 ± 4.1	960	Mg aspartate	PL
Zorbas et al. 1999 (a) [147]	Greece	P, R, CO	Endurance-trained athletes (normal activity)	♂	10	10	52	25.9 ± 6.5	24.3 ± 6.0	24.5 ± 3.2	24.9 ± 2.6	1782	Mg lactate	CO
Zorbas et al. 1999 (b) [147]	Greece	P, R, CO	Endurance-trained athletes (hypokinetic/sedentary)	♂	10	10	52	24.7 ± 8.6	25.6 ± 7.5	23.8 ± 2.3	24.8 ± 3.2	1769	Mg lactate	CO

Abbreviations: MetS, metabolic syndrome; HF, heart failure; OW, overweight; P, parallel; NIDDM, non-insulin dependent diabetes mellitus; C, crossover; IR, insulin resistance; IHD, ischemic heart disease; CO, controlled; MONW, metabolically obese normal weight; ♀, female; ♂, male; NR, not reported; OB, obesity; PCOS, polycystic ovary syndrome; PL, placebo; DB, double-blinded; CG, control group; HTN, hypertension; COPD, chronic obstructive pulmonary disease; USA, United States of America; BMI, body mass index; NAFLD, nonalcoholic fatty liver disease; SB, single-blinded; CAD, coronary artery disease; CKD, chronic kidney disease; IG, intervention group; DN, diabetic nephropathy; R, randomized; T2DM, type 2 diabetes mellitus; TB, triple blinded; PC, placebo-controlled; UK, United Kingdom; Mg, magnesium.

**Table 2:** Summary of the impacts of Mg supplementation on CMRFs.

CMRFs	Effect Sizes (*n*)	Participants (*n*)	WMD (95% CI)	*p*-Value	Heterogeneity		Certainty of the Evidence (GRADE)
					I^2^ (%)	*p*-Value	
Anthropometric measures							
BMI (kg/m^2^)	37	2636	−0.65 (−1.49, 0.19)	0.129	97.3	<0.001	Moderate (II)
WC (cm)	22	1413	−0.98 (−2.02, 0.05)	0.061	57.9	<0.001	Low (III)
BW (kg)	24	1227	−0.70 (−1.30, −0.09)	0.023	0	0.832	Moderate (II)
BFP (%)	9	400	−0.02 (−0.46, 0.43)	0.932	0	0.854	High (I)
HC (cm)	6	321	−0.60 (−1.79, 0.59)	0.321	0	0.983	Moderate (II)
Glycemic profile							
HbA1c (%)	27	1339	−0.15 (−0.26, −0.03)	0.010	68.3	<0.001	Moderate (II)
FBG (mg/dL)	44	2514	−3.60 (−6.13, −1.06)	0.005	91.4	<0.001	Low (III)
HOMA-IR	27	1655	−0.48 (−0.79, −0.18)	0.002	89.7	<0.001	Moderate (II)
FI (µIU/mL)	28	1587	−0.77 (−1.83, 0.30)	0.157	82.4	<0.001	Low (III)
Lipid profile							
TC (mg/dL)	34	1770	−1.65 (−5.65, 2.35)	0.420	65.7	<0.001	Low (III)
HDL-C (mg/dL)	40	2402	1.45 (0.37, 2.54)	0.009	97.0	<0.001	Low (III)
TG (mg/dL)	41	2328	−9.24 (−16.87, −1.61)	0.018	76.0	<0.001	Very low (IV)
LDL-C (mg/dL)	30	1571	−3.21 (−5.27, −1.15)	0.002	90.3	<0.001	Very low (IV)
Blood pressure							
DBP (mmHg)	55	3011	−1.58 (−2.50, −0.65)	0.001	82.2	<0.001	Moderate (II)
SBP (mmHg)	55	3011	−2.50 (−4.08, −0.91)	0.002	83.6	<0.001	Low (III)
Inflammatory biomarkers							
TNF-α (pg/mL)	5	176	0.15 (−0.11, 0.42)	0.261	48.6	0.100	High (I)
CRP (mg/L)	17	777	0.15 (−0.01, 0.30)	0.064	50.1	0.010	Moderate (II)
IL-6 (pg/mL)	8	341	−1.10 (−1.93, −0.27)	0.009	96.9	<0.001	Moderate (II)
Kidney & liver function markers							
Cr (mg/dL)	13	669	−0.04 (−0.08, 0.01)	0.106	75.9	<0.001	Moderate (II)
ALT (IU/L)	6	352	−3.03 (−7.33, 1.26)	0.166	95.0	<0.001	Low (III)
AST (IU/L)	6	352	−1.04 (−2.75, 0.68)	0.237	80.0	<0.001	Low (III)

Abbreviations: BFP, body fat percentage; BMI, body mass index; SBP, systolic blood pressure; WC, waist circumference; DBP, diastolic blood pressure; BW, body weight; HC, hip circumference; IL-6, interleukin-6; TG, triglycerides; ALT, alanine aminotransferase; TC, total cholesterol; AST, aspartate aminotransferase; HDL-C, high-density lipoprotein cholesterol; FBG, fasting blood glucose; LDL-C, low-density lipoproteins cholesterol; HbA1c, hemoglobin A1c; Cr, creatinine; HOMA-IR, homeostatic model assessment of insulin resistance; TNF-α, tumor necrosis factor-alpha; CRP, C-reactive protein; WMD, weighted mean difference; CI, confidence interval; CMRFs, cardiometabolic risk factors; FI, fasting insulin.

## Data Availability

The original contributions presented in this study are included in the article/Supplementary Materials. Further inquiries can be directed to the corresponding authors.

## Supplementary Materials

The following supporting information can be downloaded at https://www.mdpi.com/article/10.3390/nu18152435/s1. Figure S1: Funnel plots for the impacts of Mg supplementation on cardiometabolic risk factors (CMRFs); Figure S2: Linear dose-response association between dose (mg/day) of Mg supplementation and mean changes in cardiometabolic risk factors (CMRFs); Figure S3: Linear association between duration (weeks) of Mg supplementation and mean changes in cardiometabolic risk factors (CMRFs); Figure S4: Non-linear dose-response association between dose (mg/day) of Mg supplementation and mean changes in cardiometabolic risk factors (CMRFs); Figure S5: Non-linear association between duration (weeks) of Mg supplementation and mean changes in cardiometabolic risk factors (CMRFs); Table S1: Search strategy in PubMed (MEDLINE); Table S2: Subgroup analyses of the impacts of magnesium supplementation on cardiometabolic risk factors; Table S3: Risk of bias assessment; Table S4: GRADE assessment.

## Abbreviations

The following abbreviations are used in this manuscript:

BMI	Body mass index
ALT	Alanine aminotransferase
CKD	Chronic kidney disease
CRP	C-reactive protein
MONW	Metabolically obese normal weight
HC	Hip circumference
HF	Heart failure
LDL-C	Low-density lipoprotein cholesterol
NIDDM	Non-insulin-dependent diabetes mellitus
AST	Aspartate aminotransferase
IHD	Ischemic heart disease
Cr	Creatinine
WC	Waist circumference
ATP	Adenosine triphosphate
SBP	Systolic blood pressure
BFP	Body fat percentage
FBG	Fasting blood glucose
TG	Triglycerides
TC	Total cholesterol
DBP	Diastolic blood pressure
HDL-C	High-density lipoprotein cholesterol
IL-6	Interleukin-6
HbA1c	Hemoglobin A1c
WMD	Weighted mean difference
HOMA-IR	Homeostatic model assessment of insulin resistance
NAFLD	Nonalcoholic fatty liver disease
CAD	Coronary artery disease
Mg	Magnesium
TNF-α	Tumor necrosis factor-alpha
RCT	Randomized controlled trial
CI	Confidence interval
DN	Diabetic nephropathy
BW	Body weight
MetS	Metabolic syndrome
OS	Oxidative stress
PCOS	Polycystic ovary syndrome
T2DM	Type 2 diabetes mellitus
GRADE	Grading of recommendations assessment, development, and evaluation
COPD	Chronic obstructive pulmonary disease
USA	United States of America
HTN	Hypertension
OB	Obesity
PROSPERO	Prospective Register of Systematic Reviews
CMH	Cardiometabolic health
BP	Blood pressure
OW	Overweight
IR	Insulin resistance
UK	United Kingdom
CMD	Cardiometabolic disease
PRISMA	Preferred Reporting Items for Systematic Reviews and Meta-analyses
RoB	Risk of bias
CMRFs	Cardiometabolic risk factors
FI	Fasting insulin
CMR	Cardiometabolic risk
SD	Standard deviation
CVD	Cardiovascular disease
MCID	Minimal clinically important difference
