# Supplementary material for: Comprehensive Effects of Magnesium Supplementation on Cardiometabolic Risk Factors: A Systematic Review and Dose–Response Meta-Analysis
Source: Nutrients. 2026 Jul 25;18(15):2435. doi: 10.3390/nu18152435 (PMC13468553; doi:10.3390/nu18152435)
Supplement: Supplementary file 1 [file nutrients-18-02435-s001.zip › nutrients-4404018-supplementary.pdf]

Supplementary Materials

Table S1. Search strategy in PubMed (MEDLINE)

	Keywords	Search strategy
#1	**Magnesium**	("magnesium"[tiab] OR "magnesium supplementation"[tiab])
#2	**Cardiometabolic risk factors**	("body weight"[tiab] OR "waist circumference"[tiab] OR "WC"[tiab] OR "hip circumference"[tiab] OR "HC"[tiab] OR "body mass index"[tiab] OR "BMI"[tiab] OR "body fat percentage"[tiab] OR "BFP"[tiab] OR "creatinine"[tiab] OR "aspartate aminotransferase"[tiab] OR "AST"[tiab] OR "alanine aminotransferase"[tiab] OR "ALT"[tiab] OR "fasting insulin"[tiab] OR "homeostatic model assessment for insulin resistance"[tiab] OR "HOMA-IR"[tiab] OR "hemoglobin A1c"[tiab] OR "HbA1c"[tiab] OR "fasting blood sugar"[tiab] OR "FBS"[tiab] OR "fasting blood glucose"[tiab] OR "FBG"[tiab] OR "triglycerides"[tiab] OR "TG"[tiab] OR "total cholesterol"[tiab] OR "TC"[tiab] OR "low-density lipoprotein"[tiab] OR "LDL"[tiab] OR "high-density lipoprotein"[tiab] OR "HDL"[tiab] OR "C-reactive protein"[tiab] OR "CRP"[tiab] OR "tumor necrosis factor"[tiab] OR "TNF-alpha"[tiab] OR "TNF"[tiab] OR "interleukin-6"[tiab] OR "IL-6"[tiab] OR "diastolic blood pressure"[tiab] OR "DBP"[tiab] OR "systolic blood pressure"[tiab] OR "SBP"[tiab])
#3	**Study design**	("intervention"[tiab] OR "controlled trial"[tiab] OR "controlled clinical trial"[tiab] OR randomized [tiab] OR randomised [tiab] OR random*[tiab] OR placebo[tiab] OR "clinical trial"[tiab] OR "randomized controlled trial"[tiab] OR "randomized clinical trial"[tiab] OR "RCT" [tiab] OR blinded[tiab] OR "double blind"[tiab] OR trial[tiab] OR trials[tiab] OR crossover[tiab] OR "cross-over"[tiab] OR "crossover study"[tiab] OR "crossover trial"[tiab] OR parallel[tiab] OR "parallel study"[tiab] OR "parallel trial"[tiab])
#4	**Final search combination**	#1 AND #2 AND #3

Table S2. Subgroup analyses of the impacts of magnesium supplementation on cardiometabolic risk factors

**Sub-groups**	**Effect Sizes** (n)	**WMD** (95% CI)	**P-value**	**Heterogeneity**		
				**P-value**	**I²** (%)	**P-value between sub-groups**
**Impacts of Mg on BW** (kg)						
Overall effect	24	-0.70 (-1.30, -0.09)	**0.023**	0.832	0	
Trial duration (weeks)						
≥ 12	15	0.34 (-0.66, 1.35)	0.502	0.904	0	0.011
<12	9	-1.28 (-2.03, -0.52)	**0.001**	0.966	0	
Mg dose (mg/day)						
≥300	16	0.22 (-0.81, 1.26)	0.670	0.900	0	0.032
<300	8	-1.17 (-1.91, -0.42)	**0.002**	0.848	0	
Chemical class of Mg supplements						
Organic	8	-1.12 (-1.89, -0.35)	**0.004**	0.697	0	
Inorganic	15	-0.04 (-1.03, 0.94)	0.930	0.851	0	0.207
Chelated	1	0.90 (-4.86, 6.66)	0.760	-	-	
Bioavailability of Mg supplements						
High	9	-1.08 (-1.85, -0.32)	**0.005**	0.741	0	
Low	11	-0.42 (-1.58, 0.74)	0.477	1.000	0	0.221
Moderate	4	1.29 (-1.56, 4.15)	0.375	0.092	53.4	
Baseline BMI						
Normal	4	-0.30 (-2.06, 1.45)	0.732	0.939	0	
OW	13	-0.06 (-1.13, 1.00)	0.907	1.000	0	0.921
OB	7	0.25 (-1.78, 2.29)	0.809	0.051	52.1	
Sex						
Both	13	0.07 (-1.13, 1.27)	0.907	0.743	0	
Female	8	-1.12 (-1.86, -0.38)	**0.003**	0.789	0	0.144
Male	3	0.36 (-1.70, 2.43)	0.728	0.901	0	
Age						
≥ 50	13	-1.01 (-1.71, -0.31)	**0.004**	0.931	0	0.080
<50	11	0.22 (-0.97, 1.42)	0.712	0.652	0	
**Impacts of Mg on BMI** (kg/m^2)						
Overall effect	37	-0.65 (-1.49, 0.19)	0.129	<0.001	97.3	
Trial duration (weeks)						
≥ 12	28	-0.66 (-1.75, 0.42)	0.232	<0.001	98.0	0.815
<12	9	-0.52 (-1.00, -0.03)	**0.035**	0.184	29.3	
Mg dose (mg/day)						
≥300	27	-0.83 (-1.96, 0.29)	0.149	<0.001	98.1	0.486
<300	10	-0.41 (-0.70, -0.13)	**0.004**	0.824	0	
Chemical class of Mg supplements						
Organic	7	-0.45 (-0.78, -0.11)	**0.009**	0.414	1.4	
Inorganic	28	-0.85 (-2.16, 0.45)	0.200	<0.001	97.8	0.015
Chelated	2	0.06 (-0.11, 0.25)	0.467	0.796	0	
Bioavailability of Mg supplements						
High	9	-0.13 (-0.53, 0.26)	0.516	0.069	44.9	
Low	12	-0.39 (-0.81, 0.03)	0.071	0.195	25.3	0.488
Moderate	16	-1.16 (-3.29, 0.96)	0.283	<0.001	98.7	
Baseline BMI						
Normal	4	-5.14 (-10.93, 0.65)	0.082	<0.001	99.6	
OW	18	0.01 (-0.14, 0.16)	0.896	0.442	1.1	0.004
OB	15	-0.42 (-0.69, -0.16)	**0.001**	0.464	0	
Sex						
Both	30	-0.73 (-1.79, 0.31)	0.170	<0.001	97.8	0.619
Female	7	-0.46 (-0.75, -0.16)	**0.002**	0.737	0	
Age						
≥ 50	19	-1.06 (-2.92, 0.79)	0.260	<0.001	98.3	0.272
<50	18	- 0.02 (-0.22, 0.18)	0.842	0.322	11.0	

Table S2. *Cont.*

**Sub-groups**	**Effect Sizes** (n)	**WMD** (95% CI)	**P-value**	**Heterogeneity**		
				**P-value**	**I^2** (%)	**P-value between sub-groups**
**Impacts of Mg on WC** (cm)						
Overall effect	22	-0.98 (-2.02, 0.05)	0.061	<0.001	57.9	
Trial duration (weeks)						
≥ 12	17	-0.77 (-1.99, 0.43)	0.209	<0.001	62.8	0.416
<12	5	-1.72 (-3.65, 0.20)	0.080	0.189	34.8	
Mg dose (mg/day)						
≥300	16	-0.67 (-1.98, 0.63)	0.312	<0.001	65.1	0.285
<300	6	-1.75 (-3.22, -0.27)	**0.020**	0.292	18.6	
Chemical class of Mg supplements						
Organic	4	-0.46 (-4.45, 3.51)	0.819	0.008	74.4	
Inorganic	17	-1.00 (-2.11, 0.11)	0.078	0.001	57.9	0.968
Chelated	1	-1.00 (-4.68, 2.68)	0.595	-	-	
Bioavailability of Mg supplements						
High	5	-0.68 (-3.71, 2.33)	0.655	0.019	66.1	
Low	8	-1.78 (-3.24, -0.32)	**0.017**	0.136	36.7	0.442
Moderate	9	-0.39 (-2.02, 1.22)	0.633	0.002	67.4	
Baseline BMI						
Normal	3	-0.40 (-3.97, 3.16)	0.824	<0.001	90.2	
OW	10	-0.60 (-1.69, 0.49)	0.284	0.831	0	0.543
OB	9	-1.78 (-3.65, 0.07)	0.061	0.008	61.4	
Sex						
Both	17	-0.72 (-1.94, 0.49)	0.242	<0.001	63.1	
Female	5	-1.91 (-3.71, -0.10)	**0.038**	0.244	26.6	0.288
Age						
≥ 50	10	-1.20 (-3.14, 0.74)	0.225	<0.001	79.3	0.646
<50	12	-0.69 (-1.67, 0.29)	0.172	0.952	0	
**Impacts of Mg on HC (cm)**						
Overall effect	6	-0.60 (-1.79, 0.59)	0.321	0.983	0	
Trial duration (weeks)						
≥ 12	4	-0.79 (-2.18, 0.59)	0.263	0.936	0	0.601
<12	2	-0.08 (-2.36, 2.19)	0.943	0.973	0	
Mg dose (mg/day)						
≥300	4	-0.52 (-1.87, 0.82)	0.444	0.919	0	0.814
<300	2	-0.86 (-3.40, 1.66)	0.501	0.706	0	
Chemical class of Mg supplements						
Organic	1	-1.00 (-3.73, 1.73)	0.474	-	-	0.752
Inorganic	5	-0.50 (-1.82, 0.81)	0.450	0.964	0	
Bioavailability of Mg supplements						
High	1	-1.00 (-3.73, 1.73)	0.474	-	-	
Low	4	-0.65 (-2.14, 0.84)	0.392	0.933	0	0.877
Moderate	1	0.00 (-2.80, 2.80)	1.000	-	-	
Baseline BMI						
OW	5	-0.49 (-1.78, 0.79)	0.452	0.971	0	0.677
OB	1	-1.20 (-4.25, 1.85)	0.442	-	-	
Sex						
Both	5	-0.63 (-1.86, 0.59)	0.312	0.957	0	0.839
Female	1	-0.15 (-4.66, 4.36)	0.948	-	-	
Age						
≥ 50	3	-0.68 (-2.25, 0.87)	0.388	0.845	0	0.866
<50	3	-0.48 (-2.30, 1.34)	0.605	0.848	0	
**Impacts of Mg on BFP (%)**						
Overall effect	9	-0.02 (-0.46, 0.43)	0.932	0.854	0	
Trial duration (weeks)						
≥ 12	7	0.13 (-0.34, 0.62)	0.573	0.979	0	0.093
<12	2	-0.93 (-2.10, 0.22)	0.113	0.795	0	
Mg dose (mg/day)						
≥300	8	0.12 (-0.35, 0.59)	0.613	0.988	0	0.100
<300	1	-1.00 (-2.25, 0.25)	0.117	-	-	

Table S2. *Cont.*

**Sub-groups**	**Effect Sizes** (n)	**WMD** (95% CI)	**P-value**	**Heterogeneity** P-value	I² (%)	P-value between sub-groups
Chemical class of Mg supplements						
Organic	3	0.10 (-0.40, 0.61)	0.683	0.666	0	0.330
Inorganic	6	-0.40 (-1.30, 0.49)	0.374	0.810	0	
Bioavailability of Mg supplements						
High	3	0.10 (-0.40, 0.61)	0.683	0.666	0	0.588
Low	4	-0.45 (-1.40, 0.48)	0.343	0.561	0	
Moderate	2	0.05 (-2.81, 2.92)	0.968	0.743	0	
Baseline BMI						
Normal	2	0.13 (-0.38, 0.65)	0.610	0.572	0	0.208
OW	5	-0.79 (-1.80, 0.20)	0.118	0.965	0	
OB	2	0.59 (-1.15, 2.43)	0.504	0.964	0	
Sex						
Both	6	0.05 (-1.15, 1.26)	0.924	0.963	0	0.257
Female	1	-1.00 (-2.25, 0.25)	0.117	-	-	
Male	2	0.13 (-0.38, 0.65)	0.610	0.572	0	
Age						
≥ 50	3	-0.39 (-2.36, 1.58)	0.698	0.864	0	0.705
<50	6	0.00 (-0.45, 0.45)	0.998	0.608	0	
**Impacts of Mg on serum FBG** (mg/dL)						
Overall effect	44	-3.60 (-6.13, -1.06)	**0.005**	<0.001	91.4	
Baseline						
≥100	30	-4.91 (-8.38, -1.45)	**0.005**	<0.001	87.9	0.124
<100	14	-1.60 (-4.01, 0.81)	0.194	<0.001	84.2	
Trial duration (weeks)						
≥ 12	32	-4.30 (-7.25, -1.34)	**0.004**	<0.001	92.8	0.233
<12	12	-1.14 (-5.41, 3.13)	0.601	<0.001	74.7	
Mg dose (mg/day)						
≥300	33	-3.99 (-6.94, -1.03)	**0.008**	<0.001	92.5	0.559
<300	11	-2.31 (-7.10, 2.47)	0.343	<0.001	81.0	
Chemical class of Mg supplements						
Organic	12	-1.25 (-5.56, 3.04)	0.567	0.001	64.4	0.005
Inorganic	29	-3.39 (-6.51, -0.26)	**0.033**	<0.001	93.5	
Chelated	2	-5.43 (-10.33, -0.54)	**0.030**	0.107	61.6	
Mixed	1	-20.10 (-29.61, -10.58)	**<0.001**	-	-	
Bioavailability of Mg supplements						
High	14	-2.29 (-6.10, 1.50)	0.236	<0.001	70.8	0.002
Low	13	-1.60 (-4.25, 1.05)	0.237	<0.001	72.4	
Moderate	16	-5.15 (-10.09, -0.22)	**0.040**	<0.001	95.5	
Mixed	1	-20.10 (-29.61, -10.58)	**<0.001**	-	-	
Baseline BMI						
Normal	6	-1.61 (-7.83, 4.61)	0.612	<0.001	95.6	0.652
OW	23	-4.72 (-8.20, -1.23)	**0.008**	<0.001	86.4	
OB	15	-2.95 (-7.81, 1.90)	0.234	<0.001	90.5	
Sex						
Both	36	-3.92 (-6.91, -0.93)	**0.010**	<0.001	92.0	<0.001
Female	6	-4.93 (-9.05, -0.80)	**0.019**	0.001	76.6	
Male	2	5.42 (2.48, 8.36)	**<0.001**	0.702	0	
Age						
≥ 50	24	-3.03 (-5.73, -0.33)	**0.028**	<0.001	70.5	0.751
<50	20	-3.78 (-7.56, -0.00)	0.050	<0.001	94.3	

Table S2. *Cont.*

Sub-groups	Effect Sizes (n)	WMD (95% CI)	P-value	Heterogeneity		
				P-value	I^2 (%)	P-value between sub-groups
**Impacts of Mg on FI** (μIU/mL)						
Overall effect	28	-0.77 (-1.83, 0.30)	0.157	<0.001	82.4	
Trial duration (weeks)						
≥ 12	20	-0.95 (-2.26, 0.35)	0.154	<0.001	85.9	0.505
<12	8	-0.19 (-2.00, 1.62)	0.836	0.045	51.3	
Mg dose (mg/day)						
≥300	19	-0.90 (-1.83, 0.02)	0.058	<0.001	71.4	0.598
<300	9	0.06 (-3.41, 3.55)	0.969	<0.001	91.2	
Chemical class of Mg supplements						
Organic	6	-0.49 (-2.47, 1.49)	0.627	0.051	54.5	<0.001
Inorganic	19	-0.45 (-1.62, 0.70)	0.441	<0.001	80.7	
Chelated	2	0.00 (-1.74, 1.75)	0.998	0.724	0	
Mixed	1	-7.16 (-9.10, -5.21)	**<0.001**	-	-	
Bioavailability of Mg supplements						
High	8	-0.30 (-1.62, 1.02)	0.658	0.132	37.3	<0.001
Low	10	0.36 (-1.43, 2.16)	0.689	<0.001	82.3	
Moderate	9	-1.32 (-2.45, -0.18)	**0.023**	0.013	58.6	
Mixed	1	-7.16 (-9.10, -5.21)	**<0.001**	-	-	
Baseline BMI						
Normal	4	-0.78 (-2.51, 0.95)	0.375	0.018	70.1	0.767
OW	14	-1.20 (-2.83, 0.42)	0.149	<0.001	84.5	
OB	10	-0.21 (-2.32, 1.89)	0.844	<0.001	81.1	
Sex						
Both	22	-0.99 (-2.22, 0.24)	0.117	<0.001	85.4	0.699
Female	5	0.10 (-2.19, 2.39)	0.931	0.046	58.7	
Male	1	-0.40 (-3.38, 2.58)	0.793	-	-	
Age						
≥ 50	12	-0.73 (-2.59, 1.13)	0.440	<0.001	87.7	0.990
<50	16	-0.74 (-2.05, 0.55)	0.262	<0.001	76.9	
**Impacts of Mg on HbA1c (%)**						
Overall effect	27	-0.15 (-0.26, -0.03)	**0.010**	<0.001	68.3	
Trial duration (weeks)						
≥ 12	20	-0.13 (-0.26, -0.00)	**0.040**	<0.001	72.5	0.740
<12	7	-0.18 (-0.44, 0.07)	0.157	0.084	46.2	
Mg dose (mg/day)						
≥300	21	-0.10 (-0.22, 0.01)	0.076	<0.001	66.9	0.355
<300	6	-0.27 (-0.61, 0.05)	0.105	0.001	76.1	
Chemical class of Mg supplements						
Organic	11	-0.07 (-0.23, 0.07)	0.331	0.009	57.6	0.004
Inorganic	15	-0.16 (-0.31, -0.00)	**0.047**	<0.001	69.5	
Mixed	1	-0.83 (-1.24, -0.41)	**<0.001**	-	-	
Bioavailability of Mg supplements						
High	11	-0.07 (-0.23, 0.07)	0.331	0.009	57.6	0.008
Low	7	-0.20 (-0.40, -0.00)	**0.046**	0.007	66.5	
Moderate	8	-0.09 (-0.32, 0.12)	0.399	0.061	48.1	
Mixed	1	-0.83 (-1.24, -0.41)	**<0.001**	-	-	
Baseline BMI						
Normal	4	-0.03 (-0.10, 0.03)	0.359	0.428	0	0.120
OW	12	-0.19 (-0.54, 0.14)	0.263	<0.001	67.3	
OB	11	-0.19 (-0.34, -0.04)	**0.009**	0.003	62.7	
Sex						
Both	25	-0.13 (-0.25, -0.01)	**0.026**	<0.001	66.5	0.011
Female	1	-0.54 (-0.87, -0.20)	**0.002**	-	-	
Male	1	0.00 (-0.13, 0.13)	0.100	-	-	
Age						
≥ 50	22	-0.16 (-0.29, -0.03)	**0.011**	<0.001	71.5	0.105
<50	5	-0.02 (-0.14, 0.10)	0.740	0.565	0	

Table S2. *Cont.*

Sub-groups	Effect Sizes (n)	WMD (95% CI)	P-value	Heterogeneity		
				P-value	I^2 (%)	P-value between sub-groups
**Impacts of Mg on HOMA-IR**						
Overall effect	27	-0.48 (-0.79, -0.18)	**0.002**	<0.001	89.7	
Trial duration (weeks)						
≥ 12	21	-0.60 (-0.95, -0.24)	**0.001**	<0.001	91.5	0.138
<12	6	-0.05 (-0.68, 0.57)	0.867	0.010	66.8	
Mg dose (mg/day)						
≥300	18	-0.55 (-0.88,-0.22)	**0.001**	<0.001	90.2	0.755
<300	9	-0.38 (-1.43, 0.67)	0.480	<0.001	89.9	
Chemical class of Mg supplements						
Organic	6	-0.06 (-0.47, 0.35)	0.763	0.018	63.3	<0.001
Inorganic	18	-0.52 (-0.94, -0.10)	**0.014**	<0.001	91.2	
Chelated	2	-0.03 (-0.51, 0.44)	0.880	0.843	0.0	
Mixed	1	-3.73 (-4.83, -2.62)	**<0.001**	-	-	
Bioavailability of Mg supplements						
High	8	-0.04 (-0.34, 0.26)	0.783	0.057	48.9	<0.001
Low	10	-0.07 (-0.74, 0.60)	0.839	<0.001	84.4	
Moderate	8	-1.05 (-1.72, -0.38)	**0.002**	<0.001	94.8	
Mixed	1	-3.73 (-4.83, -2.62)	**<0.001**	-	-	
Baseline BMI						
Normal	3	-0.89 (-1.91, 0.13)	0.087	<0.001	87.0	0.663
OW	12	-0.48 (-0.92, -0.04)	**0.031**	<0.001	87.8	
OB	12	-0.36 (-0.89, 0.17)	0.185	<0.001	83.1	
Sex						
Both	20	-0.67 (-1.04, -0.29)	**<0.001**	<0.001	92.1	0.068
Female	6	-0.00 (-0.54, 0.53)	0.991	0.014	64.9	
Male	1	0.00 (-0.70, 0.70)	1.000	-	-	
Age						
≥ 50	13	-0.69 (-1.21, -0.17)	**0.009**	<0.001	86.5	0.280
<50	14	-0.31 (-0.77, 0.14)	0.179	<0.001	91.9	
**Impacts of Mg on serum TG** (mg/dL)						
Overall effect	41	-9.24 (-16.87, -1.61)	**0.018**	<0.001	76.0	
Baseline TG						
≥150	22	-21.13 (-32.57, -9.69)	**< 0.001**	<0.001	73.0	<0.001
<150	19	2.61 (-2.68, 7.93)	0.333	0.242	17.3	
Trial duration (weeks)						
≥ 12	31	-11.93 (-21.14, -2.71)	**0.011**	<0.001	79.3	0.021
<12	10	2.05 (-5.46, 9.58)	0.592	0.485	0	
Mg dose (mg/day)						
≥300	30	-13.56 (-23.14, -3.98)	**0.006**	<0.001	74.1	0.027
<300	11	0.76 (-7.58, 9.10)	0.858	0.029	50.2	
Chemical class of Mg supplements						
Organic	14	-2.68 (-18.17, 12.81)	0.734	<0.001	81.1	0.247
Inorganic	25	-13.23 (-22.60, -3.87)	**0.006**	<0.001	72.6	
Chelated	2	3.19 (-17.11, 23.50)	0.758	0.175	45.5	
Bioavailability of Mg supplements						
High	16	-1.99 (-15.74, 11.76)	0.777	<0.001	80.5	0.055
Low	12	-3.28 (-11.66, 5.08)	0.442	0.094	37.2	
Moderate	13	-25.48 (-42.42, -8.54)	**0.003**	<0.001	81.7	
Baseline BMI						
Normal	7	-3.27 (-17.25, 10.70)	0.646	0.008	65.8	0.657
OW	20	-11.71 (-23.19, -0.24)	**0.045**	<0.001	69.6	
OB	14	-7.88 (-20.76, 4.99)	0.230	<0.001	78.3	
Sex						
Both	32	-13.15 (-22.05, -4.25)	**0.004**	<0.001	73.6	0.012
Female	7	4.65 (-4.76, 14.07)	0.332	0.204	29.4	
Male	2	5.10 (-9.06, 19.26)	0.480	0.269	18.3	
Age						
≥ 50	22	-8.82 (-18.69, 1.04)	0.080	<0.001	72.3	0.850
<50	19	-10.32 (-22.30, 1.64)	0.091	<0.001	78.1	

Table S2. *Cont.*

Sub-groups	Effect Sizes (n)	WMD (95% CI)	P-value	Heterogeneity		
				P-value	I^2 (%)	P-value between sub-groups
**Impacts of Mg on serum TC** (mg/dL)						
Overall effect	34	-1.65 (-5.65, 2.35)	0.420	<0.001	65.7	
Baseline TC						
<200	18	1.82 (-2.58, 6.23)	0.418	0.007	51.1	0.043
≥200	16	-5.95 (-12.05, 0.14)	0.056	<0.001	63.4	
Trial duration (weeks)						
≥ 12	26	-1.41 (-6.30, 3.47)	0.570	<0.001	71.1	0.815
<12	8	-2.34 (-8.39, 3.69)	0.447	0.210	27.3	
Mg dose (mg/day)						
≥300	23	-3.11 (-8.15, 1.93)	0.226	<0.001	66.8	0.268
<300	11	1.28 (-4.64, 7.21)	0.670	0.017	53.9	
Chemical class of Mg supplements						
Organic	14	-2.02 (-9.09, 5.03)	0.574	<0.001	69.3	0.828
Inorganic	18	-0.40 (-4.99, 4.18)	0.863	0.002	55.4	
Chelated	2	-7.46 (-33.84, 18.91)	0.579	0.031	78.4	
Bioavailability of Mg supplements						
High	16	-2.58 (-9.16, 3.99)	0.441	<0.001	68.3	0.206
Low	11	2.57 (-1.50, 6.66)	0.216	0.175	28.3	
Moderate	7	-5.68 (-16.22, 4.85)	0.290	0.009	65.0	
Baseline BMI						
Normal	6	1.36 (-5.21, 7.93)	0.684	0.777	0.0	0.457
OW	16	-3.64 (-8.53, 1.25)	0.145	0.031	43.8	
OB	12	-0.14 (-8.48, 8.18)	0.972	<0.001	83.2	
Sex						
Both	24	-1.38 (-6.47, 3.70)	0.595	<0.001	71.6	0.760
Female	8	-3.17 (-10.23, 3.88)	0.378	0.054	49.5	
Male	2	2.09 (-10.14, 14.34)	0.737	0.388	0.0	
Age						
≥ 50	20	-2.35 (-7.87, 3.16)	0.402	<0.001	64.5	0.637
<50	14	-0.47 (-6.01, 5.05)	0.866	0.001	62.5	
**Impacts of Mg on serum LDL-C** (mg/dL)						
Overall effect	30	-3.21 (-5.27, -1.15)	0.002	<0.001	90.3	
Baseline LDL-C						
<100	7	-4.88 (-7.75, -2.02)	**0.001**	<0.001	96.8	0.420
≥100	23	-2.61 (-7.32, 2.09)	0.277	<0.001	74.4	
Trial duration (weeks)						
≥ 12	23	-3.87 (-6.08, -1.66)	**0.001**	<0.001	92.1	0.144
<12	7	0.52 (-4.94, 5.98)	0.852	0.304	16.5	
Mg dose (mg/day)						
≥300	20	-6.31 (-8.57, -4.06)	**<0.001**	<0.001	91.5	<0.001
<300	10	4.58 (-0.29, 9.45)	0.065	0.051	46.7	
Chemical class of Mg supplements						
Organic	8	-8.09 (-14.86, -1.33)	**0.019**	0.033	54.2	0.179
Inorganic	20	-1.61 (-3.92, 0.69)	0.170	<0.001	93.0	
Chelated	2	-10.81 (-42.16, 20.54)	0.499	0.001	90.4	
Bioavailability of Mg supplements						
High	10	-8.28 (-15.27, -1.30)	**0.020**	0.002	65.1	<0.001
Low	11	5.35 (1.85, 8.84)	**0.003**	0.194	26.3	
Moderate	9	-7.42 (-10.10, -4.73)	**<0.001**	<0.001	95.4	
Baseline BMI						
Normal	7	-5.74(-8.70, -2.78)	**<0.001**	< 0.001	96.5	0.271
OW	13	-5.80 (-11.16, -0.44)	**0.034**	0.001	64.1	
OB	10	1.00(-6.77, 8.78)	0.800	<0.001	83.1	
Sex						
Both	22	-3.72 (-5.94, -1.50)	**0.001**	< 0.001	92.2	0.527
Female	7	-2.25 (-10.87,6.36)	0.608	0.002	70.8	
Male	1	8.20 (-13.07, 29.47)	0.450	-	-	
Age						
≥ 50	18	-5.95 (-8.37, -3.53)	**<0.001**	<0.001	92.0	0.042
<50	12	0.72 (-5.23, 6.67)	0.812	<0.001	76.3	

Table S2. *Cont.*

Sub-groups	Effect Sizes (n)	WMD (95% CI)	P-value	Heterogeneity		
				P-value	I^2 (%)	P-value between sub-groups
**Impacts of Mg on serum HDL-C** (mg/dL)						
Overall effect	40	1.45 (0.37, 2.54)	**0.009**	<0.001	97.0	
Baseline HDL-C						
<50	31	1.56 (0.37, 2.76)	**0.010**	<0.001	97.7	0.377
≥50	9	0.59 (-1.20, 2.39)	0.516	0.423	1.3	
Trial duration (weeks)						
≥ 12	33	1.74 (0.55, 2.94)	**0.004**	<0.001	97.5	0.015
<12	7	-0.57 (-2.02, 0.86)	0.433	0.694	0	
Mg dose (mg/day)						
≥300	30	2.04 (0.75, 3.33)	**0.002**	<0.001	97.7	0.016
<300	10	-0.22 (-1.53, 1.09)	0.740	0.097	39.1	
Chemical class of Mg supplements						
Organic	11	1.40 (-1.11, 3.91)	0.274	0.001	67.8	
Inorganic	27	1.34 (0.07, 2.61)	**0.038**	<0.001	97.9	0.838
Chelated	2	3.64 (-3.83, 11.12)	0.339	0.032	78.2	
Bioavailability of Mg supplements						
High	13	1.73 (-0.49, 3.95)	0.127	<0.001	66.7	
Low	12	-0.13 (-1.11, 0.85)	0.795	0.191	25.8	0.032
Moderate	15	2.33 (0.58, 4.08)	**0.009**	<0.001	98.8	
Baseline BMI						
Normal	8	0.69 (-1.82, 3.20)	0.590	<0.001	99.4	
OW	20	1.51 (0.26, 2.76)	**0.017**	<0.001	60.4	0.803
OB	12	1.74 (-0.30, 3.78)	0.095	<0.001	80.2	
Sex						
Both	31	1.80 (0.56, 3.04)	**0.004**	<0.001	97.7	
Female	8	-0.32 (-1.81, 1.16)	0.668	0.221	26.0	0.078
Male	1	3.50 (-3.92, 10.92)	0.355	-	-	
Age						
≥ 50	23	1.46 (-0.04, 2.97)	0.056	<0.001	98.2	0.920
<50	17	1.36 (-0.00, 2.72)	0.050	<0.001	71.6	
**Impacts of Mg on SBP** (mmHg)						
Overall effect	55	-2.50 (-4.08, -0.91)	**0.002**	<0.001	83.6	
Baseline SBP						
≥130	32	-3.23 (-5.80, -0.65)	**0.014**	<0.001	86.7	0.356
<130	23	-1.73 (-3.60, 0.14)	0.070	<0.001	77.2	
Trial duration (weeks)						
≥ 12	39	-3.17 (-5.03, -1.30)	**0.001**	<0.001	83.5	0.056
<12	16	-0.37 (-2.55, 1.79)	0.732	<0.001	63.9	
Mg dose (mg/day)						
≥300	43	-3.25 (-5.11, -1.40)	**<0.001**	<0.001	83.2	0.039
<300	12	-0.02 (-2.48, 2.43)	0.986	<0.001	73.8	
Chemical class of Mg supplements						
Organic	20	-2.75 (-6.05, 0.54)	0.102	<0.001	91.7	
Inorganic	30	-2.41 (-3.91, -0.91)	**0.002**	<0.001	59.7	0.497
Chelated	3	-3.18 (-12.38, 6.01)	0.497	<0.001	86.9	
Mixed	2	2.00 (-3.60, 7.60)	0.484	0.621	0.0	
Bioavailability of Mg supplements						
High	23	-2.80 (-5.82, 0.22)	0.070	<0.001	91.4	
Low	14	-1.94 (-3.68,-0.19)	**0.030**	0.050	41.8	0.459
Moderate	16	-2.78 (-5.25, -0.32)	**0.027**	<0.001	69.0	
Mixed	2	2.00 (-3.60, 7.60)	0.484	0.621	0.0	
Baseline BMI						
Normal	8	-0.74 (-3.03, 1.54)	0.524	0.063	47.8	
OW	34	-1.99 (-3.92, -0.06)	**0.043**	<0.001	82.2	0.258
OB	13	-4.62 (-8.68, -0.56)	**0.026**	<0.001	85.1	
Sex						
Both	45	-2.60 (-4.58, -0.63)	**0.010**	<0.001	85.5	
Female	8	-1.88 (-4.04, 0.26)	0.086	0.010	61.9	0.416
Male	2	0.55 (-3.71, 4.83)	0.798	0.352	0.0	
Age						
≥ 50	36	-2.70 (-5.13, -0.27)	**0.029**	<0.001	87.8	0.702
<50	19	-2.14 (-3.67, -0.60)	**0.006**	0.002	55.8	

Table S2. *Cont.*

**Sub-groups**	**Effect Sizes** (n)	**WMD** (95% CI)	**P-value**	**Heterogeneity**		
				**P-value**	**I**^{2} (%)	**P-value between sub-groups**
**Impacts of Mg on DBP** (mmHg)						
Overall effect	55	-1.58 (-2.50, -0.65)	**0.001**	<0.001	82.2	
Baseline DBP						
≥80	26	-1.77 (-3.22, -0.32)	**0.016**	<0.001	86.7	0.704
<80	29	-1.41 (-2.59, -0.22)	**0.019**	<0.001	75.9	
Trial duration (weeks)						
≥ 12	39	-2.22 (-3.20, -1.25)	**<0.001**	<0.001	79.3	0.007
<12	16	0.23 (-1.26, 1.73)	0.761	<0.001	67.3	
Mg dose (mg/day)						
≥300	43	-1.95 (-2.91, -0.99)	**<0.001**	<0.001	77.5	0.233
<300	12	-0.38 (-2.78, 2.00)	0.752	<0.001	89.6	
Chemical class of Mg supplements						
Organic	20	-0.97 (-2.52, 0.57)	0.217	<0.001	84.8	0.100
Inorganic	30	-2.19 (-3.39, -0.99)	**<0.001**	<0.001	78.2	
Chelated	3	-2.10 (-6.55, 2.34)	0.353	0.001	86.5	
Mixed	2	2.80 (-1.18, 6.78)	0.169	0.243	26.8	
Bioavailability of Mg supplements						
High	23	-1.12 (-2.52, 0.28)	0.118	<0.001	84.5	0.047
Low	14	-1.21 (-3.04, 0.62)	0.195	<0.001	78.4	
Moderate	16	-2.97 (-4.62,-1.33)	**<0.001**	<0.001	78.8	
Mixed	2	2.80 (-1.18, 6.78)	0.169	0.243	26.8	
Baseline BMI						
Normal	8	-1.15 (-3.22, 0.91)	0.274	<0.001	77.8	0.225
OW	34	-1.27 (-2.55, 0.00)	0.051	<0.001	85.0	
OB	13	-2.80 (-4.22, -1.39)	**<0.001**	0.002	61.8	
Sex						
Both	45	-1.70 (-2.78, -0.63)	**0.002**	<0.001	83.6	0.687
Female	8	-1.25 (-3.18, 0.67)	0.203	<0.001	71.9	
Male	2	0.12 (-4.18, 4.43)	0.955	0.061	71.5	
Age						
≥ 50	36	-1.34 (-2.53, -0.15)	**0.027**	<0.001	83.3	0.481
<50	19	-2.03 (-3.48, -0.55)	**0.007**	<0.001	79.8	
**Impacts of Mg on CRP**(mg/L)						
Overall effect	17	0.15 (-0.01,0.30)	0.064	0.010	50.1	
Trial duration (weeks)						
≥ 12	12	0.13 (-0.02, 0.30)	0.101	0.006	58.0	0.688
<12	5	0.25 (-0.29, 0.80)	0.363	0.259	24.4	
Mg dose (mg/day)						
≥300	11	0.05 (-0.05, 0.16)	0.316	0.166	29.3	0.078
<300	6	0.55 (0.01, 1.09)	**0.046**	0.059	53.0	
Chemical class of Mg supplements						
Organic	7	0.15 (-0.11, 0.43)	0.251	0.121	40.5	0.835
Inorganic	10	0.20 (-0.07, 0.47)	0.159	0.009	59.0	
Bioavailability of Mg supplements						
High	7	0.15 (-0.11, 0.43)	0.251	0.121	40.5	0.202
Low	6	0.48 (-0.02, 0.99)	0.062	0.060	52.8	
Moderate	4	0.00 (-0.19, 0.20)	0.945	0.194	36.4	
Baseline BMI						
Normal	5	0.22(-0.09, 0.53)	0.170	0.019	66.0	0.459
OW	6	0.00 (-0.33, 0.34)	0.984	0.218	28.9	
OB	6	0.36 (-0.13, 0.85)	0.157	0.116	43.4	
Sex						
Both	13	0.15 (-0.00, 0.32)	0.060	0.005	57.8	0.533
Female	3	-0.00 (-0.62, 0.61)	0.984	0.296	17.9	
Male	1	1.55 (-1.18, 4.28)	0.267	-	-	
Age						
≥ 50	9	0.13 (-0.02, 0.28)	0.100	0.009	60.6	0.998
<50	8	0.13 (-0.37, 0.64)	0.611	0.127	37.9	

Table S2. *Cont.*

Sub-groups	Effect Sizes (n)	WMD (95% CI)	P-value	Heterogeneity		
				P-value	I^2 (%)	P-value between sub-groups
**Impacts of Mg on IL-6** (pg/mL)						
Overall effect	8	-1.10 (-1.93, -0.27)	**0.009**	<0.001	96.9	
Trial duration (weeks)						
≥ 12	5	-4.83 (-9.77, 0.10)	0.055	<0.001	98.1	0.043
<12	3	0.31 (-0.35, 0.97)	0.359	0.123	52.3	
Mg dose (mg/day)						
≥300	7	-3.81 (-6.57, -1.06)	**0.007**	<0.001	97.3	<0.001
<300	1	0.18 (0.04, 0.31)	**0.007**	-	-	
Chemical class of Mg supplements						
Organic	4	-5.05(-8.86, -1.20)	**0.010**	<0.001	98.6	0.027
Inorganic	4	-0.30 (-2.01, 1.40)	0.729	0.008	74.9	
Bioavailability of Mg supplements						
High	4	-5.03 (-8.86, -1.20)	**0.010**	<0.001	98.6	
Low	3	0.51 (-0.85, 1.88)	0.461	0.067	63.0	0.003
Moderate	1	-3.50 (-6.32, -0.67)	**0.015**	-	-	
Baseline BMI						
Normal	1	1.22 (-0.11, 2.55)	0.073	-	-	
OW	4	0.11 (-0.12, 0.35)	0.352	0.011	73.0	0.125
OB	3	-8.76 (-22.68, 5.14)	0.217	<0.001	98.5	
Sex						
Both	7	-3.81 (-6.57, -1.06)	**0.007**	<0.001	97.3	0.005
Female	1	0.18 (0.04, 0.31)	**0.007**	-	-	
Age						
≥ 50	4	-5.19 (-11.04, 0.65)	0.082	<0.001	98.6	0.092
<50	4	- 0.09 (-1.13, 0.95)	0.865	0.013	72.1	
**Impacts of Mg on TNF-α** (pg/mL)						
Overall effect	5	0.15 (-0.11, 0.42)	0.261	0.100	48.6	
Trial duration (weeks)						
≥ 12	3	0.10 (-0.06, 0.27)	0.234	0.252	27.5	0.043
<12	2	1.17 (0.15, 2.19)	**0.024**	0.336	0.0	
Mg dose (mg/day)						
≥300	4	0.11 (-0.13, 0.36)	0.379	0.119	48.7	0.167
<300	1	0.93 (-0.20, 2.06)	0.108	-	-	
Chemical class of Mg supplements						
Organic	3	0.10 (-0.73, 0.94)	0.804	0.121	52.7	0.649
Inorganic	2	0.34 (-0.22, 0.90)	0.237	0.212	35.7	
Bioavailability of Mg supplements						
High	3	0.10 (-0.73, 0.94)	0.804	0.121	52.7	
Low	1	0.93 (-0.20, 2.06)	0.108	-	-	0.445
Moderate	1	0.20 (0.02, 0.37)	**0.029**	-	-	
Baseline BMI						
OW	4	0.28 (-0.43, 1.00)	0.441	0.086	54.5	0.827
OB	1	0.20 (0.02, 0.37)	**0.029**	-	-	
Sex						
Both	4	0.11 (-0.13, 0.36)	0.379	0.119	48.7	0.167
Female	1	0.93 (-0.20, 2.06)	0.108	-	-	
Age						
≥ 50	2	0.01(-0.18, 0.20)	0.891	0.355	0	0.165
<50	3	0.64 (-0.22, 1.51)	0.146	0.114	53.9	

Table S2. *Cont.*

Sub-groups	Effect Sizes (n)	WMD (95% CI)	P-value	Heterogeneity		
				P-value	I^2 (%)	P-value between sub-groups
**Impacts of Mg on AST** (IU/L)						
Overall effect	6	-1.04 (-2.75, 0.68)	0.237	<0.001	80.0	
Trial duration (weeks)						
≥ 12	4	-1.56 (-3.63, 0.49)	0.137	0.004	77.9	0.147
<12	2	0.22 (-1.04, 1.49)	0.729	0.398	0.0	
Mg dose (mg/day)						
≥300	5	-1.47 (-3.23, 0.28)	0.100	0.006	72.3	0.087
<300	1	0.50 (-0.92, 1.92)	0.490	-	-	
Chemical class of Mg supplements						
Organic	2	0.22 (-1.04, 1.49)	0.729	0.398	0.0	0.147
Inorganic	4	-1.56 (-3.63, 0.49)	0.137	0.004	77.9	
Bioavailability of Mg supplements						
High	2	0.22 (-1.04, 1.49)	0.729	0.398	0.0	
Low	1	3.50 (0.30, 6.70)	**0.032**	-	-	<0.001
Moderate	3	-2.64 (-3.45, -1.82)	**<0.001**	0.871	0.0	
Baseline BMI						
Normal	3	-1.60 (-3.50, 0.29)	0.098	<0.001	85.3	
OW	1	-3.77 (-8.04, 0.50)	0.084	-	-	0.261
OB	2	1.25 (-3.01, 5.52)	0.566	0.045	75.1	
Sex						
Both	5	-1.47 (-3.23, 0.28)	0.100	0.006	72.3	0.087
Male	1	0.50 (-0.92, 1.92)	0.490	-	-	
Age						
≥ 50	2	-2.60 (-3.43,-1.77)	**<0.001**	1.000	-	0.025
<50	4	0.12 (-2.11, 2.36)	0.911	0.045	62.7	
**Impacts of Mg on ALT** (IU/L)						
Overall effect	6	-3.03 (-7.33, 1.26)	0.166	<0.001	95.0	
Trial duration (weeks)						
≥ 12	4	-3.10 (-9.55, 3.34)	0.345	<0.001	96.1	0.793
<12	2	-2.14 (-5.35, 1.06)	0.190	0.054	73.0	
Mg dose (mg/day)						
≥300	5	-3.43 (-8.54, 1.66)	0.187	<0.001	94.9	0.320
<300	1	-0.70 (-2.44, 1.04)	0.431	-	-	
Chemical class of Mg supplements						
Organic	2	-2.14 (-5.35, 1.06)	0.190	0.054	73.0	0.793
Inorganic	4	-3.10 (-9.55, 3.34)	0.345	<0.001	96.1	
Bioavailability of Mg supplements						
High	2	-2.14 (-5.35, 1.06)	0.190	0.054	73.0	
Low	1	13.15 (6.33, 19.96)	**<0.001**	-	-	<0.001
Moderate	3	-7.38 (-13.25, -1.52)	**0.014**	<0.001	95.7	
Baseline BMI						
Normal	3	-3.91 (-7.96, 0.12)	0.058	<0.001	91.6	
OW	1	-10.62 (-12.55, -8.68)	**<0.001**	-	-	0.004
OB	2	4.28 (-12.51, 21.08)	0.617	<0.001	95.2	
Sex						
Both	5	-3.43 (-8.54, 1.66)	0.187	<0.001	94.9	0.320
Male	1	-0.70 (-2.44, 1.04)	0.431	-	-	
Age						
≥ 50	2	-5.72 (-12.68, 1.22)	0.106	<0.001	93.9	0.364
<50	4	-1.24 (-7.97, 5.48)	0.718	<0.001	96.4	

Table S2. *Cont.*

Sub-groups	Effect Sizes (n)	WMD (95% CI)	P-value	Heterogeneity		
				P-value	I^2 (%)	P-value between sub-groups
**Impacts of Mg on Cr (mg/dL)**						
Overall effect	13	-0.04 (-0.08, 0.01)	0.106	<0.001	75.9	
Trial duration (weeks)						
≥ 12	10	-0.04 (-0.09, 0.00)	0.102	<0.001	71.9	0.787
<12	3	-0.02 (-0.13, 0.08)	0.634	0.004	82.2	
Mg dose (mg/day)						
≥300	10	-0.05 (-0.10, -0.00)	**0.032**	<0.001	73.0	0.017
<300	3	0.03 (-0.01, 0.08)	0.212	0.729	0.0	
Chemical class of Mg supplements						
Organic	6	-0.02 (-0.06, 0.02)	0.395	0.034	58.5	0.289
Inorganic	7	-0.06 (-0.12, 0.00)	0.052	0.061	50.2	
Bioavailability of Mg supplements						
High	6	-0.02 (-0.06, 0.02)	0.395	0.034	58.5	
Low	3	-0.02 (-0.09, 0.05)	0.545	0.900	0.0	0.469
Moderate	4	-0.08 (-0.16, 0.00)	0.070	0.079	55.8	
Baseline BMI						
Normal	3	-0.03 (-0.16, 0.10)	0.650	<0.001	92.7	
OW	5	-0.02 (-0.07, 0.03)	0.432	0.387	3.5	0.835
OB	5	-0.04 (-0.09, 0.00)	0.094	0.139	42.3	
Sex						
Both	11	-0.05 (-0.09, -0.00)	**0.027**	<0.001	70.0	
Female	1	0.00 (-0.11, 0.11)	1.000	-	-	0.041
Male	1	0.04 (-0.01, 0.09)	0.153	-	-	
Age						
≥ 50	10	-0.03 (-0.08, 0.01)	0.127	<0.001	73.0	0.968
<50	3	-0.04 (-0.18, 0.10)	0.565	0.004	82.2	

Abbreviations: BMI, body mass index; BFP, body fat percentage; Mg, magnesium; WC, waist circumference; SBP, systolic blood pressure; DBP, diastolic blood pressure; HC, hip circumference; BW, body weight; ALT, alanine aminotransferase; AST, aspartate aminotransferase; TG, triglycerides; TC, total cholesterol, LDL-C, low-density lipoproteins cholesterol; HDL-C, high-density lipoprotein cholesterol; FBG, fasting blood glucose; HbA1c, hemoglobin A1c; HOMA-IR, homeostatic model assessment of insulin resistance; CRP, C-reactive protein; IL-6, interleukin-6; TNF-α, tumor necrosis factor alpha; WMD, weighted mean difference; CI, confidence interval; OW, overweight; OB, obesity; Cr, creatinine; FI, fasting insulin. Bold numbers indicate statistical significance ($p < 0.05$).

Table S3. Risk of bias assessment

Reference	**Bias arising from the randomization process** (Allocation bias)	**Bias due to deviations from the intended interventions** (Performance bias)	**Bias due to missing outcome data** (Attrition bias)	**Bias in the measurement of the Outcome** (Detection bias)	**Bias in the selection of reported results** (Reporting bias)	**The overall risk of bias**
Afitska et al. 2021	L	L	L	L	L	L
Alizadeh et al. 2021	L	L	L	L	L	L
Albaker et al. 2022	H	U	L	L	U	H
Barragán-Rodríguez et al. 2008	U	U	L	L	U	H
Borrello et al.1996	L	L	L	L	L	L
Cappuccio et al.1985	L	L	L	L	L	L
Chacko et al.2011	L	L	L	L	L	L
Cunha et al.2017	L	L	L	L	L	L
Cosaro et al.2014	L	L	L	L	L	L
Day et al.2010	L	L	L	L	L	L
de Lourdes et al.1998	U	L	L	L	L	U
de Souza et al.2014	L	L	L	L	L	L
de Valk et al.1998	H	U	L	L	U	H
Drenthen et al.2024	L	L	L	L	L	L
Dyckner & Wester 1983	H	U	L	L	U	H
Eibl et al.1995	L	L	L	L	L	L
Abbasi et al.2012	L	L	L	L	L	L
ELDerawi et al. 2019	H	U	L	L	U	H
Farsinejad-Marj et al.2020	L	L	L	L	L	L
Ferrara et al.1992	L	L	L	L	L	L
Gullestad et al.1994	L	L	L	L	L	L
Guerrero-Romero et al.2004	L	L	L	L	L	L
Guerrero-Romero & Rodríguez-Morán 2009	L	L	L	L	L	L
Guerrero-Romero et al.2015	L	L	L	L	L	L
Guerrero-Romero & Rodríguez-Morán 2011	L	L	L	L	L	L
Halawa et al. 2023	H	U	L	L	U	H
Hasan et al.2017	H	U	L	L	U	H
Henderson et al. 1986	L	L	L	L	L	L
Itoh et al.1997	U	L	L	L	L	U
Joris et al. 2017	L	L	L	L	L	L
Karandish et al. 2013	L	L	L	L	L	L
Kazaks et al.2010	L	L	L	L	L	L
Khalid et al.2026	U	U	L	L	U	H
Kisters et al.2024	L	L	L	L	L	L
Lee et al.2009	L	L	L	L	L	L
Lind et al.1991	U	L	L	L	L	U
Lutsey et al.2018	L	L	L	L	L	L
Marken et al.1989	L	L	L	L	L	L
Mortazavi et al.2013	L	L	L	L	L	L
Mooren et al.2011	L	L	L	L	L	L
Moslehi et al.2012	L	L	L	L	L	L
Navarrete-Cortes et al.2014	L	L	L	L	L	L
Nowson & Morgan 1989	L	L	L	L	L	L
Paolisso et al.1994	L	L	L	L	L	L
Paolisso et al.1989	H	U	L	L	U	H
Poikolainen & Alho 2008	L	L	L	L	L	L
Pokan et al.2006	L	L	L	L	L	L

Table S3. *Cont.*

**Reference**	**Bias arising from the randomization process** (Allocation bias)	**Bias due to deviations from the intended interventions** (Performance bias)	**Bias due to missing outcome data** (Attrition bias)	**Bias in the measurement of the Outcome** (Detection bias)	**Bias in the selection of reported results** (Reporting bias)	**The overall risk of bias**
Rajizadeh et al.2017	L	L	L	L	L	L
Rasmussen et al. 1989	L	L	L	L	L	L
Rashvand et al.2019	L	L	L	L	L	L
Rodríguez-Morán & Guerrero-Romero 2003	L	L	L	L	L	L
Rodríguez-Morán & Guerrero-Romero 2014	L	L	L	L	L	L
Rodríguez-Morán et al.2018	L	L	L	L	L	L
Rodríguez-Ramírez et al.2017	L	L	L	L	L	L
Rylander & Arnaud 2004	L	L	L	L	L	L
Sacks et al.1998	U	L	L	L	L	U
Sadeghian et al.2020	L	L	L	L	L	L
Salehidoost et al.2022	L	L	L	L	L	L
Sanjuliani et al.1996	L	L	L	L	L	L
Schutten et al.2022	L	L	L	L	L	L
Shahmoradi et al.2024	L	L	L	L	L	L
Shechter et al.2000	L	L	L	L	L	L
Simental-Mendía et al.2012	L	L	L	L	L	L
Simental-Mendía et al.2014	L	L	L	L	L	L
Solati et al.2014	L	L	L	L	L	L
Solati et al.2019	L	L	L	L	L	L
Sobhani et al.2020	L	L	L	L	L	L
Toprak et al.2017	L	L	L	L	L	L
Walker et al.2002	L	L	L	L	L	L
Widman et al.1993	L	L	L	L	L	L
Wary et al.1999	L	L	L	L	L	L
Wirell et al.1994	L	L	L	L	L	L
Witteman et al.1994	L	L	L	L	L	L
Yang et al.2026	L	L	L	L	L	L
Yamamoto et al.1995	L	L	L	L	L	L
Zanforlini et al.2022	L	L	L	L	L	L
Zemel et al.1990	L	L	L	L	L	L
Zorbas et al.1999	H	U	L	L	U	H

Abbreviations: L, low risk of bias; H, high risk of bias; U, unclear risk of bias (some concerns)

Table S4. GRADE assessment

Outcomes	Risk of bias	Inconsistency	Indirectness	Imprecision	Publication bias	Quality of evidence
**BW**	No serious limitation	No serious limitation	No serious limitation	No serious limitation	Serious limitation[3]	⊕⊕⊕⊖ Moderate
**BMI**	No serious limitation	Serious limitation[1]	No serious limitation	No serious limitation	No serious limitation	⊕⊕⊕⊖ Moderate
**WC**	No serious limitation	Serious limitation[1]	No serious limitation	Serious limitation[2]	No serious limitation	⊕⊕⊖⊖ Low
**HC**	No serious limitation	No serious limitation	No serious limitation	Serious limitation[2]	No serious limitation	⊕⊕⊕⊖ Moderate
**BFP**	No serious limitation	No serious limitation	No serious limitation	No serious limitation	No serious limitation	⊕⊕⊕⊕ High
**FBG**	No serious limitation	Serious limitation[1]	No serious limitation	Serious limitation[2]	No serious limitation	⊕⊕⊖⊖ Low
**HbA1c**	No serious limitation	Serious limitation[1]	No serious limitation	No serious limitation	No serious limitation	⊕⊕⊕⊖ Moderate
**FI**	No serious limitation	Serious limitation[1]	No serious limitation	Serious limitation[2]	No serious limitation	⊕⊕⊖⊖ Low
**HOMA-IR**	No serious limitation	Serious limitation[1]	No serious limitation	No serious limitation	No serious limitation	⊕⊕⊕⊖ Moderate
**TG**	No serious limitation	Serious limitation[1]	No serious limitation	Serious limitation[2]	Serious limitation[3]	⊕⊖⊖⊖ Very Low
**TC**	No serious limitation	Serious limitation[1]	No serious limitation	Serious limitation[2]	No serious limitation	⊕⊕⊖⊖ Low
**LDL-C**	No serious limitation	Serious limitation[1]	No serious limitation	Serious limitation[2]	Serious limitation[3]	⊕⊖⊖⊖ Very Low
**HDL-C**	No serious limitation	Serious limitation[1]	No serious limitation	Serious limitation[2]	No serious limitation	⊕⊕⊖⊖ Low
**SBP**	No serious limitation	Serious limitation[1]	No serious limitation	Serious limitation[2]	No serious limitation	⊕⊕⊖⊖ Low
**DBP**	No serious limitation	Serious limitation[1]	No serious limitation	No serious limitation	No serious limitation	⊕⊕⊕⊖ Moderate
**CRP**	No serious limitation	Serious limitation[1]	No serious limitation	No serious limitation	No serious limitation	⊕⊕⊕⊖ Moderate
**IL-6**	No serious limitation	Serious limitation[1]	No serious limitation	No serious limitation	No serious limitation	⊕⊕⊕⊖ Moderate
**TNF-α**	No serious limitation	No serious limitation	No serious limitation	No serious limitation	No serious limitation	⊕⊕⊕⊕ High
**AST**	No serious limitation	Serious limitation[1]	No serious limitation	Serious limitation[2]	No serious limitation	⊕⊕⊖⊖ Low
**ALT**	No serious limitation	Serious limitation[1]	No serious limitation	Serious limitation[2]	No serious limitation	⊕⊕⊖⊖ Low
**Cr**	No serious limitation	Serious limitation[1]	No serious limitation	No serious limitation	No serious limitation	⊕⊕⊕⊖ Moderate

Abbreviations: BMI, body mass index; BFP, body fat percentage; WC, waist circumference; SBP, systolic blood pressure; BW, body weight; DBP, diastolic blood pressure; HC, hip circumference; ALT, alanine aminotransferase; AST, aspartate aminotransferase; TG, triglycerides; TC, total cholesterol, LDL-C, low-density lipoprotein cholesterol; HDL-C, high-density lipoprotein cholesterol; FBG, fasting blood glucose; HbA1c, hemoglobin A1c; HOMA-IR, homeostatic model assessment of insulin resistance; FI, fasting insulin; CRP, C-reactive protein; IL-6, interleukin-6; Cr, creatinine; TNF-α, tumor necrosis factor alpha.

[1] It was downgraded due to the presence of significant heterogeneity (I^2>50%).
[2] It was downgraded because the confidence interval for the effect size range was > 2.
[3] It was downgraded due to publication bias based on Begg's or Egger's test.

A) BW

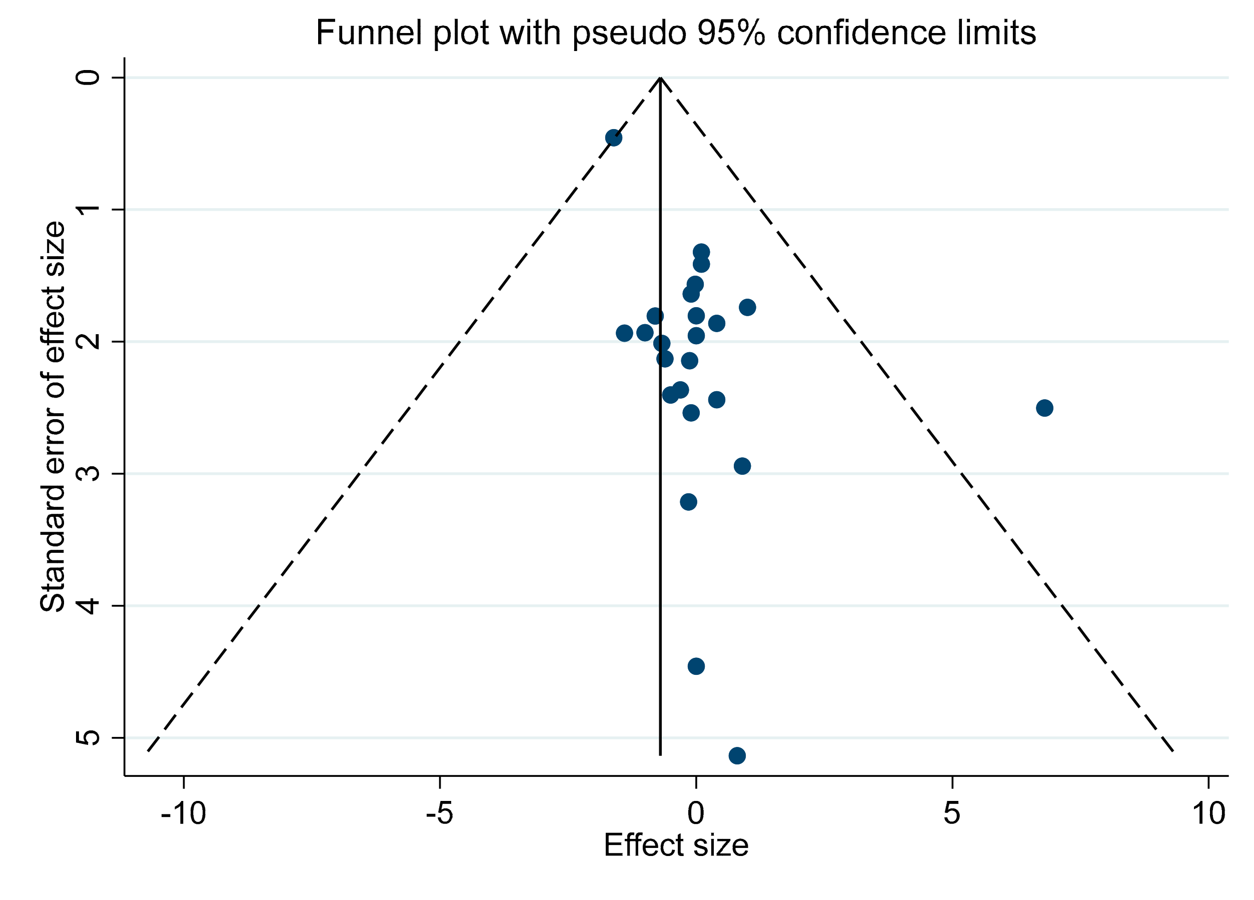

B) BMI

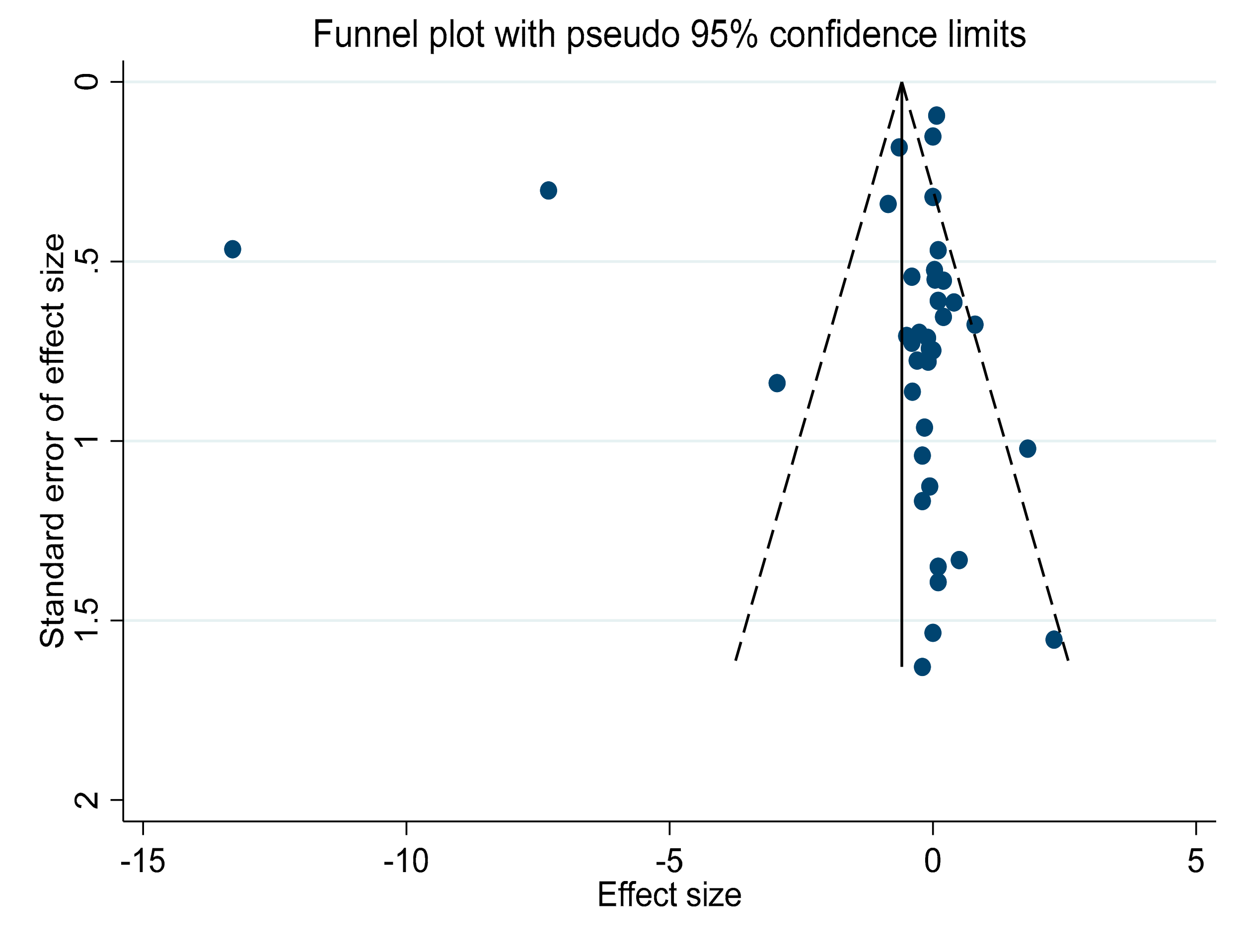

Figure S1. *Cont.*

C) WC

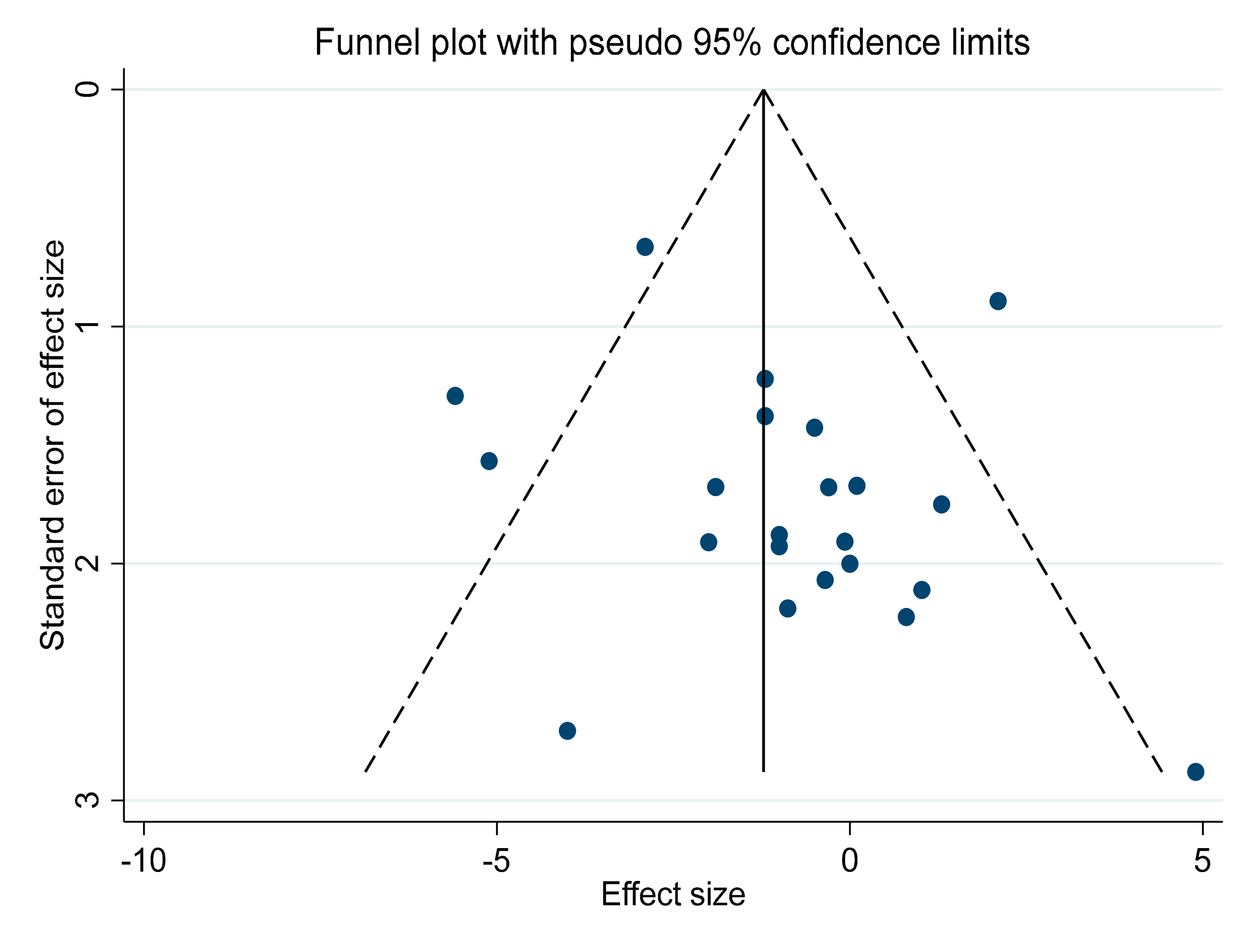

D) HC

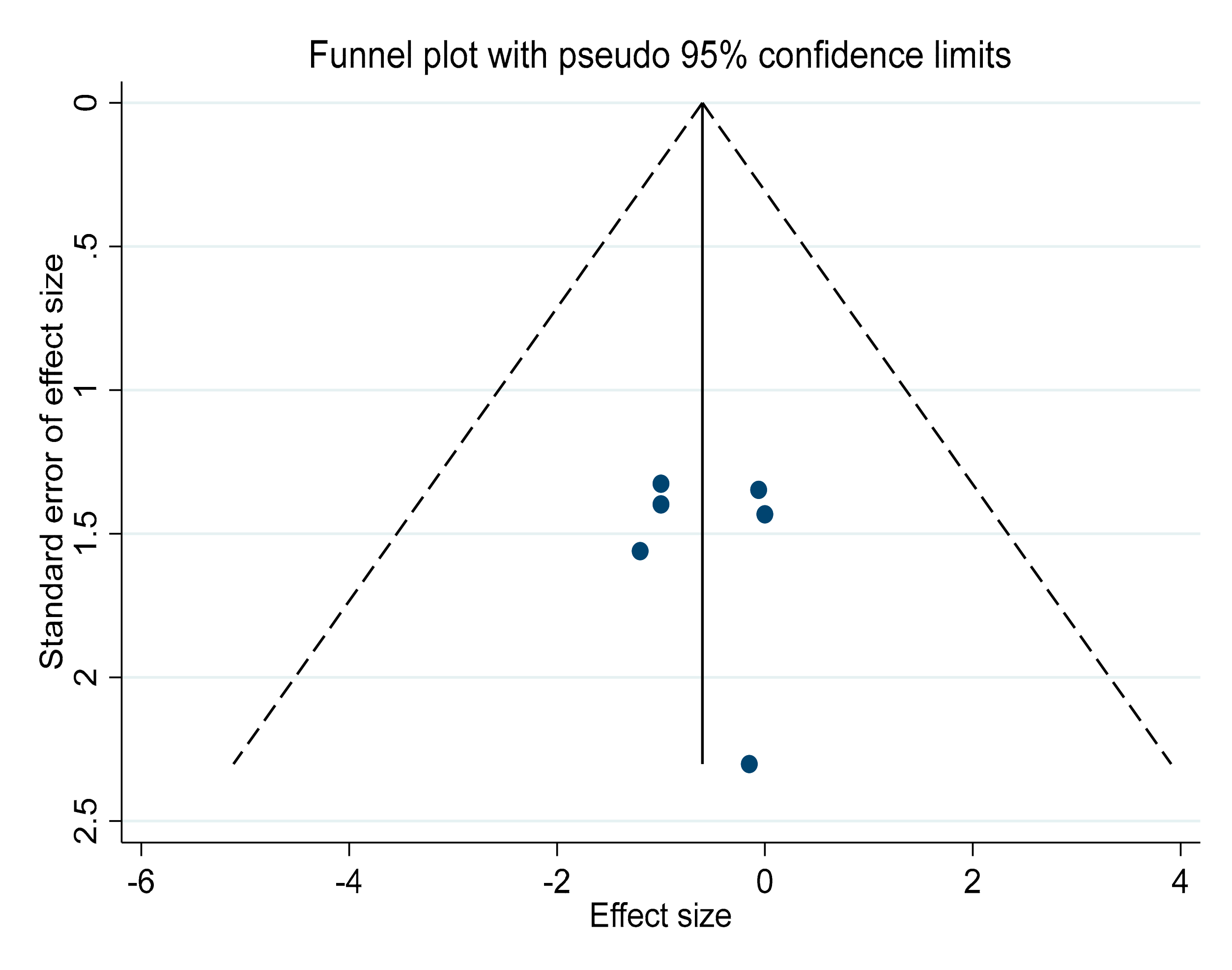

Figure S1. *Cont.*

E) BFP

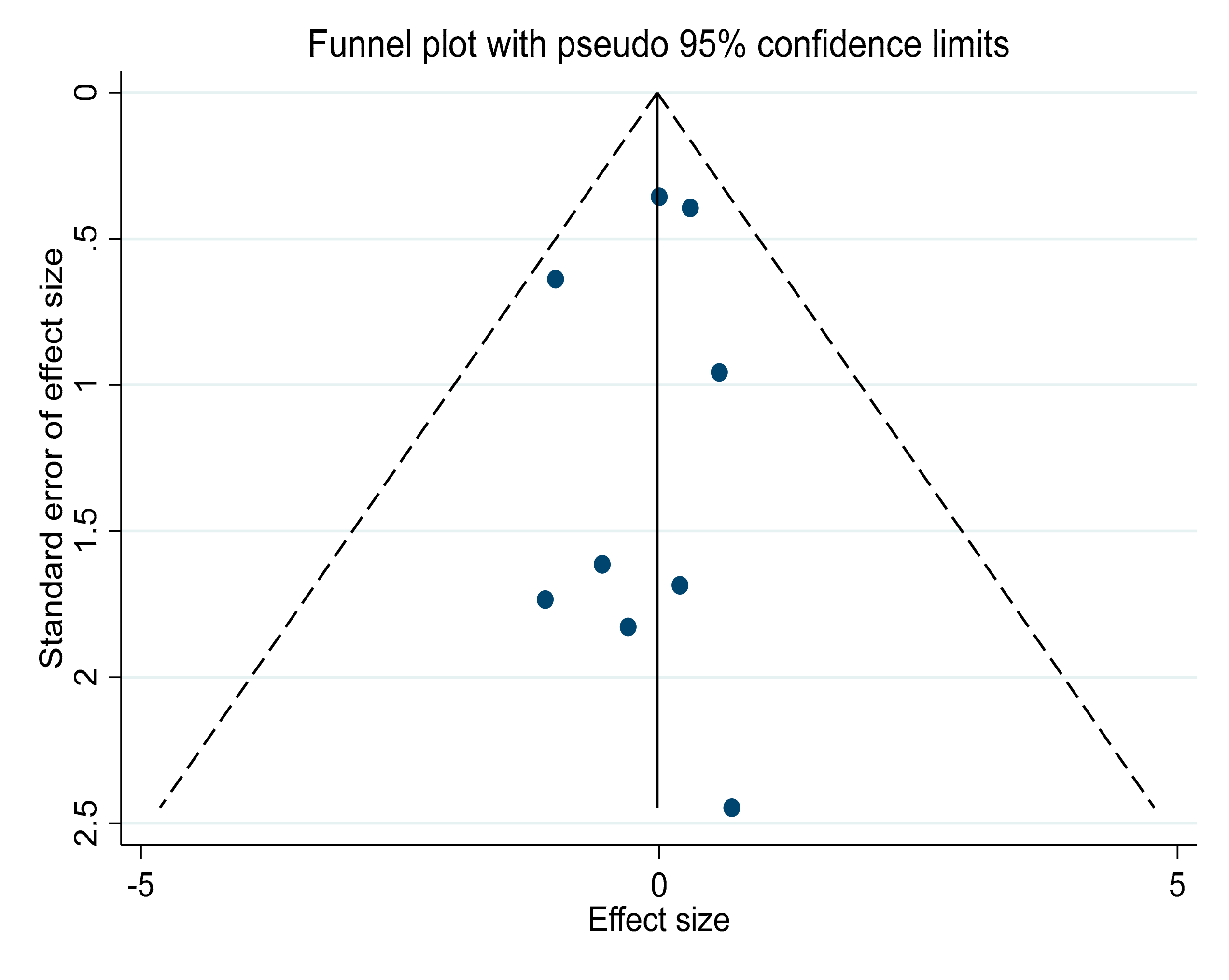

F) FBG

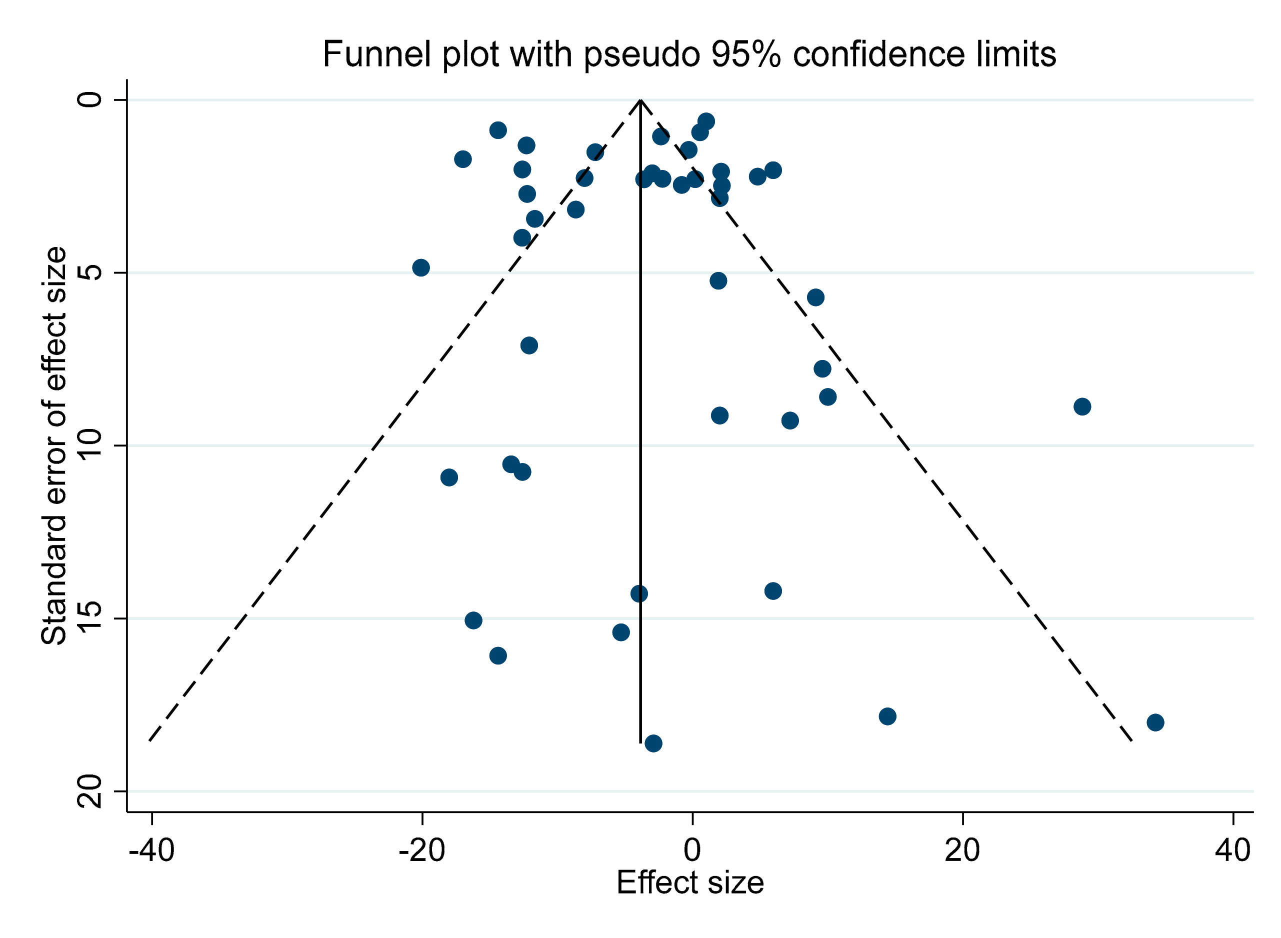

Figure S1. *Cont.*

G) HbA1c

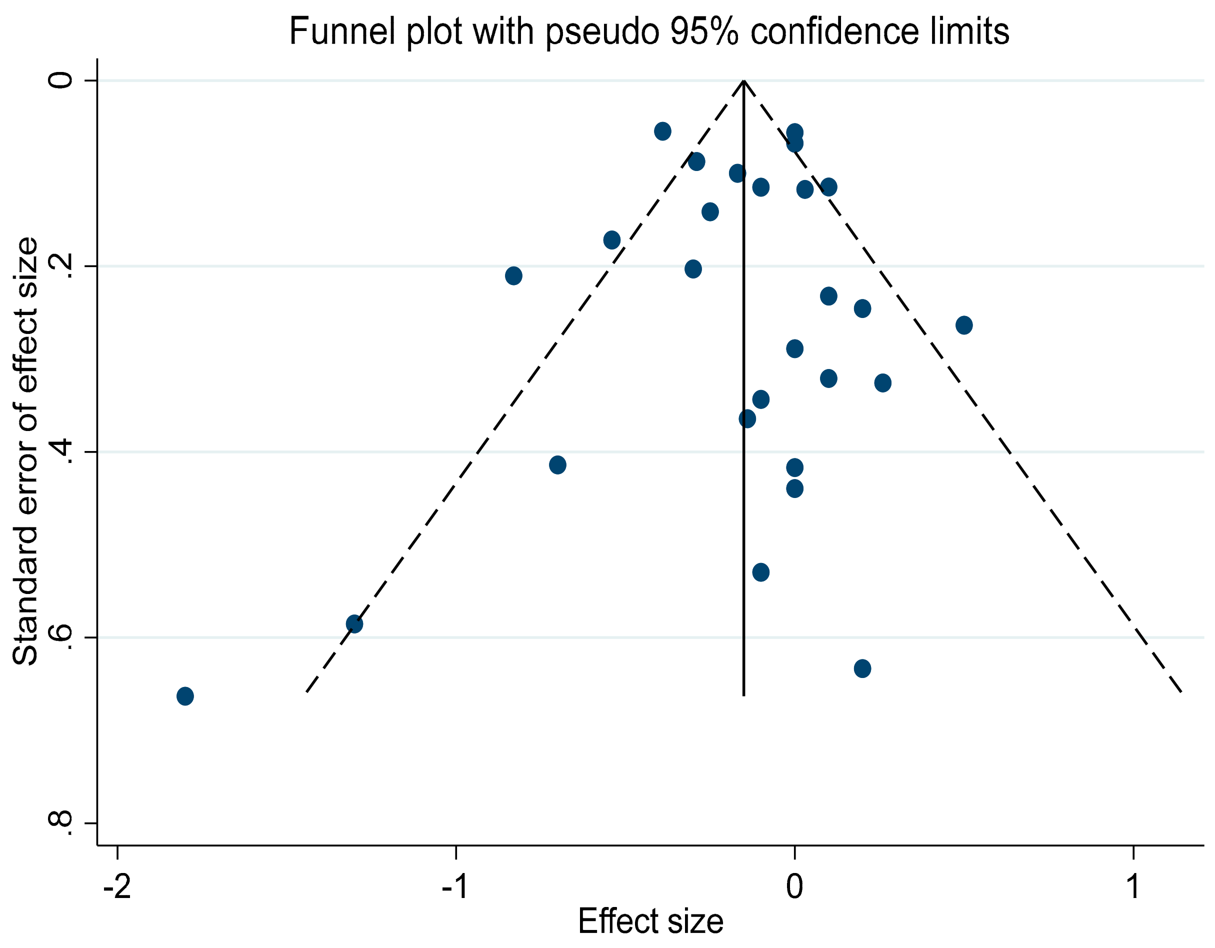

H) FI

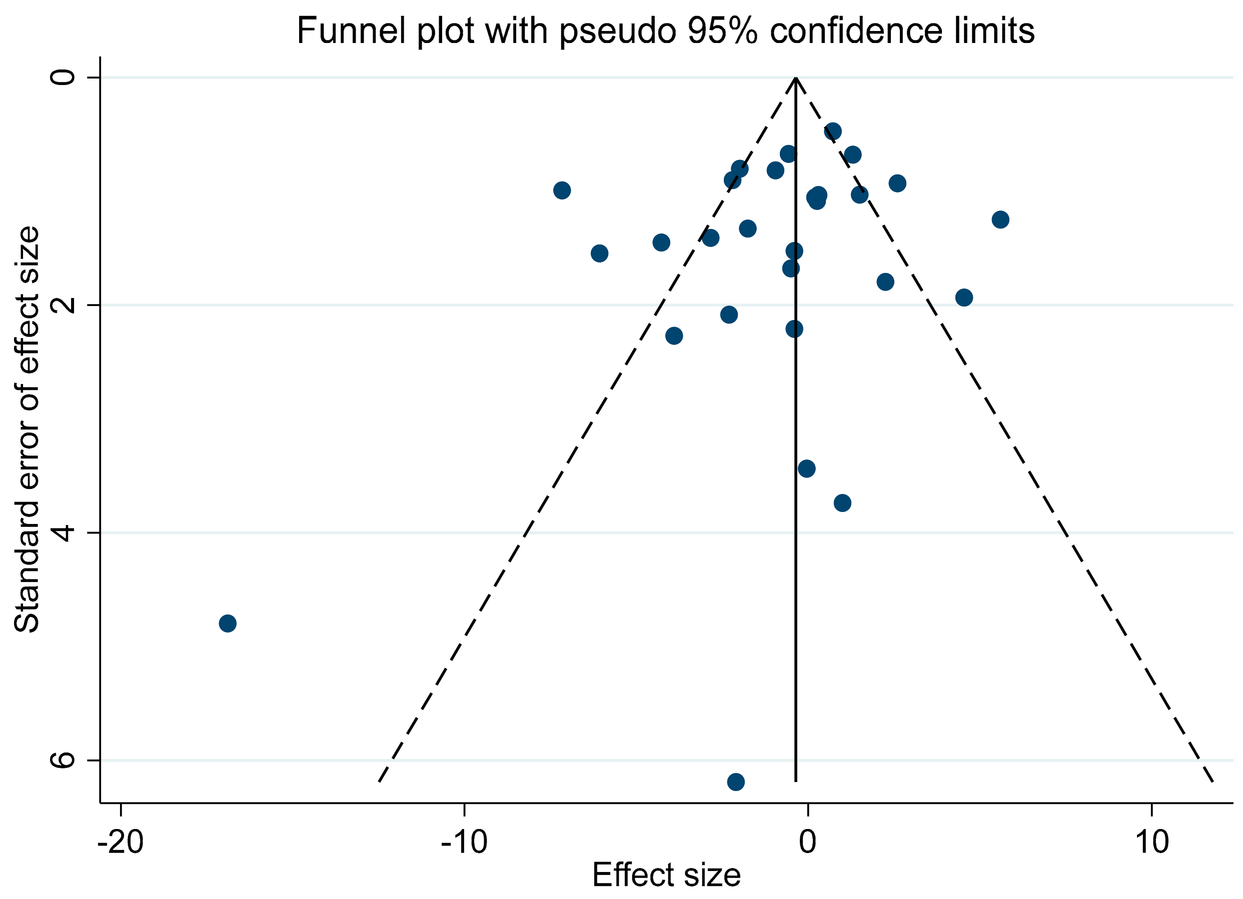

Figure S1. *Cont.*

I) HOMA-IR

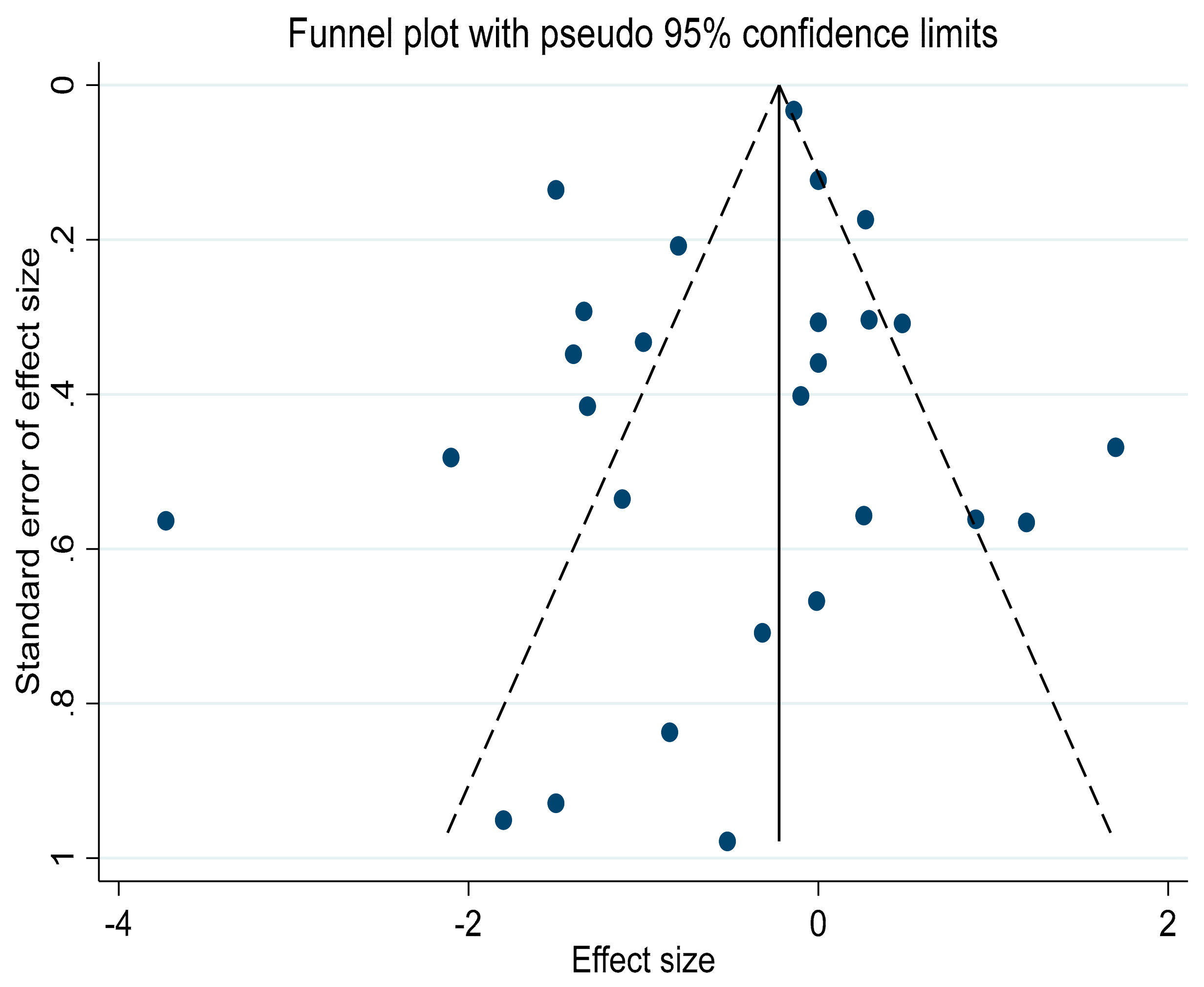

J) TG

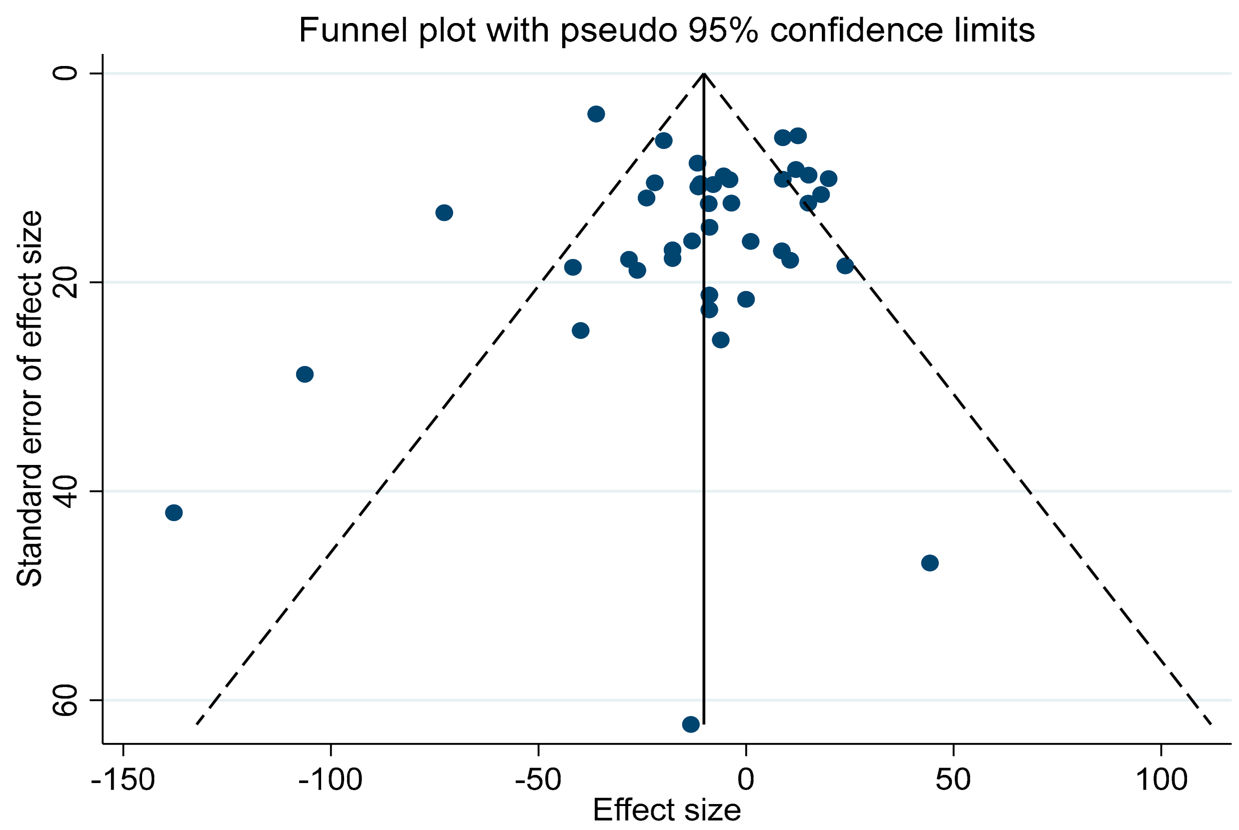

Figure S1. *Cont.*

K) TC

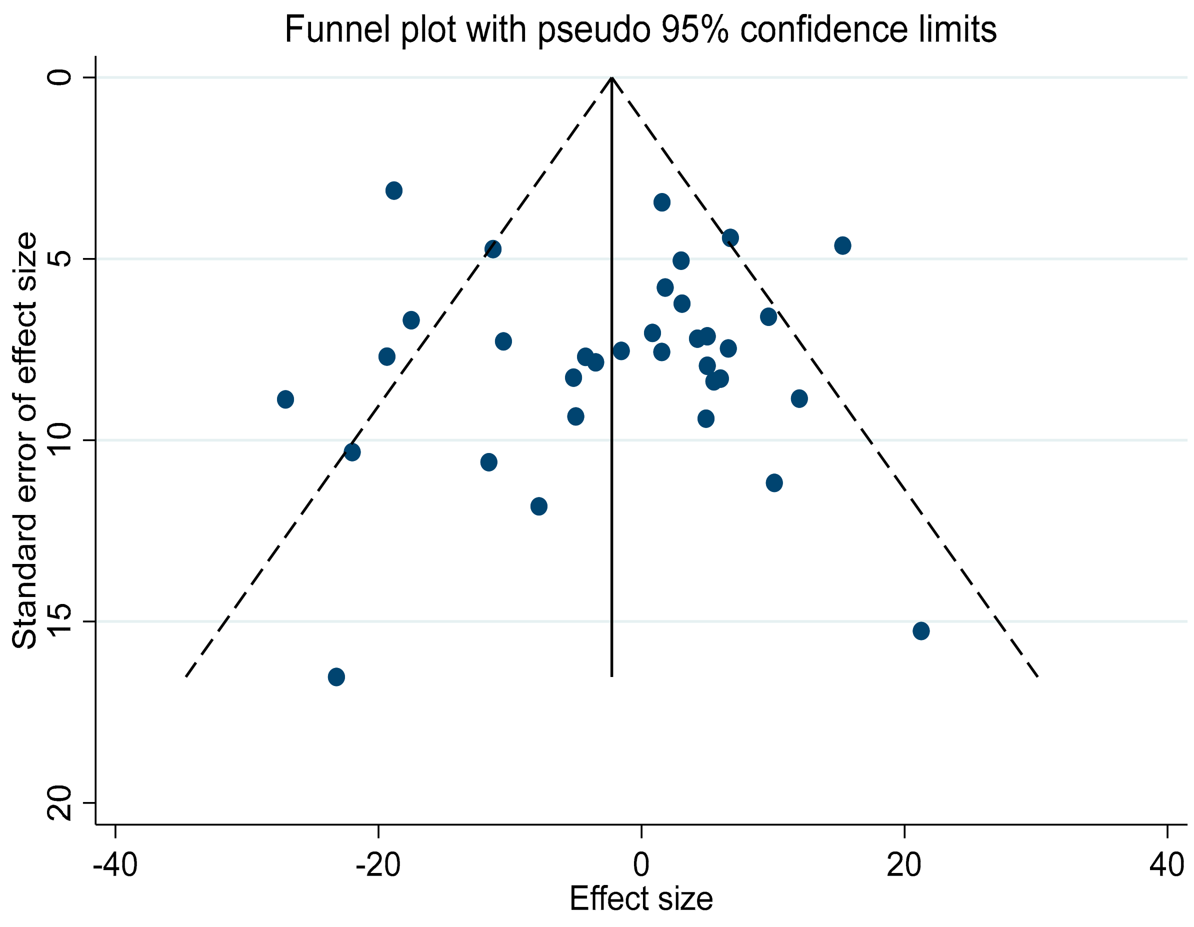

L) LDL-C

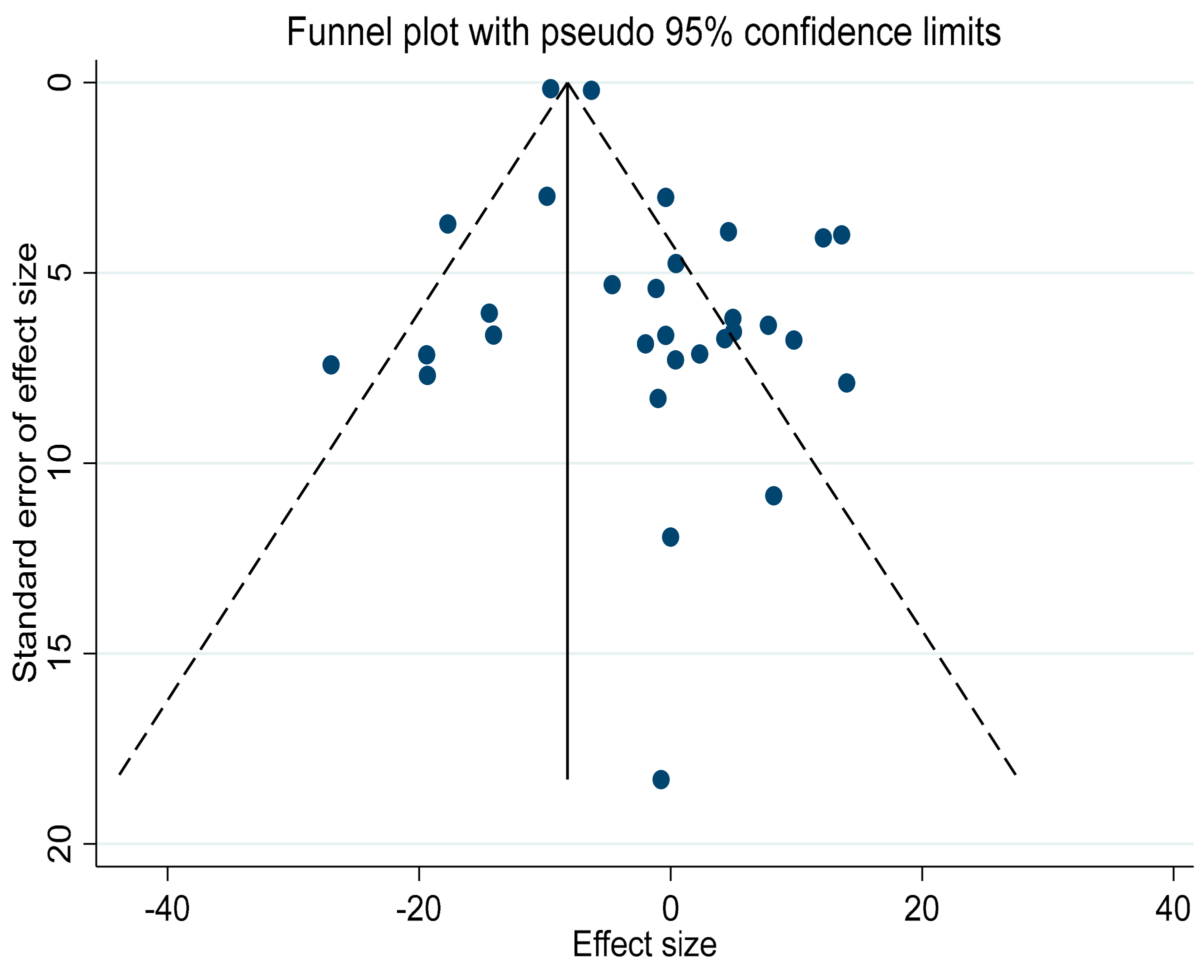

Figure S1. *Cont.*

M) HDL-C

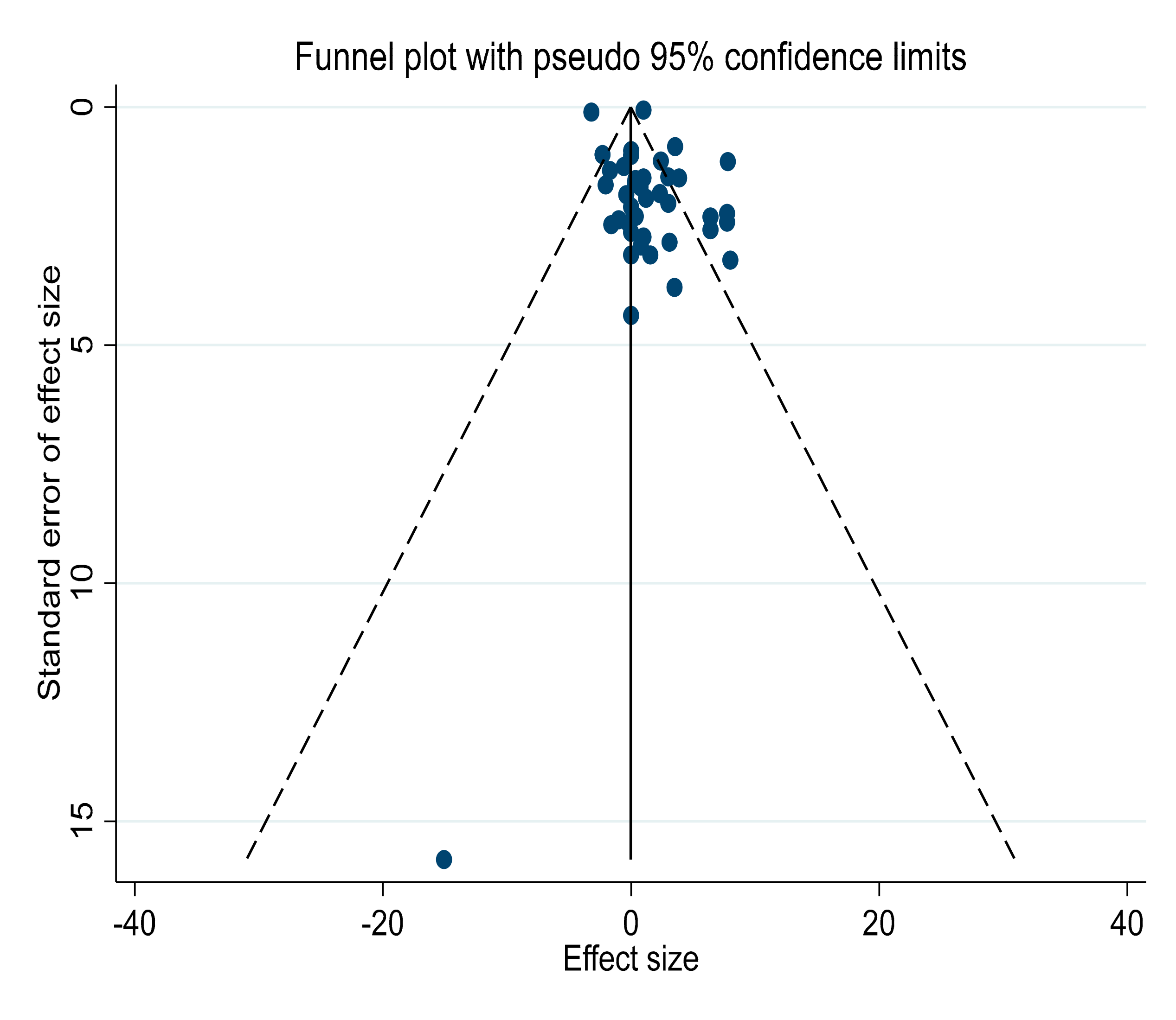

N) SBP

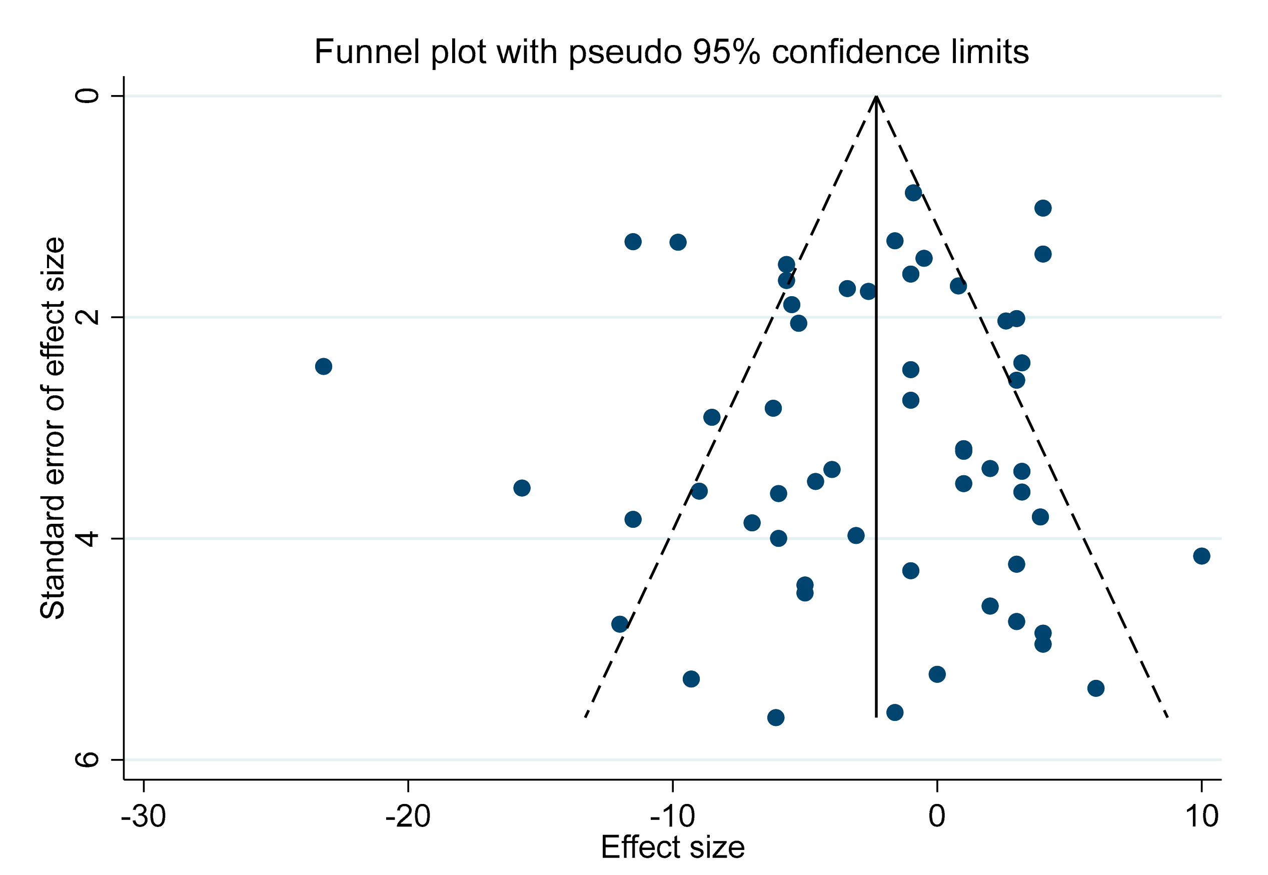

Figure S1. *Cont.*

O) DBP

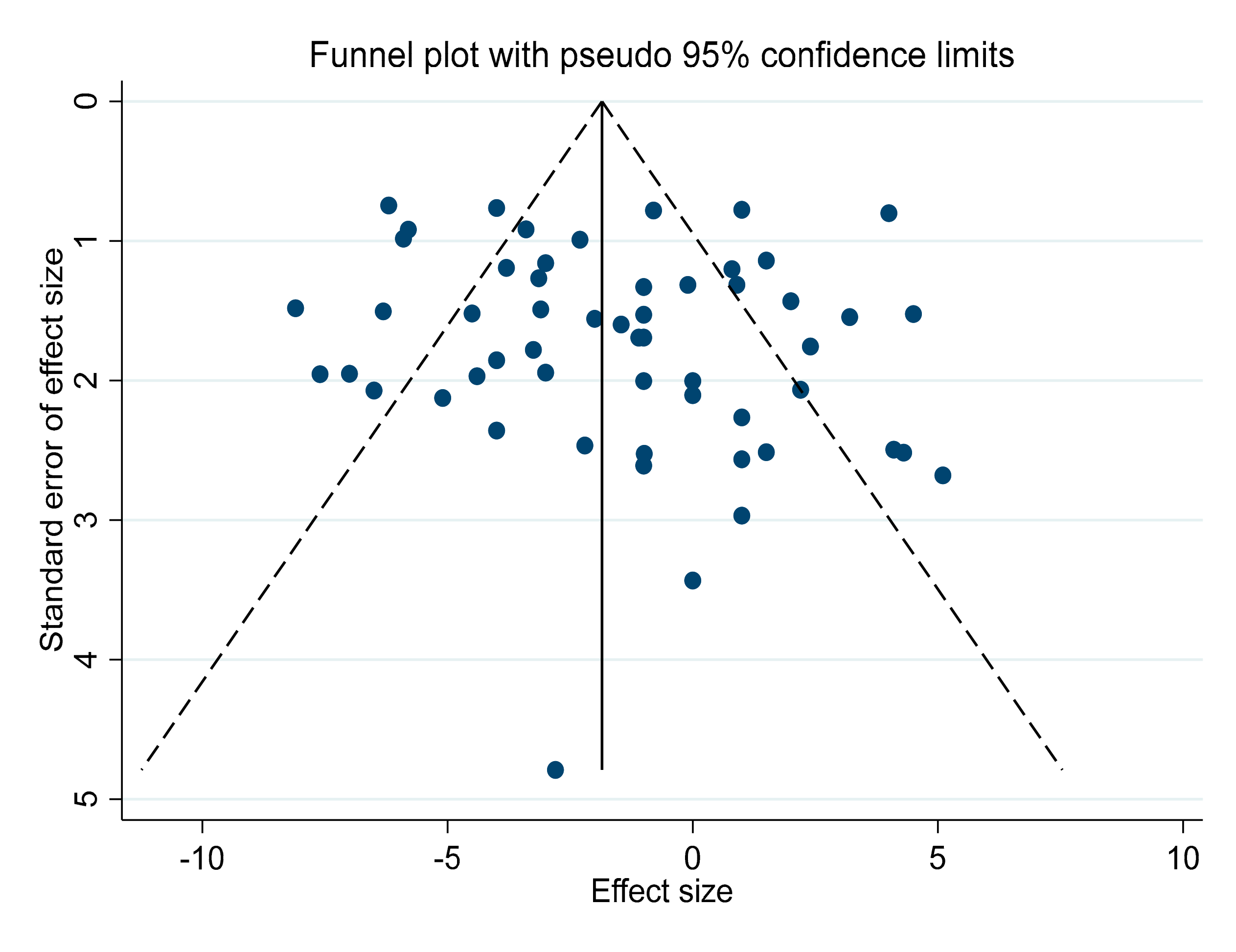

P) CRP

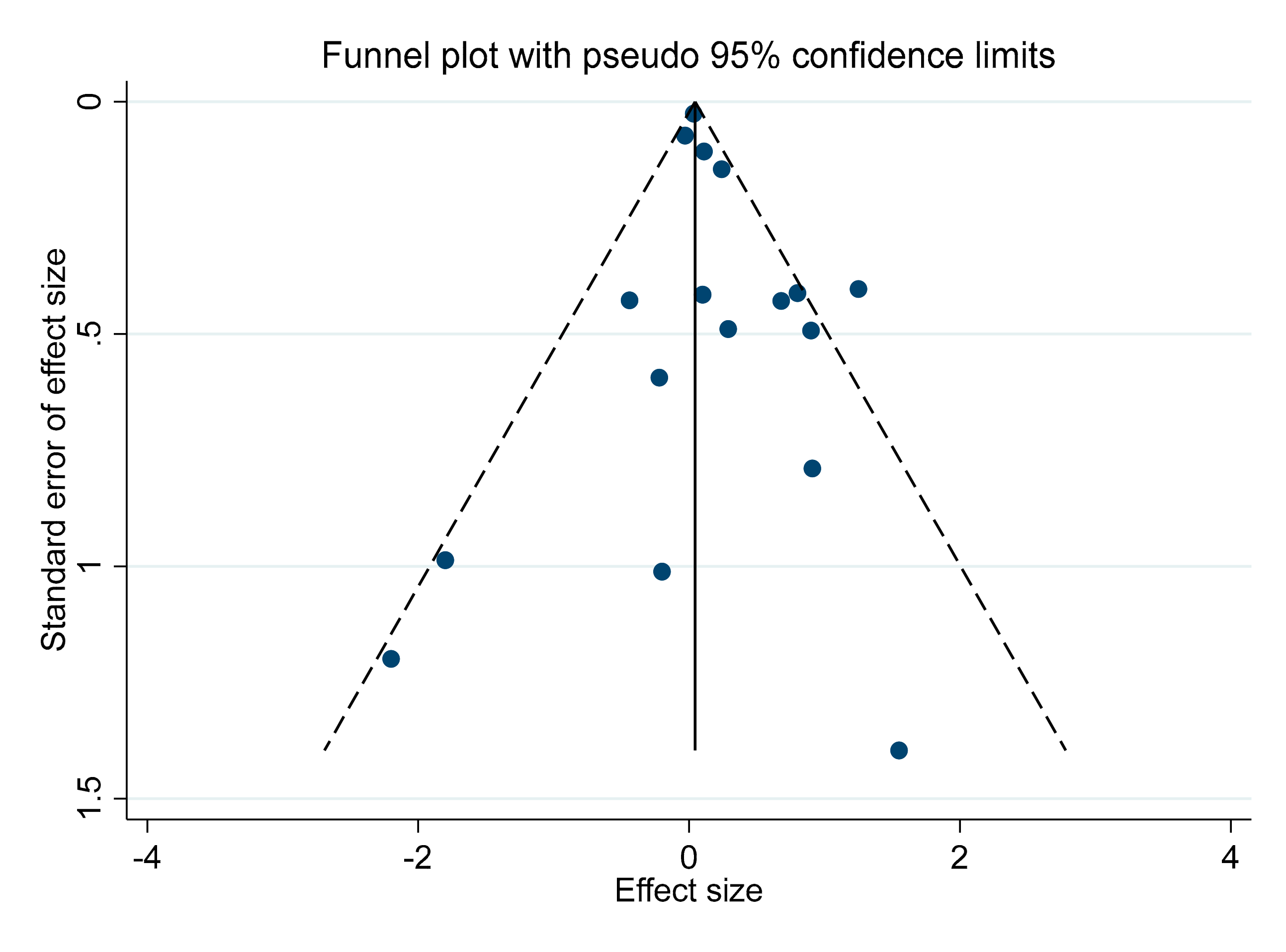

Figure S1. *Cont.*

Q) IL-6

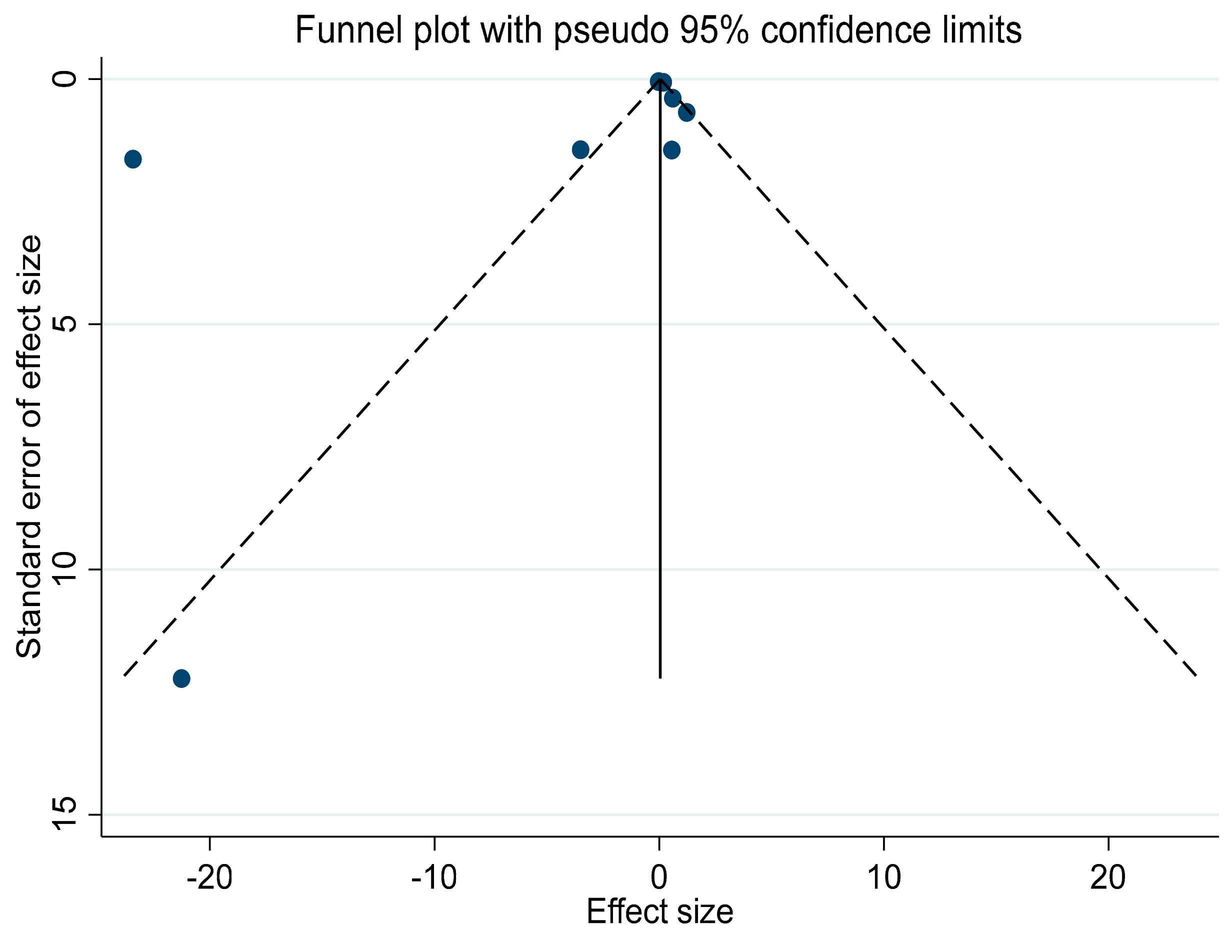

R) TNF-α

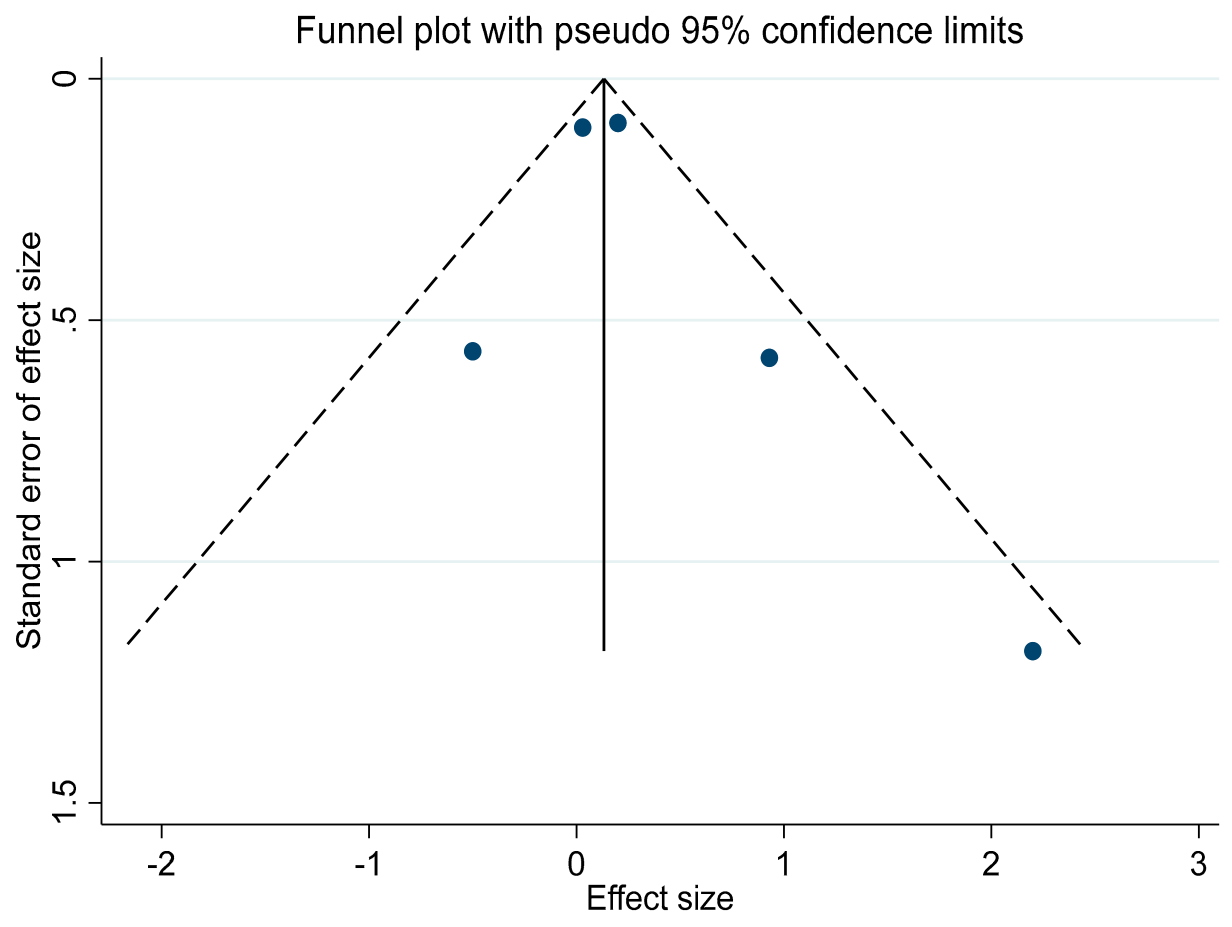

Figure S1. *Cont.*

S) AST

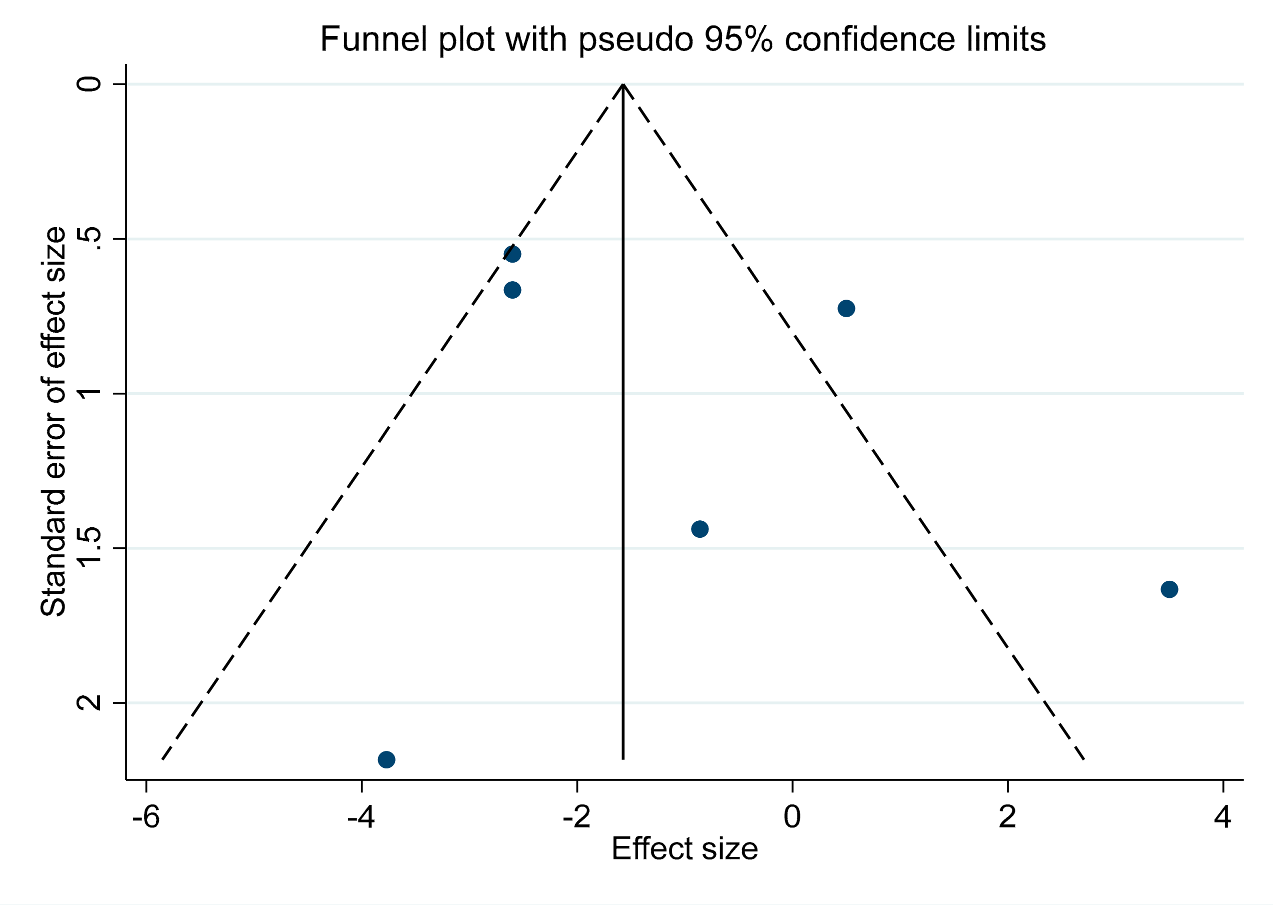

T) ALT

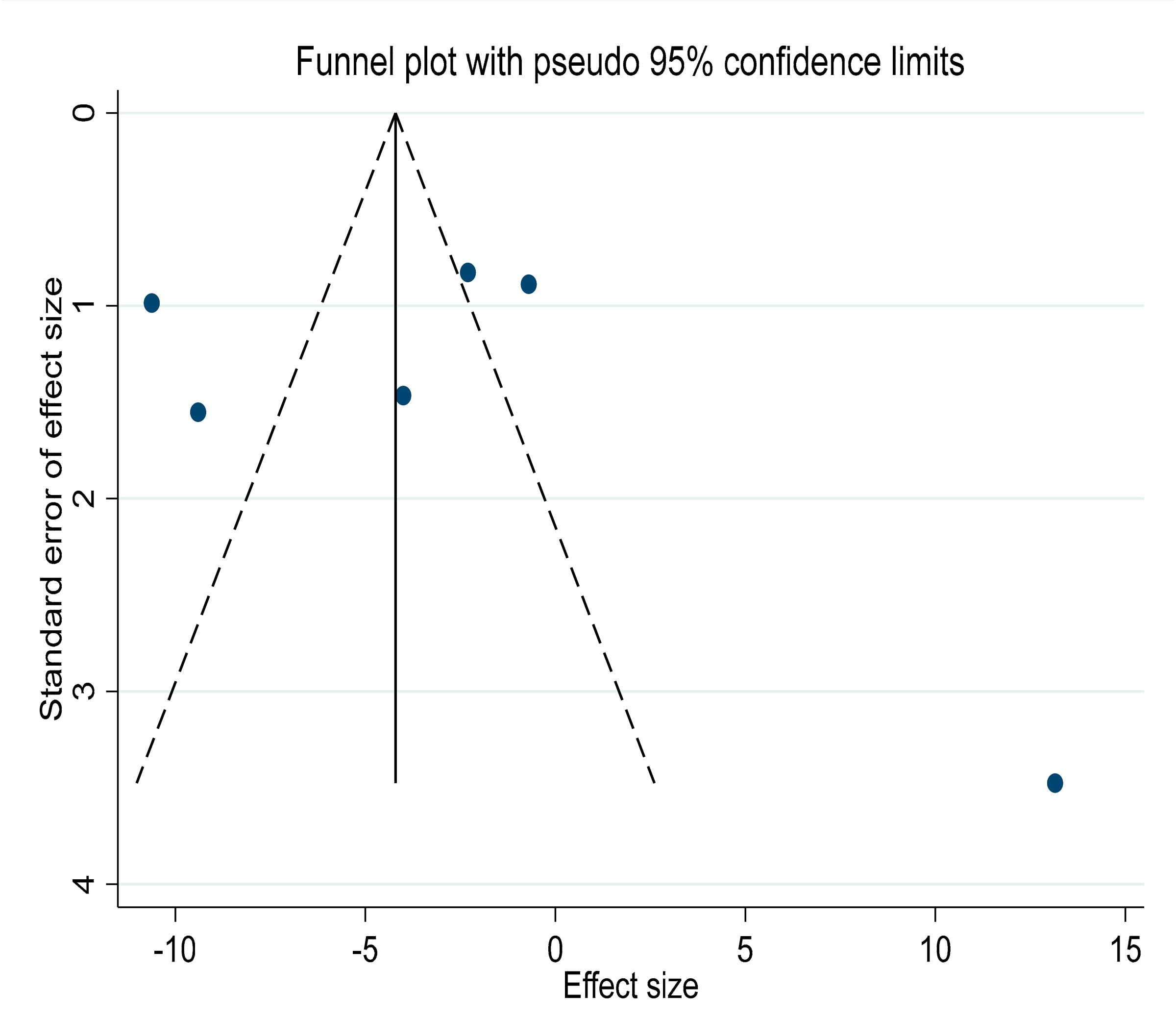

Figure S1. *Cont.*

U) Cr

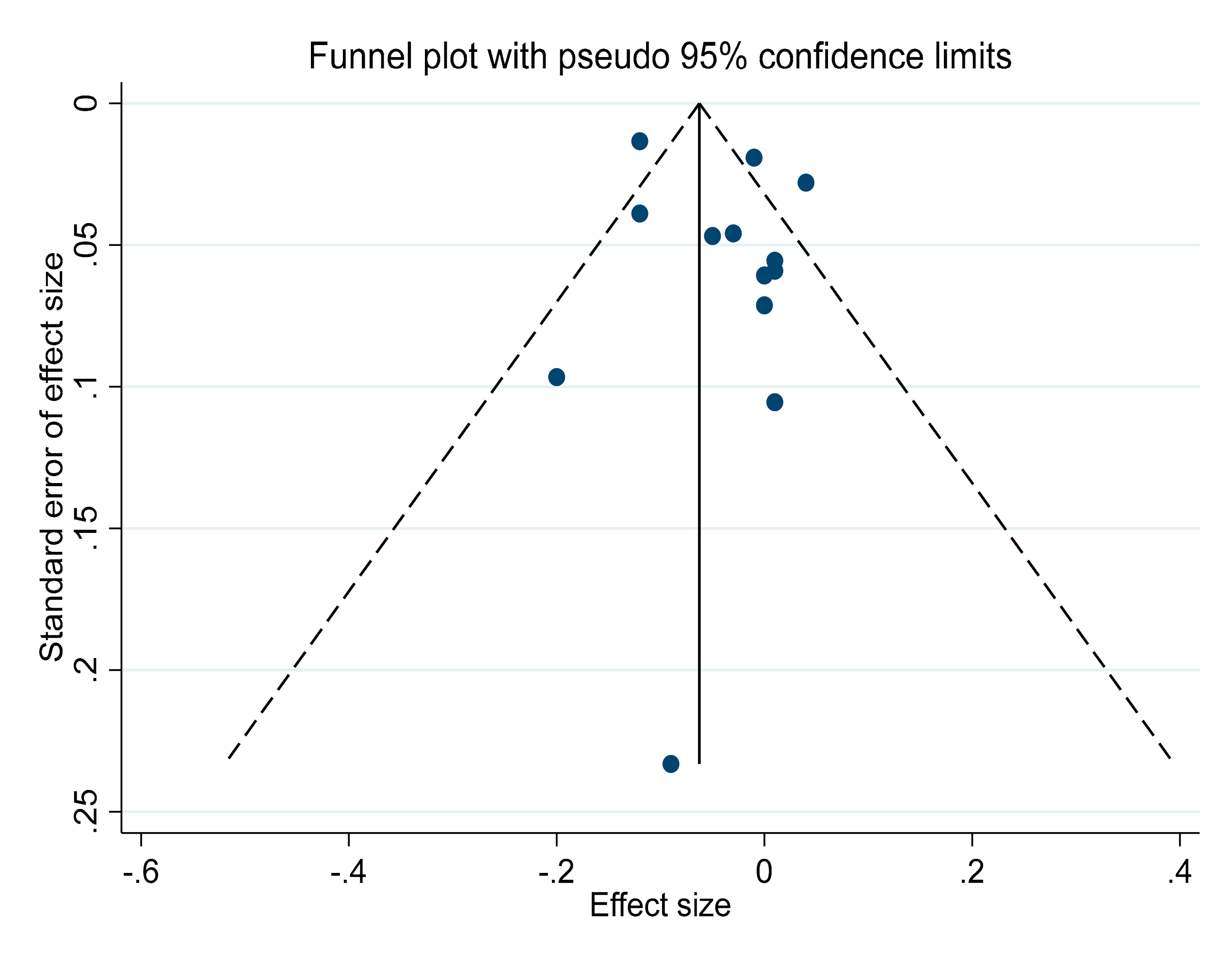

Figure S1. Funnel plots for the impacts of Mg supplementation on cardiometabolic risk factors (CMRFs), including **(A)** BW, **(B)** BMI, **(C)** WC, **(D)** HC, **(E)** BFP, **(F)** FBG, **(G)** HbA1c, **(H)** FI, **(I)** HOMA-IR, **(J)** TG, **(K)** TC, **(L)** LDL-C, **(M)** HDL-C, **(N)** SBP, **(O)** DBP, **(P)** CRP, **(Q)** IL-6, **(R)** TNF-α, **(S)** AST, **(T)**ALT, and **(U)** Cr.

A) BW

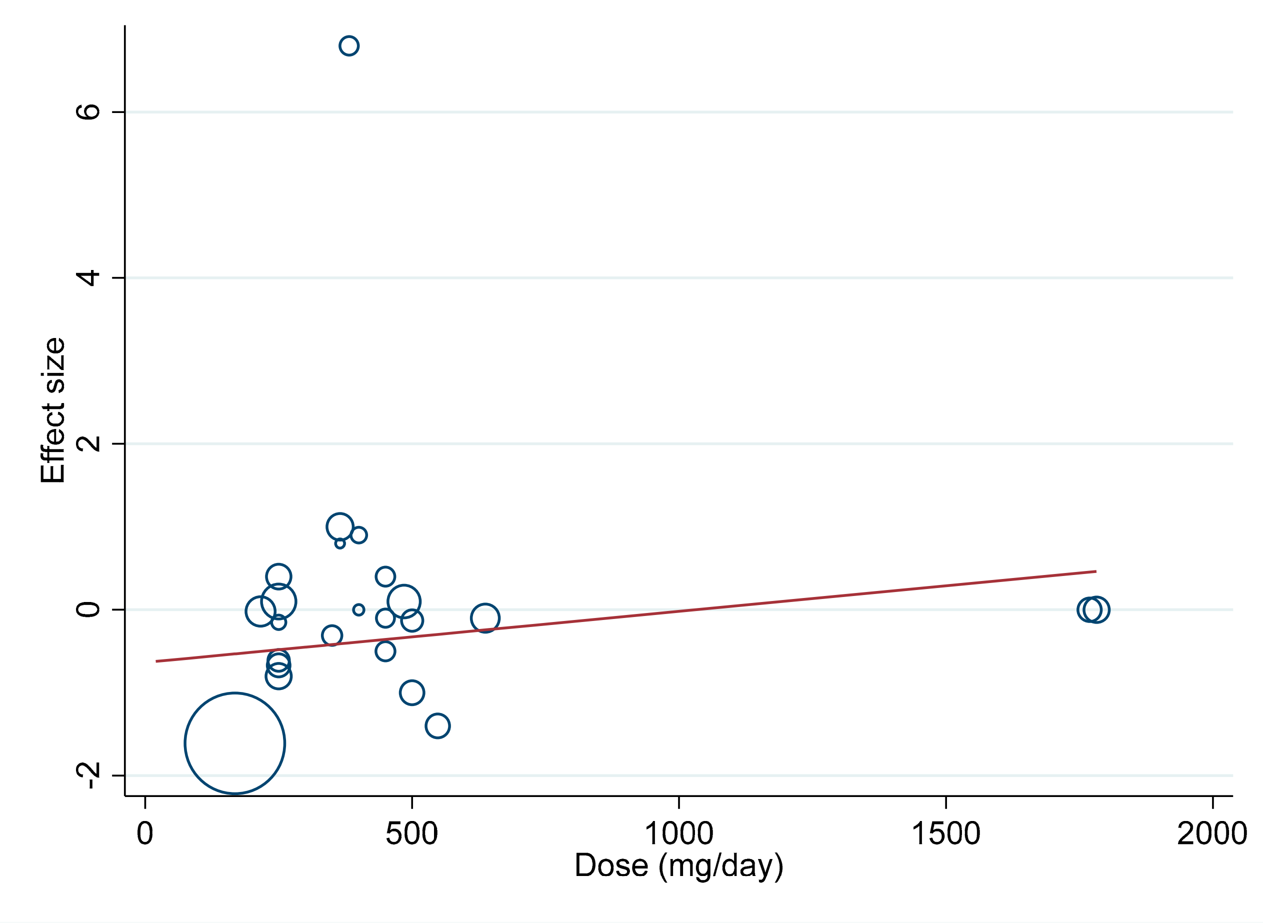

B) BMI

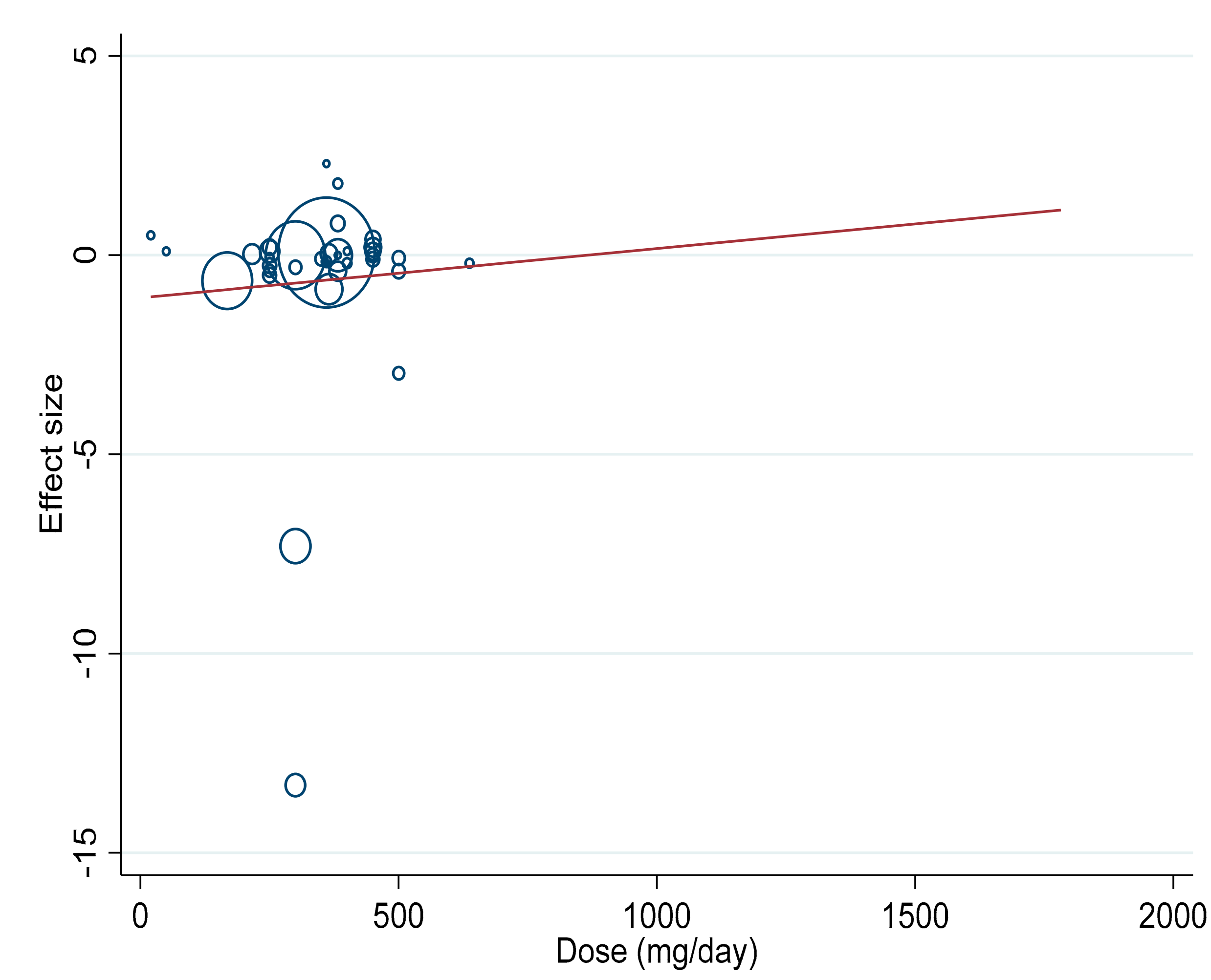

Figure S2. *Cont.*

C) WC

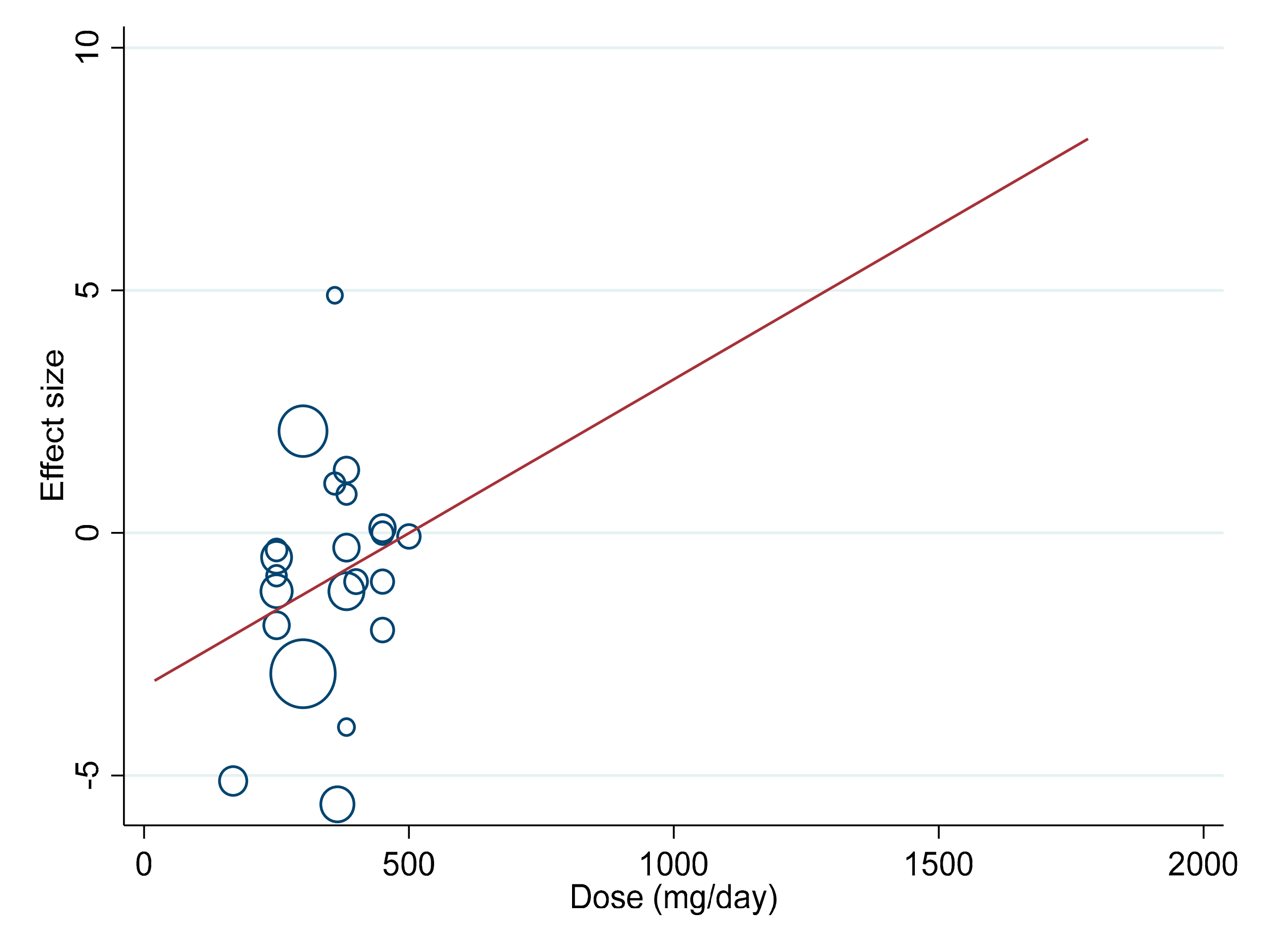

D) HC

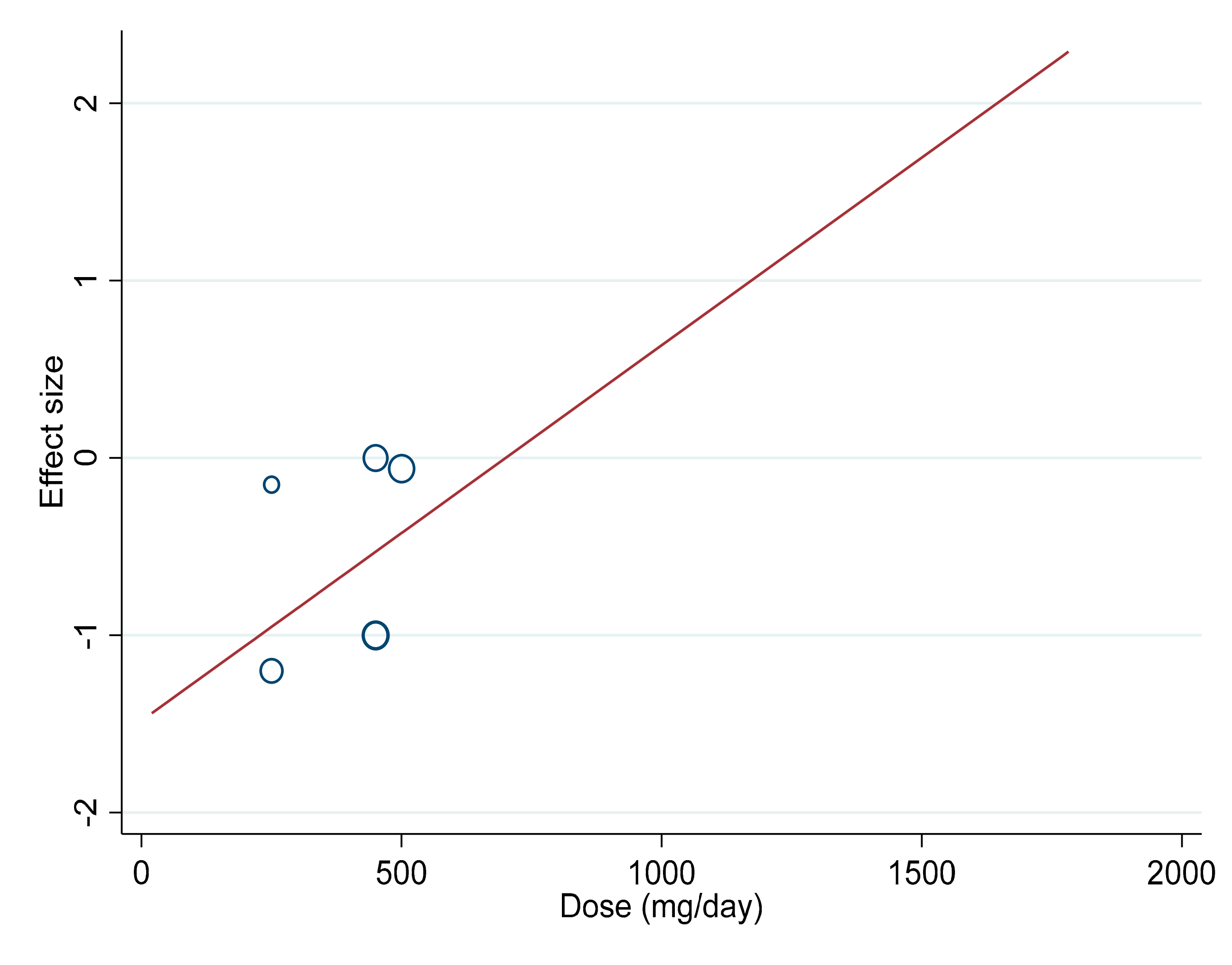

Figure S2. *Cont.*

E) BFP

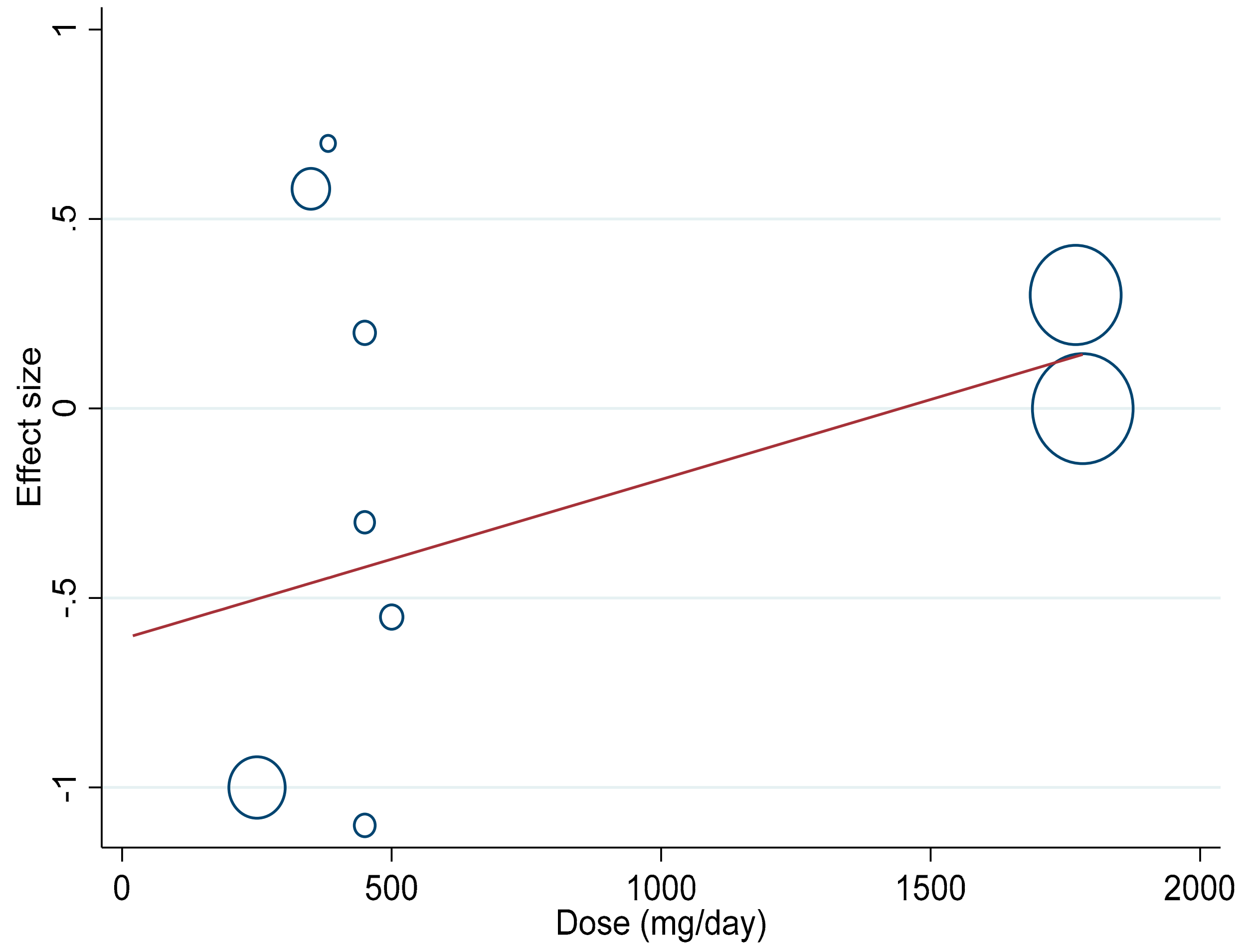

F) FBG

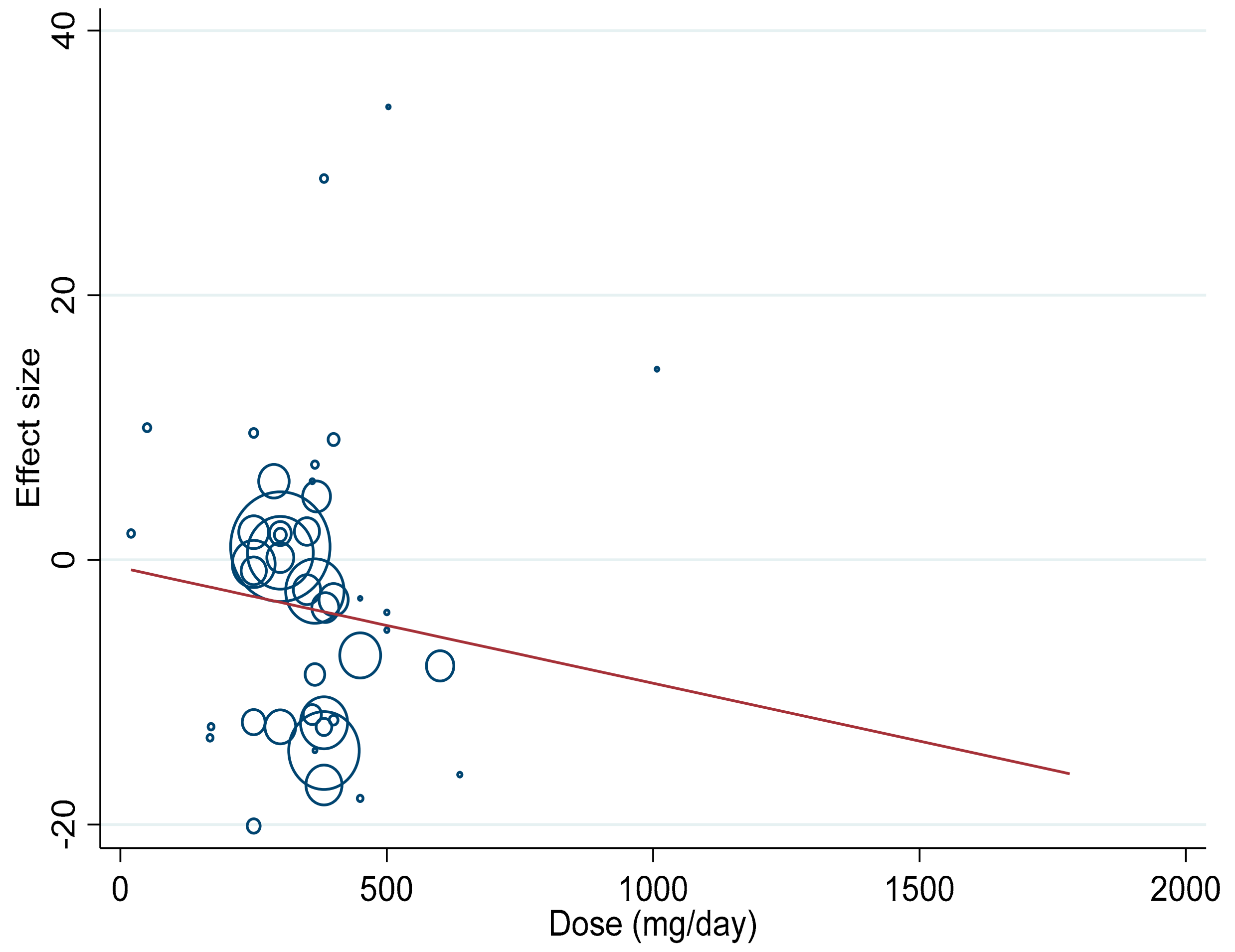

Figure S2. *Cont.*

G) HbA1c

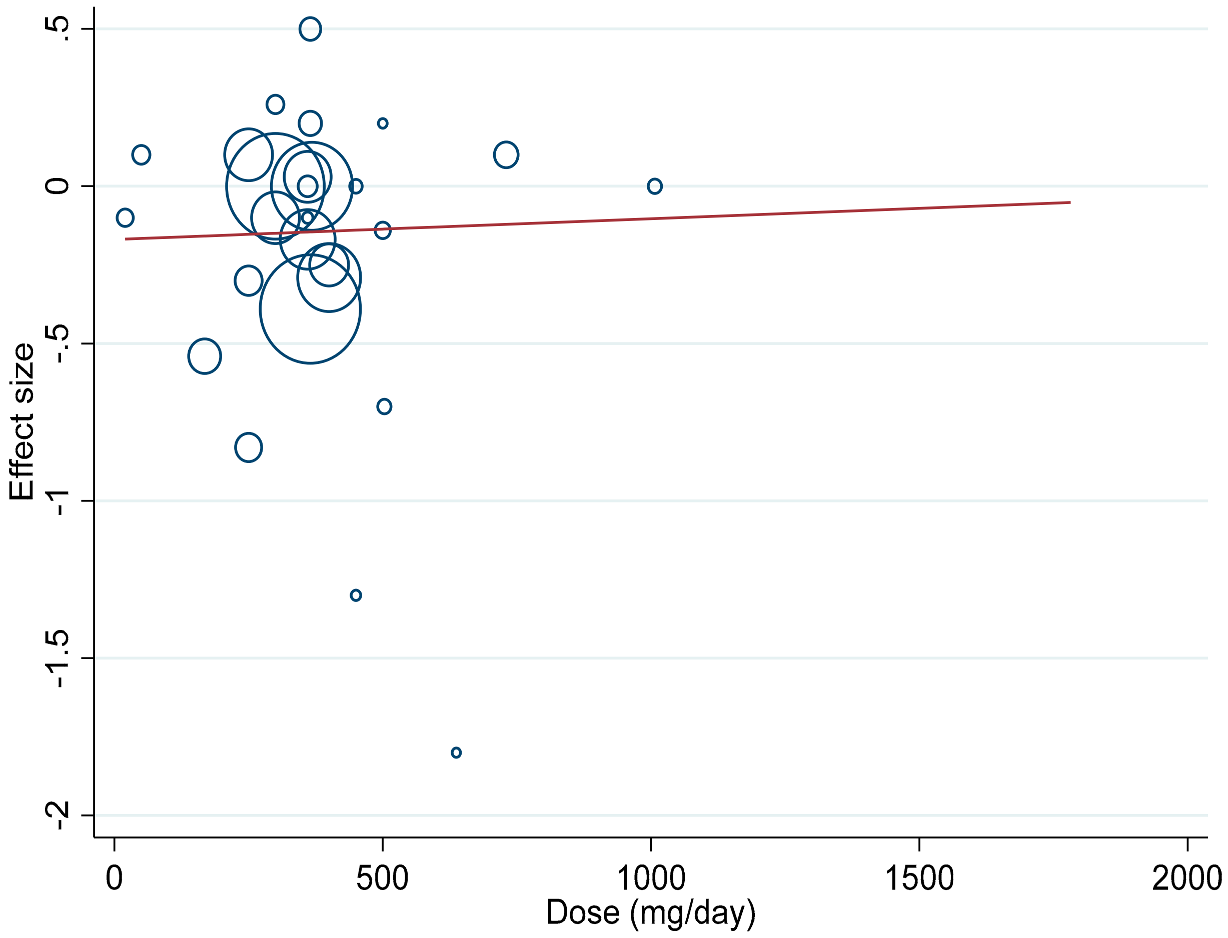

H) FI

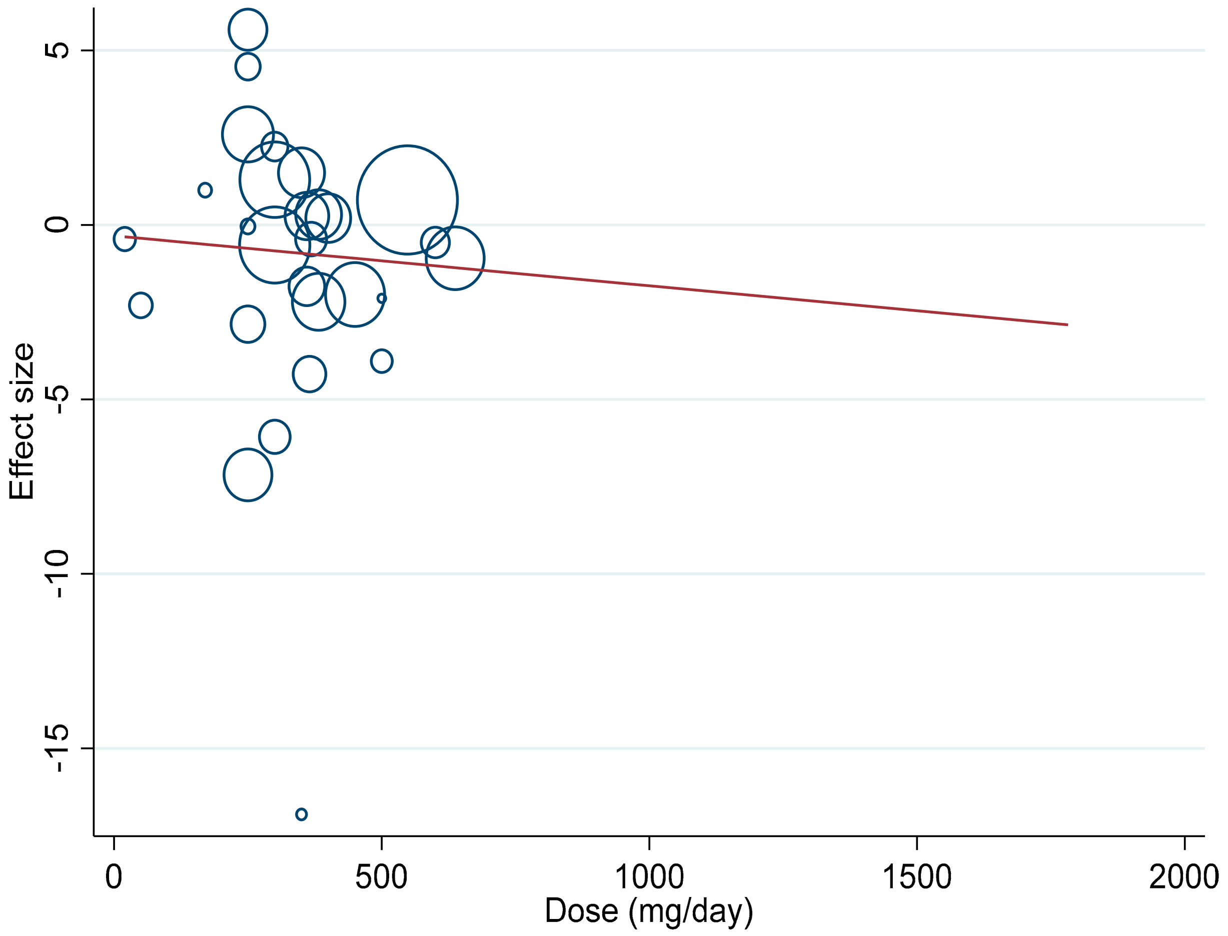

Figure S2. *Cont.*

I) HOMA-IR

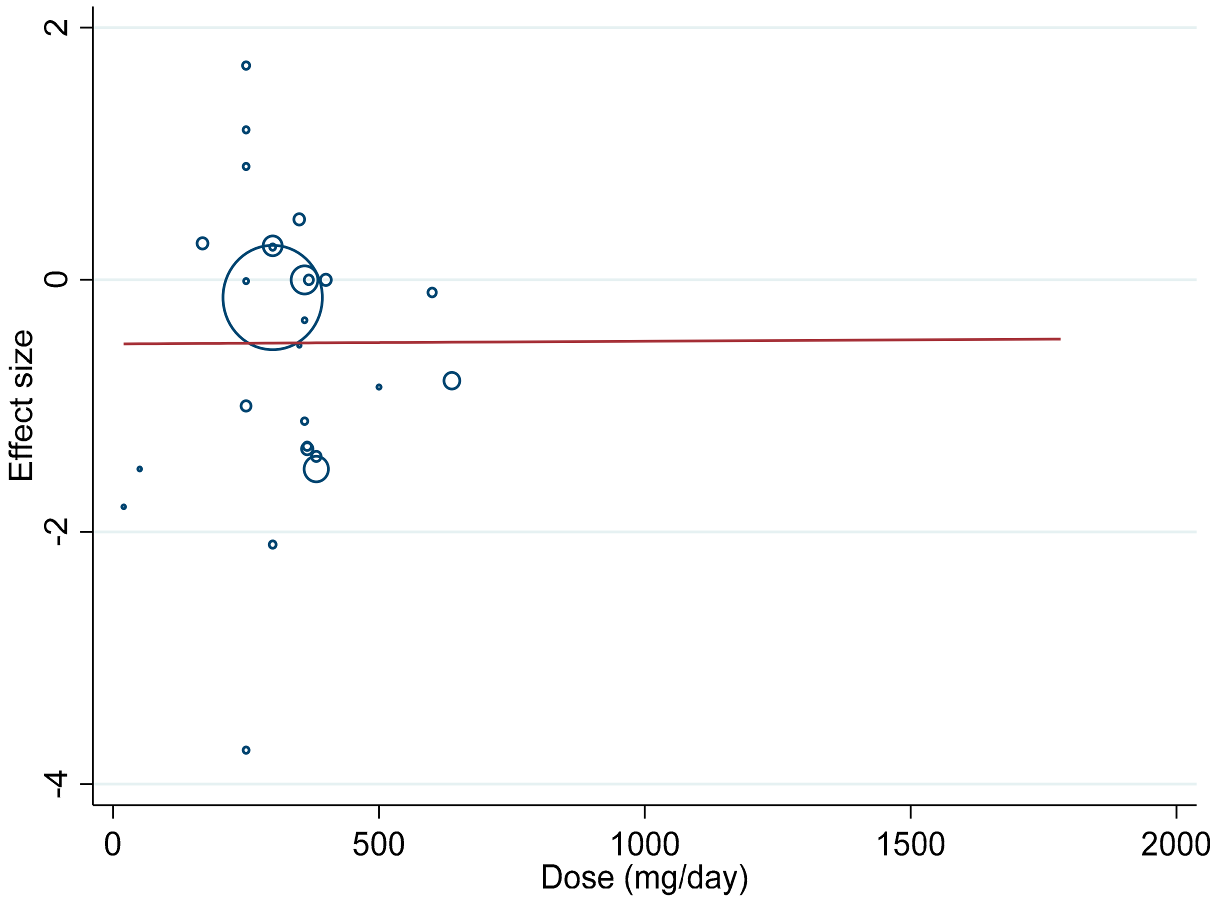

J) TG

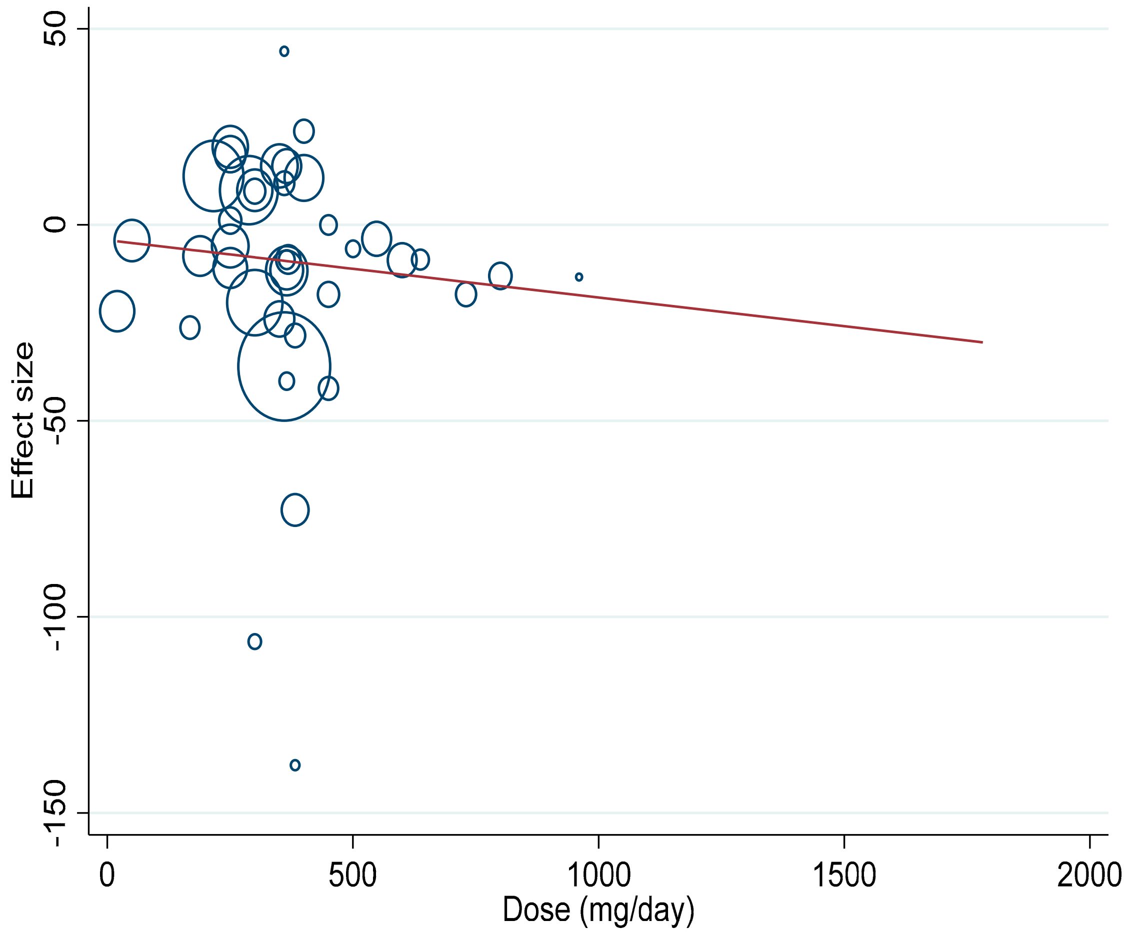

Figure S2. *Cont.*

K) TC

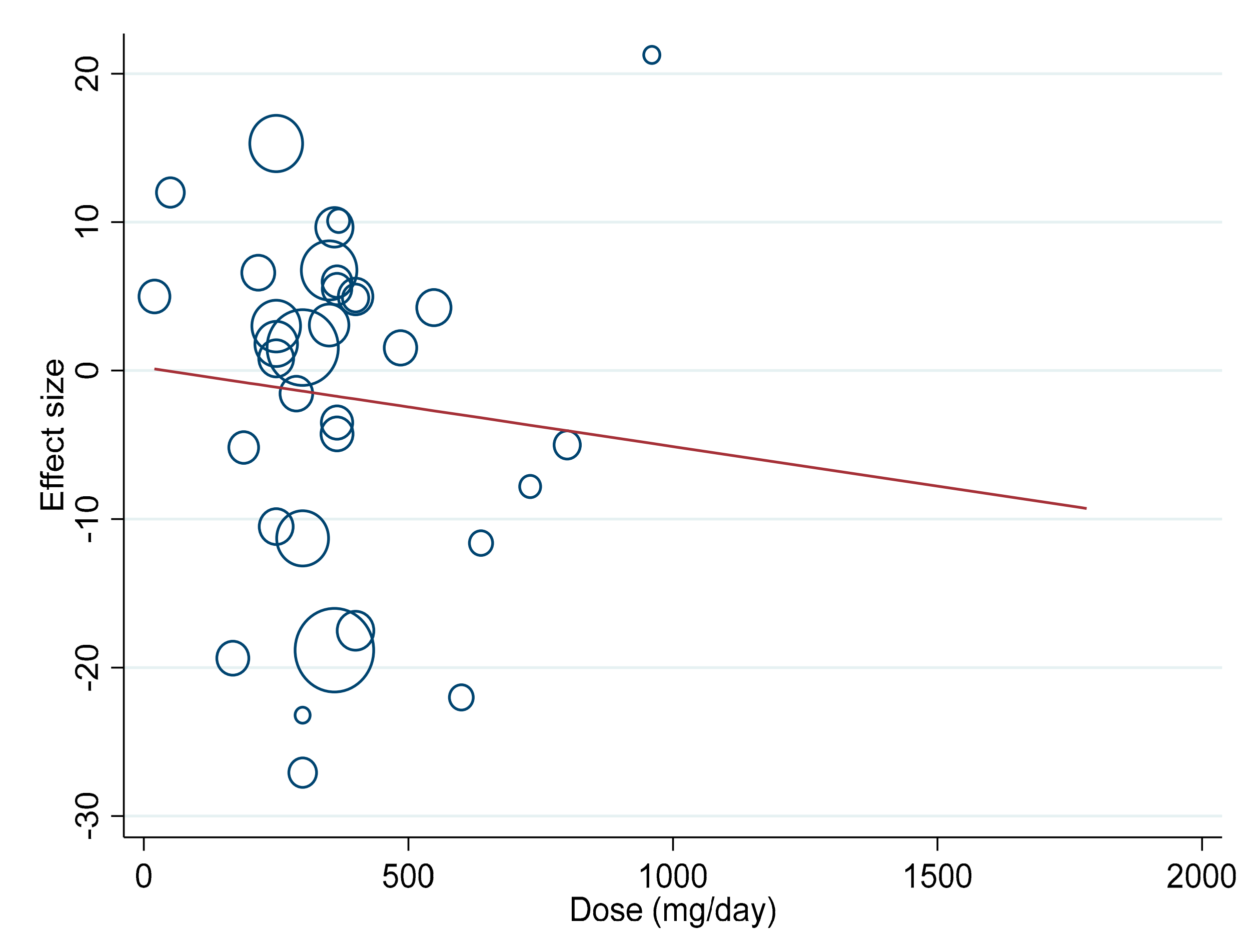

L) LDL-C

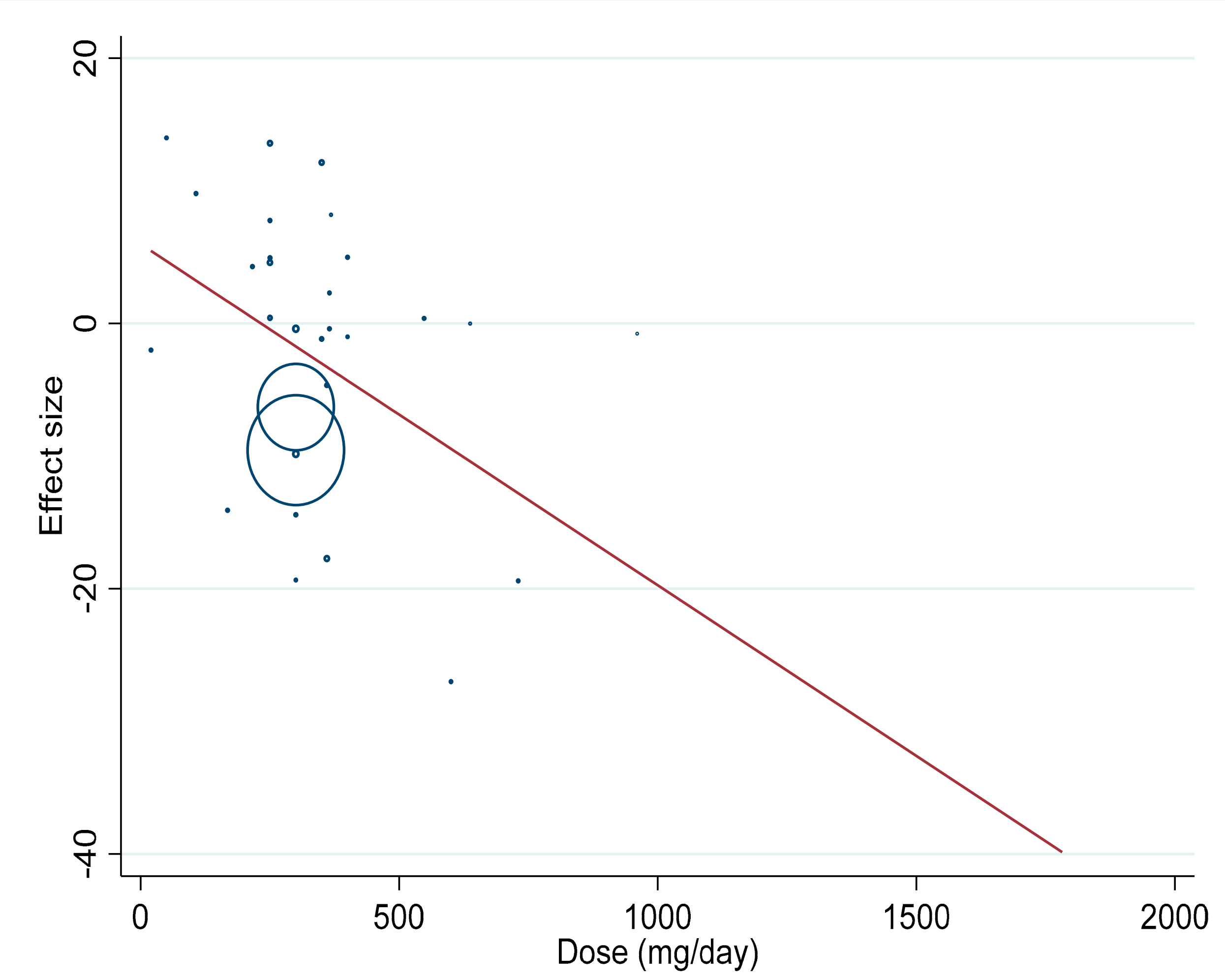

Figure S2. *Cont.*

M) HDL-C

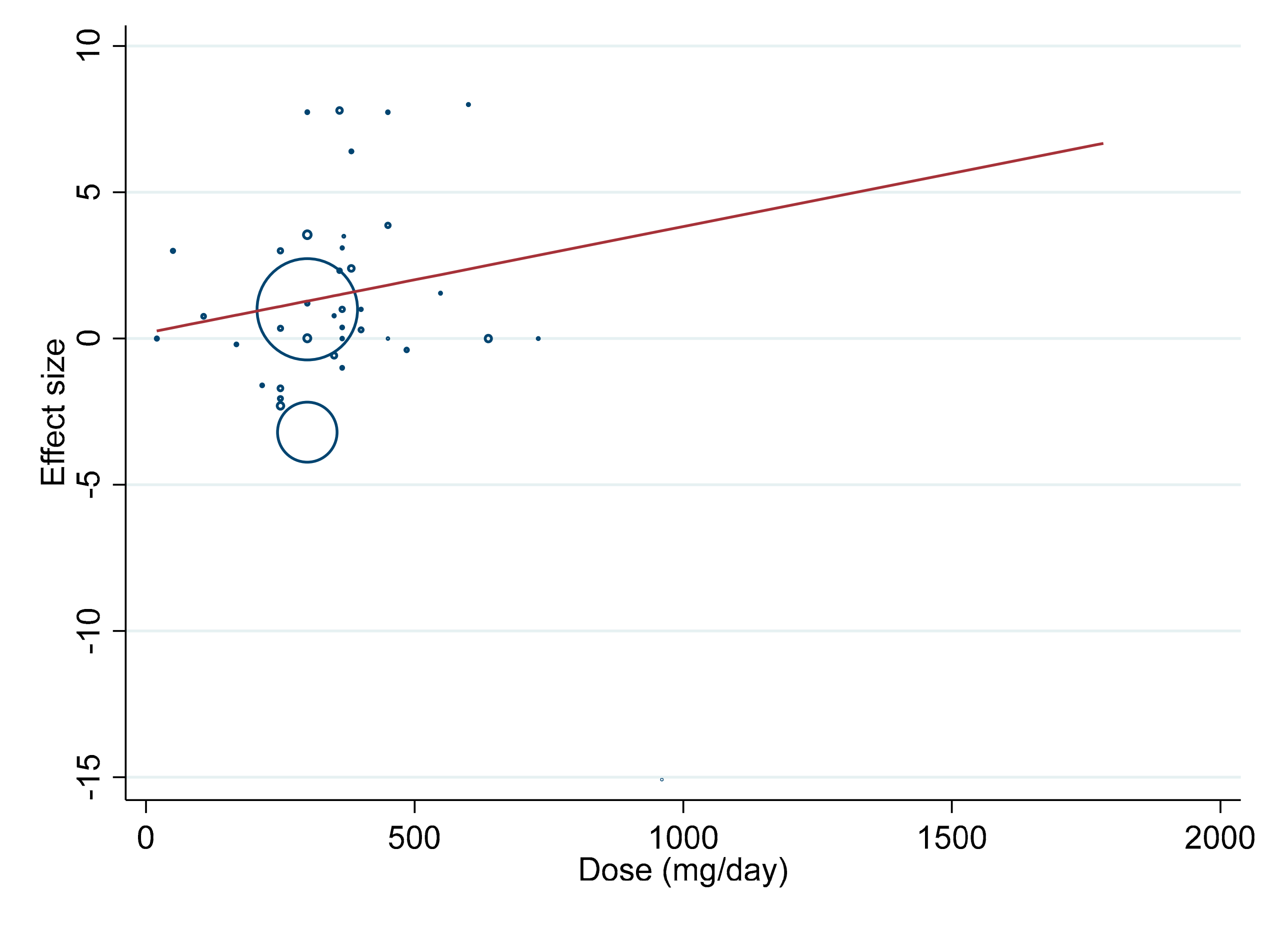

N) SBP

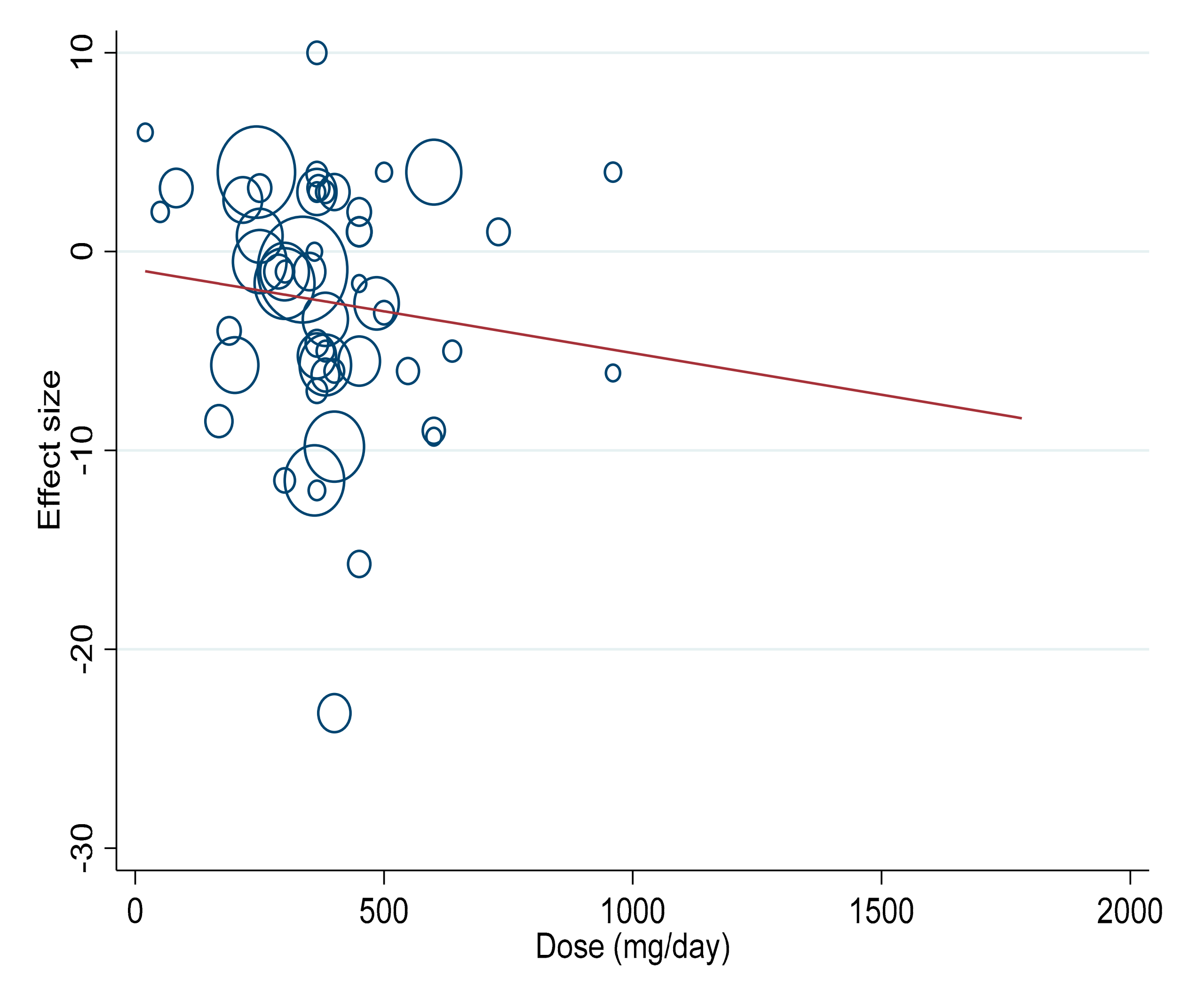

Figure S2. *Cont.*

O) DBP

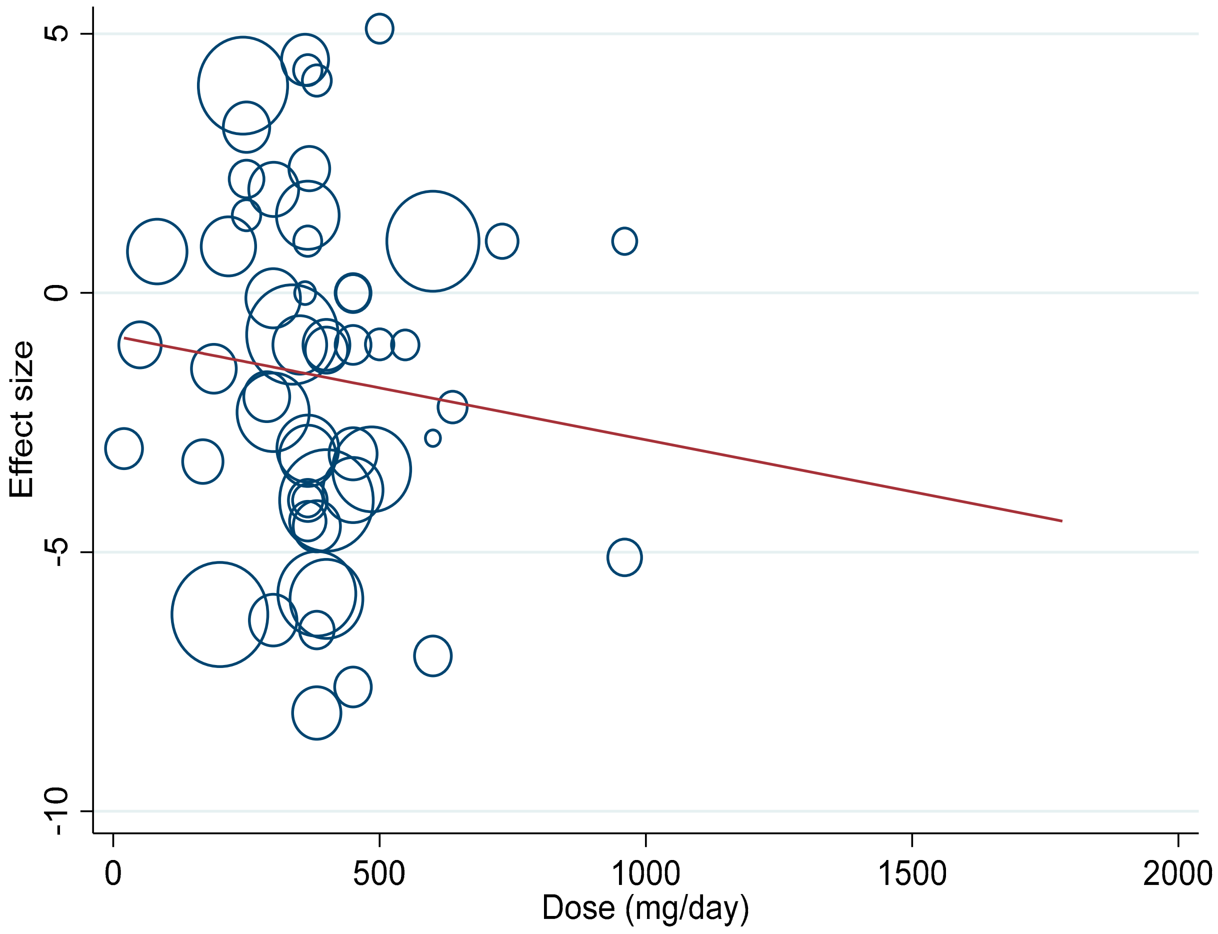

P) CRP

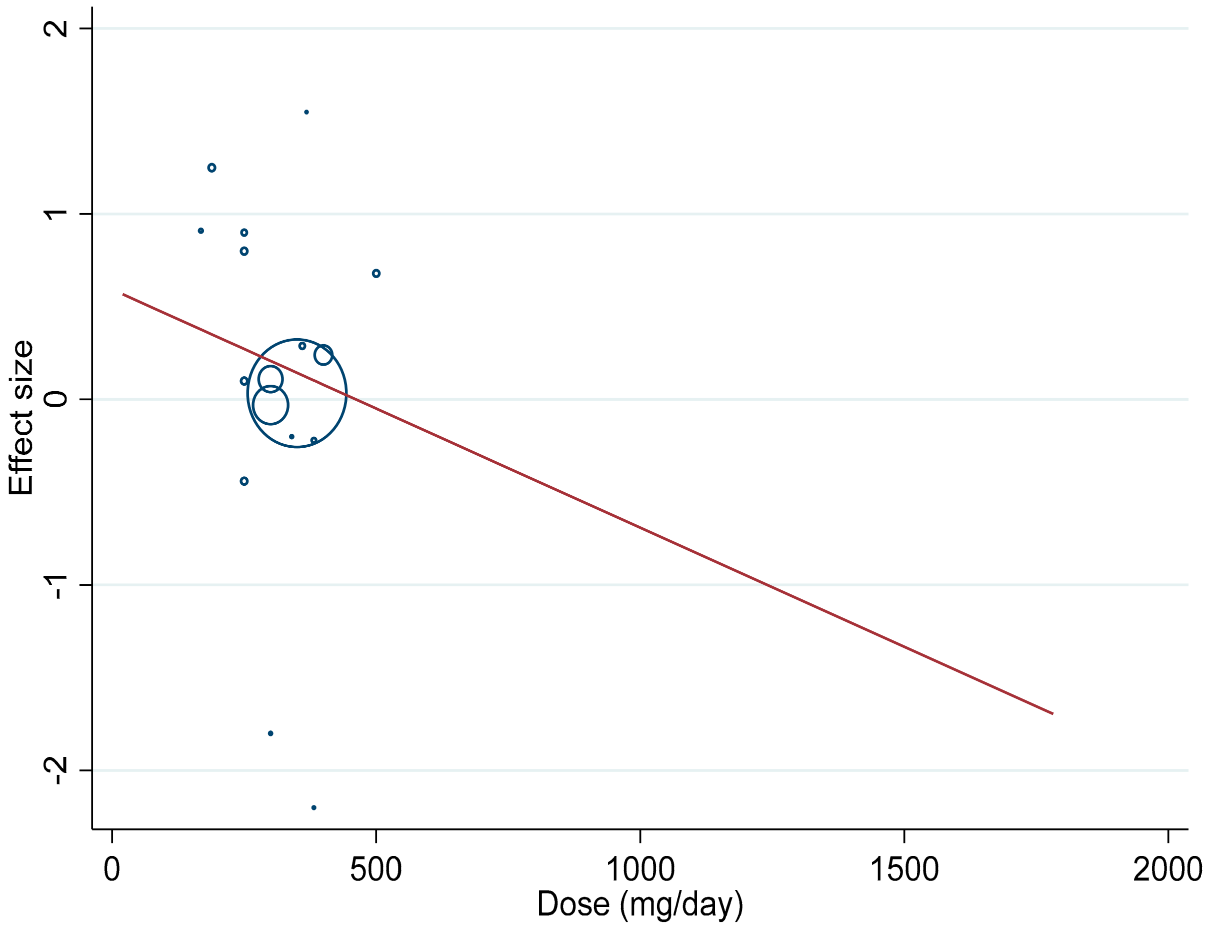

Figure S2. *Cont.*

Q) IL-6

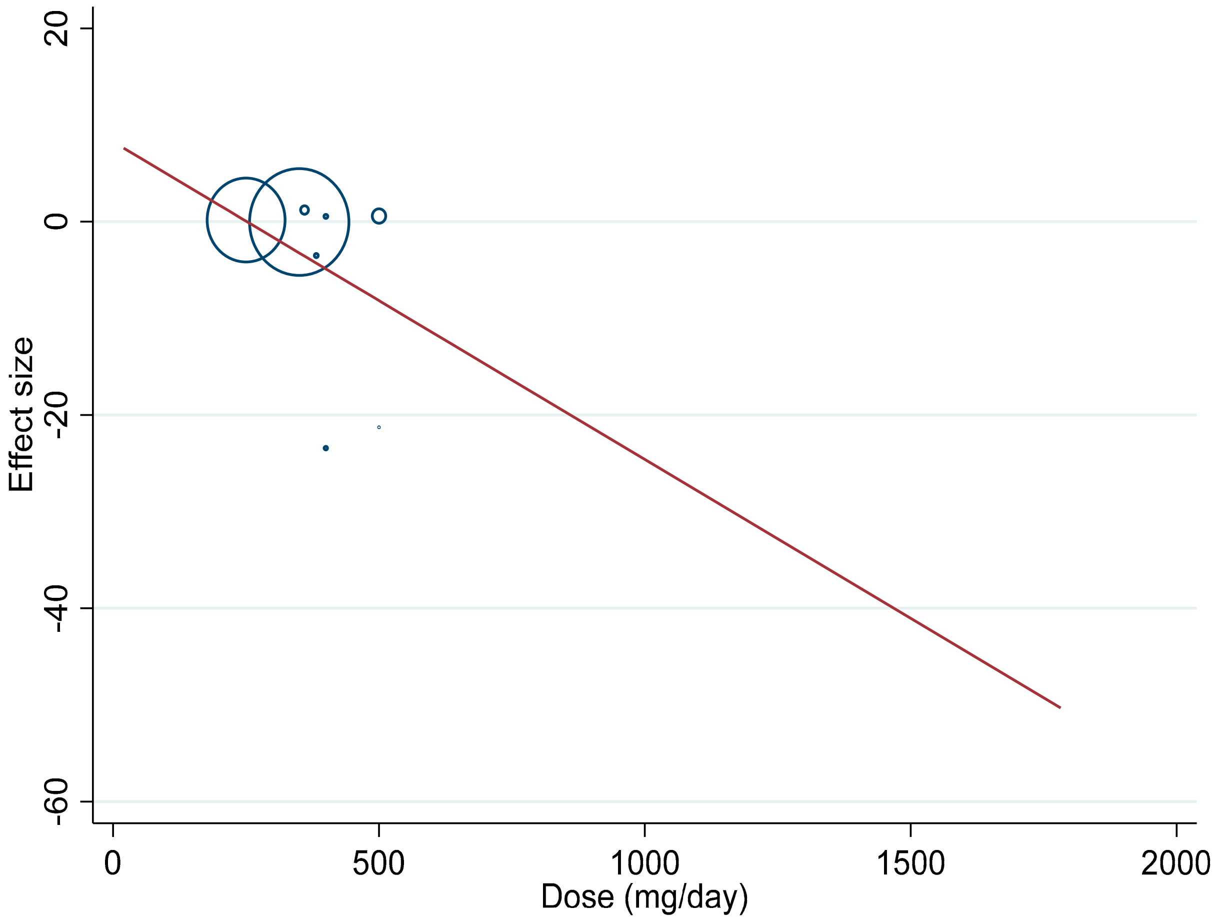

R) TNF-α

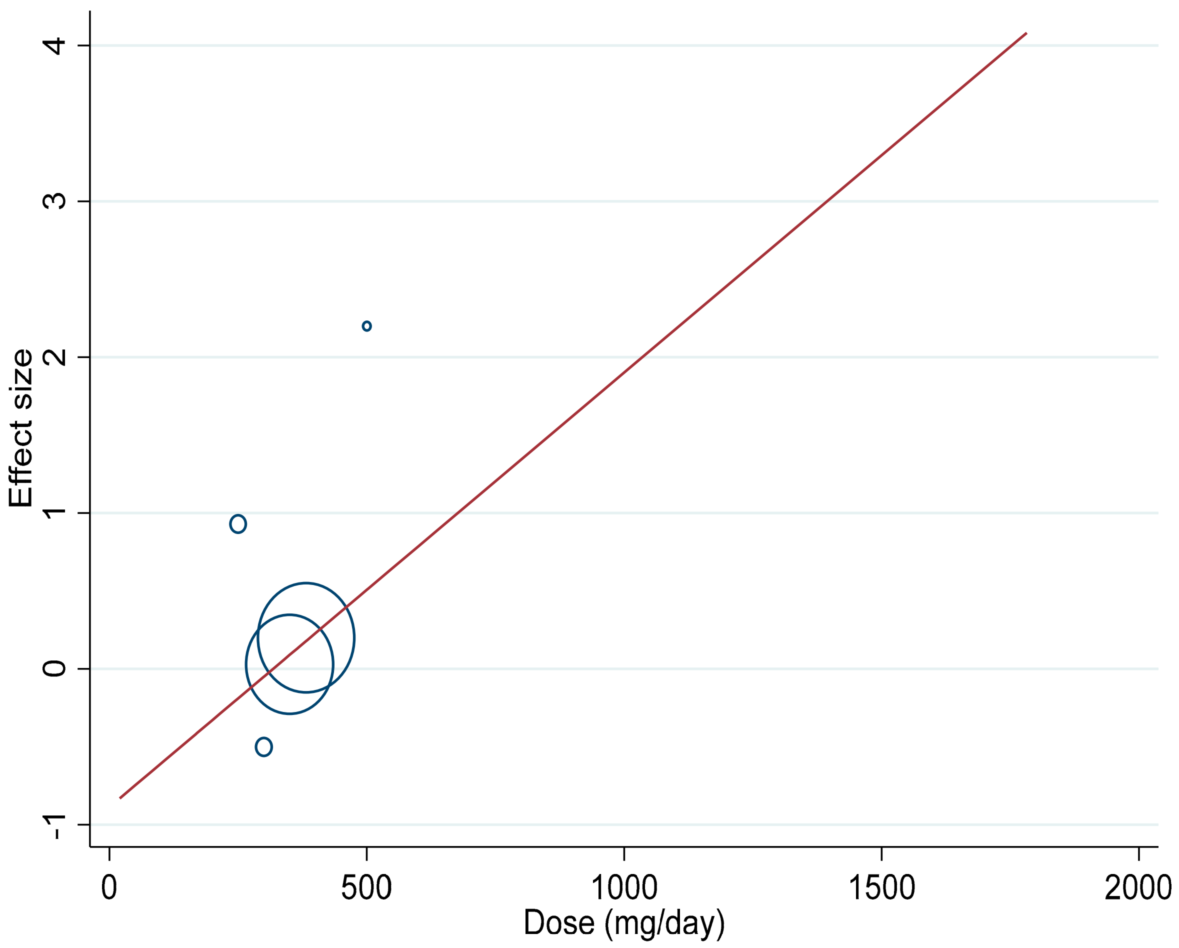

Figure S2. *Cont.*

S) AST

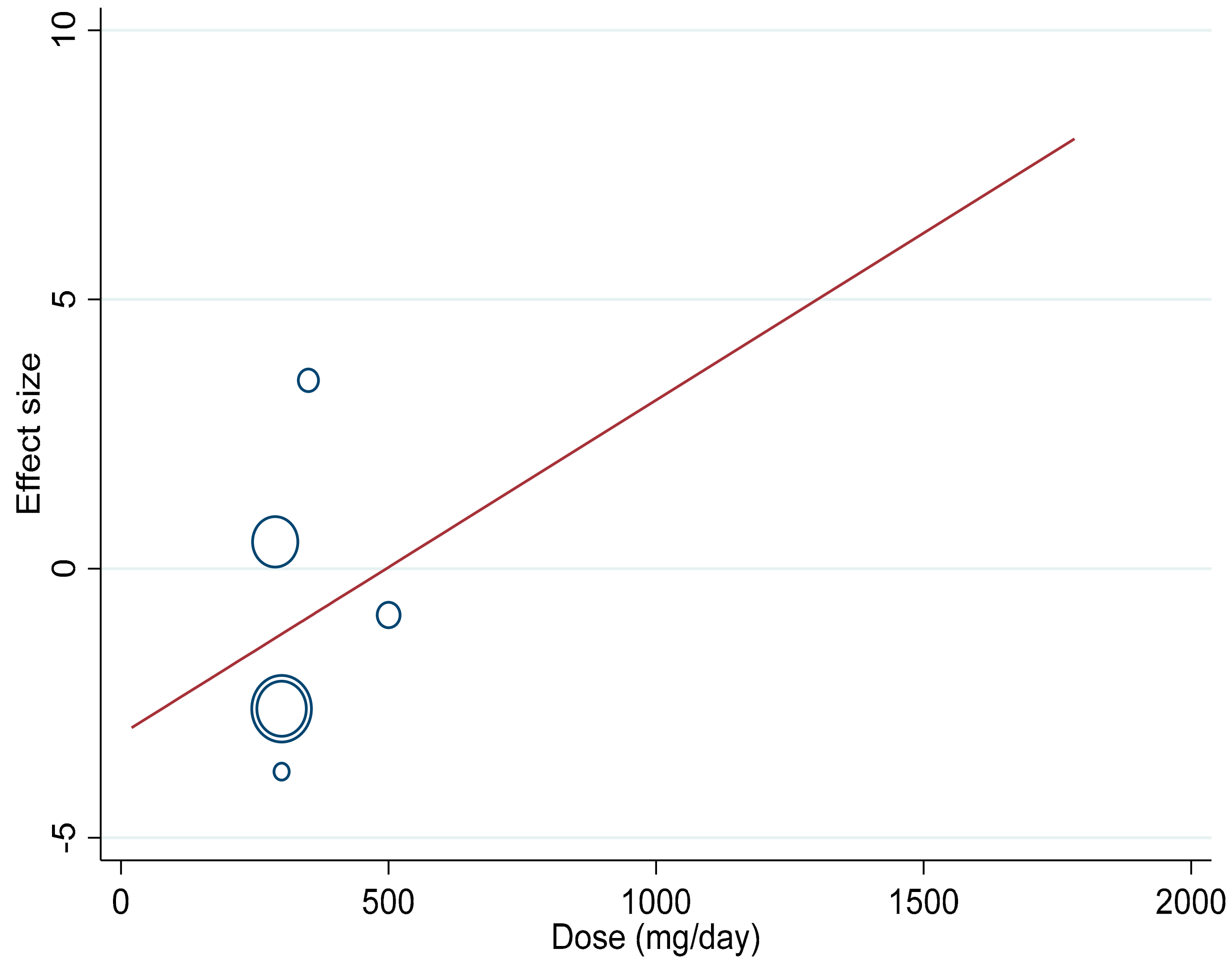

T) ALT

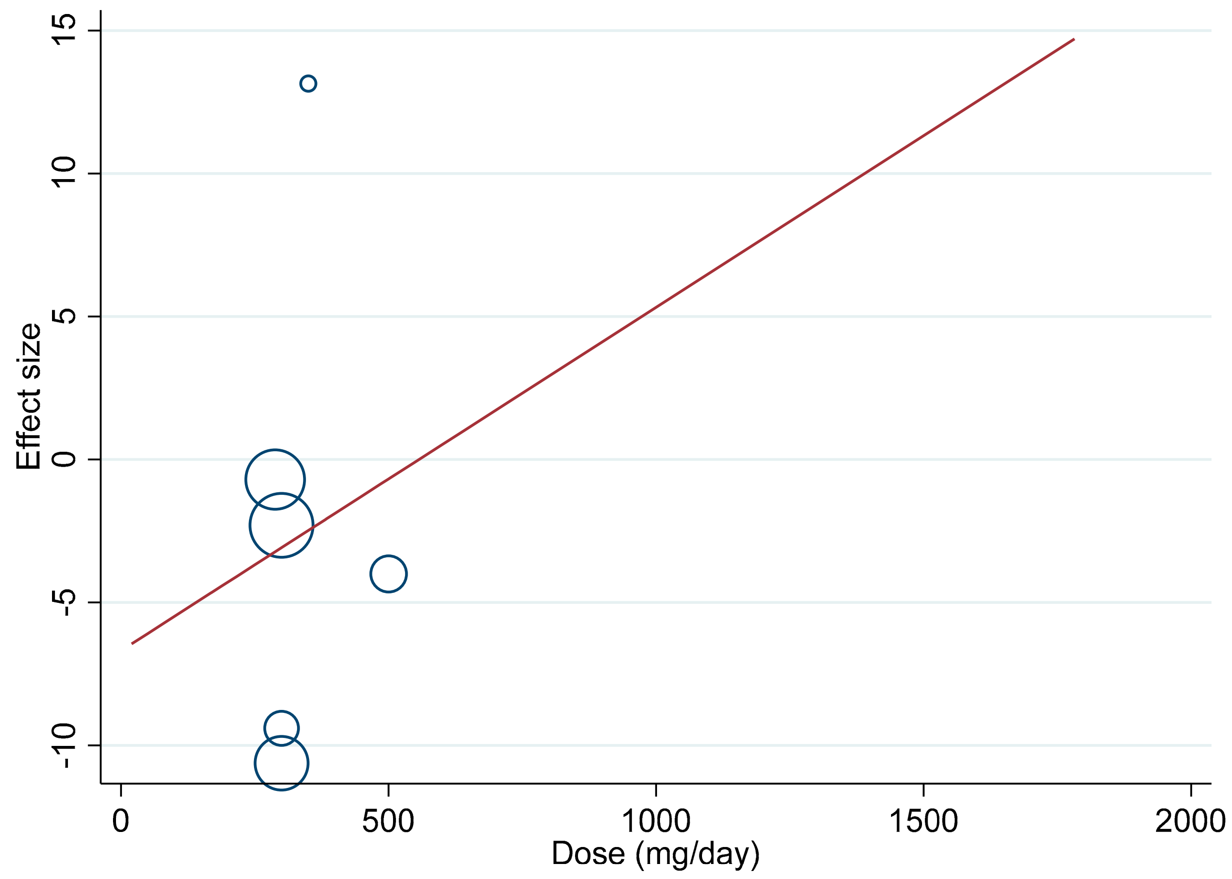

Figure S2. *Cont.*

U) Cr

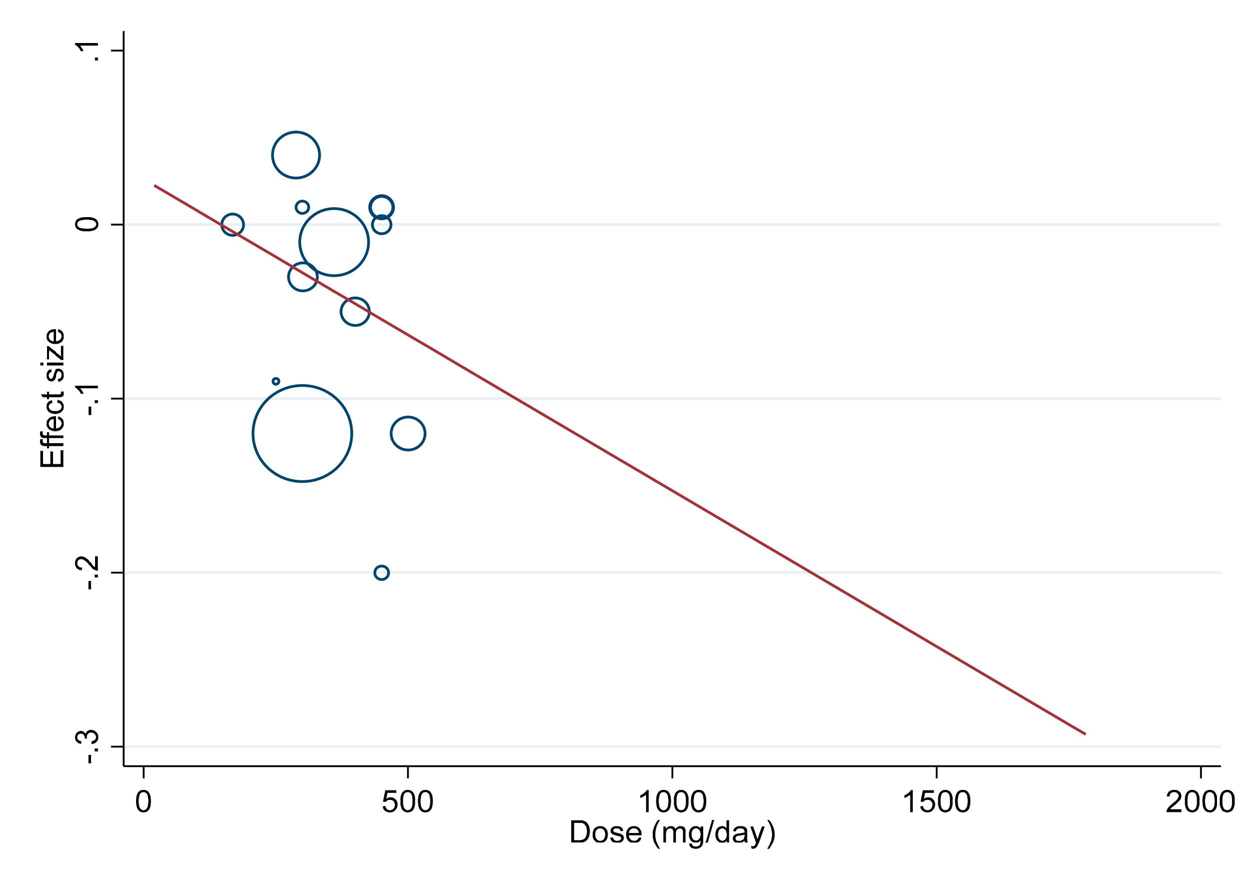

Figure S2. Linear dose-response association between dose (mg/day) of Mg supplementation and mean changes in cardiometabolic risk factors (CMRFs), including **(A)** BW (kg), **(B)** BMI (kg/m²), **(C)** WC (cm), **(D)** HC (cm), **(E)** BFP (%), **(F)** FBG (mg/dL), **(G)** HbA1c (%), **(H)** FI (μIU/mL), **(I)** HOMA-IR, **(J)** TG (mg/dL), **(K)** TC (mg/dL), **(L)** LDL-C (mg/dL), **(M)** HDL-C (mg/dL), **(N)** SBP (mmHg), **(O)** DBP (mmHg), **(P)** CRP (mg/L), **(Q)** IL-6 (pg/mL), **(R)** TNF-α (pg/mL), **(S)** AST (IU/L), **(T)** ALT (IU/L), and **(U)** Cr (mg/dL).

A) BW

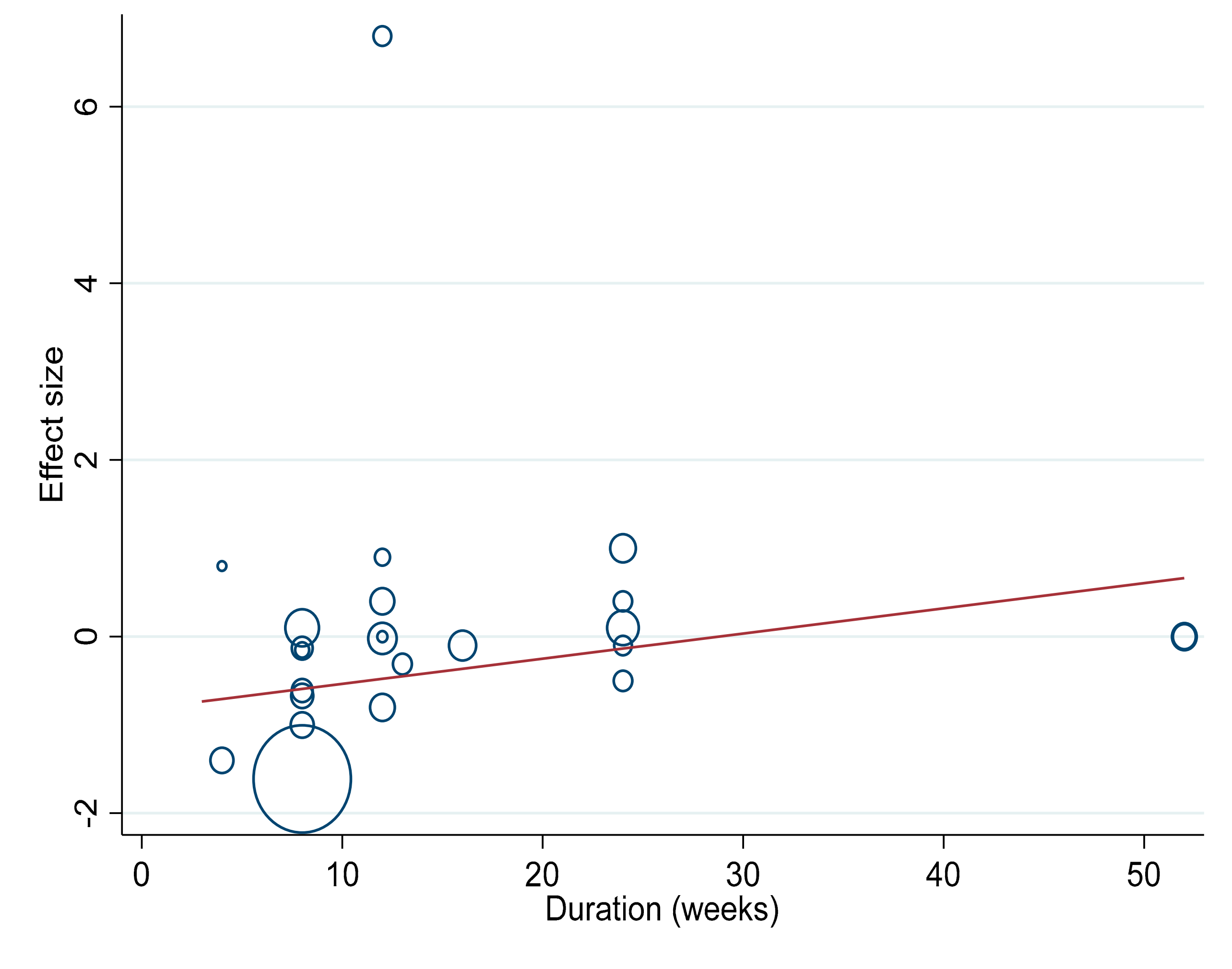

B) BMI

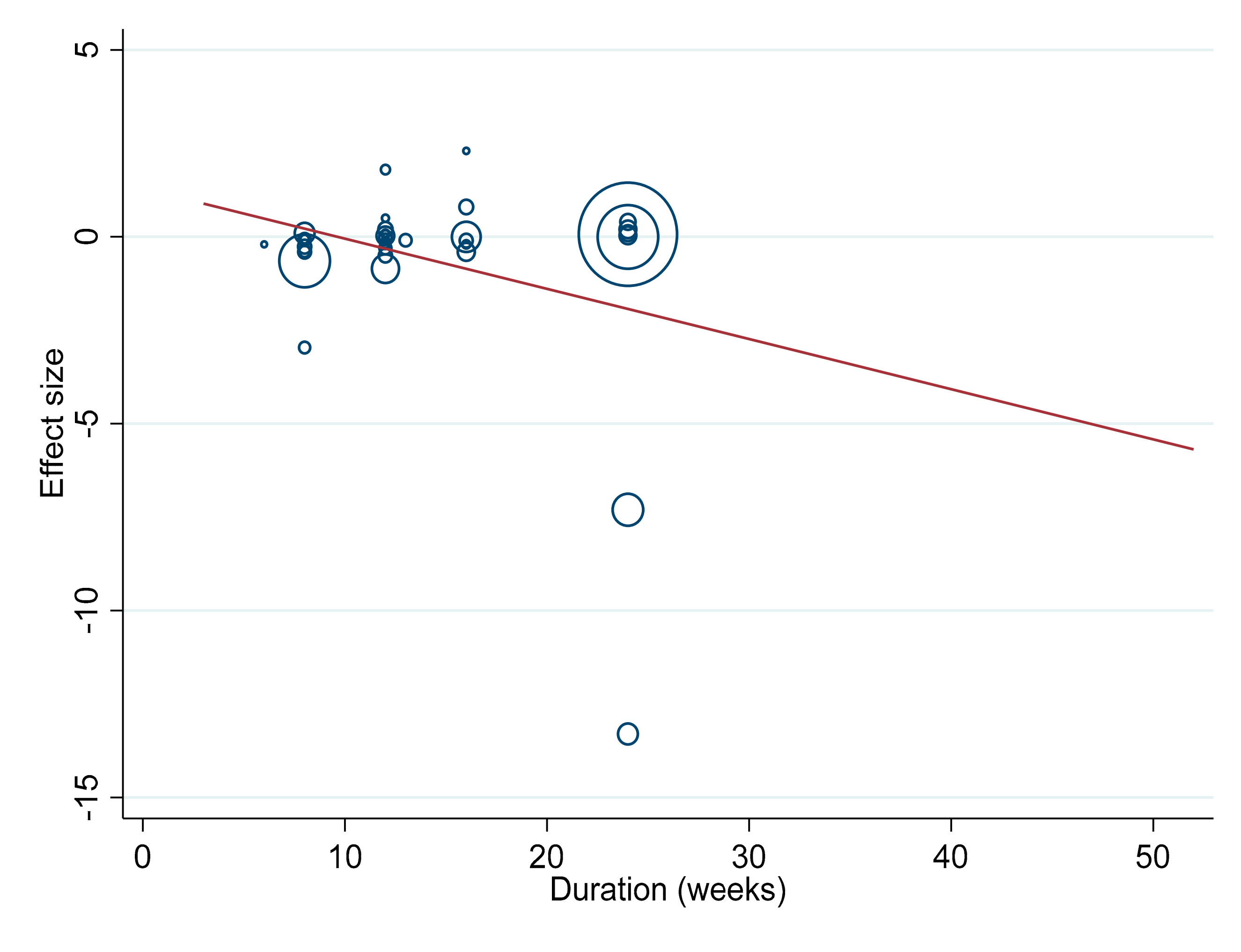

Figure S3. *Cont.*

C) WC

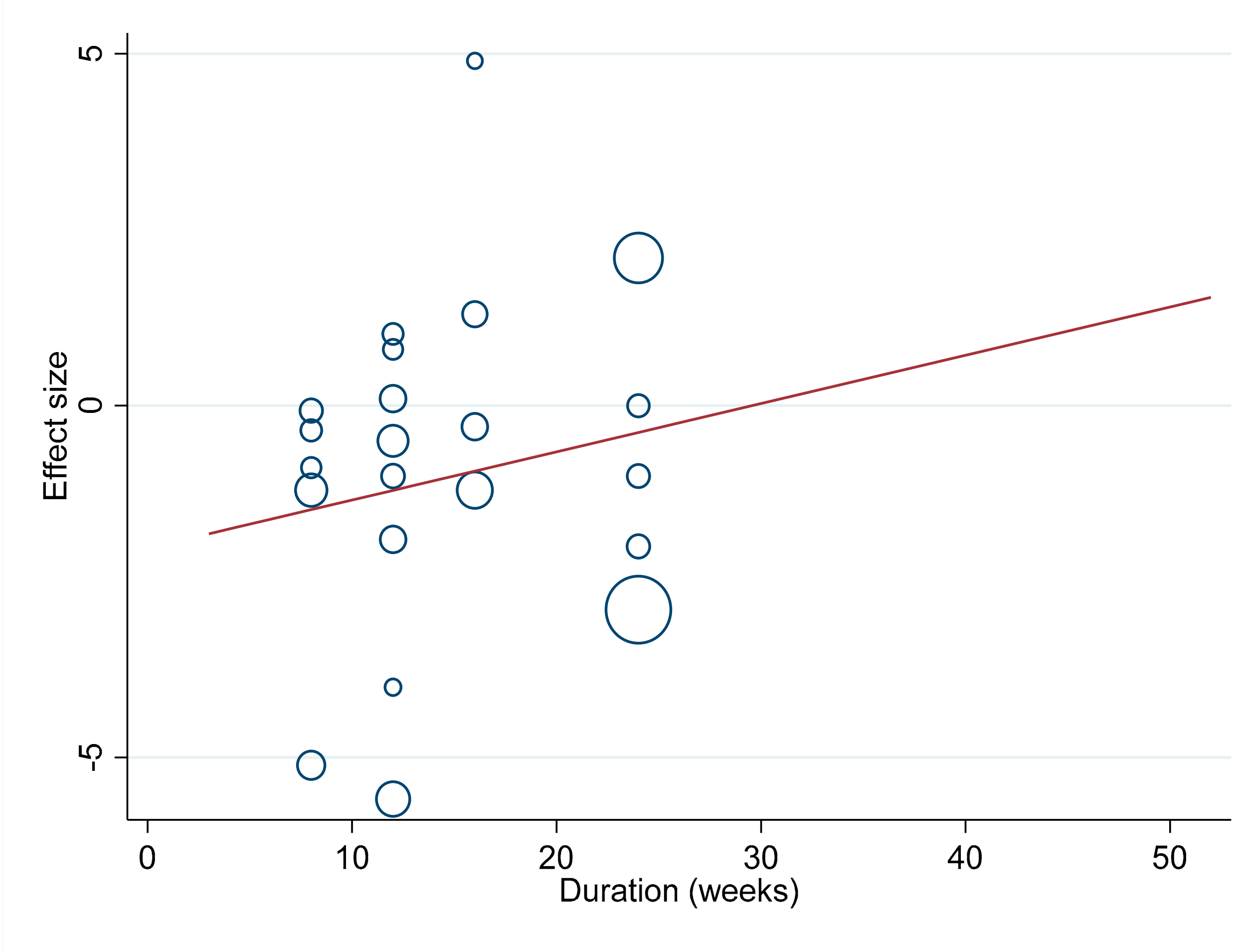

D) HC

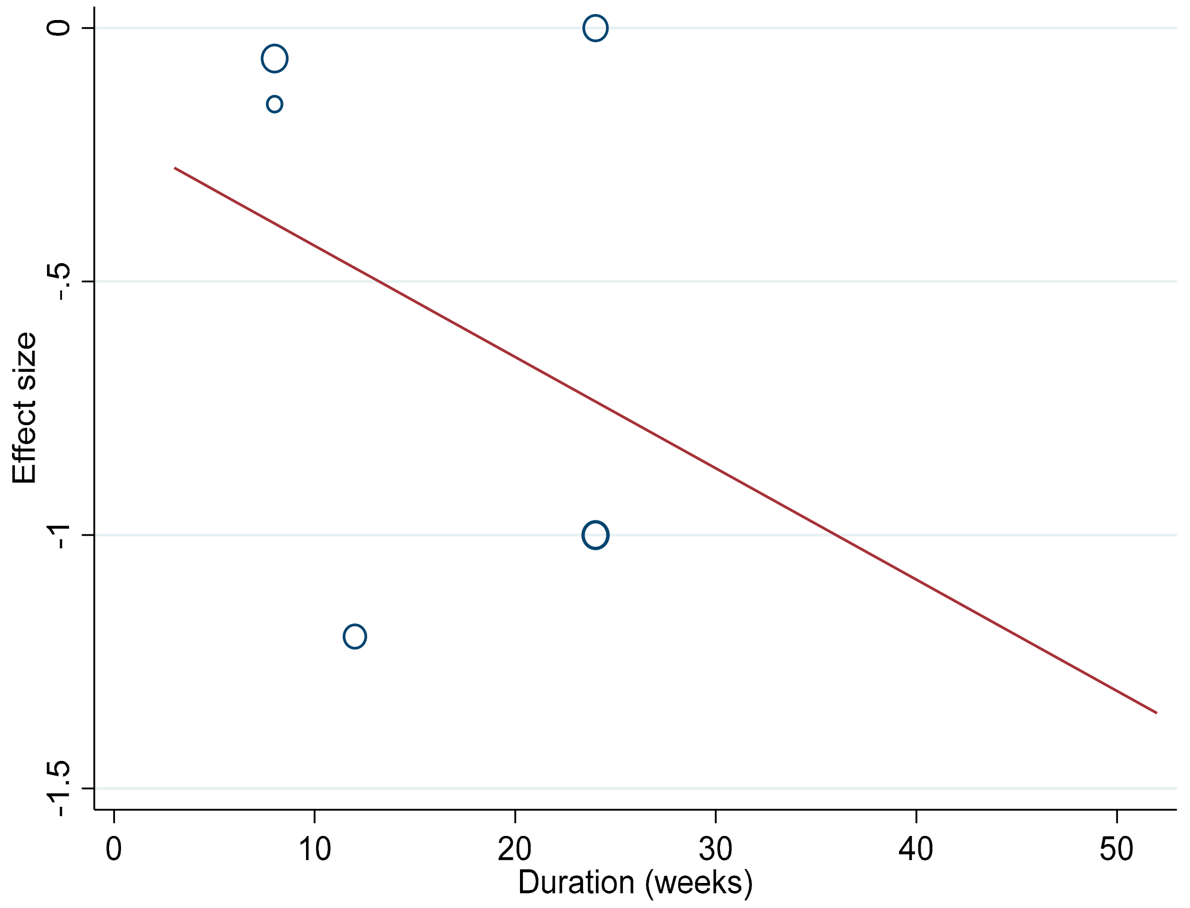

Figure S3. *Cont.*

E) **BFP**

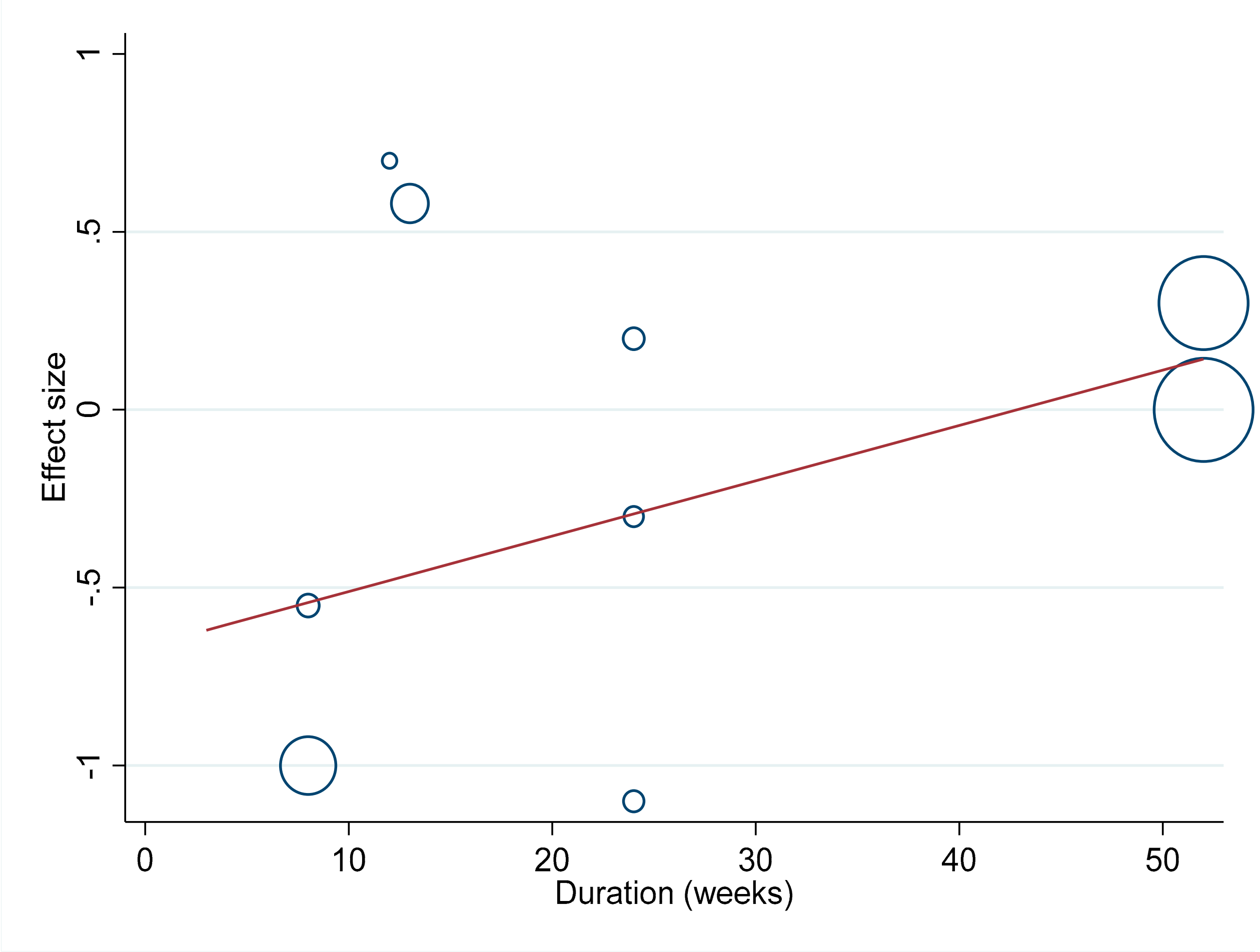

F) **FBG**

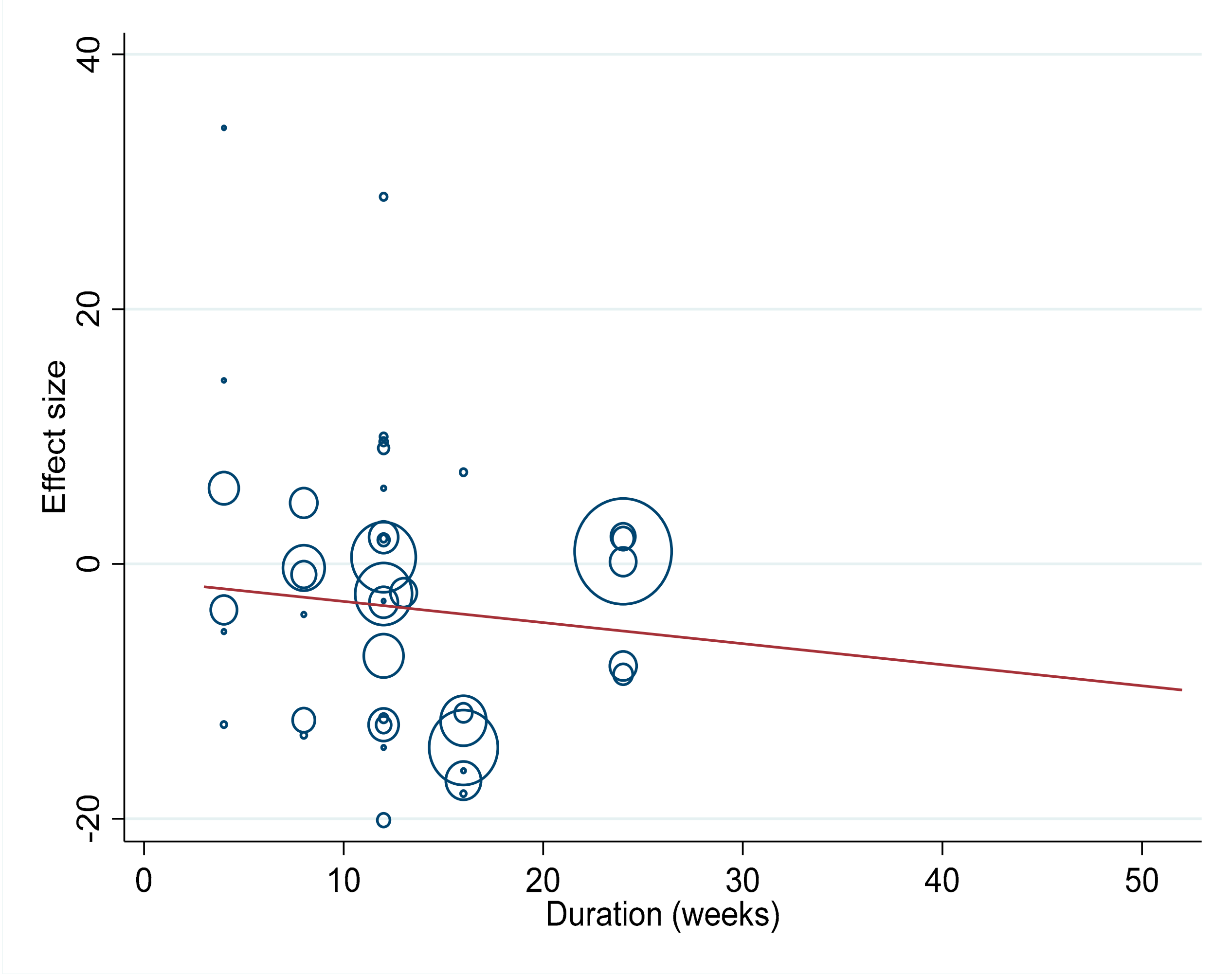

Figure S3. *Cont.*

G) HbA1c

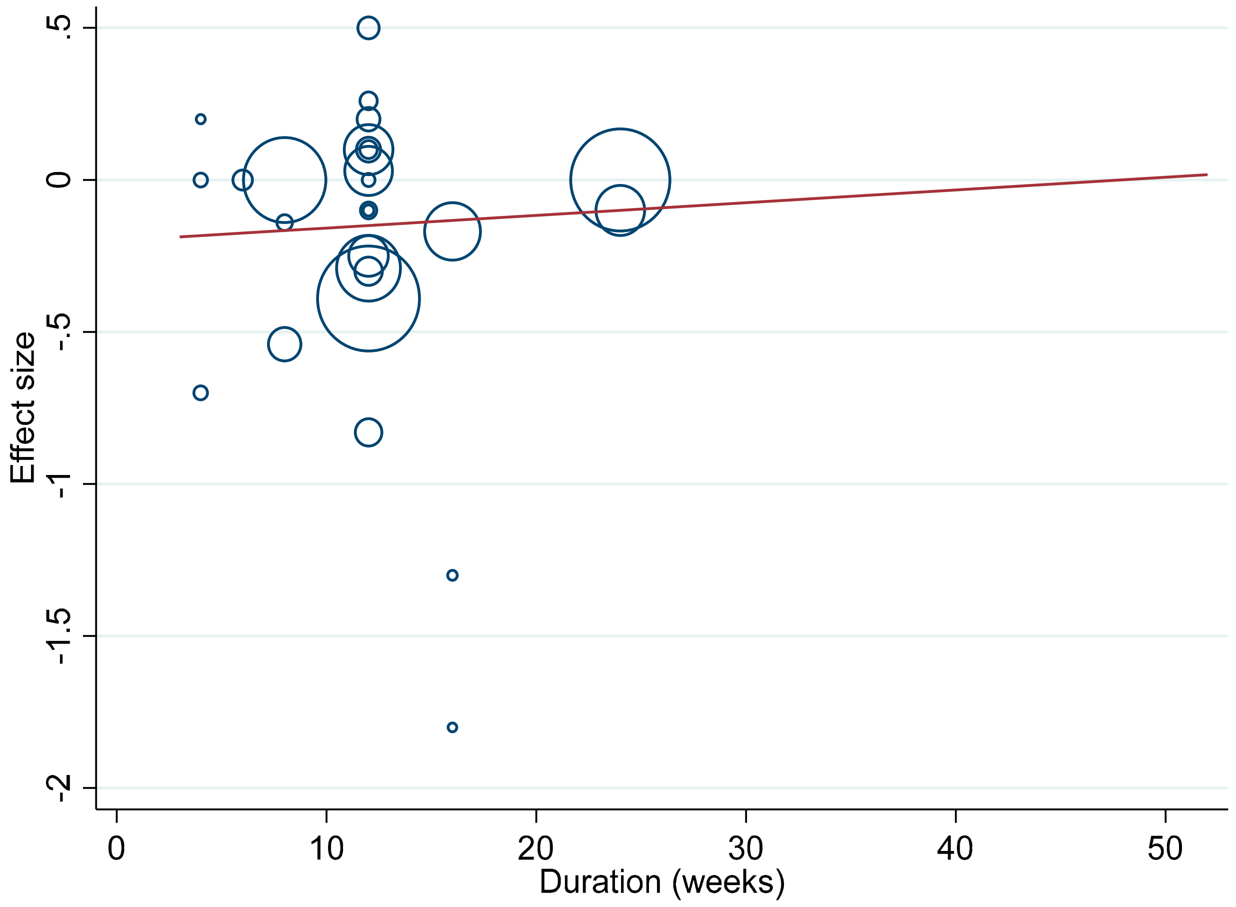

H) FI

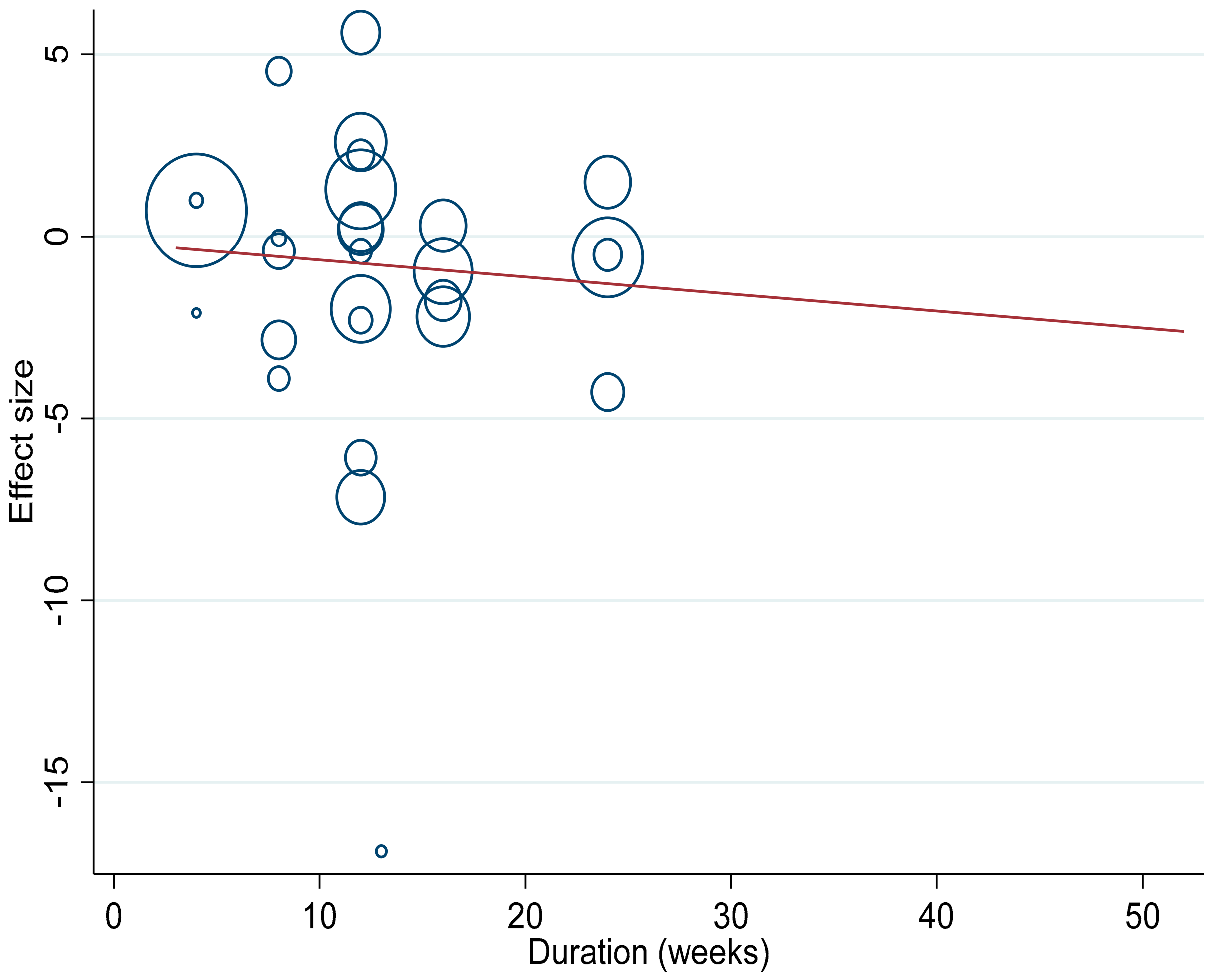

Figure S3. *Cont.*

I) HOMA-IR

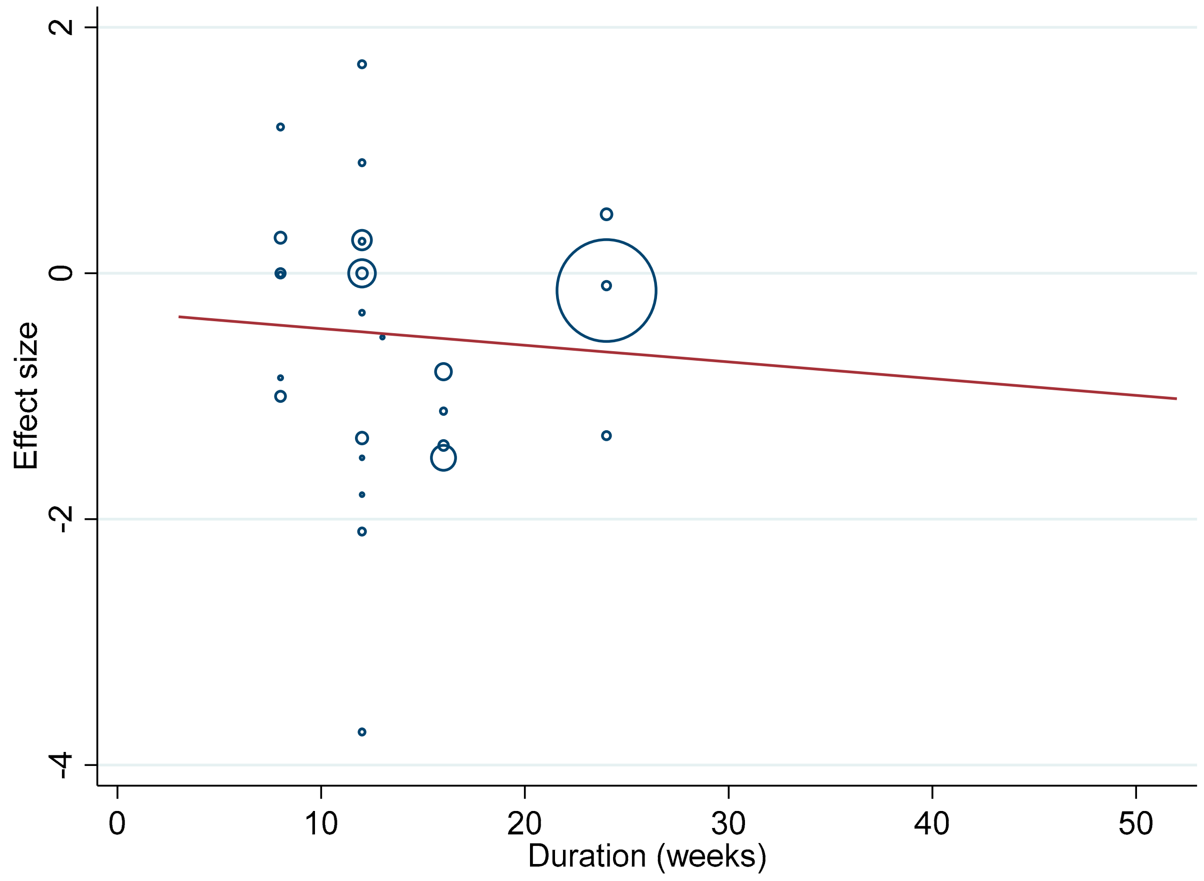

J) TG

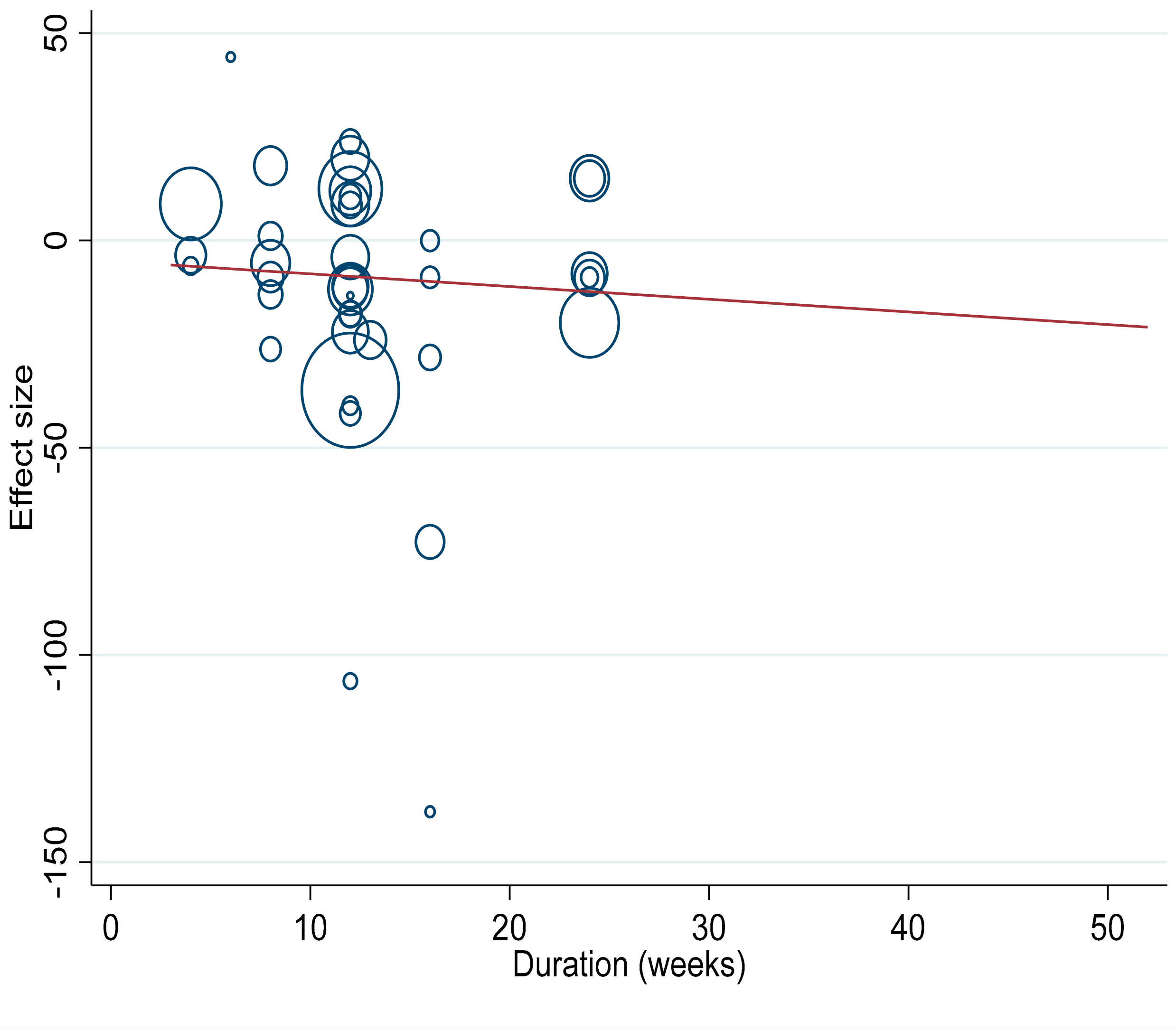

Figure S3. *Cont.*

K) TC

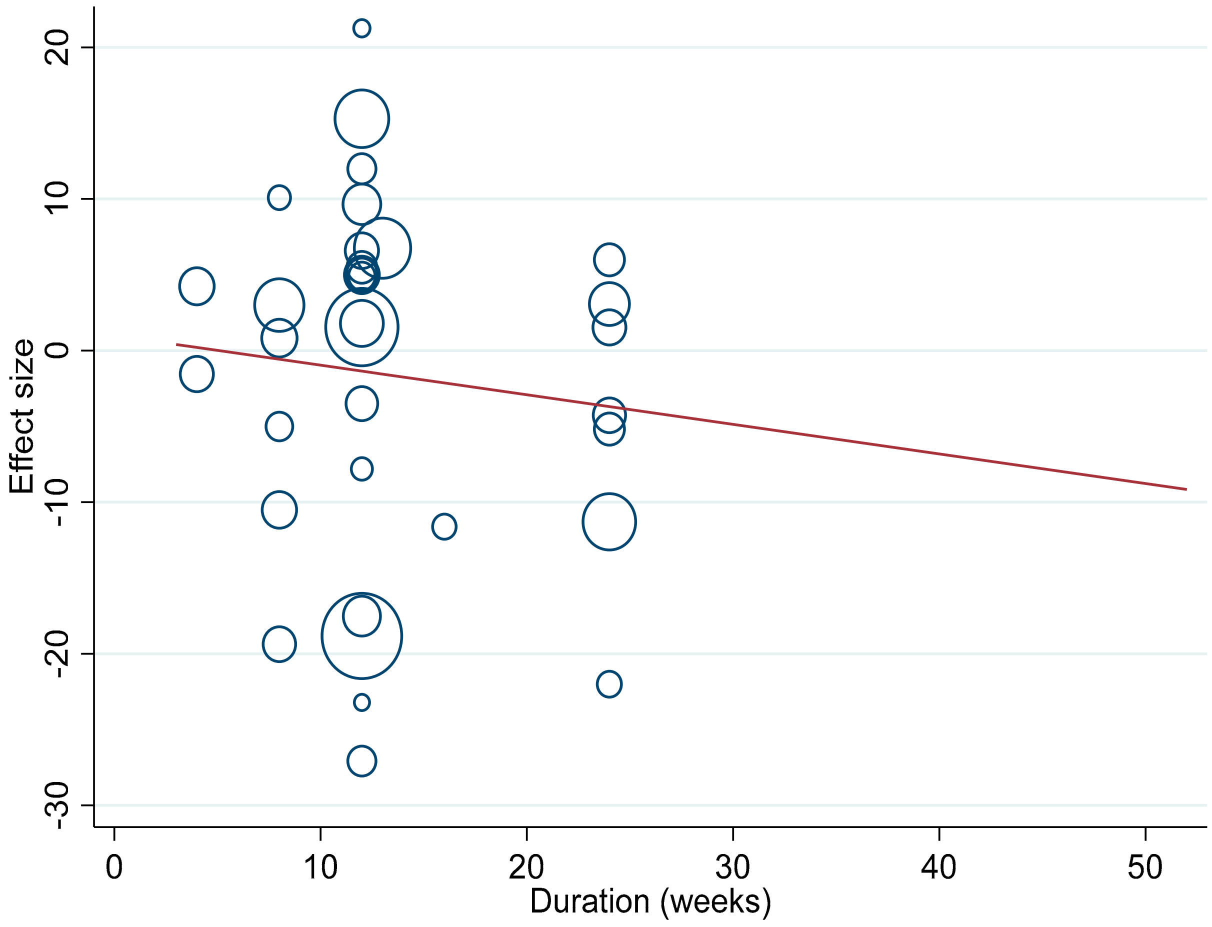

L) LDL-C

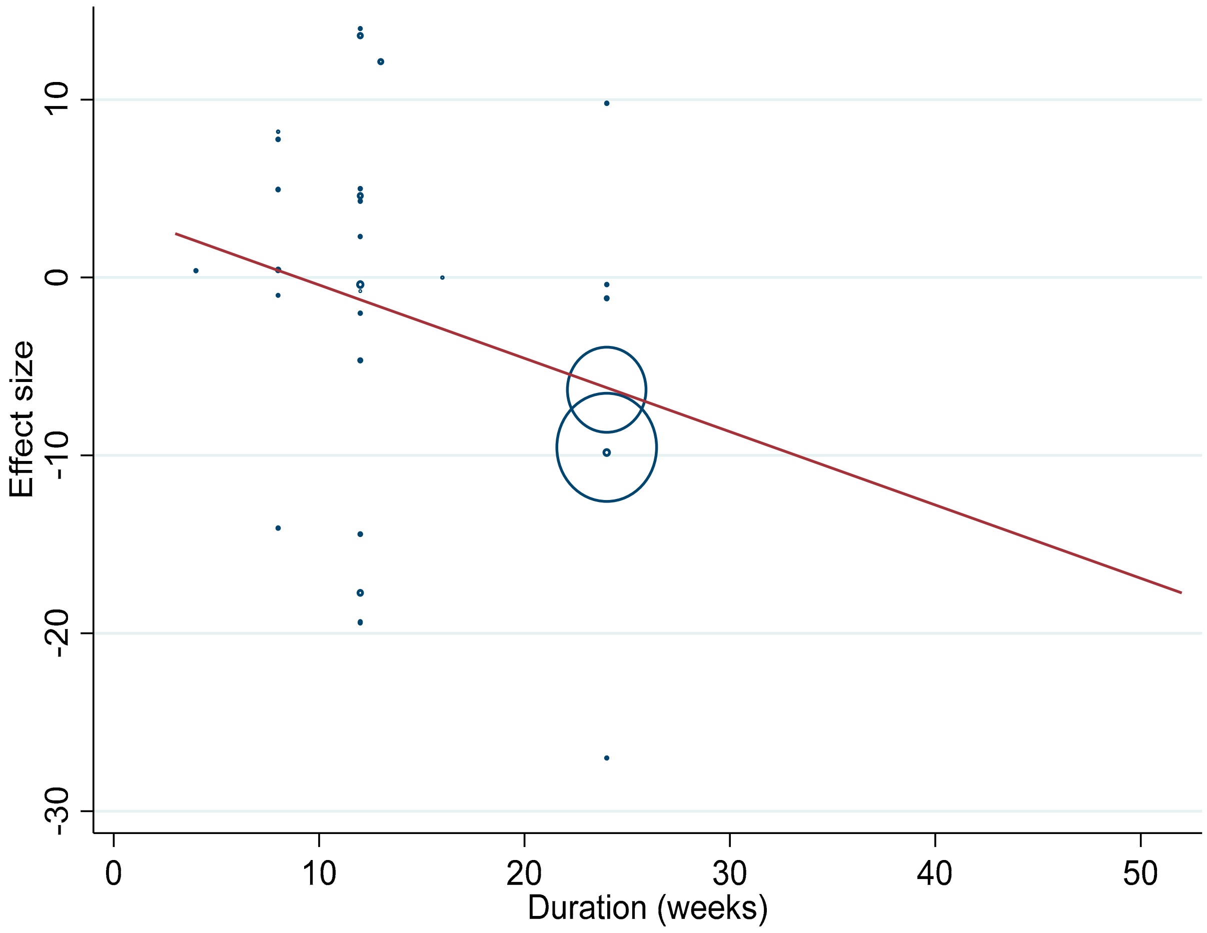

Figure S3. *Cont.*

M) HDL-C

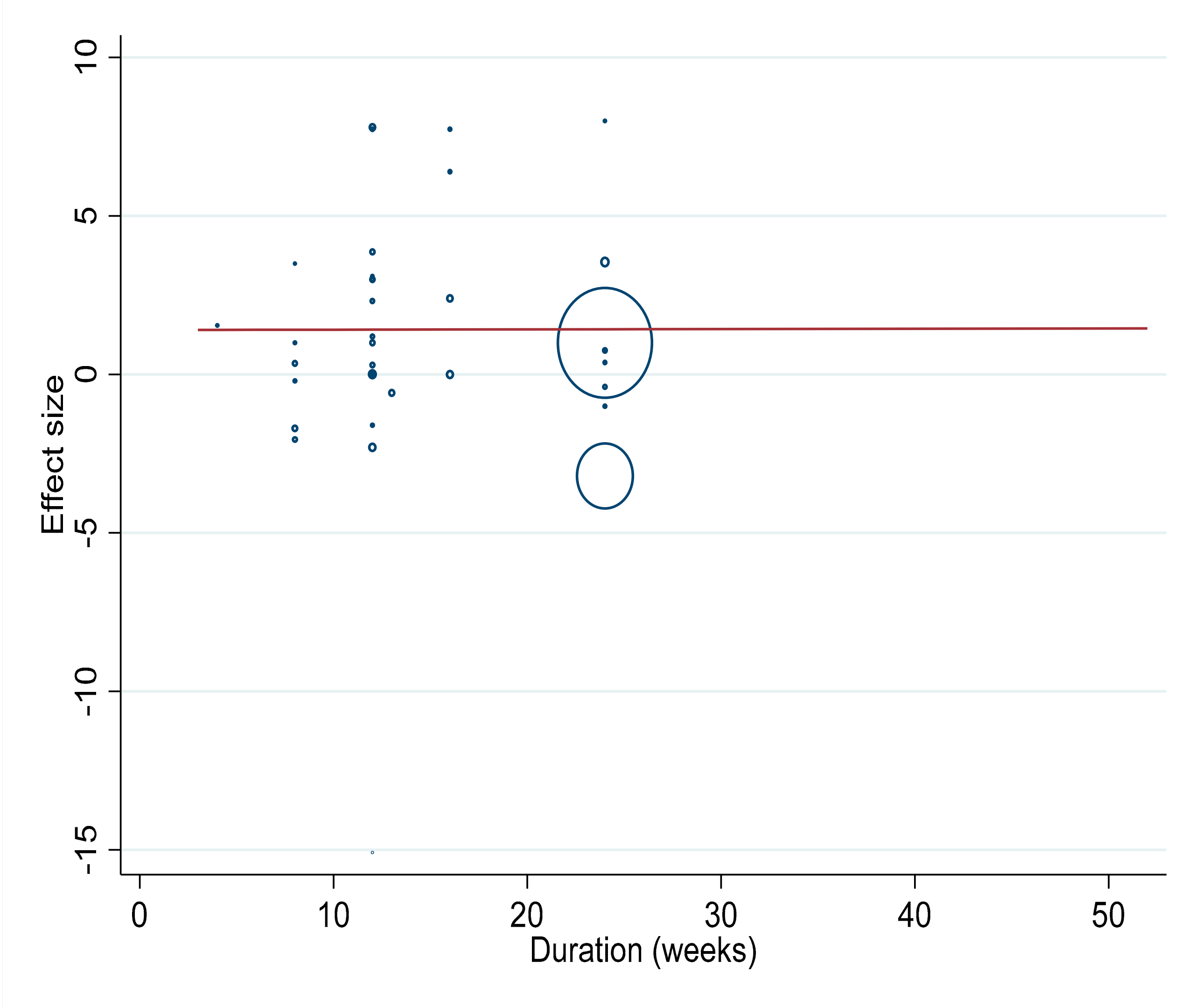

N) SBP

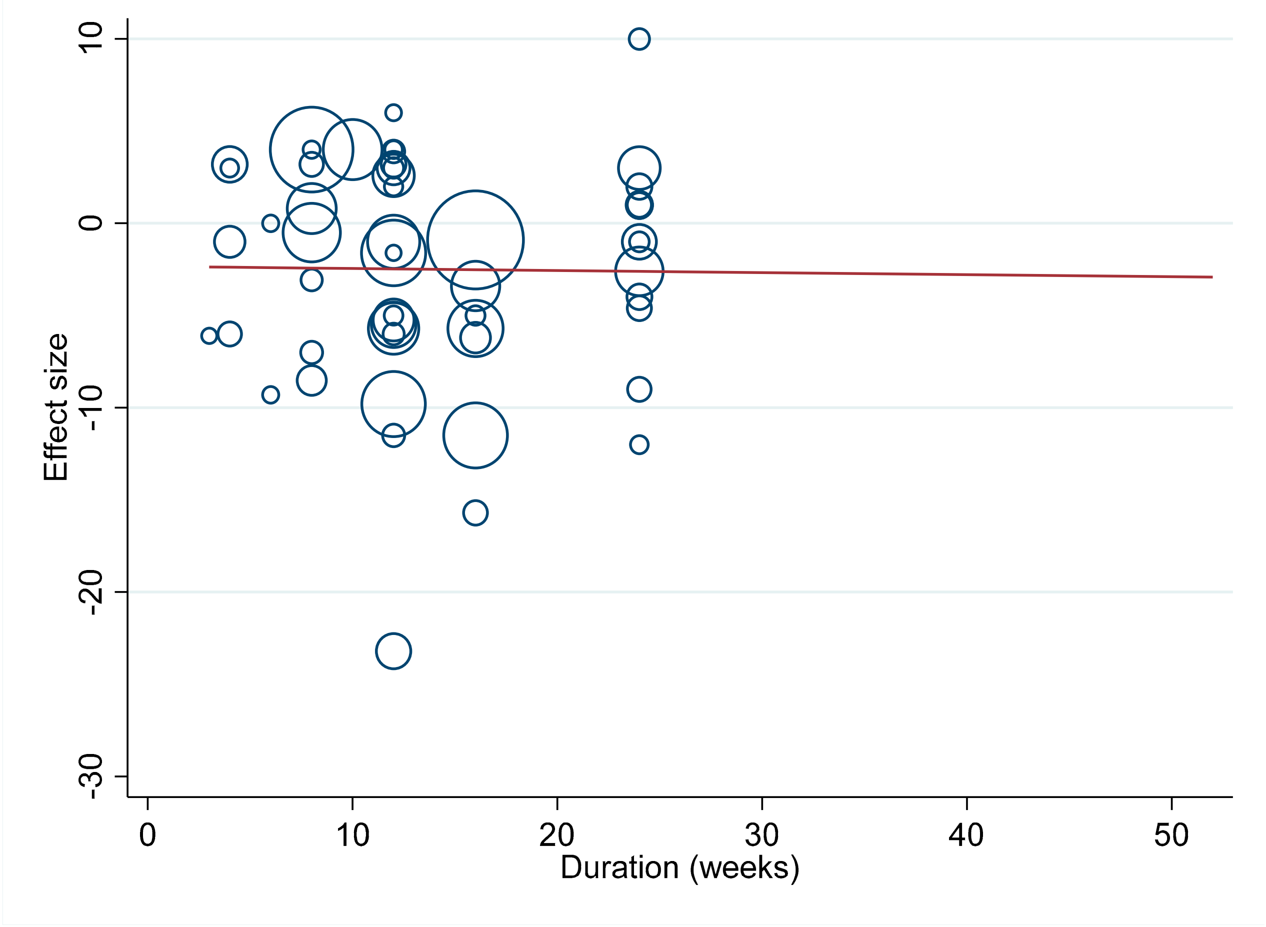

Figure S3. *Cont.*

O) DBP

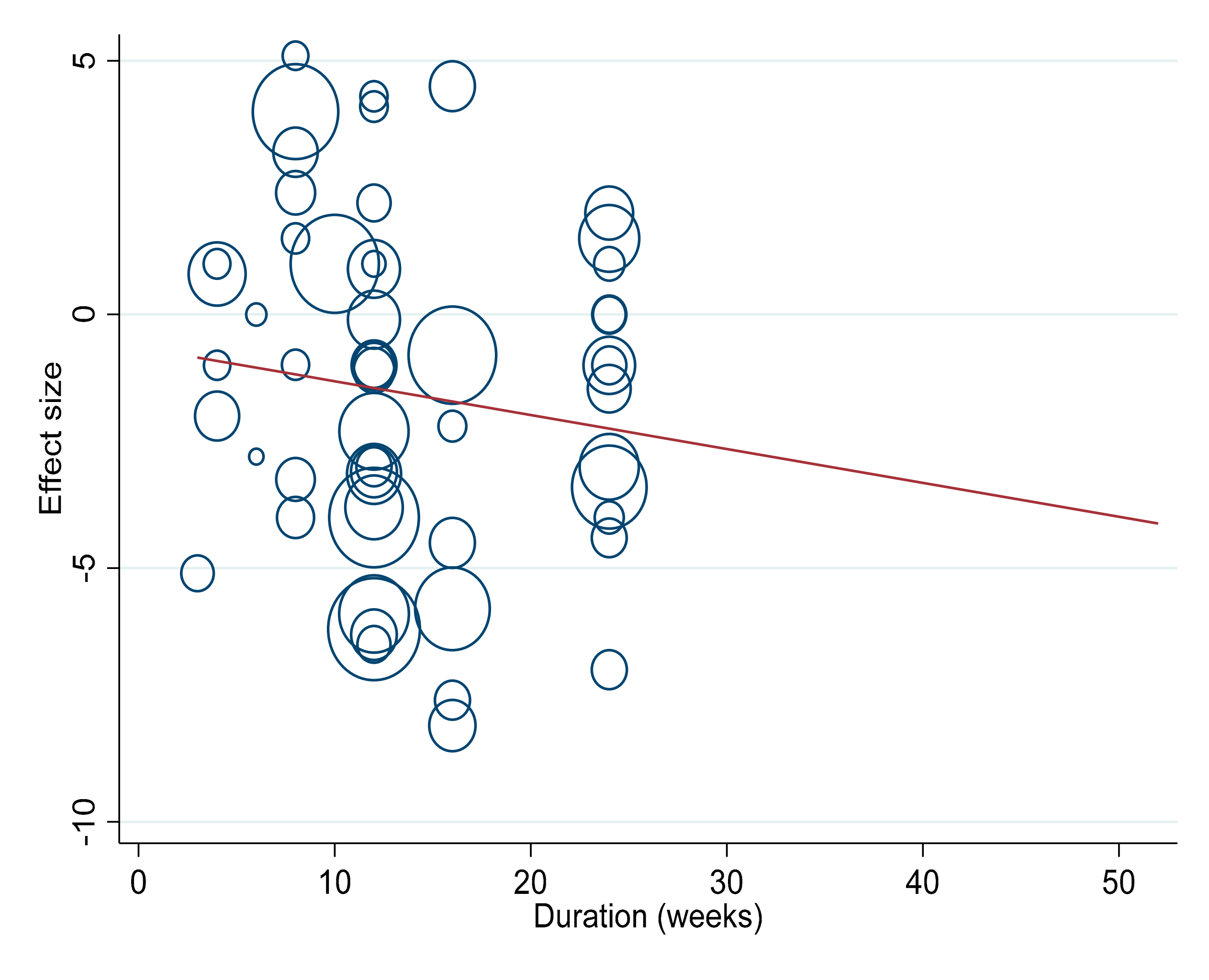

P) CRP

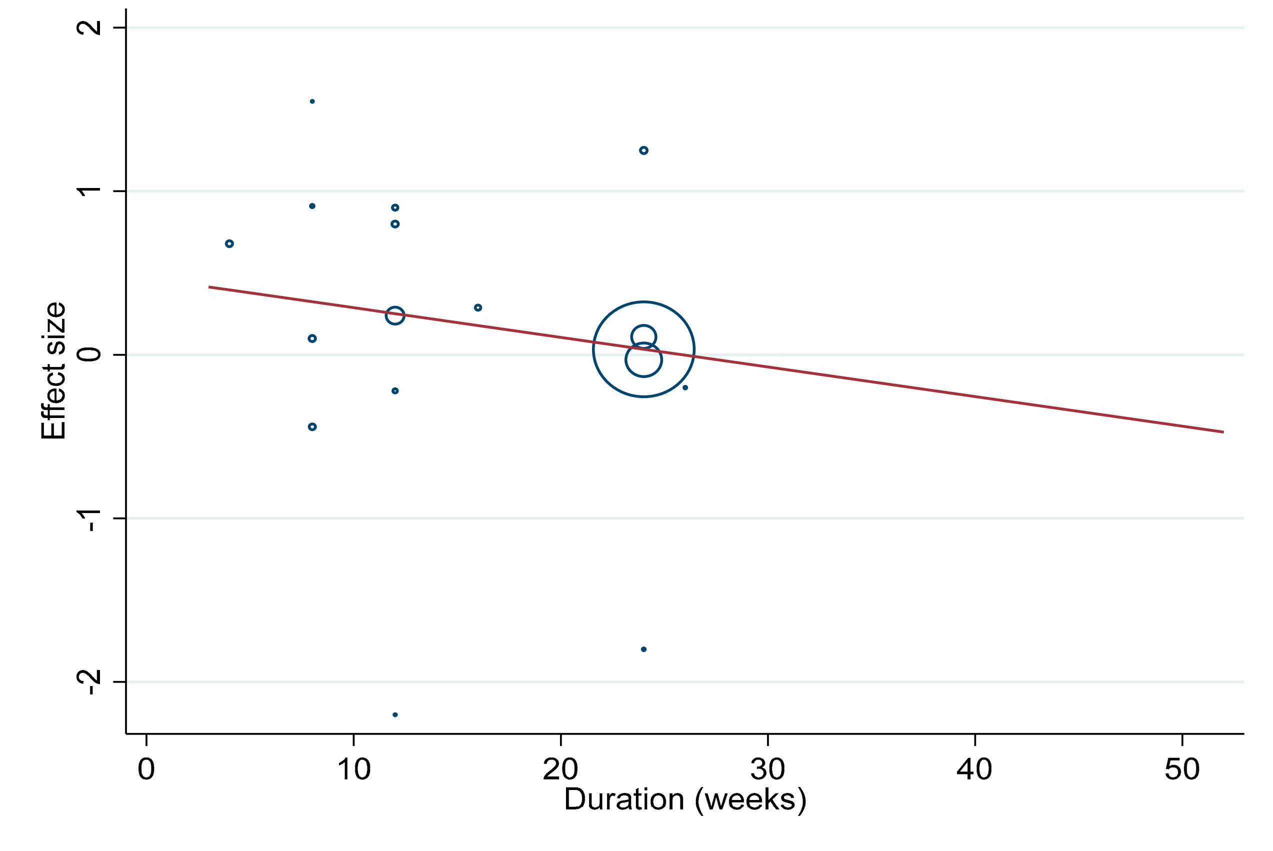

Figure S3. *Cont.*

Q) IL-6

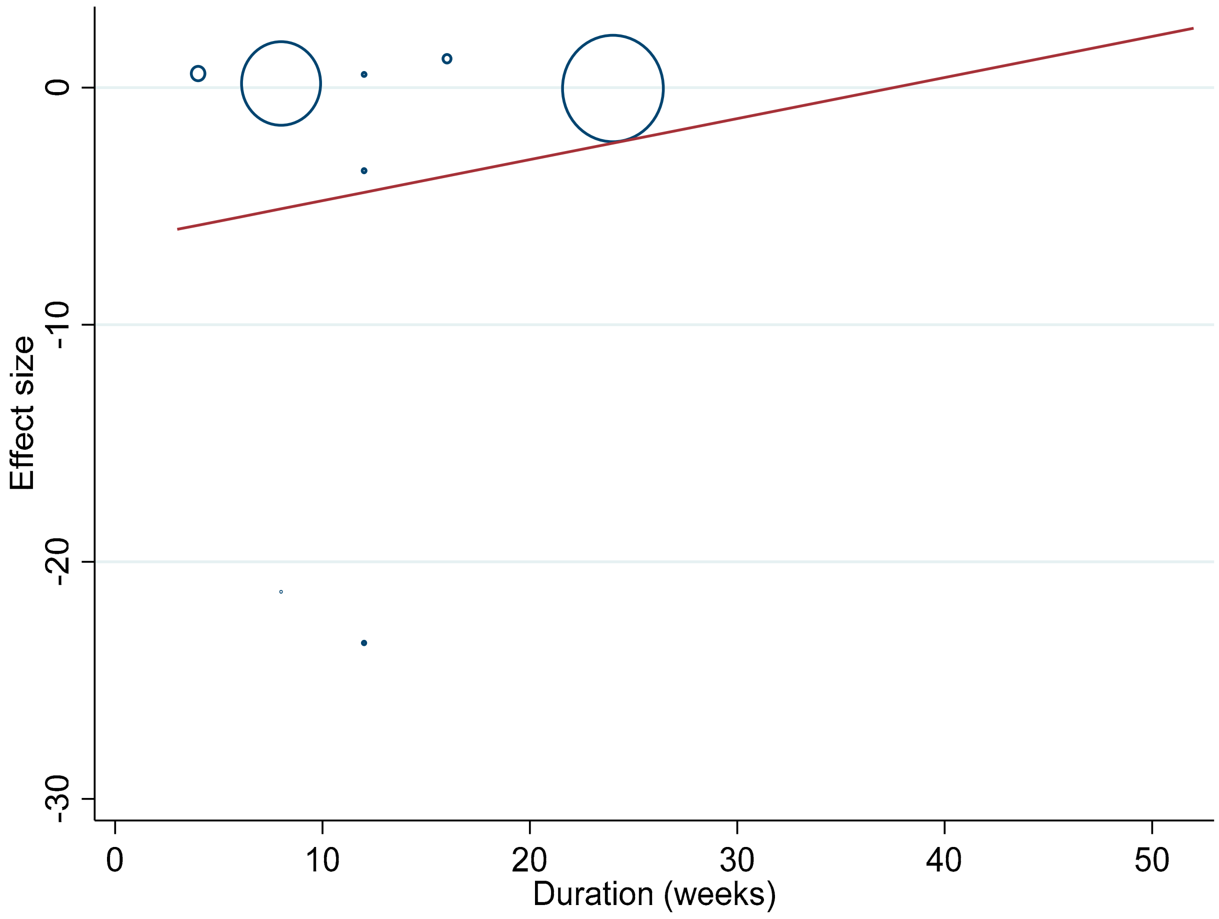

R) TNF-α

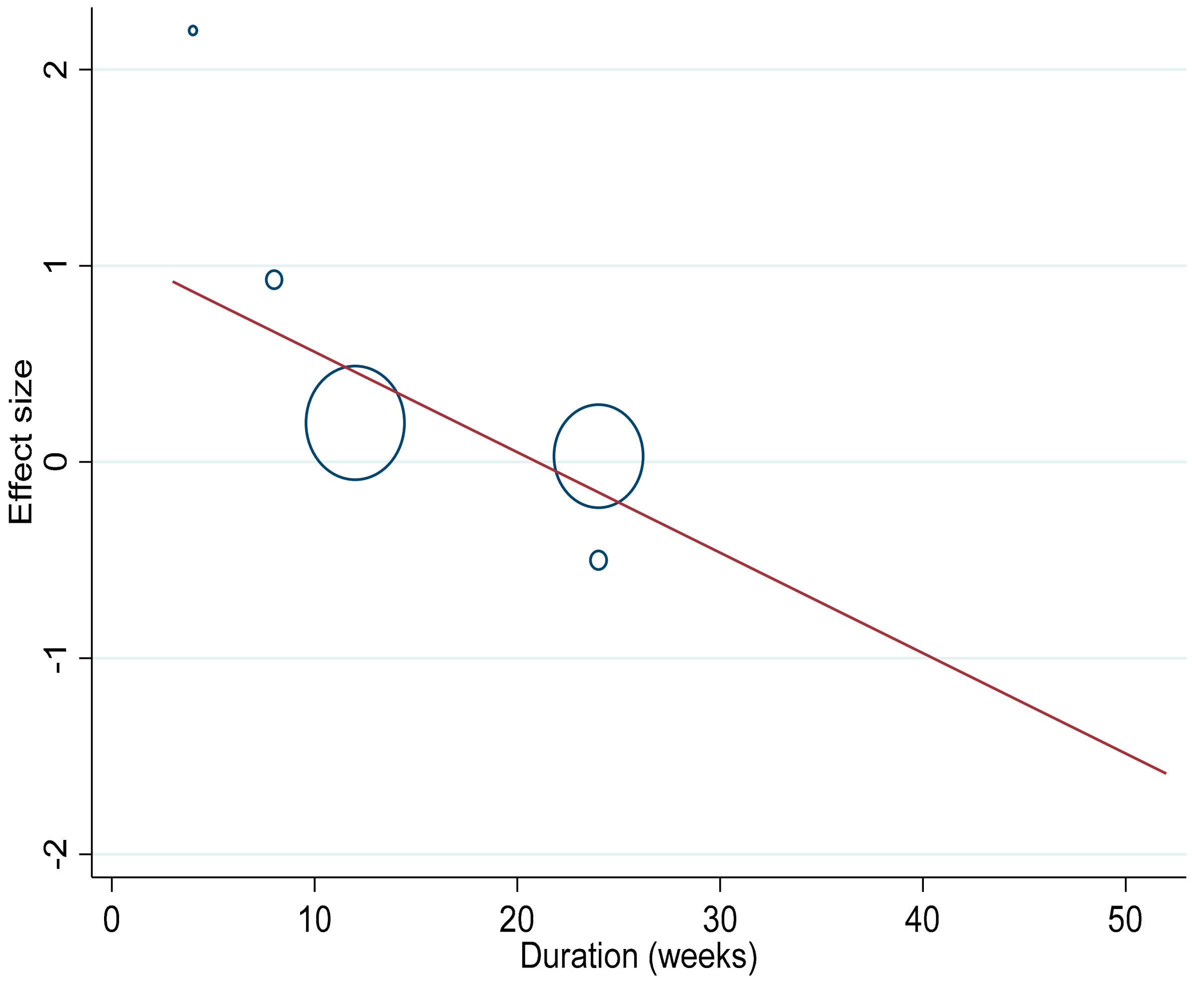

Figure S3. *Cont.*

S) AST

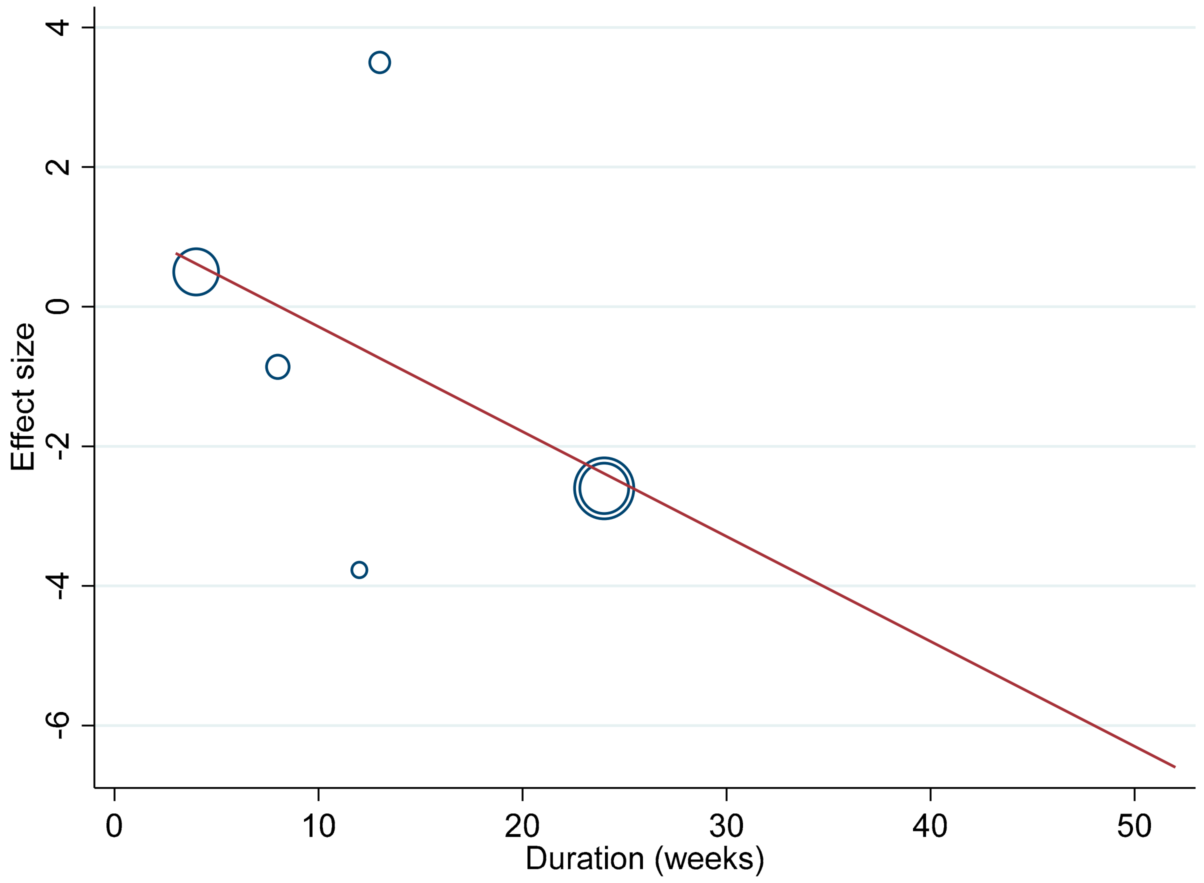

T) ALT

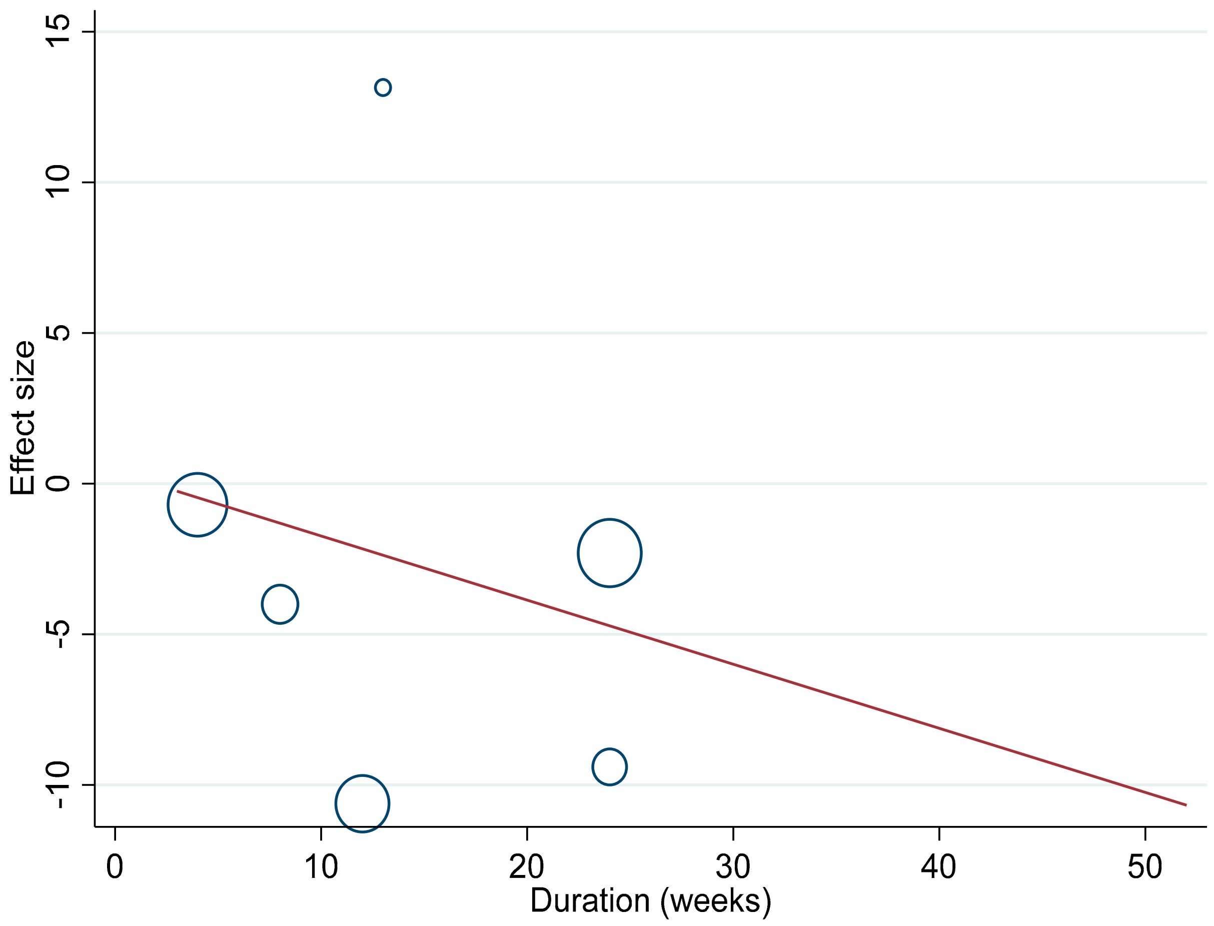

Figure S3. *Cont.*

U) Cr

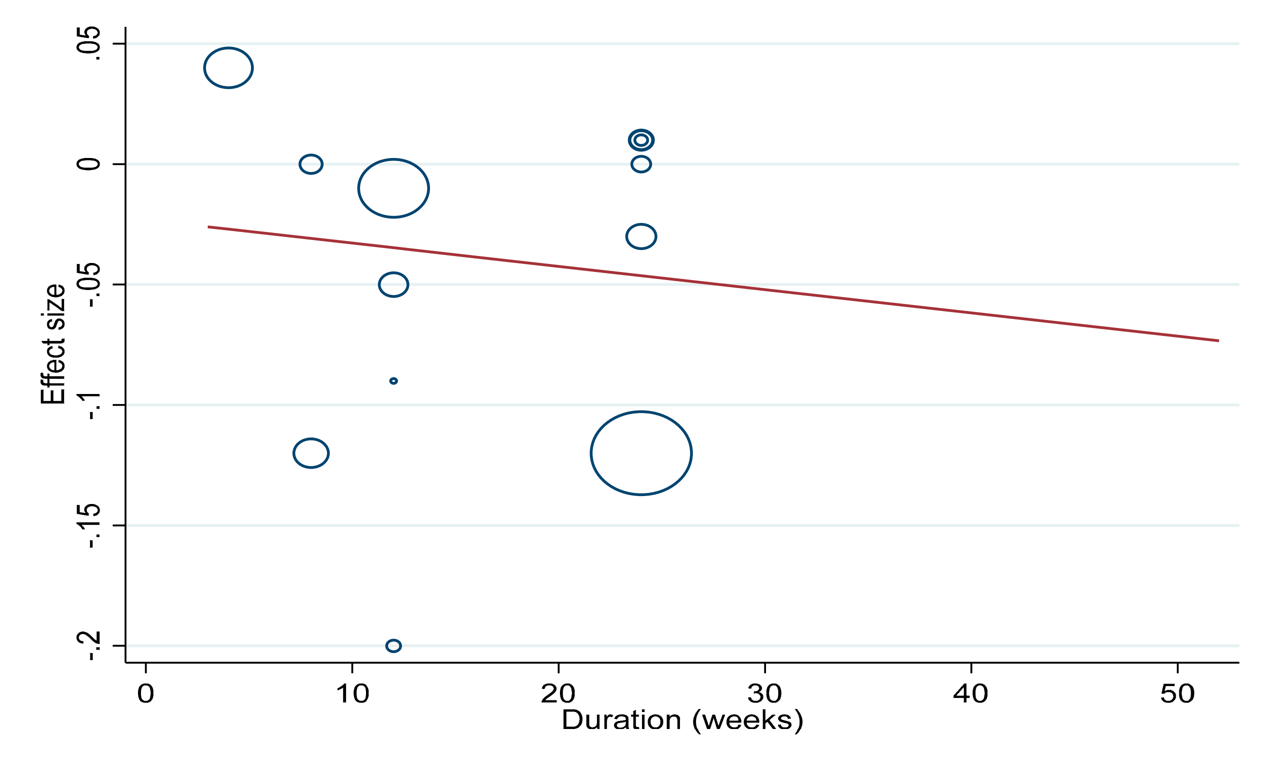

Figure S3. Linear association between duration (weeks) of Mg supplementation and mean changes in cardiometabolic risk factors (CMRFs), including **(A)** BW (kg), **(B)** BMI (kg/m²), **(C)** WC (cm), **(D)** HC (cm), **(E)** BFP (%), **(F)** FBG (mg/dL), **(G)** HbA1c (%), **(H)** FI (µIU/mL), **(I)** HOMA-IR, **(J)** TG (mg/dL), **(K)** TC (mg/dL), **(L)** LDL-C (mg/dL), **(M)** HDL-C (mg/dL), **(N)** SBP (mmHg), **(O)** DBP (mmHg), **(P)** CRP (mg/L), **(Q)** IL-6 (pg/mL), **(R)** TNF-α (pg/mL), **(S)** AST (IU/L), **(T)** ALT (IU/L), and **(U)** Cr (mg/dL).

A) BW

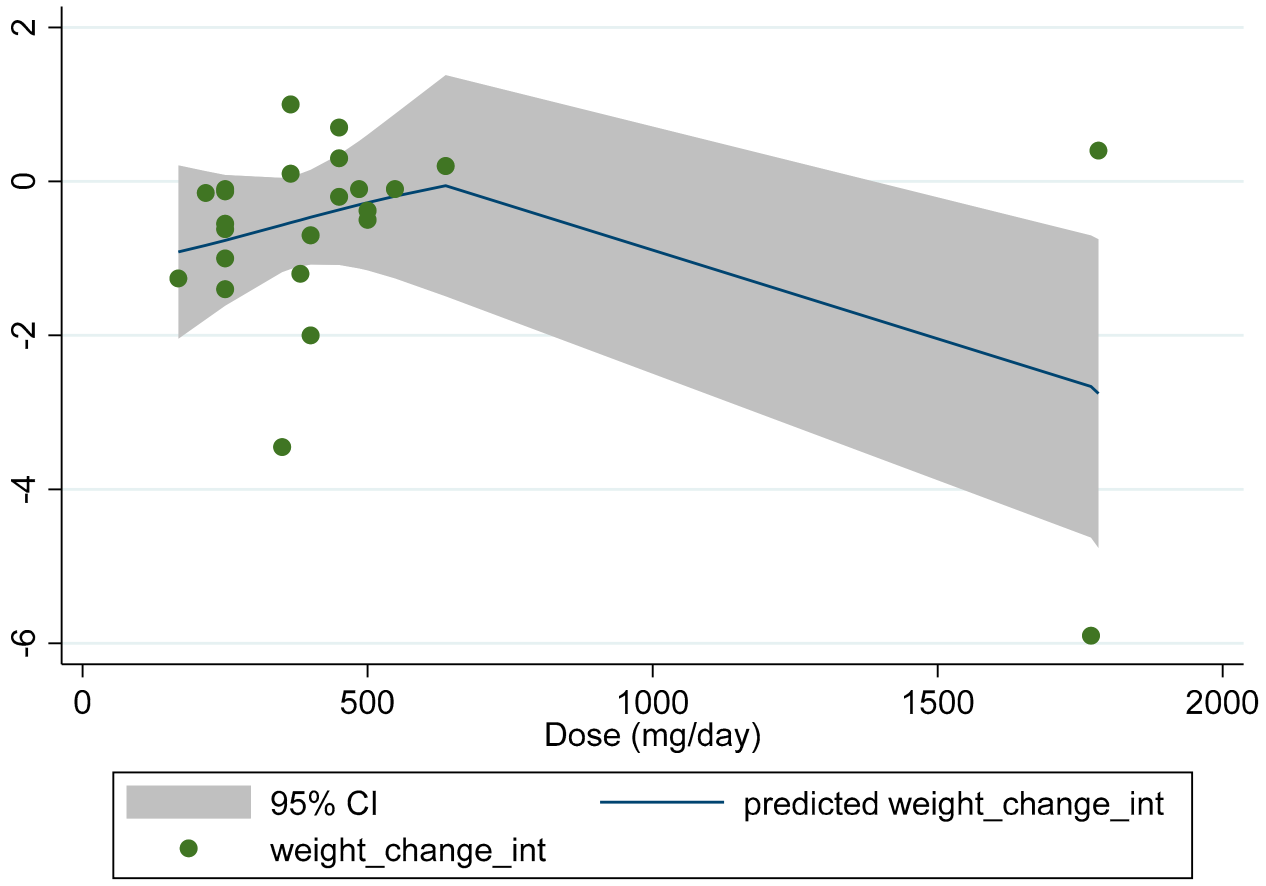

B) BMI

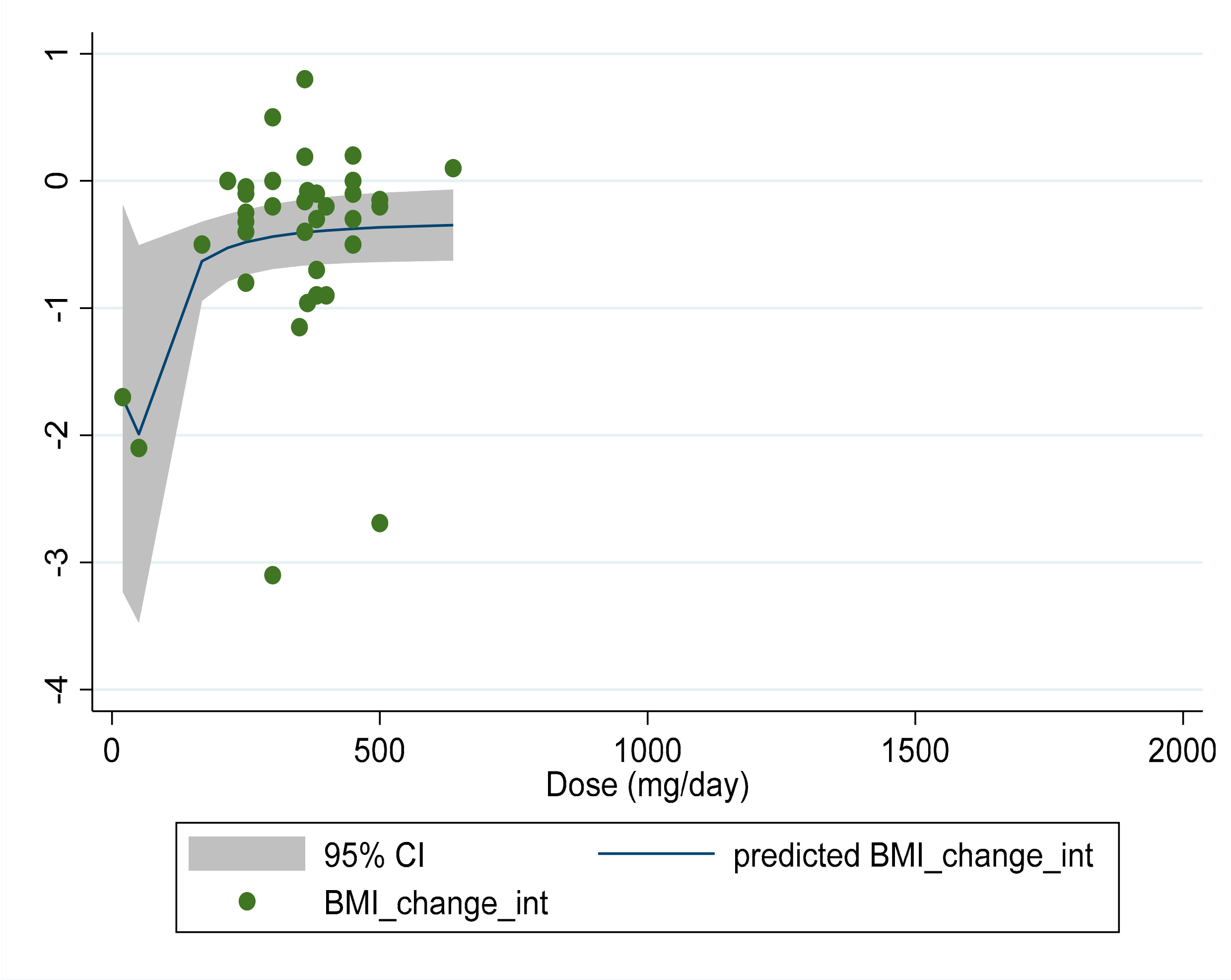

Figure S4. *Cont.*

C) WC

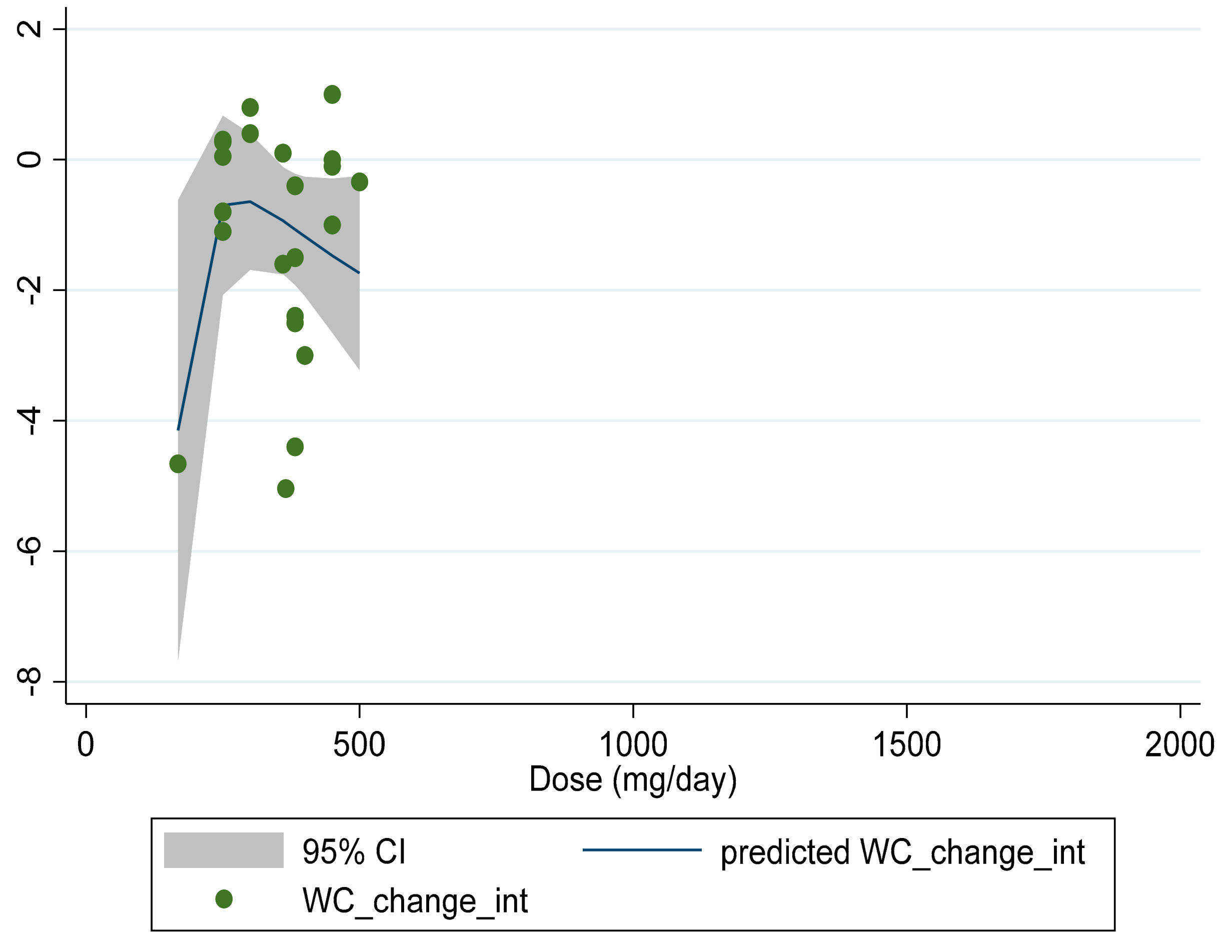

D) HC

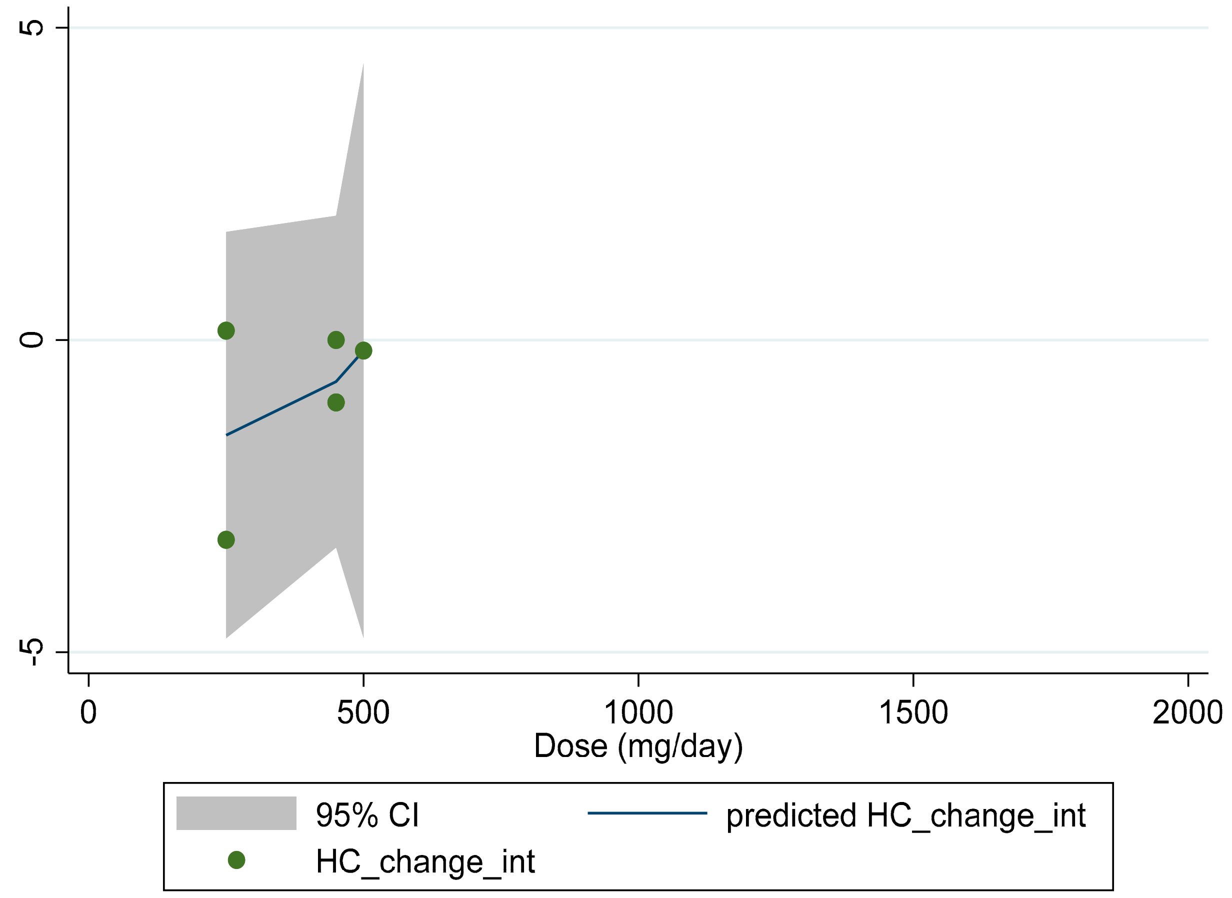

Figure S4. *Cont.*

E) **BFP**

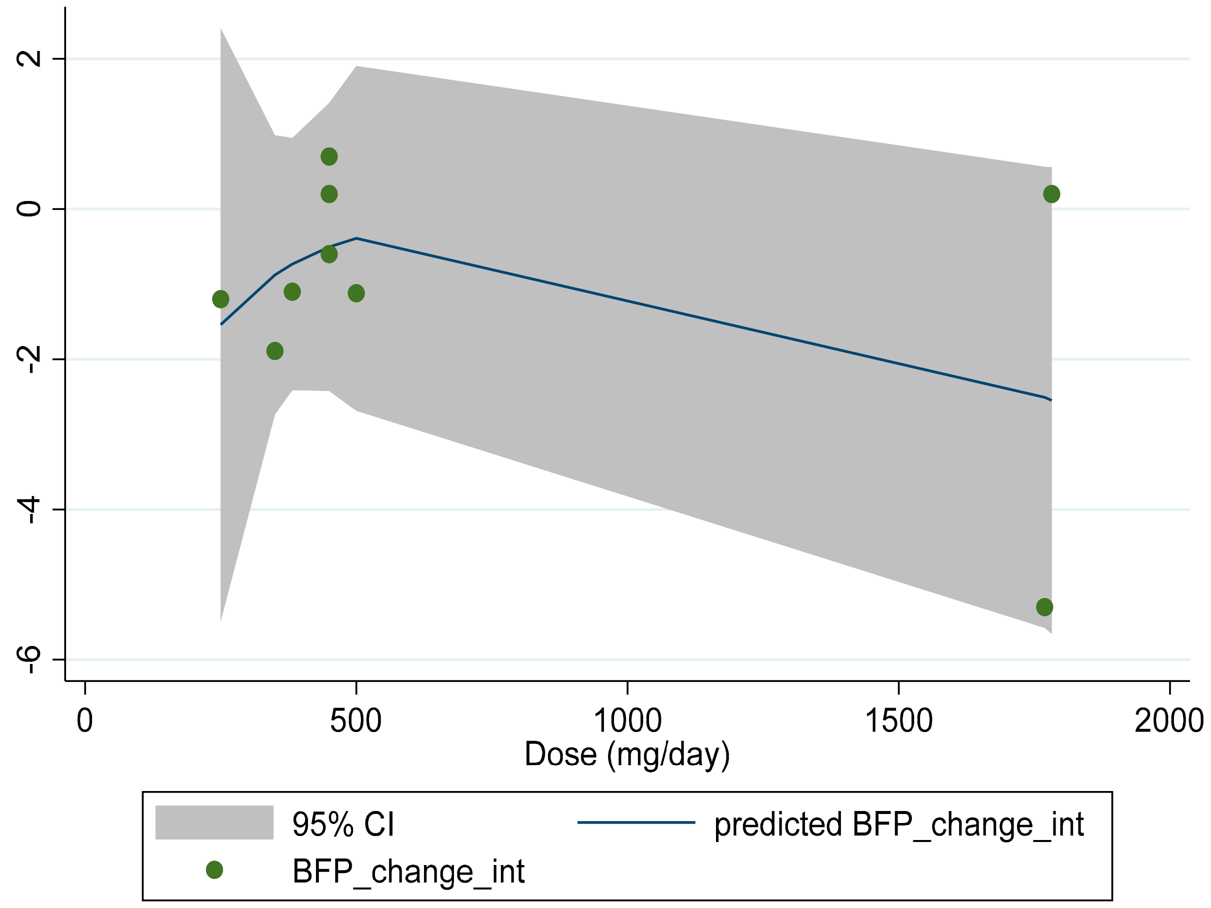

F) **FBG**

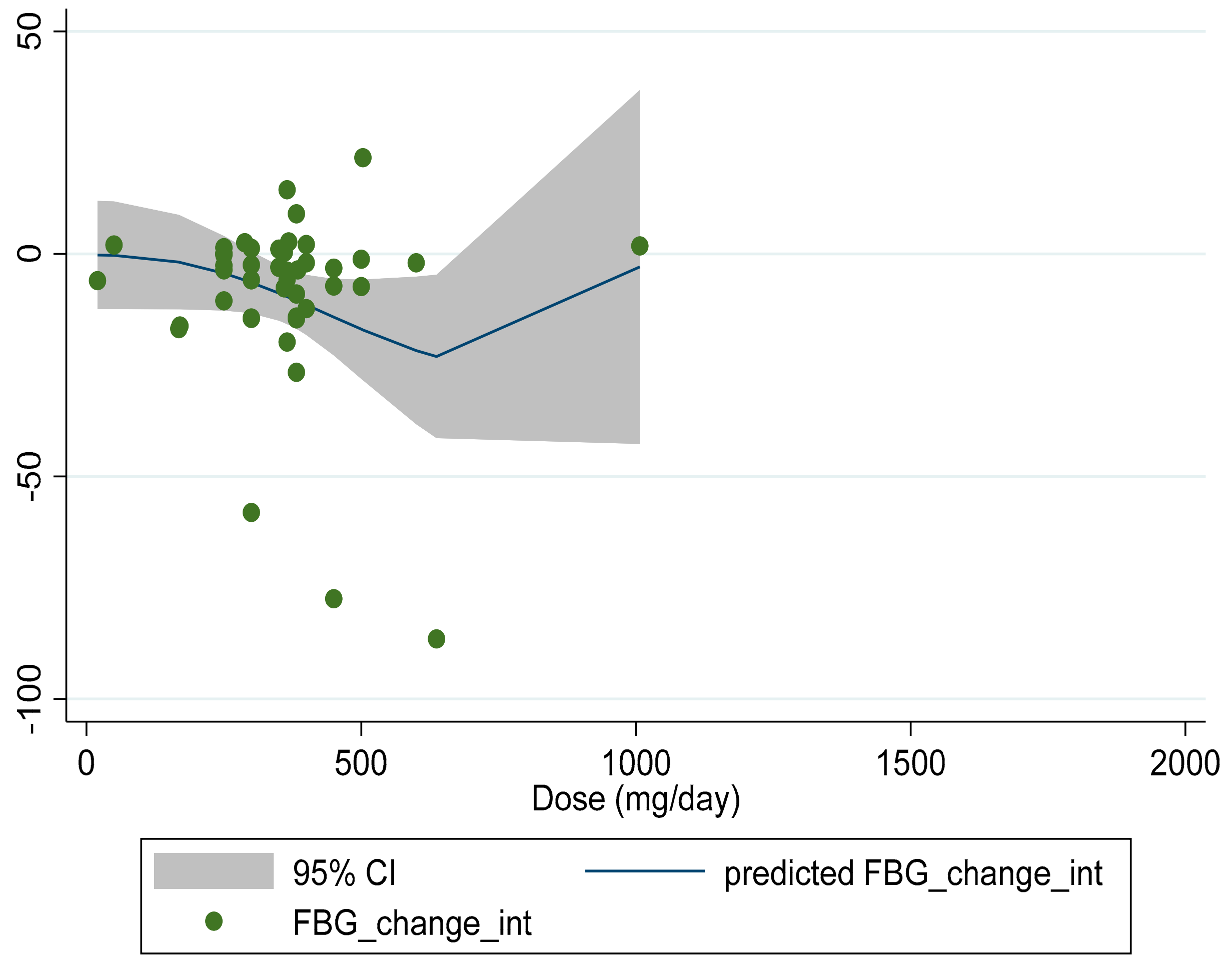

Figure S4. *Cont.*

G) HbA1c

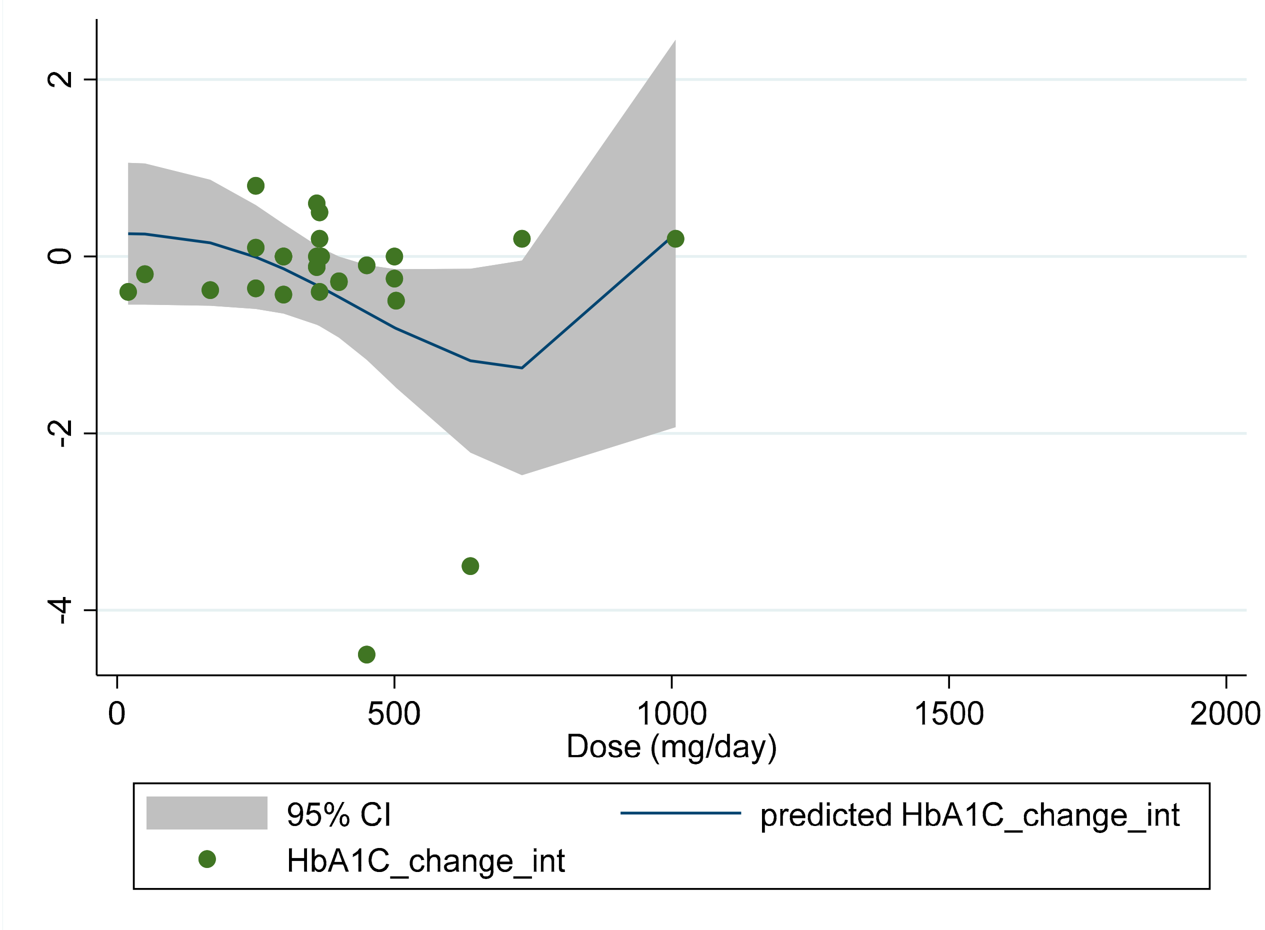

H) FI

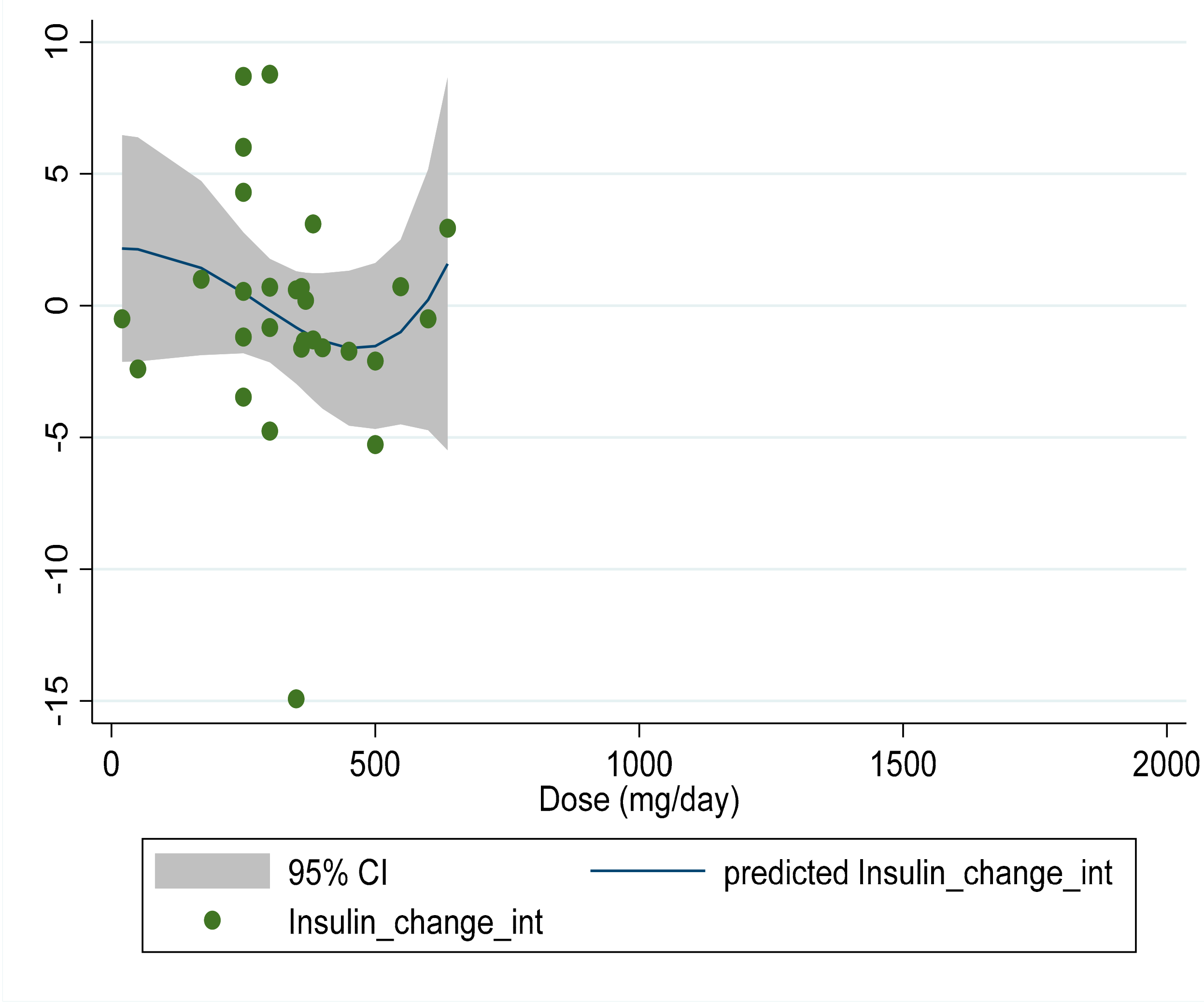

Figure S4. *Cont.*

I) HOMA-IR

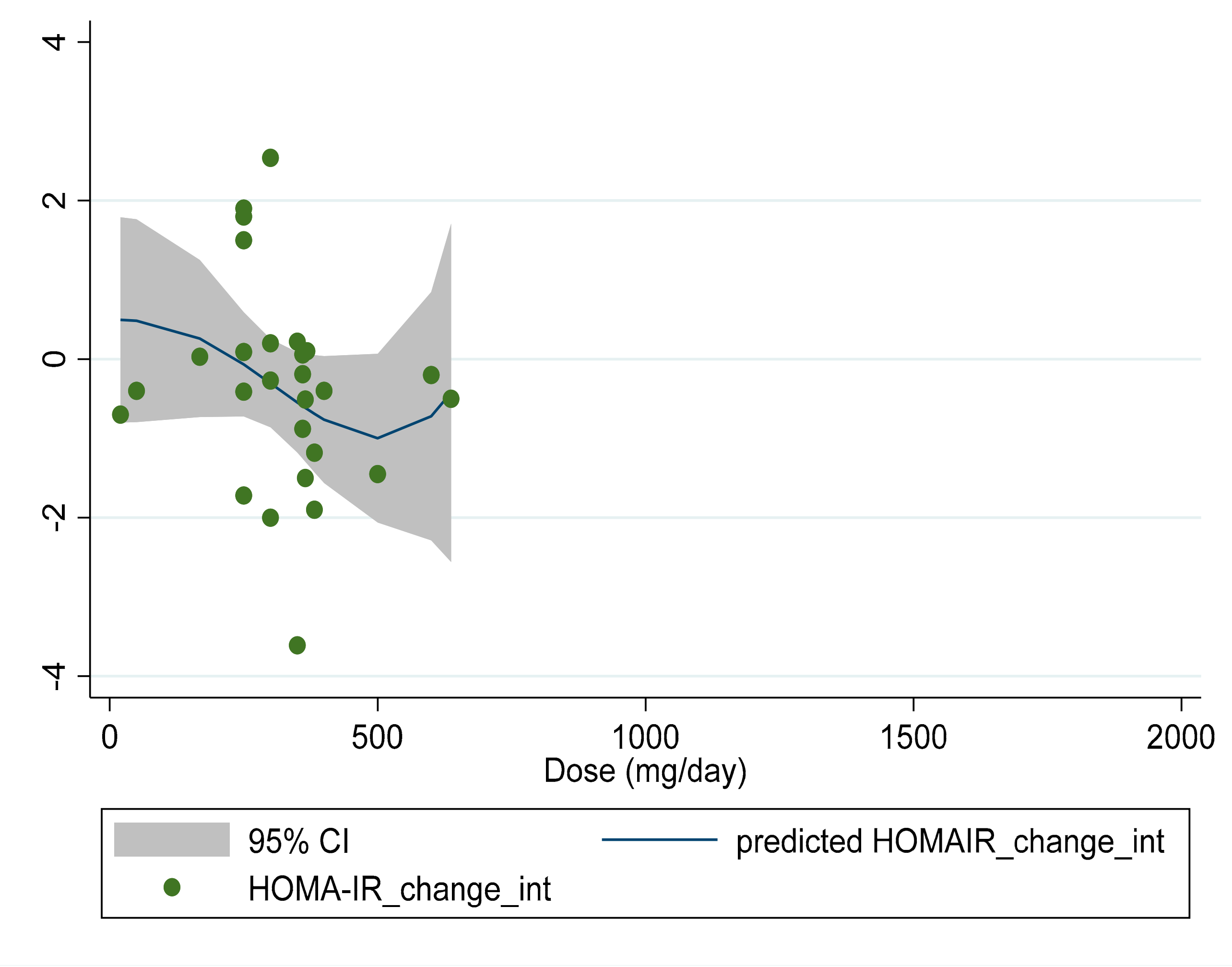

J) TG

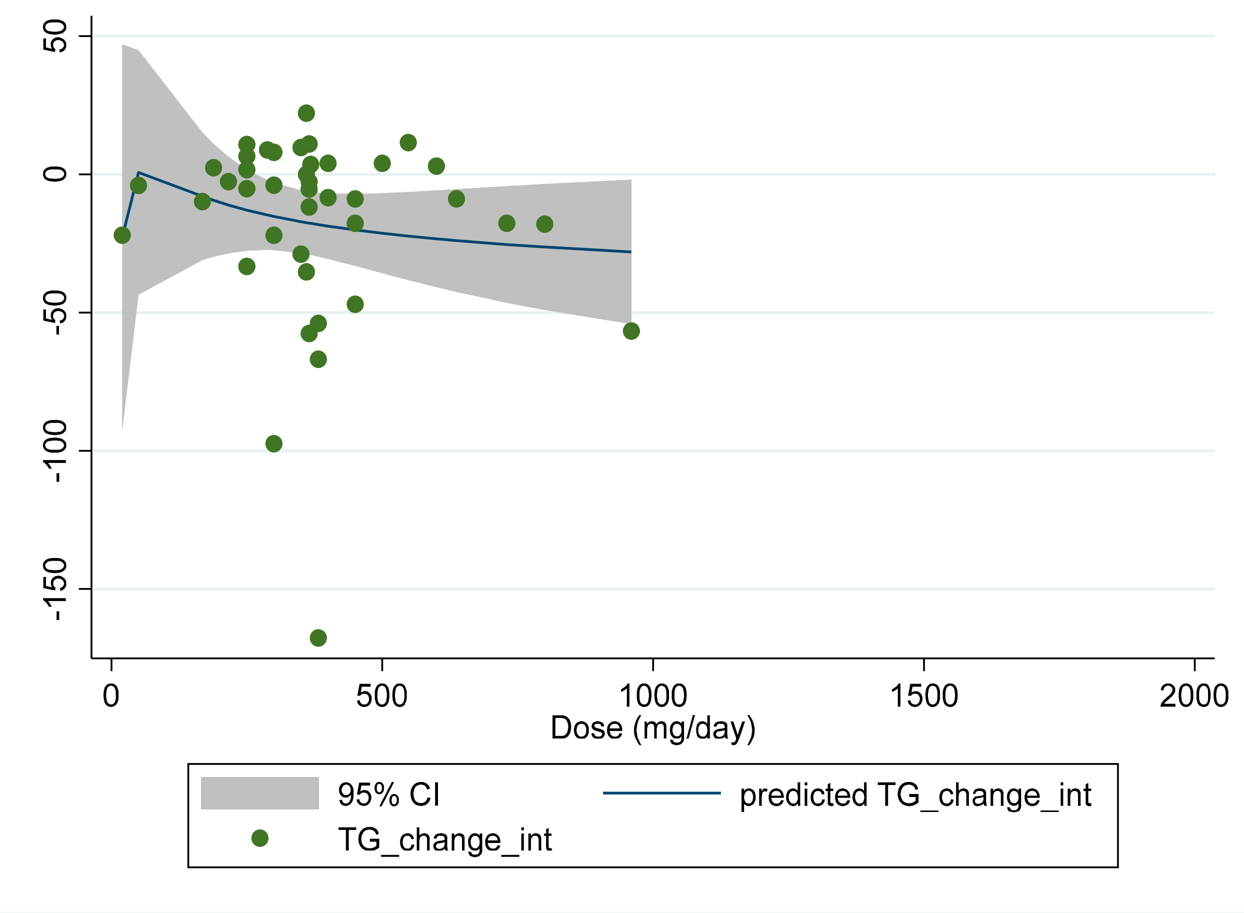

Figure S4. *Cont.*

K) TC

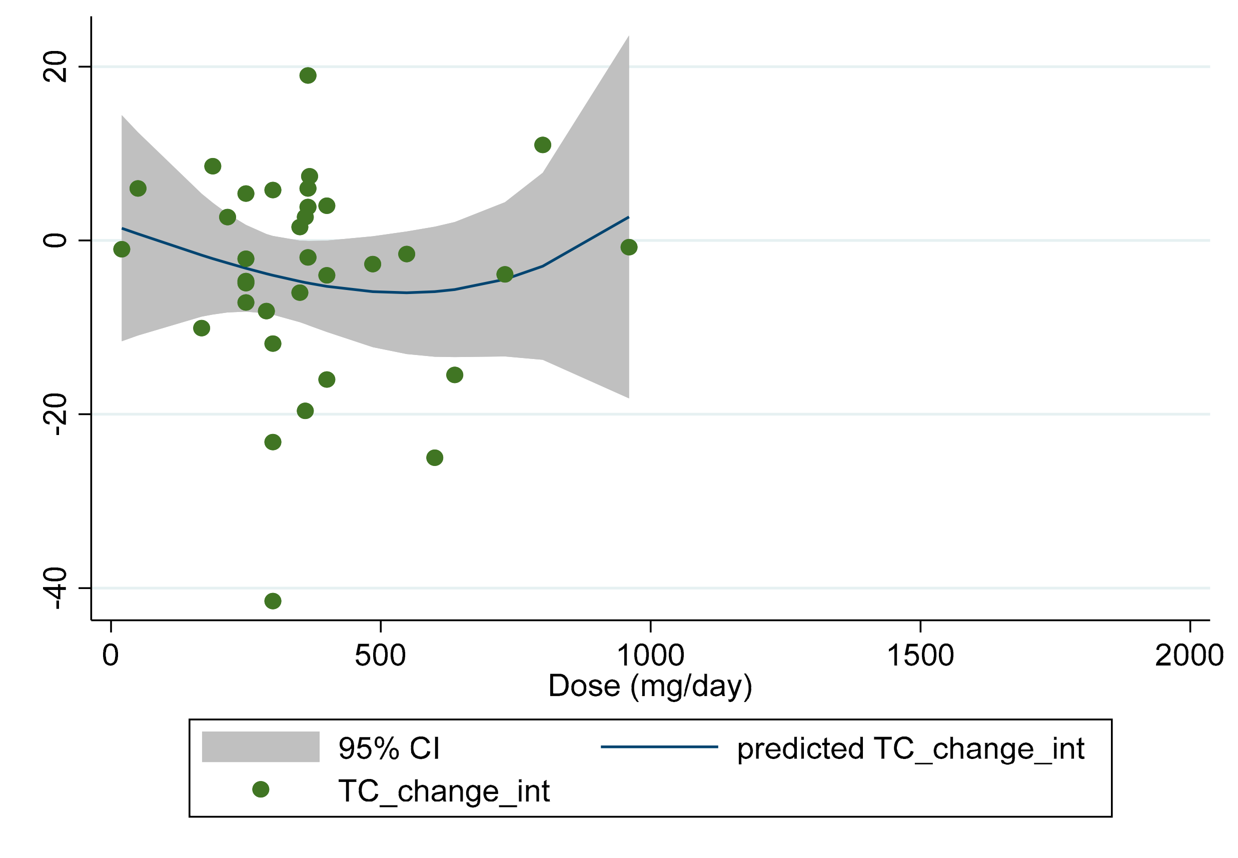

L) LDL-C

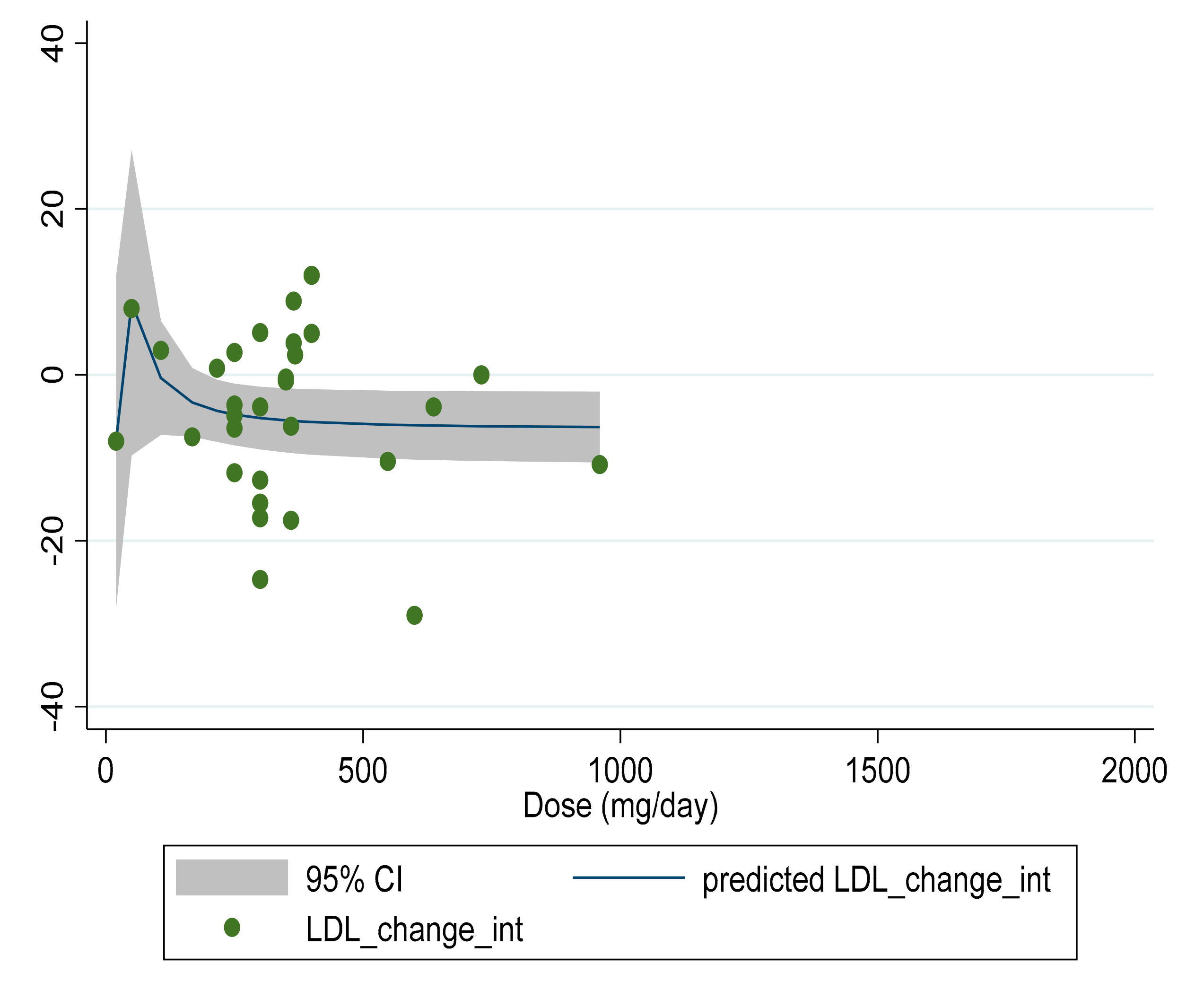

Figure S4. *Cont.*

M) HDL-C

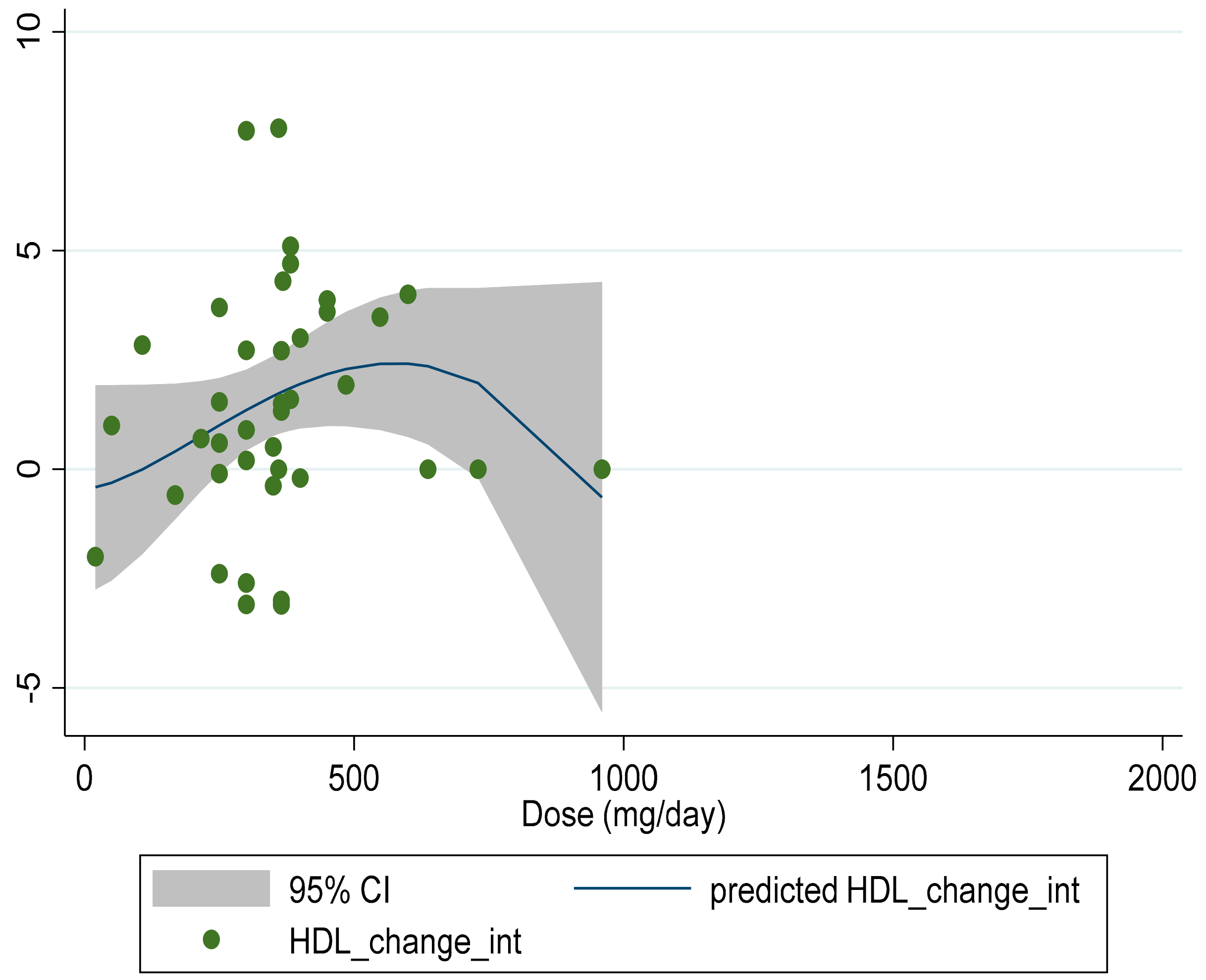

N) SBP

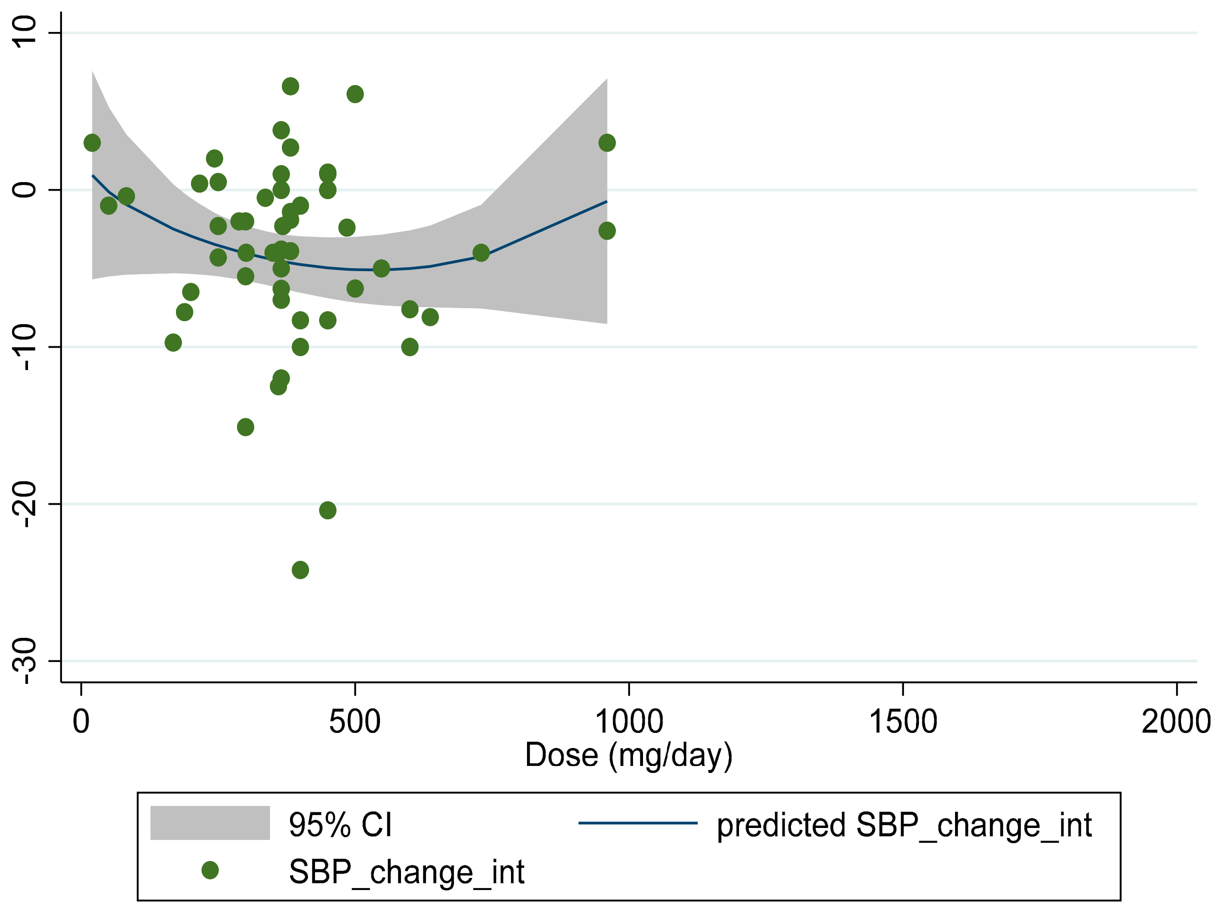

Figure S4. *Cont.*

O) DBP

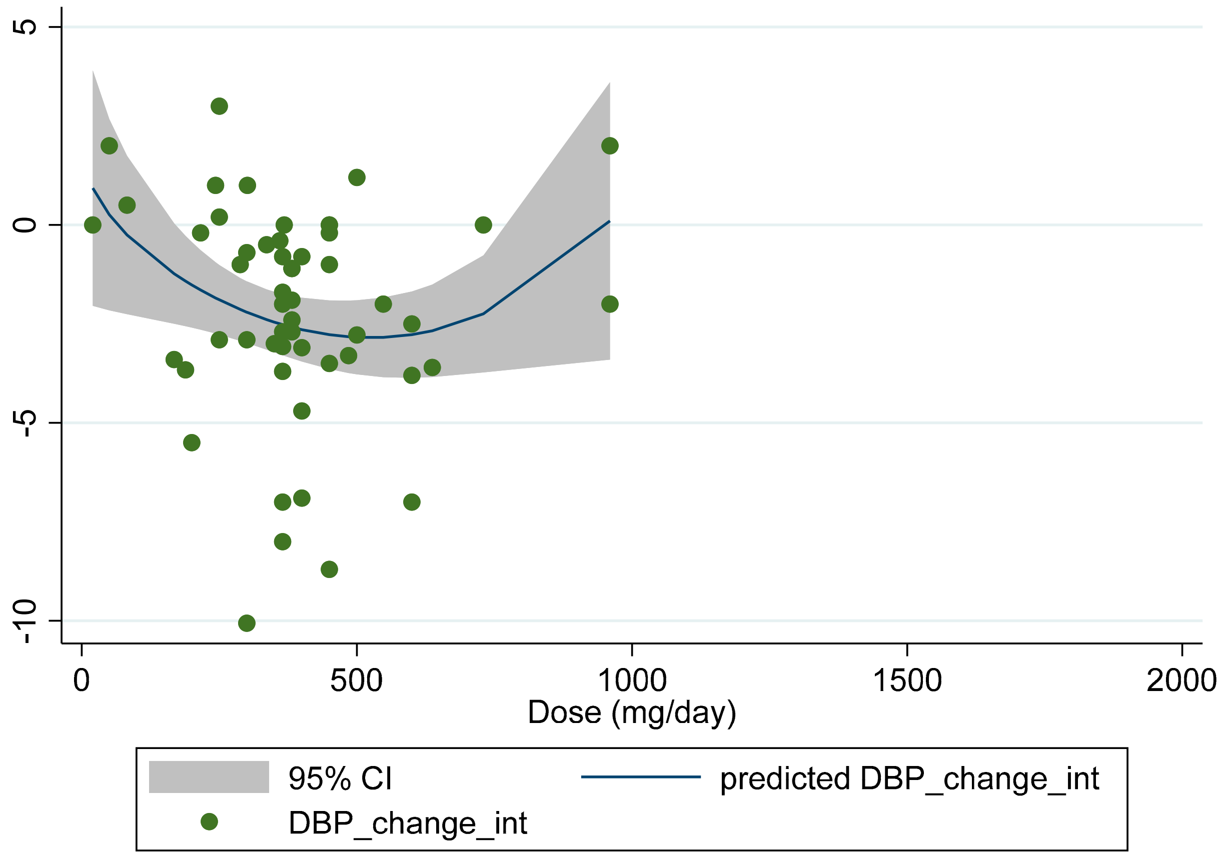

P) CRP

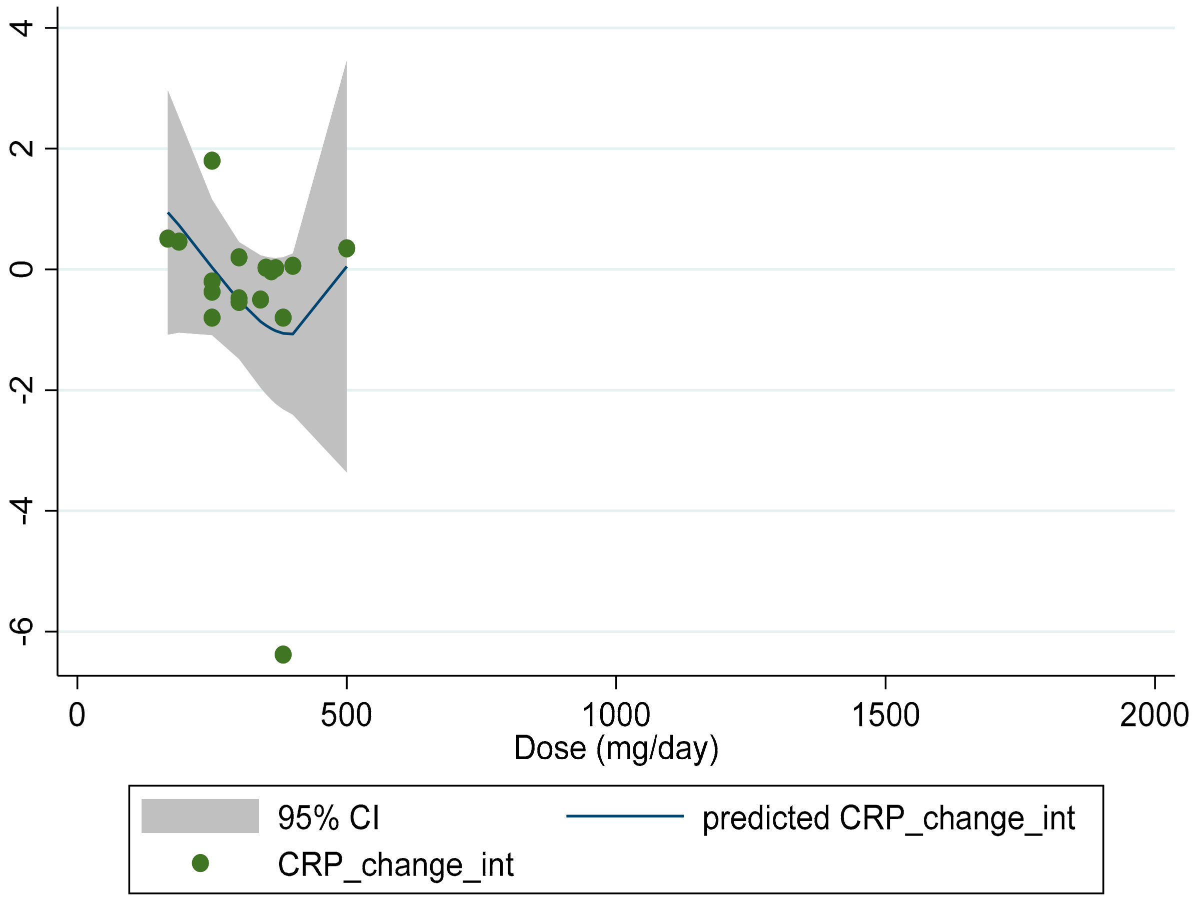

Figure S4. *Cont.*

Q) IL-6

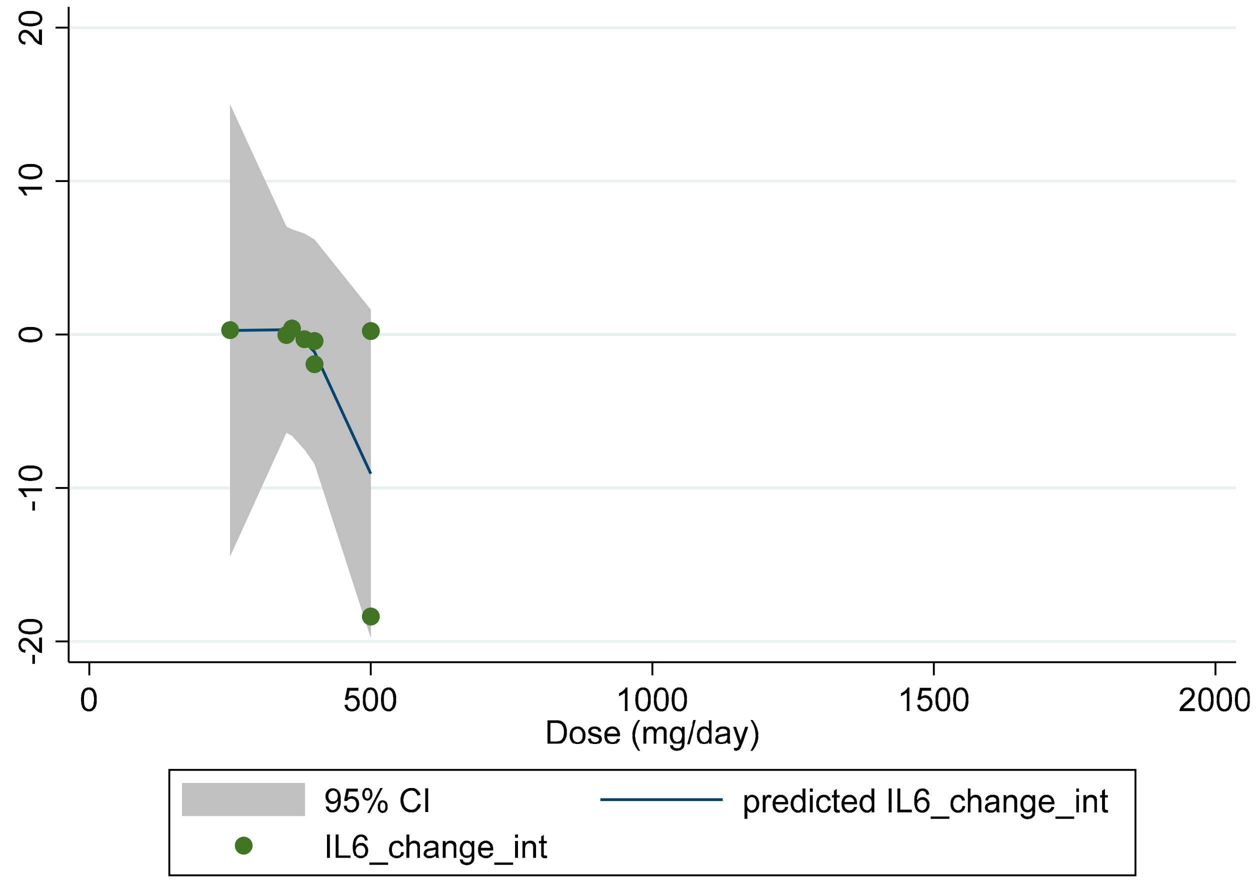

R) TNF-α

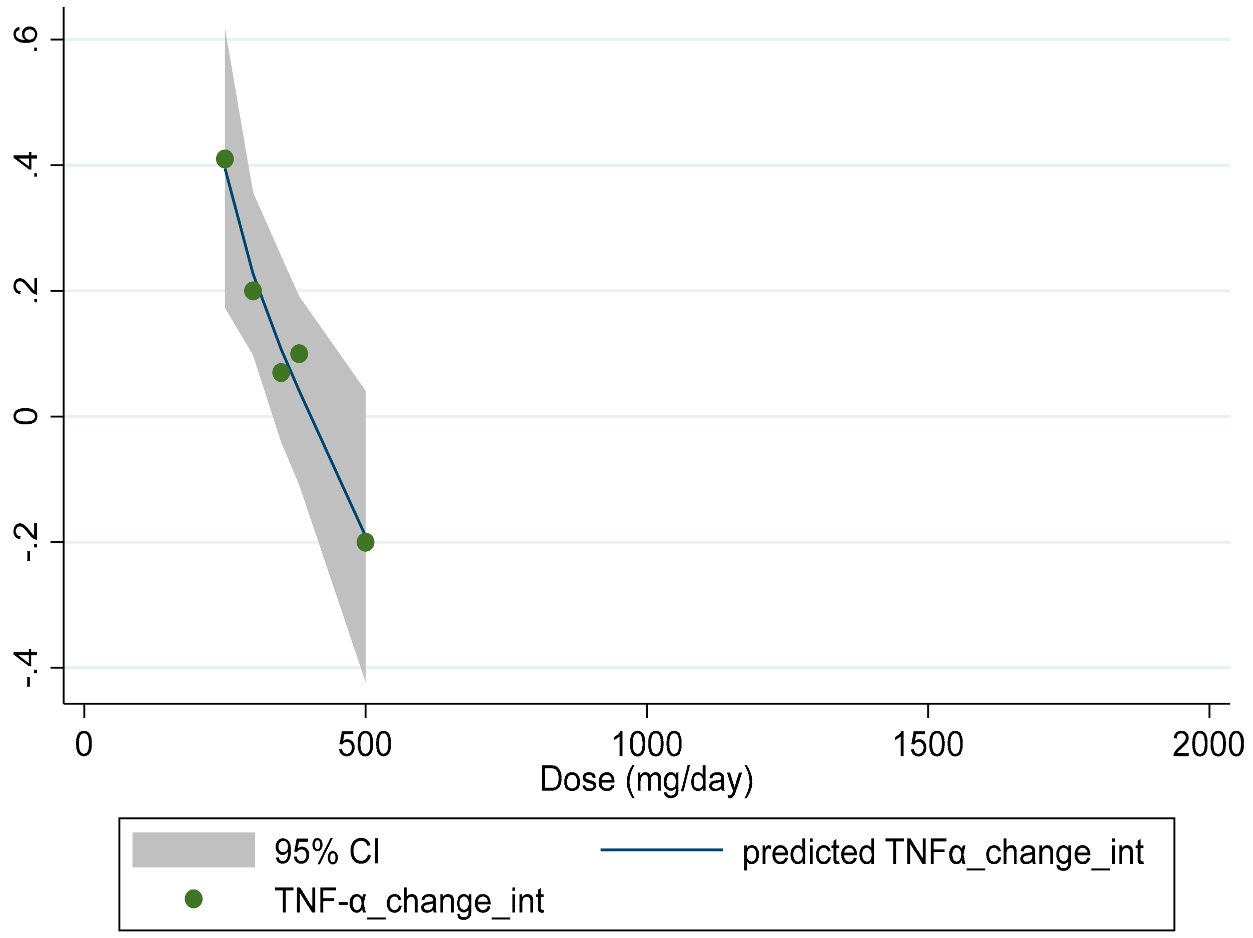

Figure S4. *Cont.*

S) AST

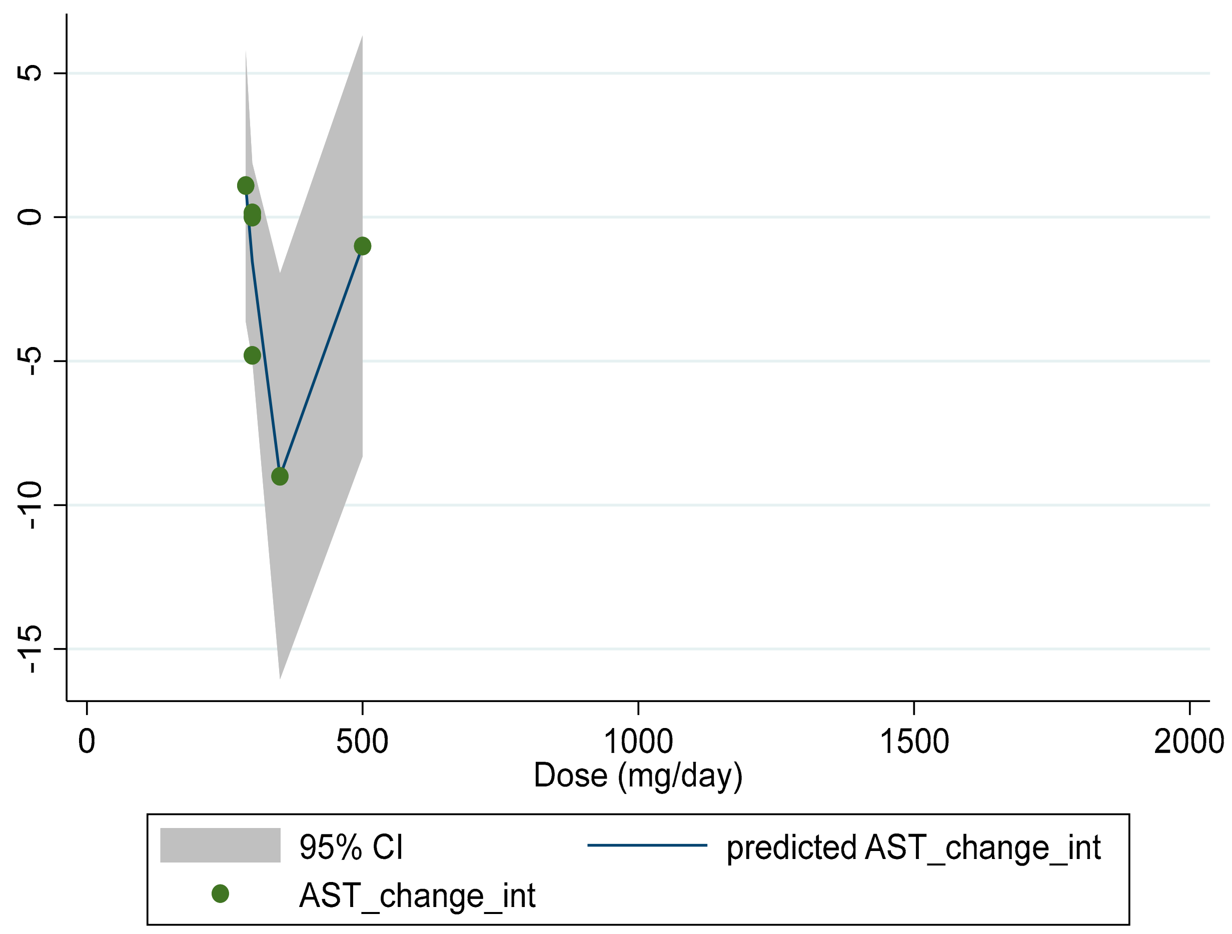

T) ALT

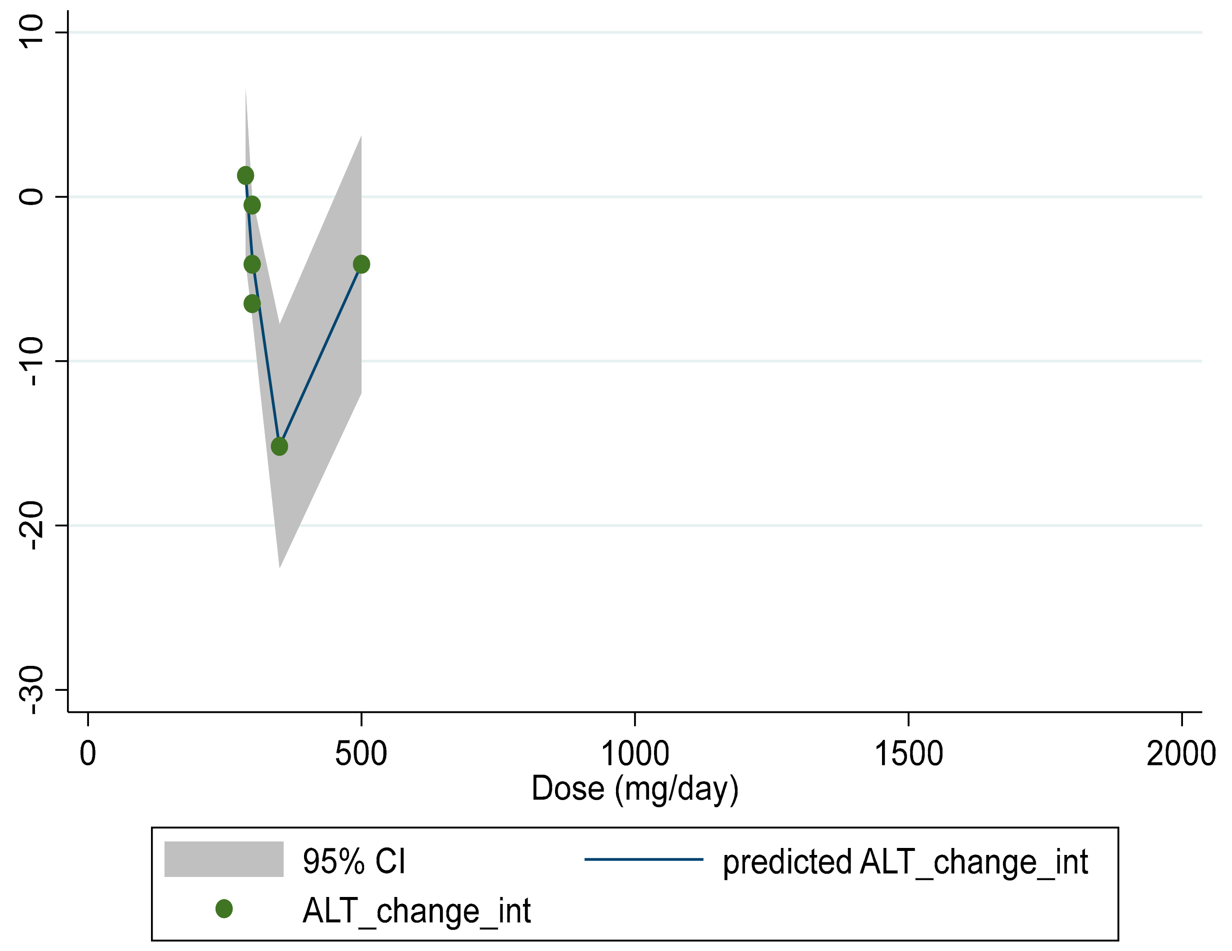

Figure S4. *Cont.*

U) Cr

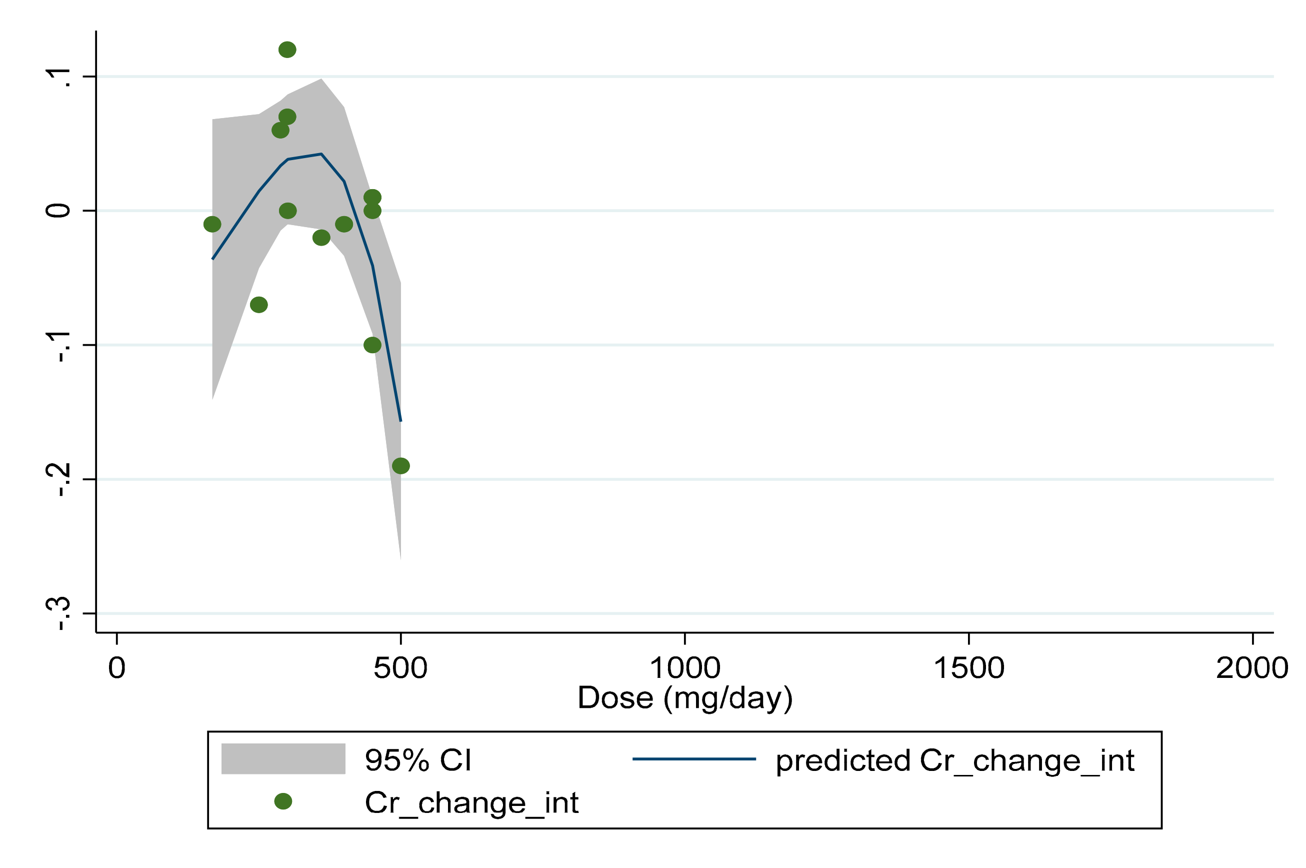

Figure S4. Non-linear dose-response association between dose (mg/day) of Mg supplementation and mean changes in cardiometabolic risk factors (CMRFs), including **(A)** BW (kg), **(B)** BMI (kg/m²), **(C)** WC (cm), **(D)** HC (cm), **(E)** BFP (%), **(F)** FBG (mg/dL), **(G)** HbA1c (%), **(H)** FI (µIU/mL), **(I)** HOMA-IR, **(J)** TG (mg/dL), **(K)** TC (mg/dL), **(L)** LDL-C (mg/dL), **(M)** HDL-C (mg/dL), **(N)** SBP (mmHg), **(O)** DBP (mmHg), **(P)** CRP (mg/L), **(Q)** IL-6 (pg/mL), **(R)** TNF-α (pg/mL), **(S)** AST (IU/L), **(T)** ALT (IU/L), and **(U)** Cr (mg/dL).

A) BW

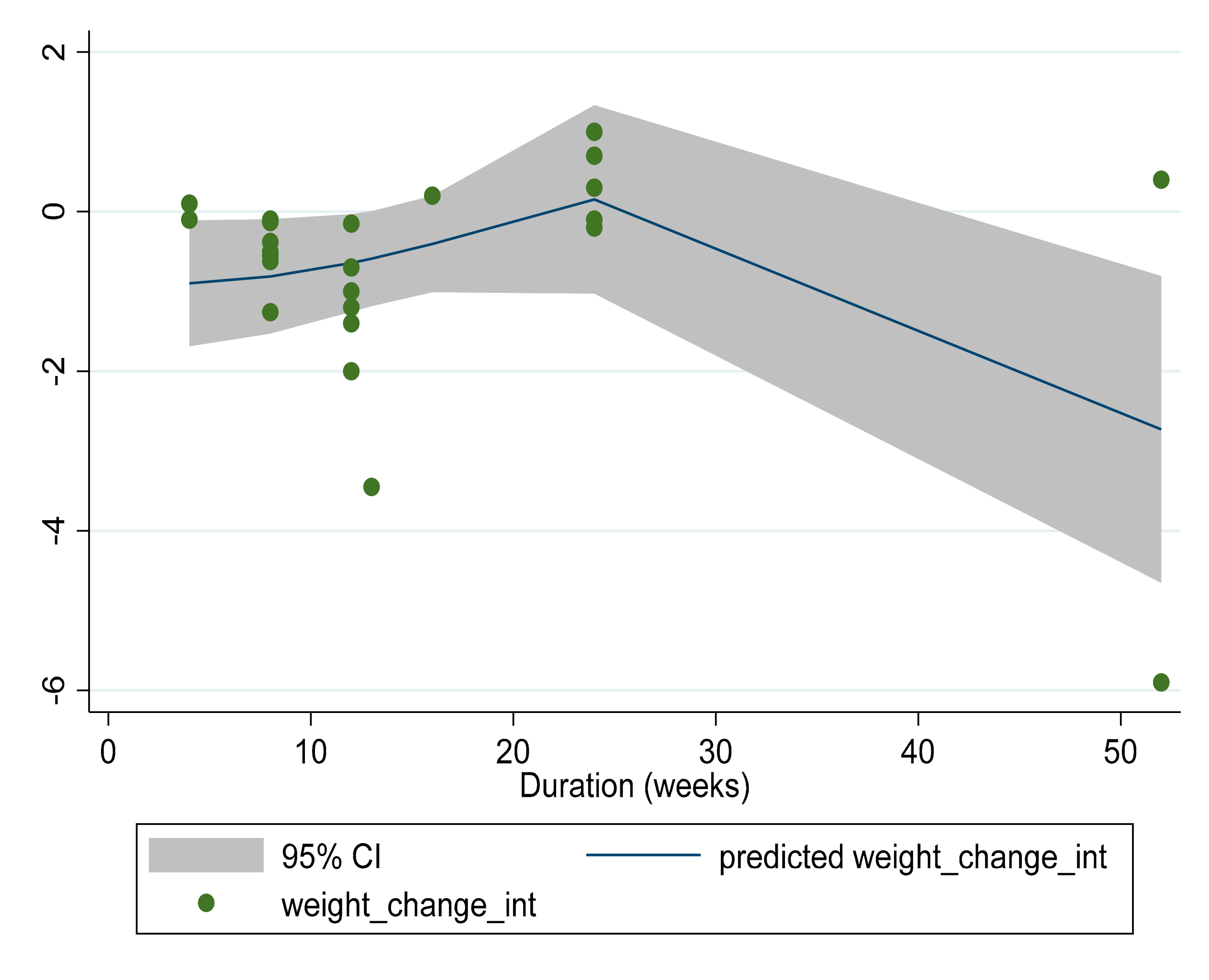

B) BMI

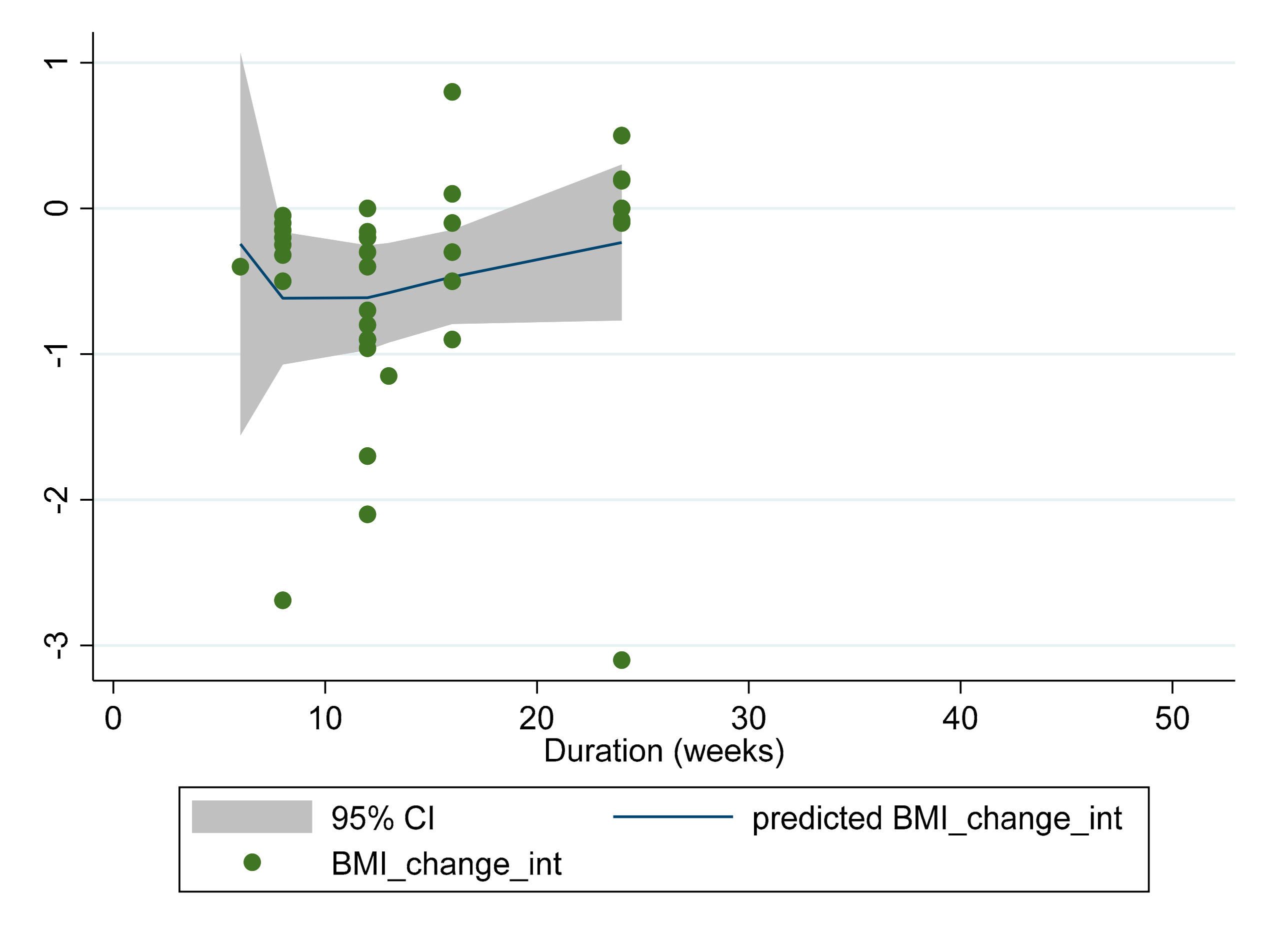

Figure S5. *Cont.*

C) WC

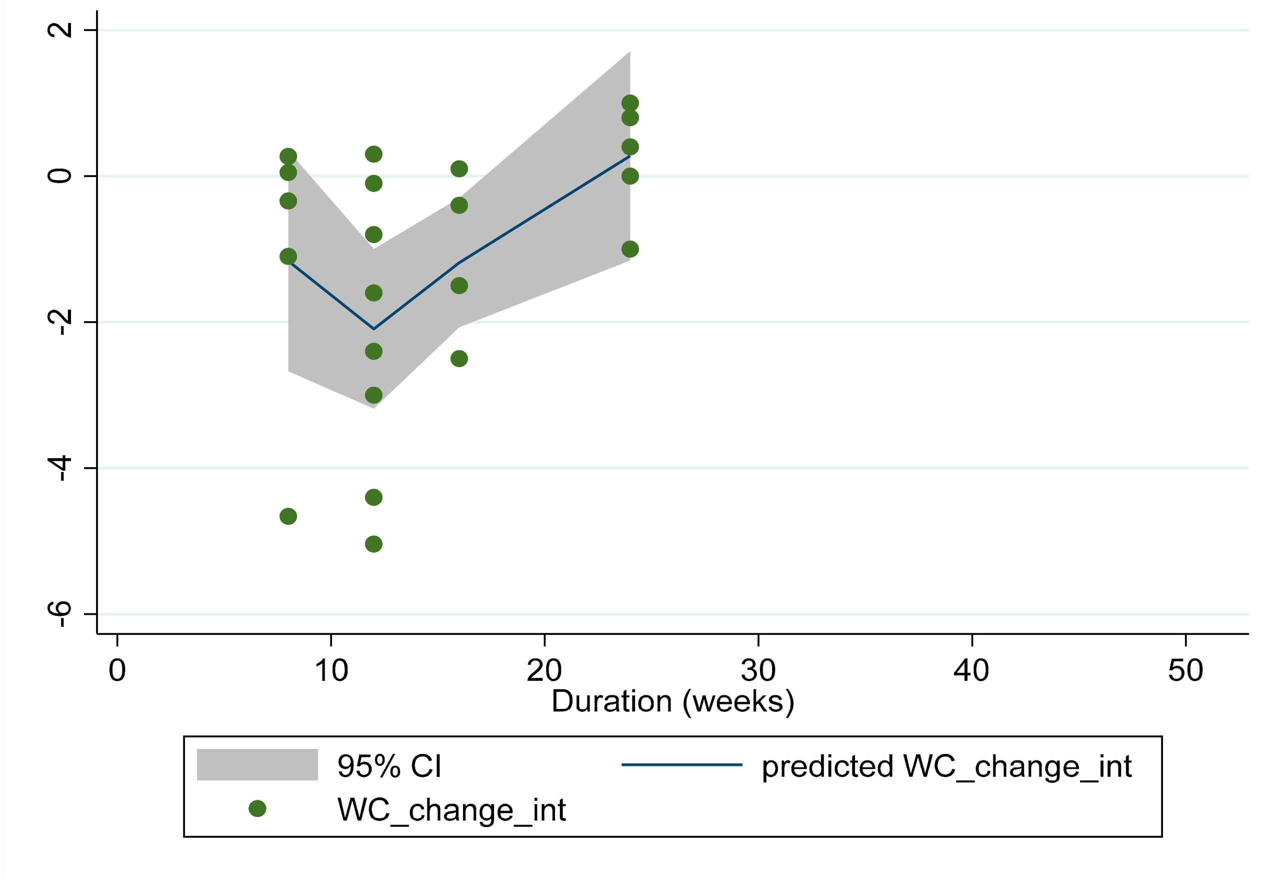

D) HC

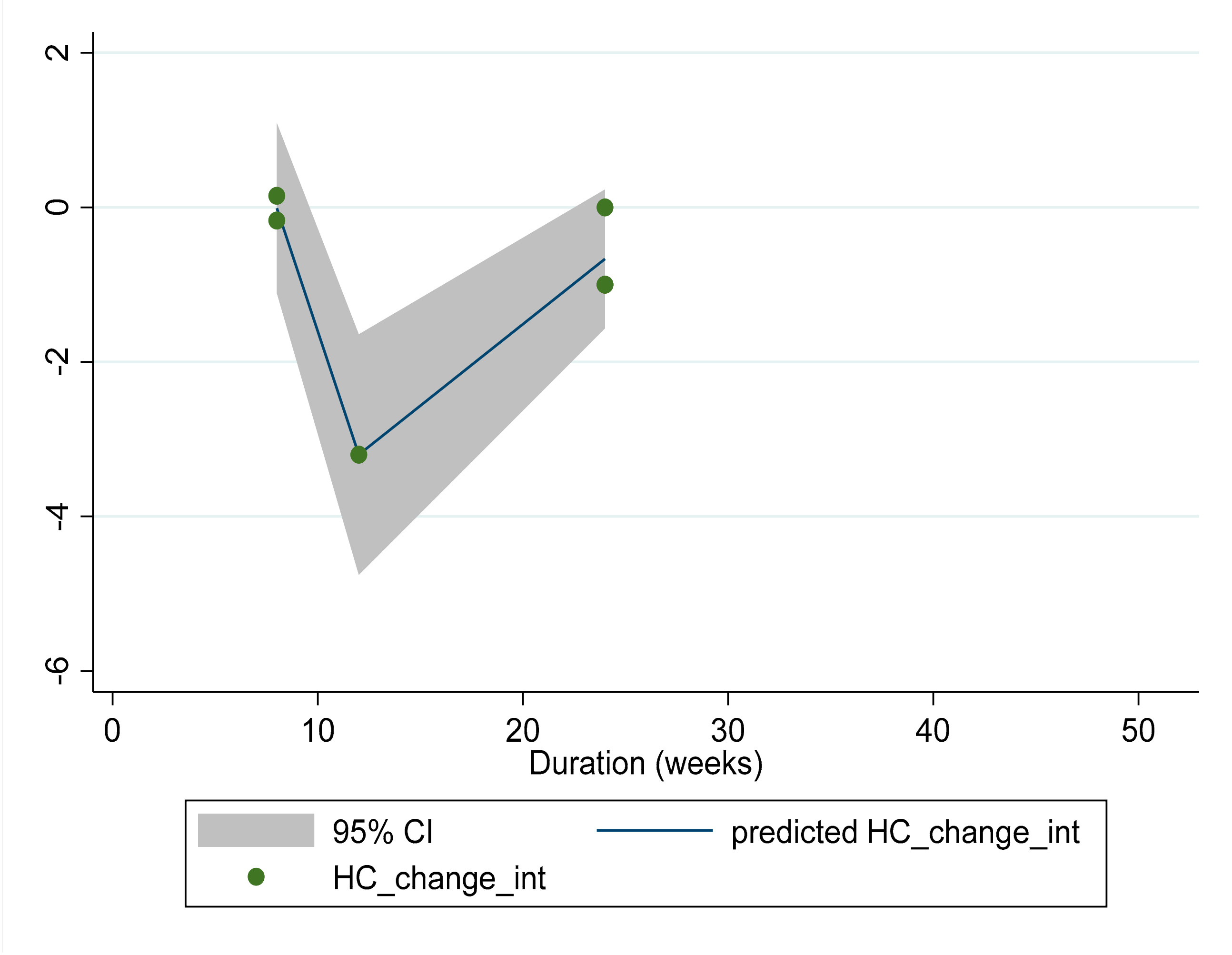

Figure S5. *Cont.*

E) BFP

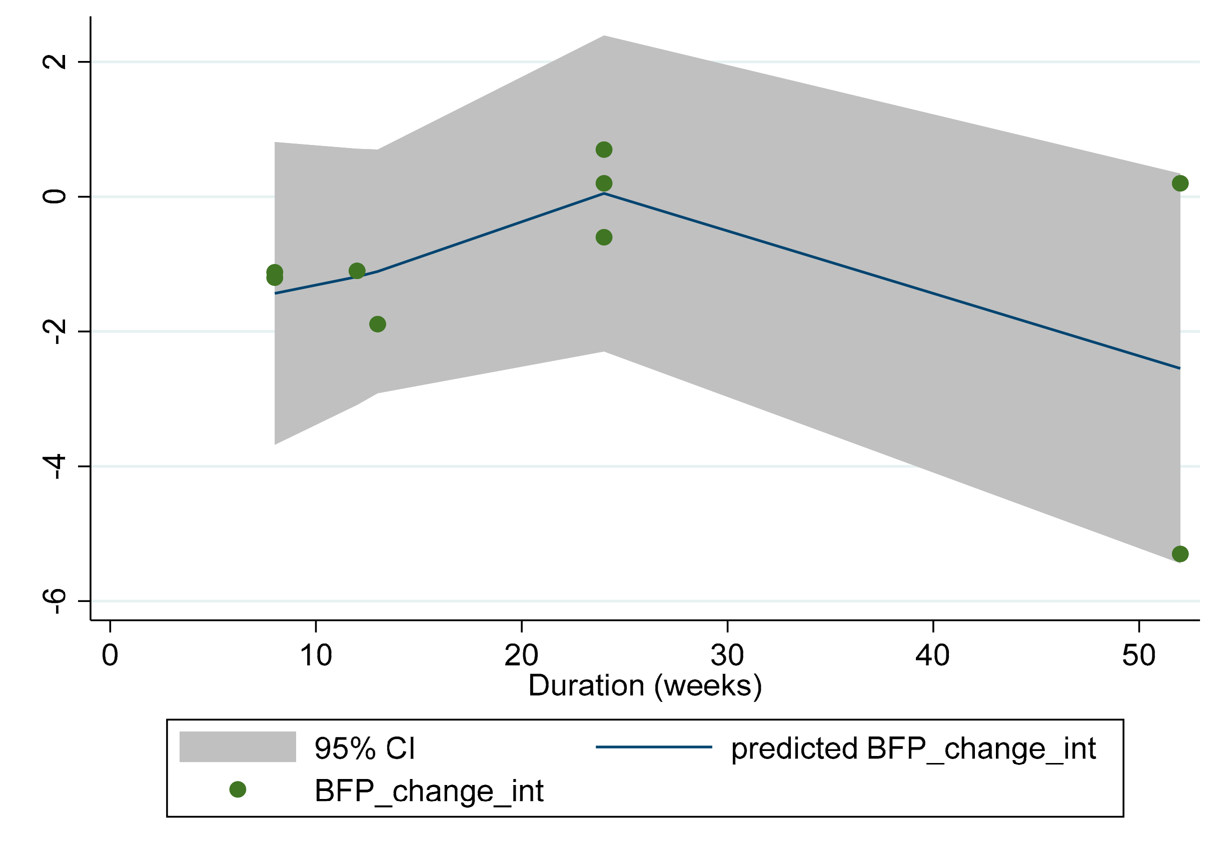

F) FBG

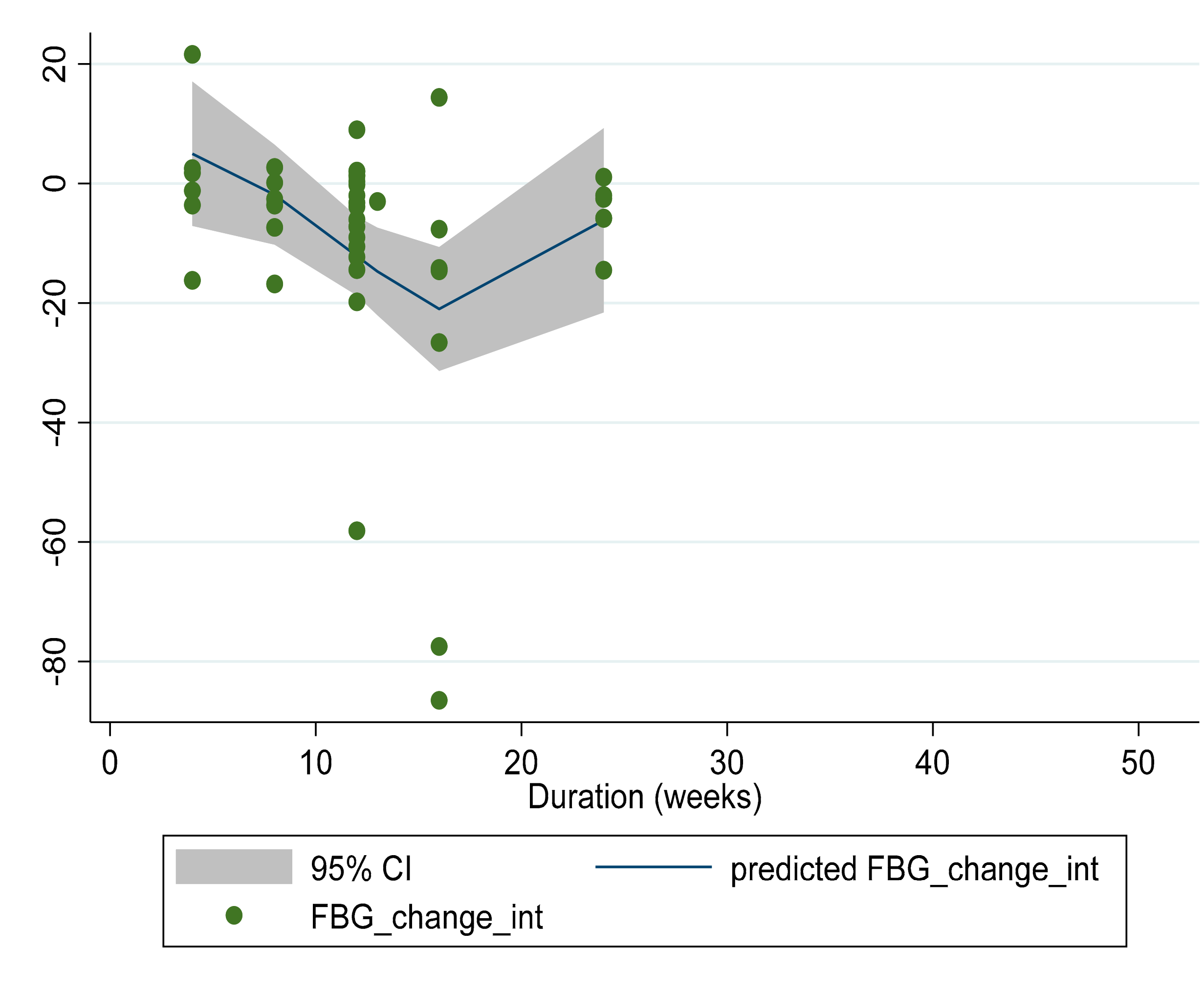

Figure S5. *Cont.*

G) HbA1c

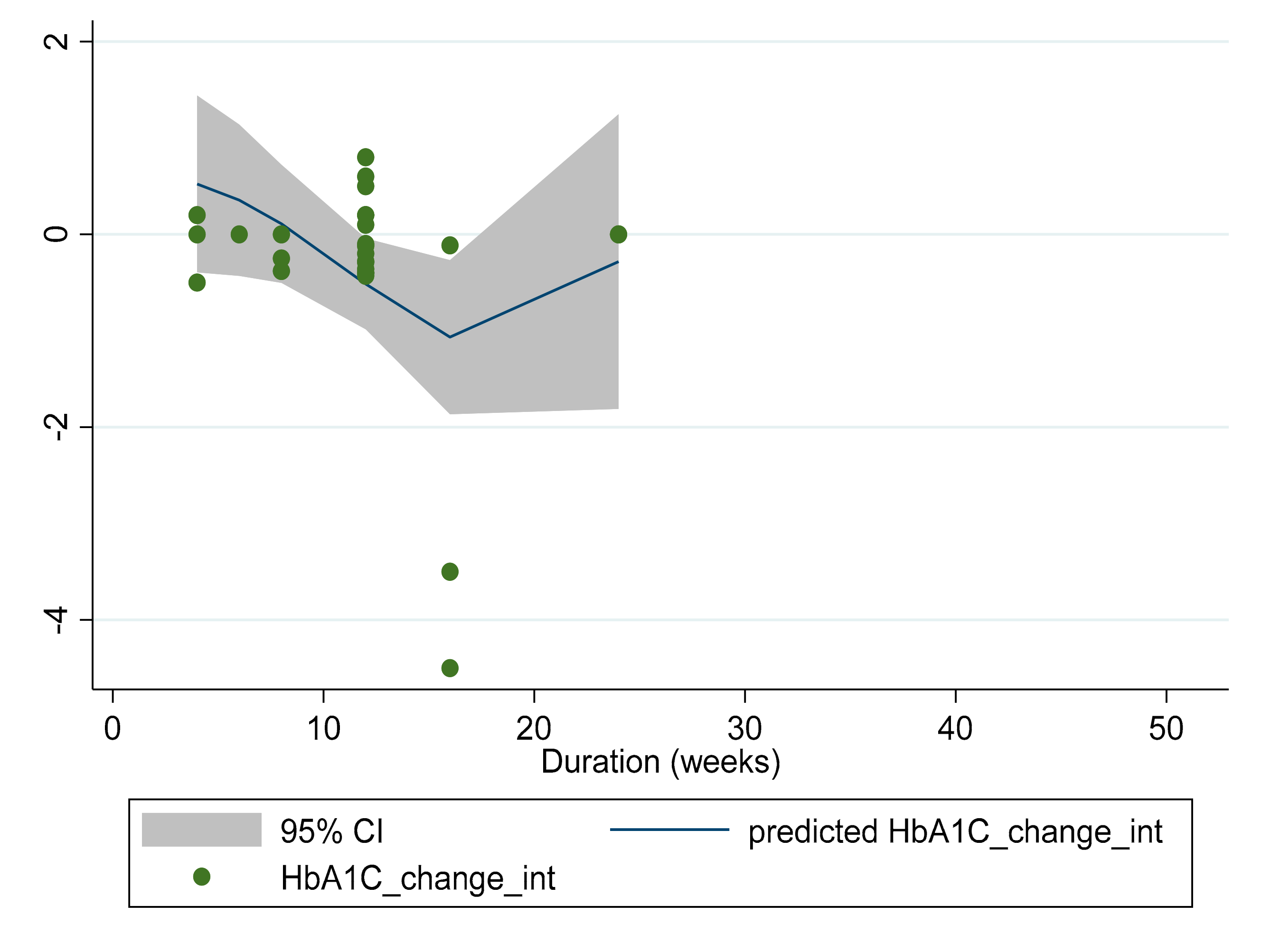

H) FI

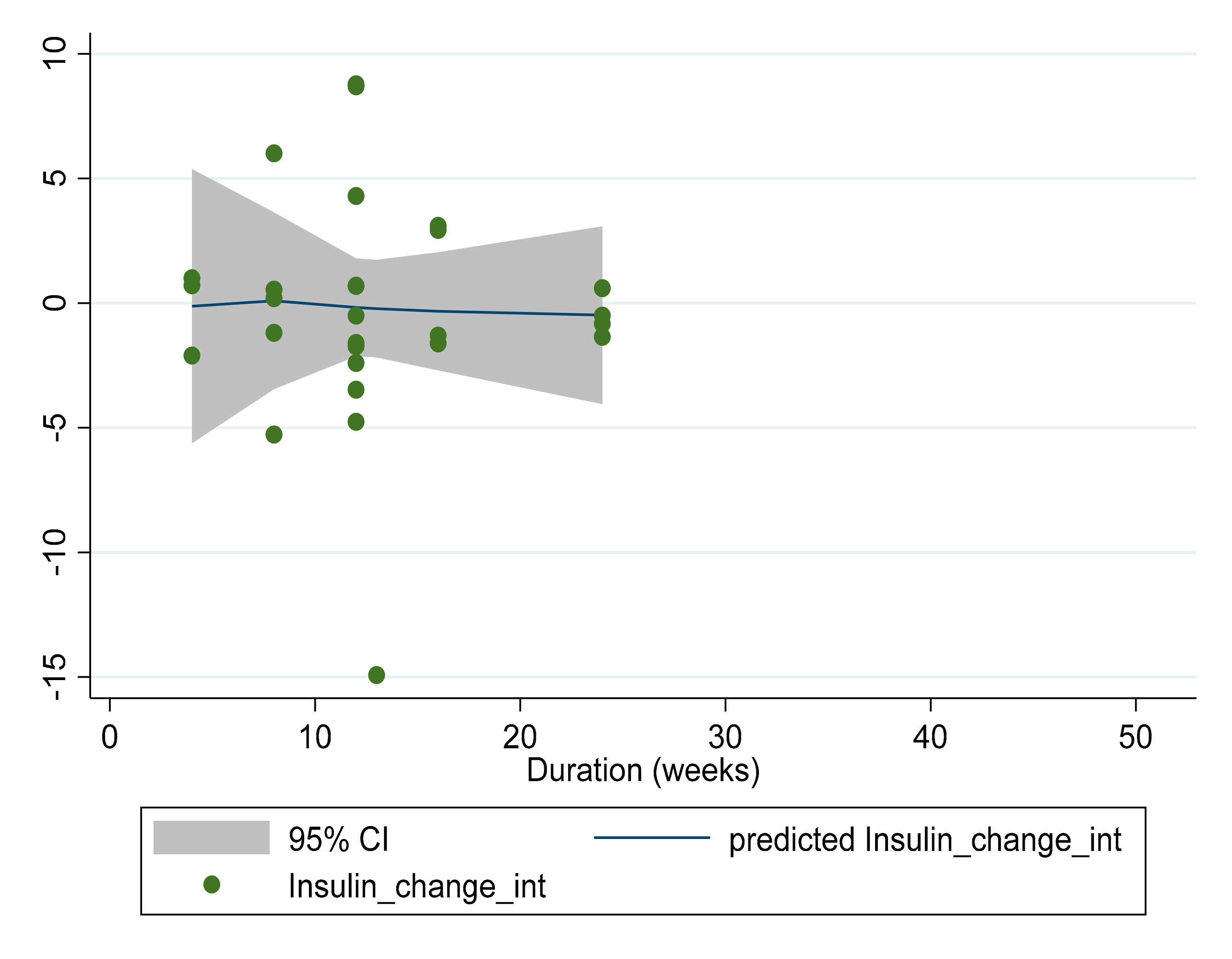

Figure S5. *Cont.*

I) HOMA-IR

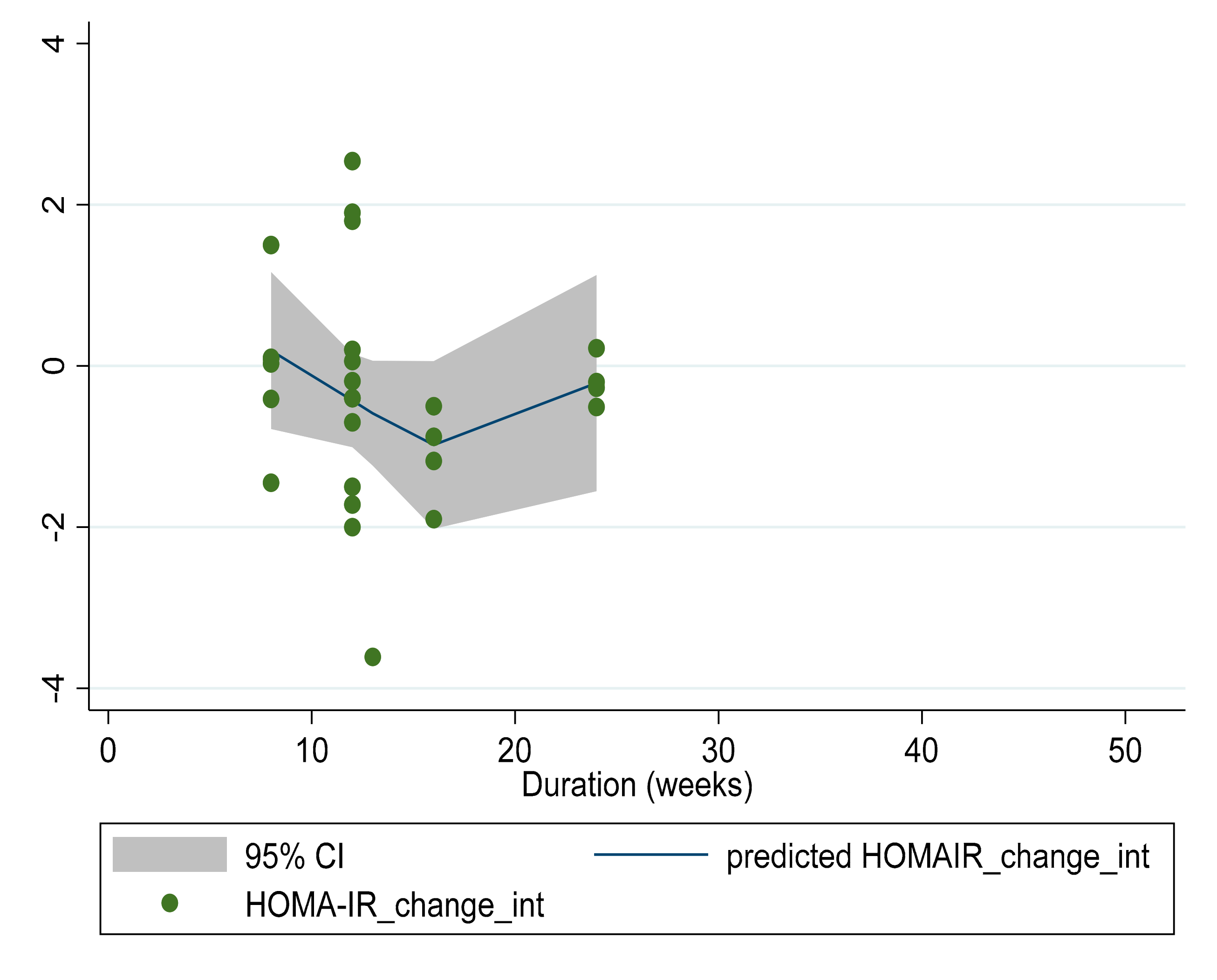

J) TG

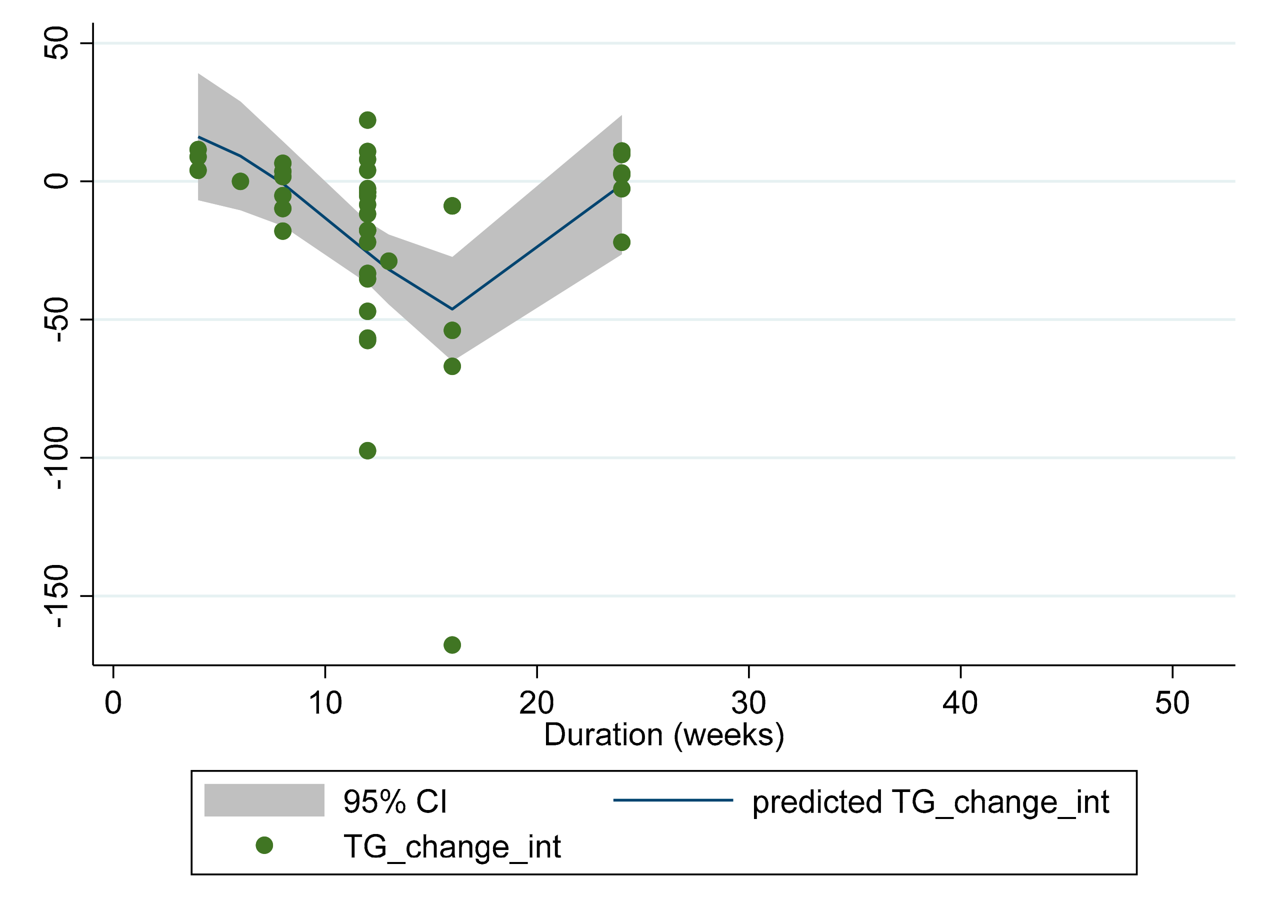

Figure S5. *Cont.*

K) TC

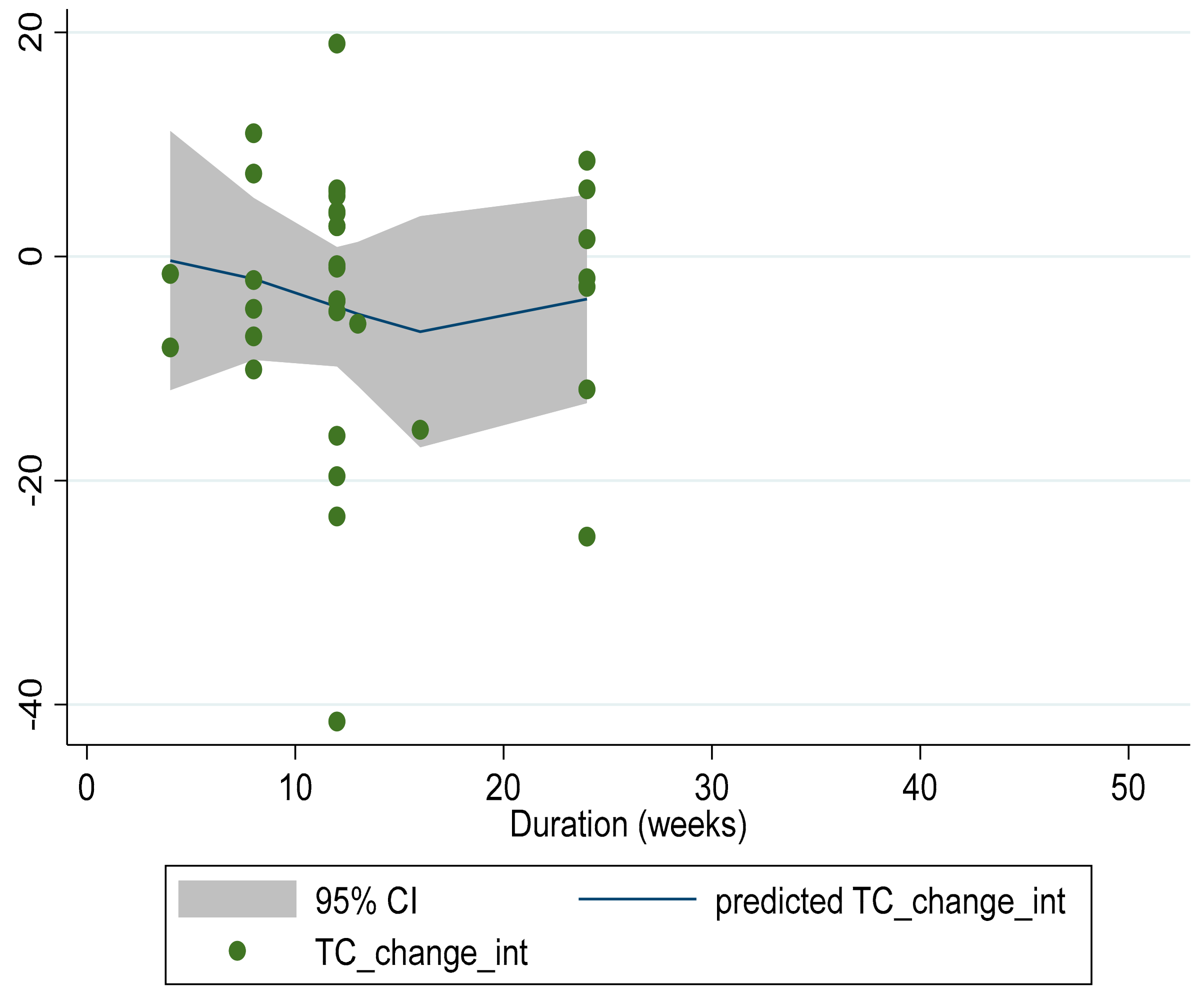

L) LDL-C

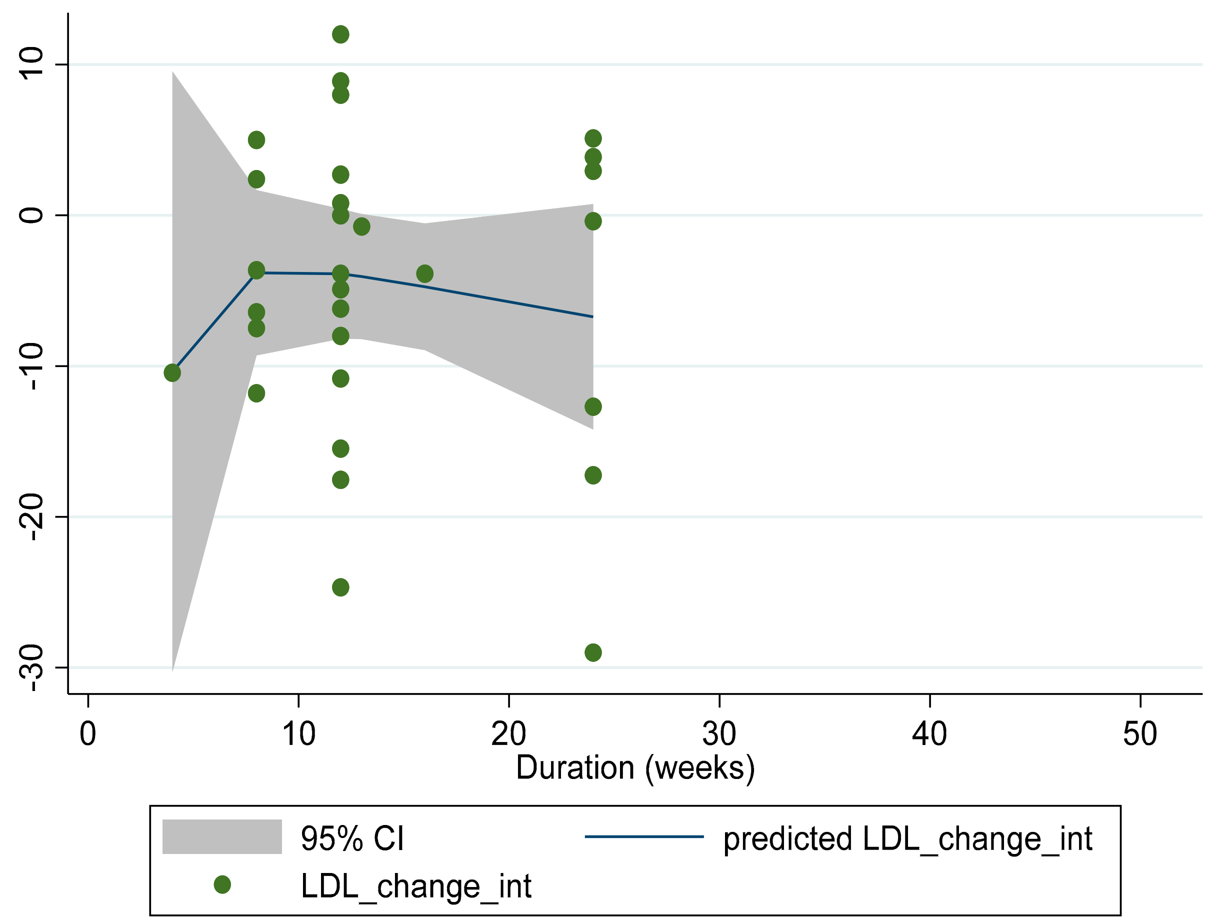

Figure S5. *Cont.*

M) HDL-C

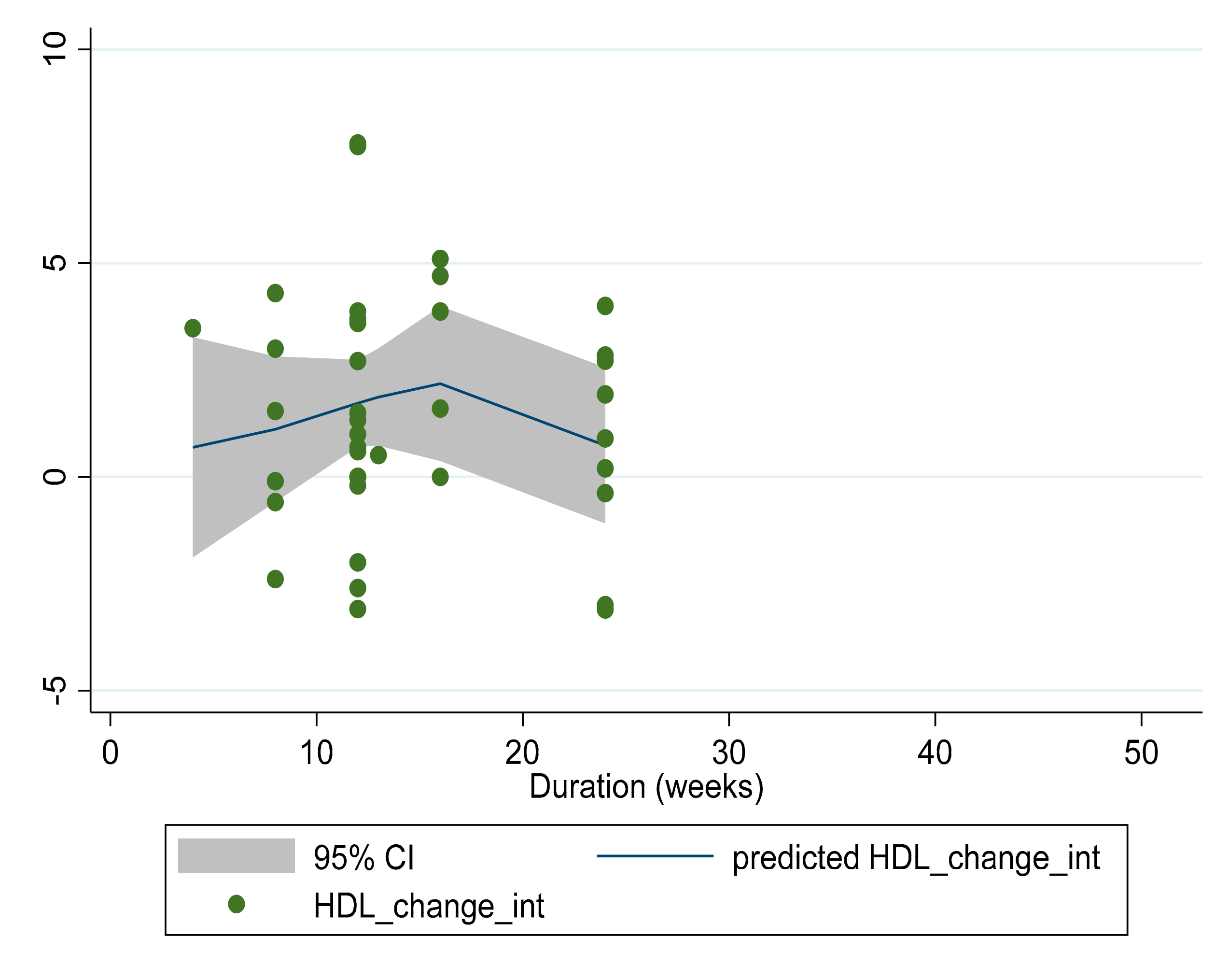

N) SBP

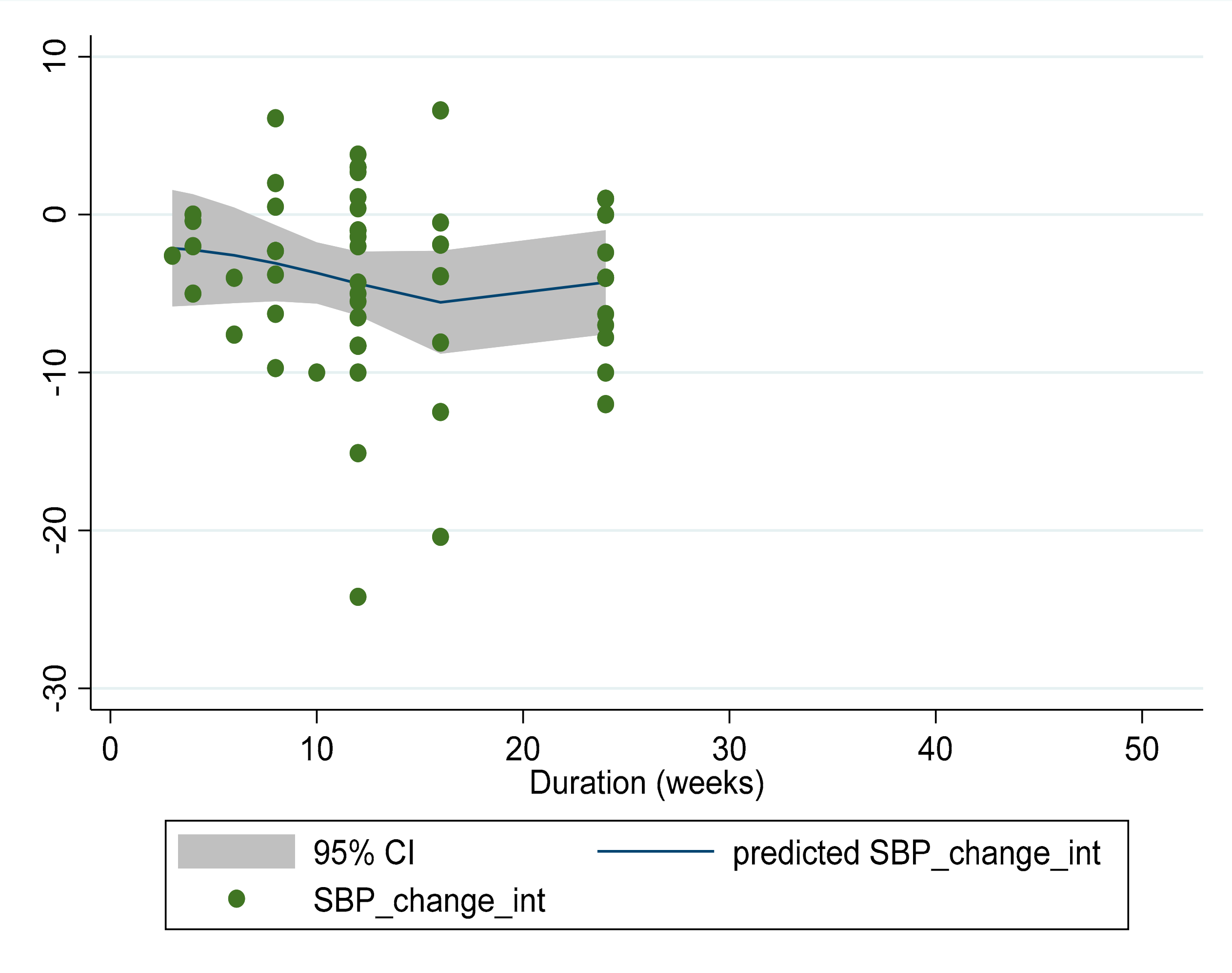

Figure S5. *Cont.*

O) DBP

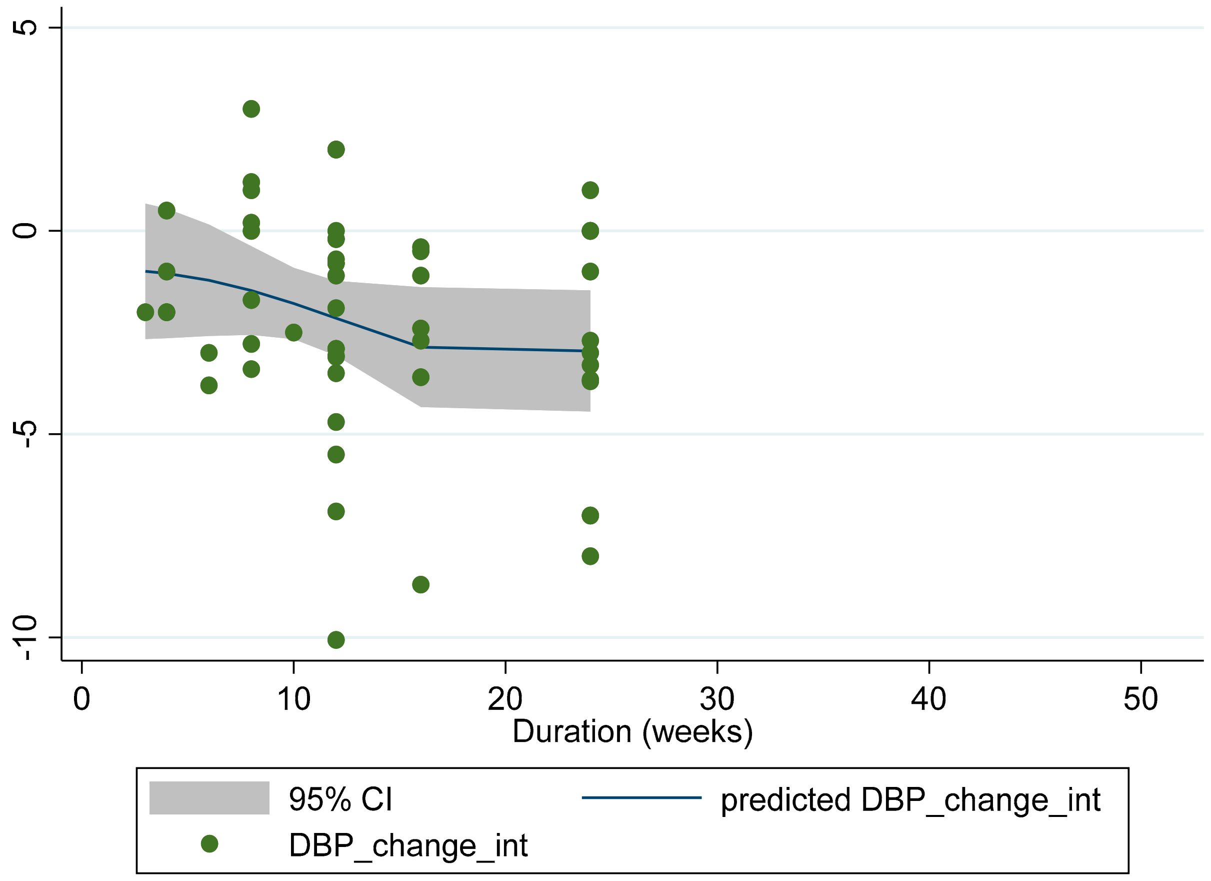

P) CRP

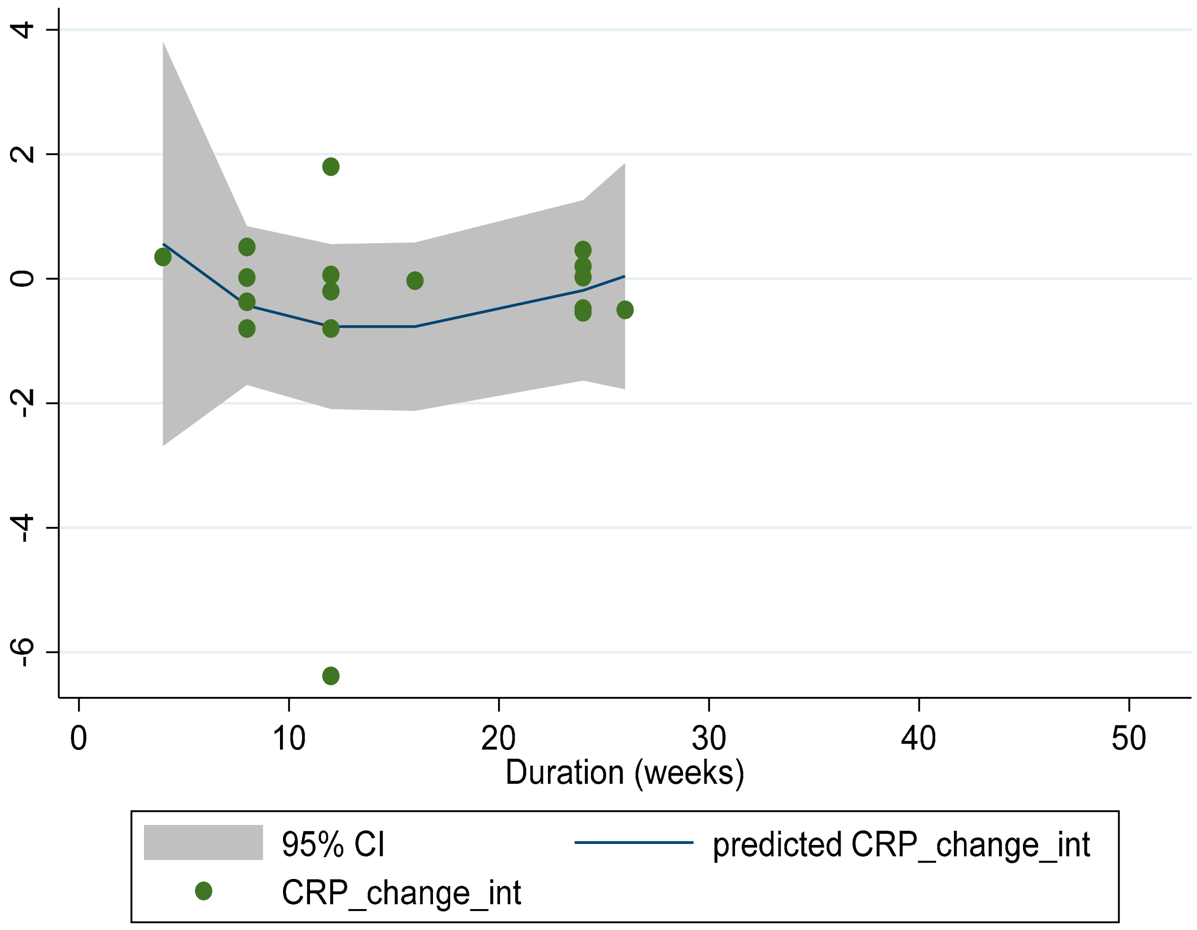

Figure S5. *Cont.*

Q) IL-6

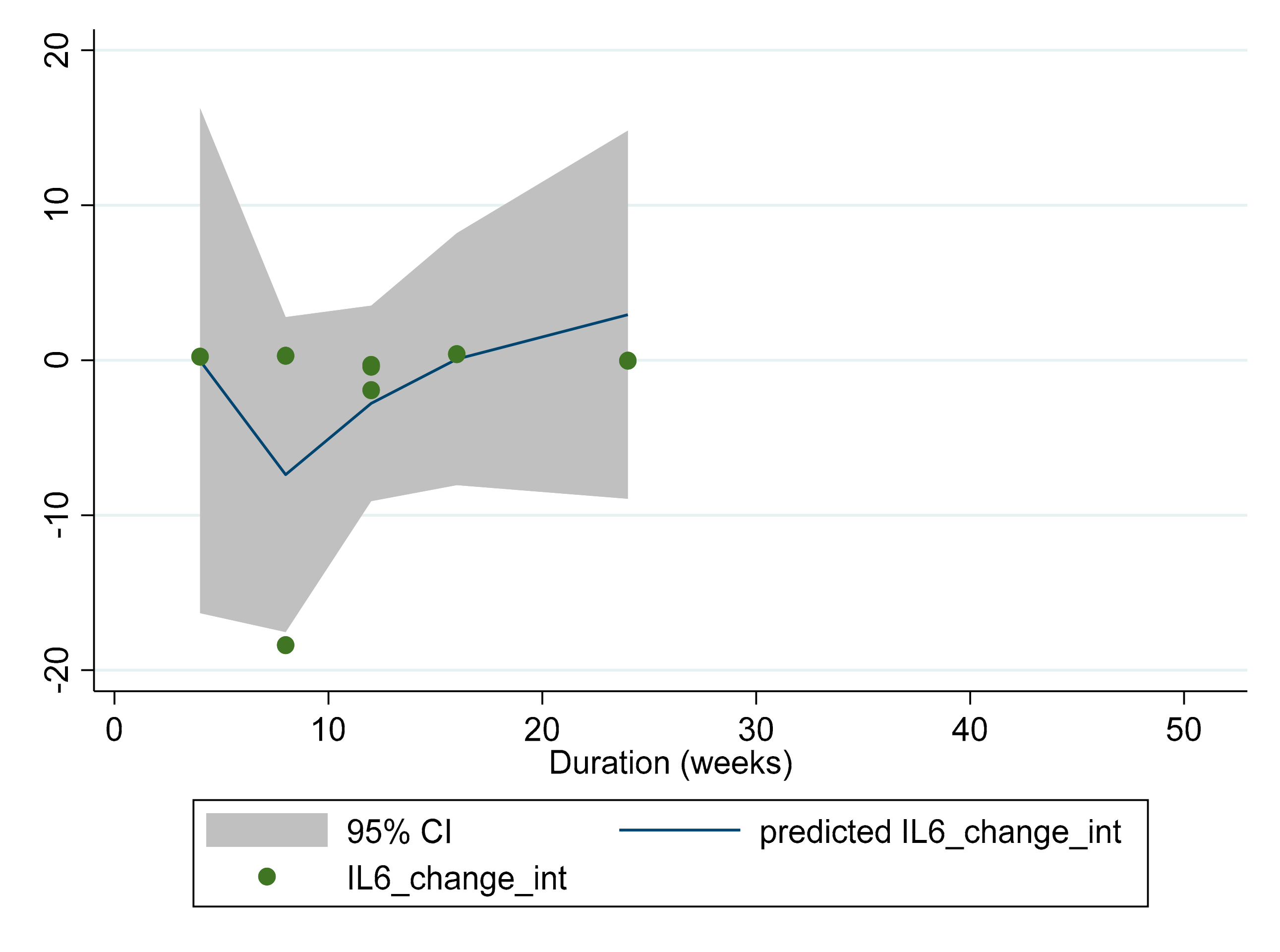

R) TNF-α

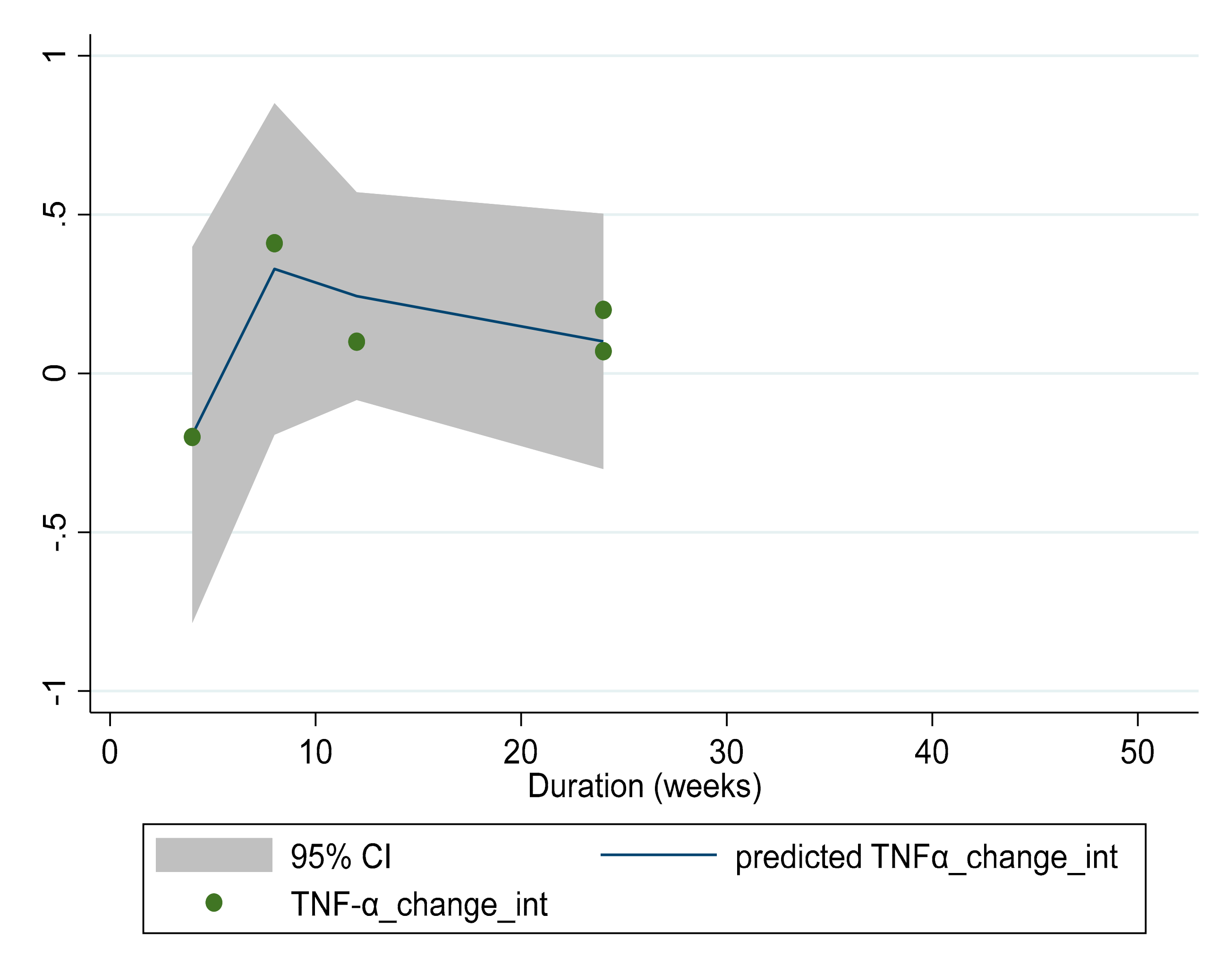

Figure S5. *Cont.*

S) AST

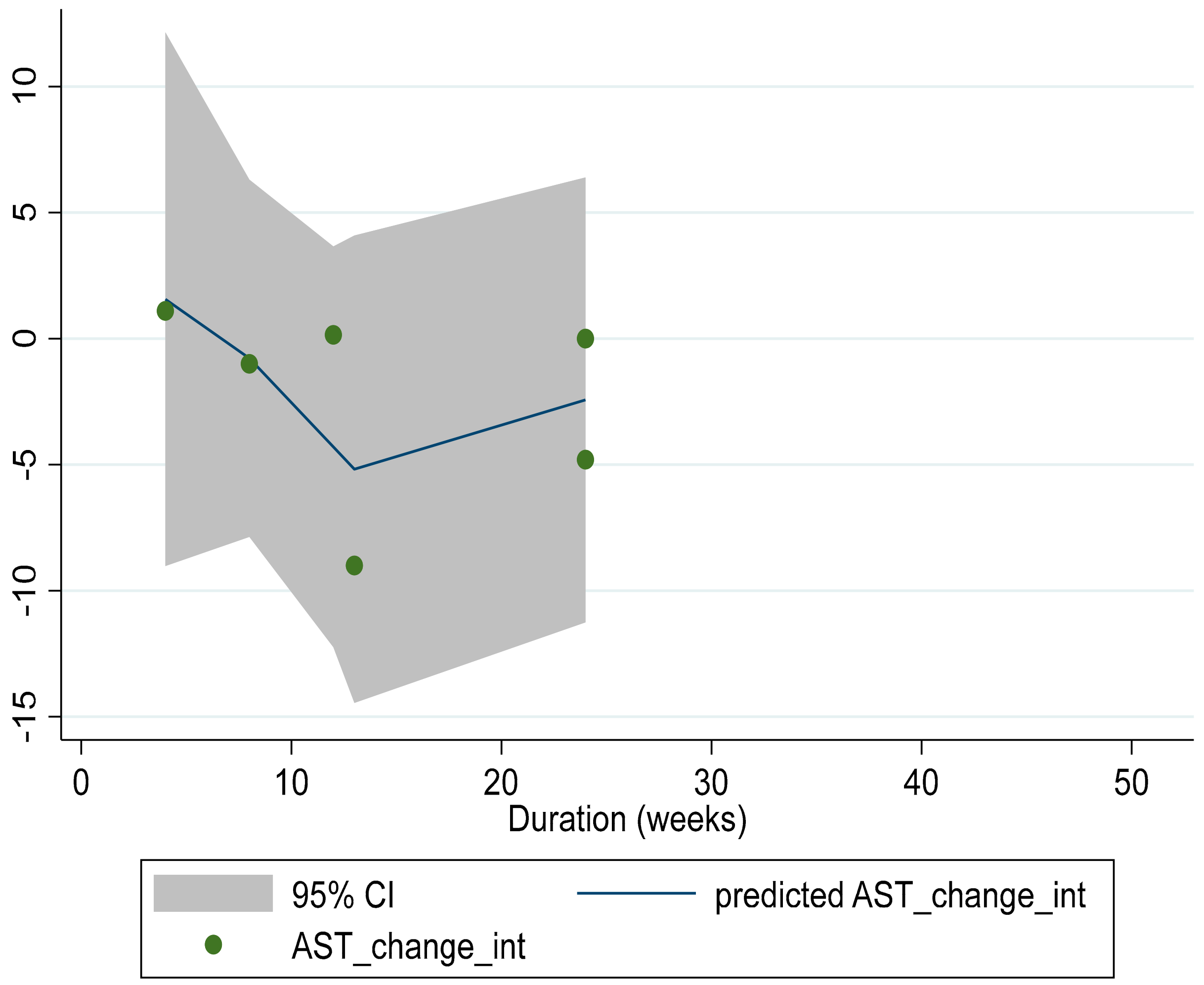

T) ALT

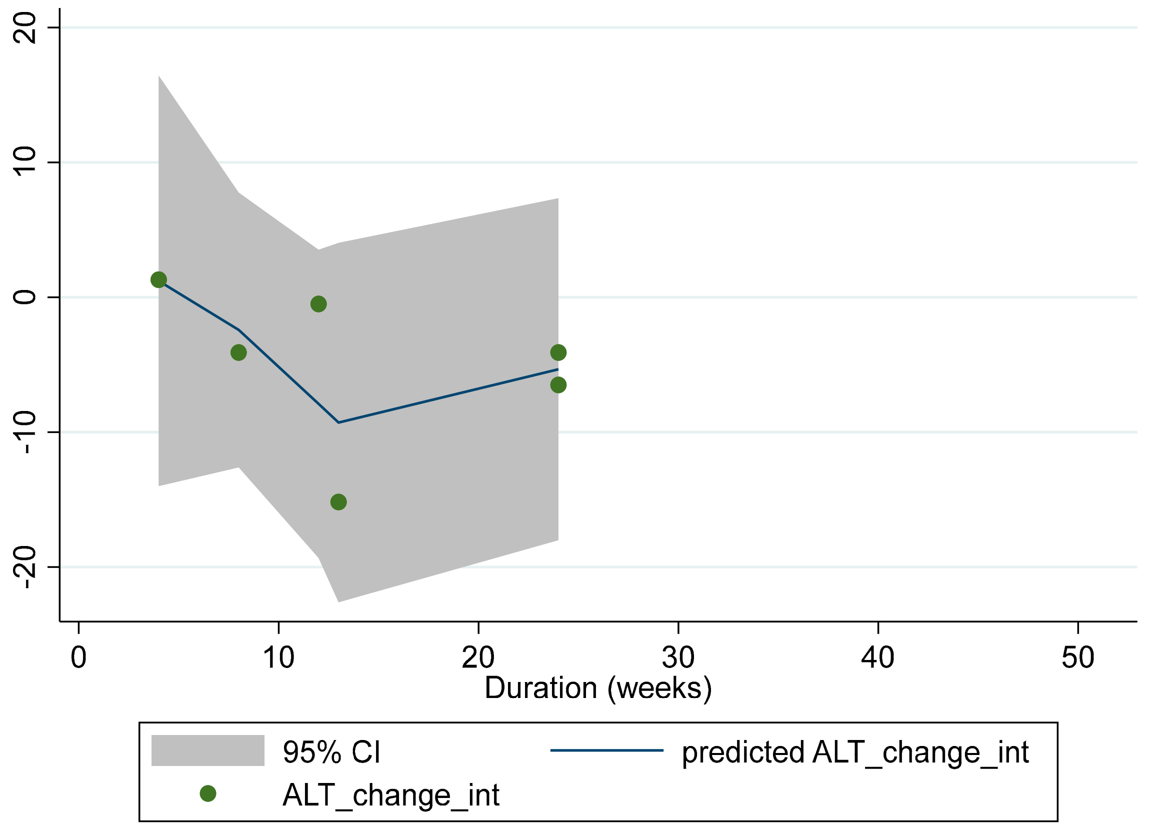

Figure S5. *Cont.*

U) Cr

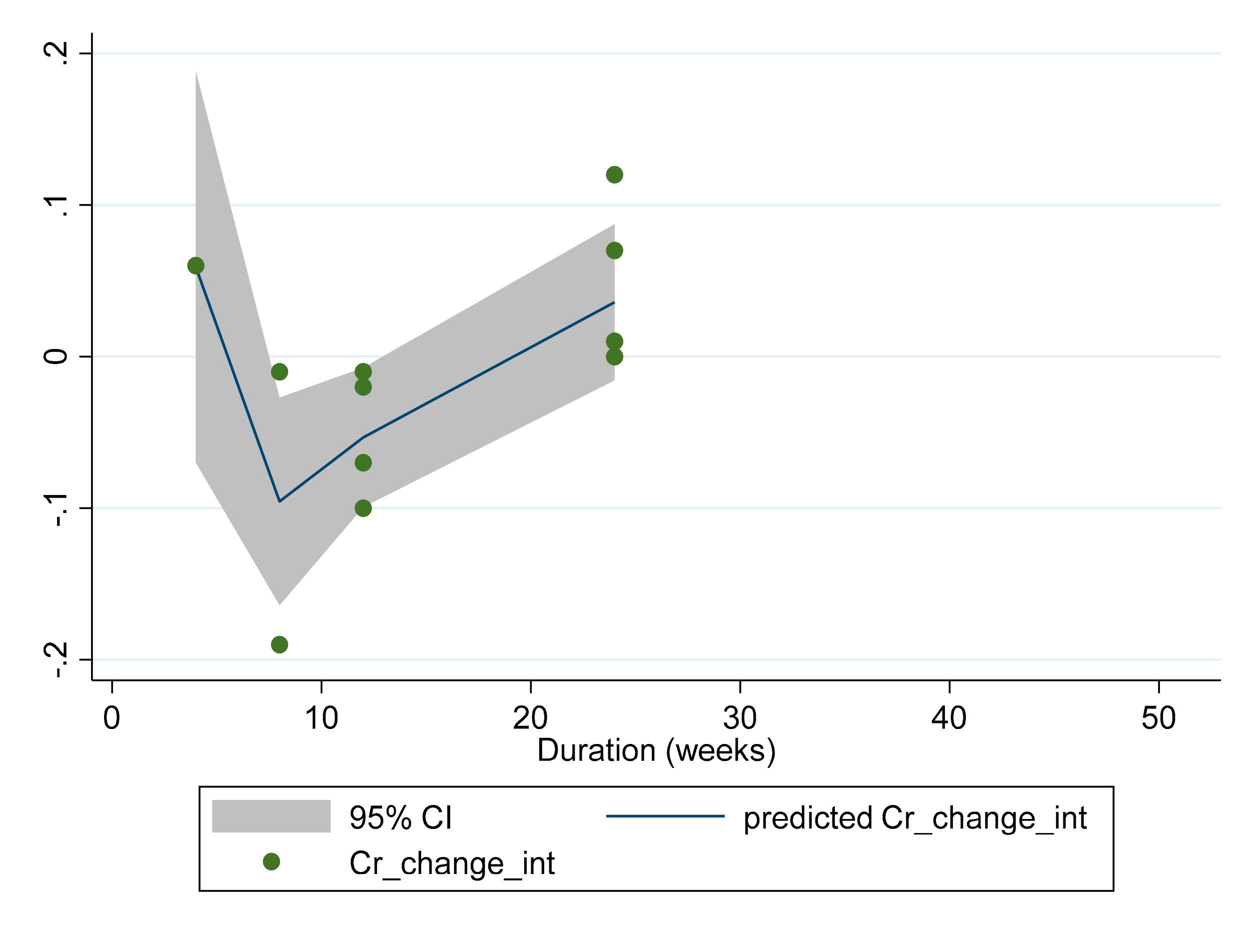

Figure S5. Non-linear association between duration (weeks) of Mg supplementation and mean changes in cardiometabolic risk factors (CMRFs), including **(A)** BW (kg), **(B)** BMI (kg/m²), **(C)** WC (cm), **(D)** HC (cm), **(E)** BFP (%), **(F)** FBG (mg/dL), **(G)** HbA1c (%), **(H)** FI (μIU/mL), **(I)** HOMA-IR, **(J)** TG (mg/dL), **(K)** TC (mg/dL), **(L)** LDL-C (mg/dL), **(M)** HDL-C (mg/dL), **(N)** SBP (mmHg), **(O)** DBP (mmHg), **(P)** CRP (mg/L), **(Q)** IL-6 (pg/mL), **(R)** TNF-α (pg/mL), **(S)** AST (IU/L), **(T)** ALT (IU/L), and **(U)** Cr (mg/dL).
